# Prevalence estimation of Italian ovine cystic echinococcosis in slaughterhouses: A retrospective Bayesian data analysis, 2010–2015

**DOI:** 10.1371/journal.pone.0214224

**Published:** 2019-04-01

**Authors:** Federica Loi, Paola Berchialla, Gabriella Masu, Giovanna Masala, Paola Scaramozzino, Andrea Carvelli, Vincenzo Caligiuri, Annalisa Santi, Maria Cristina Bona, Carmen Maresca, Maria Grazia Zanoni, Gioia Capelli, Simona Iannetti, Annamaria Coccollone, Stefano Cappai, Sandro Rolesu, Toni Piseddu

**Affiliations:** 1 Osservatorio Epidemiologico Veterinario Regionale, Cagliari, Italy; 2 Dipartimento di Scienze Cliniche e Biologiche, Università degli studi di Torino, Torino, Italy; 3 Istituto Zooprofilattico Sperimentale della Sardegna “G. Pegreffi”, Sassari, Italy; 4 Istituto Zooprofilattico Sperimentale del Lazio e della Toscana, Roma, Italy; 5 Istituto Zooprofilattico Sperimentale del Mezzogiorno, Portici – Napoli, Italy; 6 Istituto Zooprofilattico Sperimentale della Lombardia Emilia Romagna "B. Ubertini", Brescia, Italy; 7 Istituto Zooprofilattico Sperimentale del Piemonte, Liguria e Valle d’Aosta, Torino, Italy; 8 Istituto Zooprofilattico Sperimentale dell’Umbria e delle Marche, Perugia, Italy; 9 Istituto Zooprofilattico Sperimentale delle Venezie, Legnaro - Padova, Italy; 10 Istituto Zooprofilattico Sperimentale dell’Abruzzo e del Molise “G. Caporale”, Teramo, Italy; University of Illinois, UNITED STATES

## Abstract

Cystic echinococcosis (CE) is a complex zoonosis with domestic and sylvatic life-cycles, involving different intermediate and definitive host species. Many previous studies have highlighted the lack of a surveillance system for CE, its persistence in Italy, and endemicity in several Italian regions. Because of the absence of a uniform surveillance program for both humans and animals, disease occurrence is widely underestimated. This study aimed to estimate the prevalence of ovine CE in Italy. Survey data on the prevalence of *Echinococcus granulosus* complex infections in Italian sheep farms from 2010 to 2015 were obtained in collaboration with Regional Veterinary Epidemiology Observatories (OEVRs). Bayesian analysis was performed to estimate the true CE farm prevalence. The prior true CE prevalence was estimated using data from Sardinia. Second, Bayesian modelling of the observed prevalence in different regions and the true prevalence estimation from the first step were used to ultimately estimate the prevalence of ovine CE in Italy. We obtained survey data from 10 OEVRs, covering 14 Italian regions. We observed that the risk of CE infection decreased over the years, and it was strictly correlated with the density of susceptible species. Using Sardinia as prior distribution, where the disease farm prevalence was approximately 19% (95% CI, 18.82–20.02), we estimated that the highest endemic CE farm prevalence was in Basilicata with a value of 12% (95% BCI: 7.49–18.9%) and in Piemonte 7.64%(95% BCI: 4.12–13.04%). Our results provide spatially relevant data crucial for guiding CE control in Italy. Precise information on disease occurrence location would aid in the identification of priority areas for disease control implementation by the authorities. The current underestimation of CE occurrence should urge the Italian and European governments to become aware of the public health importance of CE and implement targeted interventions for high-risk areas.

## Introduction

**Fig 1:**
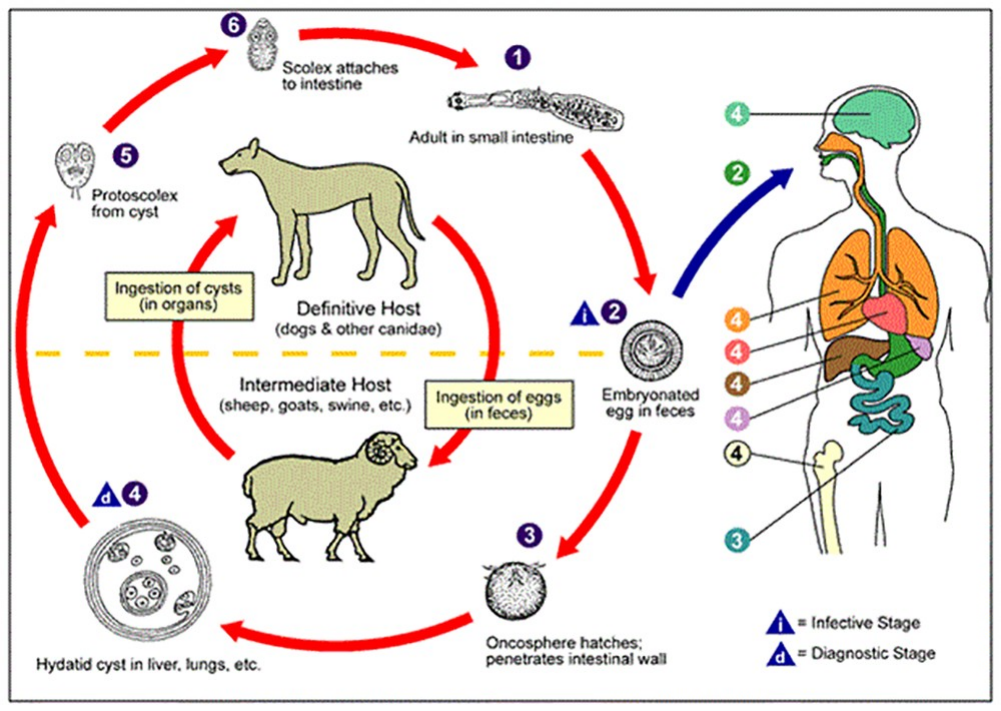
Life-cycle of cystic echinococcosis, involving definitive hosts (Canidae species), intermediate hosts (sheep and dogs) and humans.

The World Health Organization (WHO) defines zoonosis as any disease or infection that is naturally transmissible from vertebrate animals to humans, and *vice versa* [1]. Cystic echinococcosis (CE), or hydatid disease, is a cosmopolitan parasitic zoonosis caused by taeniid tapeworms (cestodes)–only a few millimetres long–of the *Echinococcus granulosus* complex. *E*. *granulosus* have indirect life-cycles involving different host species, as shown in Fig 1. Carnivores, such as dogs and wolves, are the definitive hosts that harbour the adult parasite in their intestines and commonly shed *E*. *granulosus* eggs through their faeces. Intermediate hosts harbour the larval stage of the parasite and include several farm animals, mainly sheep and cattle. Humans are accidental intermediate hosts. *Echinococcus granulosus* sensu stricto (s. s.) (G1-3) is responsible for the great majority of human CE worldwide (88.48%), has the most cosmopolitan distribution and is often associated with transmission via sheep as intermediate hosts [2]. In intermediate hosts, the ingestion of *E*.*granulosus* s.s. eggs leads to the development of one or several fluid-filled cysts located mainly in the liver and lungs and less frequently in other organs [3]. The cysts can disrupt the function of the infected organs, causing poor growth, reduced milk and meat production in ovine, and even organ rejection following meat inspection [4]. Clinical signs of CE may occur after a highly variable incubation period from several months to years [5]. Disease diagnosis in livestock is mainly based on *post-mortem* slaughterhouse inspection. Furthermore, the rate of cyst development is variable and has been estimated at approximately 1–5 cm per year [6] [7]. Consequently, as observed by Liu in 1993 [8], early CE lesions can be missed during inspection, making the sensitivity (*Se*) of diagnosis quite low. The meat inspection specificity (*Sp*) may also be affected due to lesion similarity caused by different infections [1] [9] [10]. Due to these reasons, CE detection should be strictly correlated to the age of the animal. A complete review of CE diagnosis and detection was published first by Craig et al. in 2015 [11] [12], providing specific results of diagnostic test performance, with respect to *Se* and *Sp*. In humans, after many years of initial asymptomatic incubation, the disease can be severe, and occasionally fatal if left untreated. Moreover, CE treatment can be lengthy and expensive [5] [13] [14] [15]. The diagnosis of CE in humans is based on different imaging techniques, such as computed tomography scan, ultrasonography, magnetic resonance [16]. Moreover, the long duration of the asymptomatic period and the associated nonspecific symptoms, contribute to the massive underreporting of CE, both in humans and animals. Thus, human clinical cases can be defined as ‘the tip of the iceberg’ with regards to CE prevalence. The highest prevalence rates among humans and animals occur where livestock production is extensive, where large numbers of dogs are kept (i.e. for guarding livestock), and where dogs have access to dead livestock carcasses or offal derived from uncontrolled slaughter [17] [18]. The annual Community Summary Report produced by the European Food Safety Authority (EFSA) and the European Centre for Disease Prevention and Control (ECDC) highlighted that a surveillance system for human CE is absent in Italy and epidemiological data regarding livestock are incomplete [19]. In 2014, the results of an 11-year retrospective analysis were published by the Italian National Reference Centre for Echinococcosis (CeNRE), based on hospital discharge records (HDRs) for CE cases drawn from the Ministry of Health. The analysis showed that in Italy the incidence of human CE hospitalisations is approximately 1.4/100.000 inhabitants, presenting a considerable socio-economic burden [13]. In this regard, it should be noted that even if HDRs are not exhaustive, they are often the only available source for detecting CE cases in humans [14]. As shown by Possenti in 2016, human and animal CE cases seem to be strictly associated with farm owner demographics (i.e. age, educational level) [20]. Wang in 2006 and Torgerson in 2010 demonstrated that human infection depends on personal hygiene and socio-economic variables that favour close contact with *Echinococcus* spp. eggs and their inadvertent ingestion [21] [22]. The latest systematic-review and meta-analysis of the current year confirmed this association [23]. Although Italian legislation (European Directive 2003/99/CE, Italian National Decree n°19, 4^th^ April, 2006) encourages data collection regarding the incidence and the type of zoonotic agents found in food, animals or humans, ovine CE is largely underreported, as it is solely recorded through slaughterhouse reports. Across Europe, the actual prevalence of CE in animals or humans remains unclear, partly due to the lack of efficient and dedicated reporting systems [15]. As the transmission cycle of CE may involve different species and may vary between territories, identification of specific risk factors, which play a role in this transmission may be difficult [24]. The aim of this work, carried out by CeNRE in collaboration with 10 Regional Veterinary Epidemiology Observatories (OEVRs), is to create an Italian epidemiological network that focuses on data collection and sharing among the 74 Italian provinces (listed in S1 Table). The collection of such essential ovine data from slaughterhouses regarding CE positivity in Italy will ultimately aid in the estimation of disease prevalence in sheep at a regional level. Accurate and robust CE prevalence estimation is paramount for the ongoing widespread public CE awareness campaign and the future development of active surveillance-sampling disease control measures.

## Material and methods

### Study area and data collection

The Ministry of Health, with the Reference Centre for Veterinary Epidemiology (COVEPI), in compliance with Directive 2003/99/EC have designed and implemented the National Information System of Zoonosis (SINZOO). The system collects epidemiological data periodically, including annual disease prevalence data, which need further validation by experts on the relevant zoonosis. Critical evaluation of the COVEPI platform data reveals that EC positivity in sheep slaughtered in the Italian regions is underreported, especially in Central and Southern Italy. Due to this lack of data, the accurate representation of parasitosis distribution in Italy is impossible. To fill this information gap, the CeNRE conducted a research project funded by Ministry of Health in 2016. The project involved the collaboration and coordination of all the Animal Health Epidemiological Observatories in the Italian National Network (Istituti Zooprofilattici Sperimentali—IZS) that adhere to the SINZOO platform. A complete list of all the Regional Veterinary Epidemiology Observatories (OEVRs), regions and provinces covered in this study are listed in S1 Table. According to the study protocol, CeNRE and OEVRs were committed to collect data regarding ovine CE prevalence in slaughterhouses, which would provide invaluable support in ovine disease prevalence estimation. For this, slaughterhouses widely distributed throughout Italy, or the ones receiving animals from large territorial areas were selected, in order to ensure a representative regional coverage. According to the World Organisation for Animal Health (OIE) Manual of Diagnostic Tests and Vaccines for Terrestrial Animals (Terrestrial Manual, 7^th^ edition, 2012), CE detection was performed by visual inspection, palpation and incision, during meat inspection (either in an abattoir or prior to consumption/sale), since *E*. *granulosus* infection is most commonly diagnosed at necropsy. For this study, an *ad hoc* database was created for Sardinian and Italian slaughtered CE positive farms (S2, S3 and S4 Tables). CeNRE provided each OEVR with a specific data collection Excel worksheet template for the uniform collection of the following data: the identification of the Local Sanitary Agency (ASL) that carried out the inspection and its province, the slaughterhouse code, the date of slaughter, the slaughtered species, the farm code of the slaughtered head and the animal code. Since only 20–25% of sheep farms move animals for slaughter, and assuming that a single CE positive sheep is indicative of a positive farm, CE prevalence was estimated as the sum of positive farms over the total number of farms that moved sheep to slaughterhouses, by province. The entire census farm population was assumed to be susceptible to the disease due to the lack of information on animal-specific prevalence and low disease frequency. Data regarding ovine CE positive farms observed during slaughterhouse inspection constitute the analysis outcome. The CE prevalence outcomes were related to all well-known factors that could be involved in the disease cycle. For each Italian province, data regarding the number of farms that move animals for slaughter and data of animal population census (expressed as the total number of animals in each province) were collected annually, using the National Italian Database (BDN) established by the Ministry of Health at the National Surveillance Center of the IZS in the Abruzzo and Molise region. Hospital discharge records (HDRs) for CE cases were provided to CeNRE by the Italian Ministry of Health. HDRs are widely used in epidemiological studies since they contain anonymised individual patient codes for tracking hospital admissions and discharges. HDRs include, patient admission and discharge dates, age, gender, domicile code, primary and secondary diagnoses codes according to the International Classification of Diseases, Ninth Revision, Clinical Modification (ICD-9-CM), type of treatment (surgical or medical), length of hospitalisation and mortality data. Hospital-related data include, regional and province codes, department, ward and type of hospitalization, including ordinary hospitalization (OH) or day hospitalization (DH). Patients with discharge code ICD-9 122.0–122.4 and 122.8–122.9 were associated with CE cases diagnosis thus, allowing for the identification of CE-related hospitalisations [25]. In order to investigate further the association between farm owner demographics and CE positivity, the farmers’ age, sex, educational levels, type of farm, and socio-economic factors were included in the analyses. Specifically, farm owner demographics were collected through the BDN and the National Institute of Statistics for Agriculture (Agri-ISTAT, http://dati-censimentoagricoltura.istat.it/Index.aspx?lang=it), while the assessed socio-economic factors included were the territorial indicators for developmental polices, as released by the National Institute of Statistics (ISTAT) (http://www.istat.it/it/archivio/16777). Because not all indicators were available for every territorial level, only those released at the province level were considered for this study. Table 1 shows the complete list of collected variables in relation to their source.

**Table 1:** **List of collected variables, in association with variable definition and macroarea of argument based on source of collection:** Slaughterhouse, National Italian Database (BDN), hospital discharge records (HDRs), and National Institute of Statistics (ISTAT).

VARIABLE COLLECTED	VARIABLE DEFINITION	MACROAREA OF ARGUMENT
ASL	Local Sanitary Agency	Ovine CE—slaughterhouse
ID Slaughterhouse	Identification of slaughterhouse	Ovine CE—slaughterhouse
Slaughter Date	Data in which animals were slathered	Ovine CE—slaughterhouse
Species	Species of animal slathered	Ovine CE—slaughterhouse
ID animal	Animal code	Ovine CE—slaughterhouse
ID farm	The farm code of origin	Ovine CE—slaughterhouse
N. of farms to slaughterhouse	The number of farms which move to slaughterhouse, in each province and by year	Ovine CE—BDN
N farms	The number of total farms in each province, by year	Ovine CE—BDN
N. animals	The number of total animal (susceptible species) censed in each province, by year	Ovine CE—BDN
Age of the farmer	In year	Agri-ISTAT
Sex of the farmer	Male/Female	Agri-ISTAT
Human cases	Number of human CE positive in each province, by year	Human CE—HDRs
Admission dates	Patient’s admission dates	Human CE—HDRs
Discharge date	Patient’s discharge dates	Human CE—HDRs
Age	Patient’s age	Human CE—HDRs
Gender	Patient’s gender	Human CE—HDRs
Domicile code	Patient’s residence address	Human CE—HDRs
Primary DC	Codes associated to primary diagnosis records	Human CE—HDRs
Secondary DC	Codes associated to secondary diagnosis records	Human CE—HDRs
Length of stay	Length of stay in the hospital for each admission	Human CE—HDRs
Exitus	Mortality data, if patient dead in hospital	Human CE—HDRs
Treatment	Type of treatment can be surgical (S) or medical (M)	Human CE—HDRs
Regional hospital code	ISTAT code	Human CE—HDRs
Province hospital code	ISTAT code	Human CE—HDRs
Department code	ISTAT code	Human CE—HDRs
Type of hospitalization	Hospitalization can be ordinary hospitalization (OH) or day hospitalization (DH)	Human CE—HDRs
Burnt forests (Ind_255)	Forest surface covered by fire over total forests area (km^2^)	ISTAT—Environment
Flood risk population (Ind_278)	Inhabitants flood risk exposed (by km^2^)	ISTAT—Environment
Cultural demand (Ind_018)	Number of visitors to antiquity and art national institutes of by state institute (thousands)	ISTAT–Cultural heritages
Degree of promotion of the cultural offer of state institutions (Ind_024)	Paying visitors on non-paying visitors to state institutes of antiquities and art with paid admission (%)	ISTAT–Cultural heritages
Weight of cooperative society (Ind_120)	Employees of cooperative companies on the total number of employees (%)	ISTAT–Social capital
Air quality monitoring (Ind_265)	Equipped with air monitoring stations / 100.000 inhabitants	ISTAT–Cities
Enrollment rate in the business register (Ind_242)	Companies registered fewer companies ceased on the total number of companies registered in the previous year (%)	ISTAT–Business demographics
Ability to export in sectors with dynamic global demand (Ind_168)	Share of the value of exports in sectors with dynamic global demand on total exports (%)	ISTAT—Internationalization
Unemployment rate (Ind_012)	15 aged and over job seekers on the workforce in the corresponding age group (%)	ISTAT—Work
Employment rate (Ind_013)	15–64 years aged employed people on the population in the corresponding age group (%)	ISTAT—Work
Difference between male and female employment rate (Ind_057)	Absolute difference between 15–64 years male and female employment rate (%)	ISTAT—Work
Participation of the population in the labor market (Ind_108)	Labor force aged 15–64 years of the total population aged 15–64 years (%)	ISTAT—Work
Rate of reported thefts (Ind_279)	Reported thefts /1.000 inhabitants	ISTAT–Legality and security
Rate of reported robberies (Ind_280)	Reported robberies/1.000 inhabitants	ISTAT–Legality and security
Homicides rate (Ind_281)	Voluntary homicides/1.000 inhabitants	ISTAT–Legality and security
Micro criminality index (Ind_134)	Crimes linked to petty crime in cities /1.000 inhabitants	ISTAT–Legality and security
Funding risk (Ind_162)	Decay rate of cash loans (%)	ISTAT—Finance
Separate municipal waste (Ind_052)	Urban separate waste on total urban waste (%)	ISTAT—Garbage
Municipal waste (Ind_083)	Kg of urban waste collected/ 1 inhabitant	ISTAT—Garbage
Elderly in social assistance (Ind_415)	Elderly treated in social assistance on the total elderly population (65 years and over) (%)	ISTAT—Health care
Childhood services (Ind_142)	Percentage of Municipalities that have activated childcare services (nursery school, micronids or supplementary and innovative services) out of the total number of municipalities in the province	ISTAT—Health care
Taking charge of all users of childcare services (Ind_414)	Children between 0–3 years who have used childcare services (nursery, micronids, or supplementary and innovative services) on the total population aged 0–3 years (%)	ISTAT—Health care
Hospital emigration (Ind_141)	Hospital emigration to another region for acute ordinary hospitalizations of the total hospitalized persons residing in the region (%)	ISTAT–Health care
Tourism in not-summer period (ind_165)		ISTAT—Tourism
Tourism rate (Ind_105)		ISTAT—Tourism

### Statistical analysis

**Fig 2:**
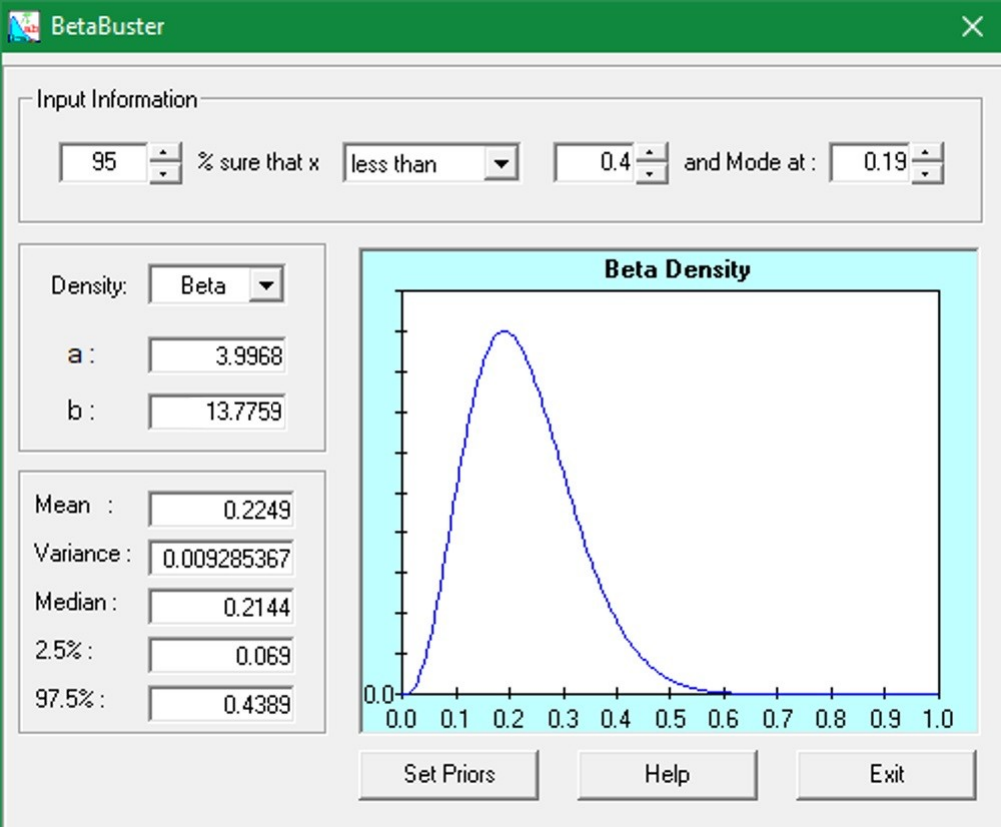
BetaBuster software output derived from mode of 0.19 and 95% certainty of the value being less than 0.4. Obtaining beta parameters (3.9968, 13.7759).

After data collection regarding CE slaughter prevalence by the OEVRs (S2 Table), a database was created. Data quality was assessed in terms of accuracy and completeness. Based on the completeness of the collected information, one Italian region was defined as more ‘truthful’, given its relatability to the real-world outcomes. Data analyses aimed to define the parameters of the ovine CE prior distribution based on the most reliable regional information. Descriptive statistical analysis (Table 2) was performed to evaluate the baseline distribution of each variable and to evaluate the amount of missing data (Fig 2). The majority of the collected variables were quantitative and expressed as mean values with standard deviation (SD) or median values with interquartile range (IQR). Categorical variables were described as frequencies (n) and percentages (%). The nature of the association between the outcome (CE farm prevalence) and each of the continuous independent variables was assessed. When a linear relation was assumed, bivariate analysis was performed to evaluate the strength of the association, by means of the Spearman’s non-parametric correlation coefficient to avoid collinearity that could lead to wrongful estimation [26]. Variables with correlation coefficient greater than 0.8 were excluded from the analysis. Bayesian variable selection was carried out to identify the most important predictors of disease risk using the BayesVarSel package in R-software [27]. In BayesVarSel, the most probable models and their probabilities are viewed by printing the created object (ob), while the summary displays a table with the inclusion probabilities. The package provides the model most supported by the information (data and prior) called highest posterior model (HPM) and the median probability model (MPM), which is the model containing the covariates with inclusion probability larger than 0.5. The use of HPM and MPM was studied by Barbieri and Berger (2004), who demonstrated that in certain situations, these are optimal for prediction [28]. The posterior distribution of the model size can be plotted and all possible models are saved in the ob$modelslogBF matrix, which holds the Bayes factors of each model in logarithmic scale. For the inclusion/exclusion probabilities, the ‘Robust" prior distribution recently proposed by Bayarri et al. (2012) was chosen to produce Bayes factors with closed-form expressions [29]. The highest number of most probable models to be kept was set as 10. All models generated from the combinations of all the potential predictors were fitted and the predictors that were included in more than 50% of the models were considered as important (S5 Table). Data from the ‘truthful’ region were fitted to a linear regression model against CE survey data to obtain an explicit estimate of the infection risk. The predictors selected from the variable selection procedure were included in the multivariable model. Although count data often follow a Poisson distribution, the overdispersion was tested with the likelihood ratio of the alpha parameter [30]. The model that best fitted the data was the negative binomial regression model (NBRM), widely used for count-derived outcomes and suitable for dealing with situations of excessive data overdispersion. NBRM (Eq (1)) is a type of generalised linear model, in which the dependent variable Y is a count of the number of times an event occurs. A convenient parameterisation of the negative binomial distribution given by Hilbe [31] is derived from a Poisson-gamma mixture. The count of CE positive animals Y_ij_ of an Italian province j in year i = 1, …, N, is modelled as a negative binomial variation with mean μ_ij_ and can be described as:

$$\text{Pr}\left(y_{i}|x_{i}\right)=\frac{\Gamma ({{k+y}_{i}}_{j})}{{{\Gamma (k)y}_{i}}_{j}!}\frac{k^{k}\mu_{ij}^{y_{ij}}}{{\left(k+\mu_{ij}\right)}^{k+y_{ij}}}$$ (1)

where $\Gamma (t)=\int_{0}^{\infty }x^{t-1}e^{-x}dx$ is the gamma function. Since var(Y_ij_) = μ_ij_ + μ^2^_ij_/ k, it is clear that the second parameter of the negative binomial distribution k > 0 incorporates extra-Poisson variation. Larger values of k correspond to less variability, with the limiting case k = ∞ corresponding to the Poisson distribution. A step-wise analysis was performed, and all non-statistically significant variables (*p* ≥ 0.10) were excluded from the NBRM. Results of this model are presented as β coefficient estimation, confidence intervals (95% CI) and *p*-values. The study outcome of this model was the annualised number of CE infected animals per Italian province. A Bayesian statistical analysis of prevalence was chosen since it allows incorporating previously collected data and expert-elicited information into the current calculations. Furthermore, it provides a practical alternative for data analysis when the sample size for prevalence surveys is small [32]. Results of the regression model, based on truthful region data, were used to set up the prior distribution parameters. With a binomial *(n*, *θ)* likelihood, a *beta(a*, *b)* prior for *θ* results in a *beta(x+a*, *n+b+x)* posterior for *θ*, where *x* represents the number of positive cases on *n* controls. Posterior inference for *p* was described by using the median and 95% credible interval of the posterior distribution. The Bayesian model herd prevalence estimation proposed by Messam et al. in 2008 was used [33]. Since a herd is defined as any cluster or grouping of animals, often defined by location [34] [35], for the present study we defined the group of sheep farms in a specific province as the reference group. The province (or province-level) prevalence (*PPS*) of CE was defined as the proportion of infected farms in province slaughterhouses over the total number of farms that move animals to slaughterhouse, in each province:

PPS=numberofinfectedfarms/totalnumberoffarms−slaughterhousesintheprovince.

**Fig 3:**
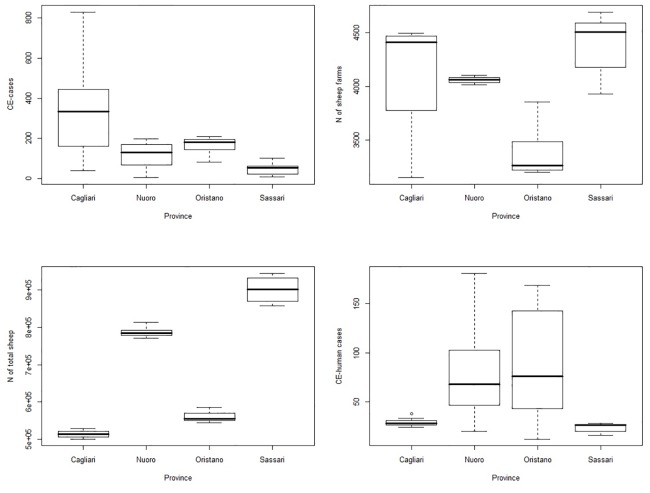
Baseline distribution of Sardinian farm main characteristics, including number of infected farms, number of total sheep, number of total sheep farms, and number of CE cases in the human population, by province.

For the purpose of this study some terms require further definition: the apparent prevalence (*PA*) is the probability of a randomly-chosen unit of observation to test positively [36] and therefore is dependent on the true population within-province prevalence (*PT*) and *Se* and *Sp* of the diagnostic test used. In the Bayesian setting with binomial sampling, we modelled *PT* independently and identically distributed according to a common prevalence distribution, assuming that *Se* and *Sp* are invariant from province to province. The number of animals that test positive in each province is modelled as *x*_*i*_*~bin(n*_*i*_, *PT*_*iSe*_ *+ (1− PT*_*i*_*)(1 − Sp))* with independent beta priors for *Se* and *Sp*. Each *PT* is modelled according to a mixture distribution that models zero infection prevalence with probability *1− PP*_*T*_ where *PP*_*T*_ denotes the province-level prevalence. For infected provinces, *PT* distribution is given by *PT*_*i*_*~beta(μϕ*, *ϕ(1−μ))* where μ is the mean true prevalence in the population and ϕ is a parameter related to the variance of the prevalence distribution that flexibly allows for disperse (e.g. uniformly distributed) or very similar infection prevalence among provinces [37]. To specify values for the hyperparameters *a* and *b* for a *beta(a*, *b)* prior, historical data and/ or expert opinion could be used. All expert opinion came from the veterinarian responsible for each OEVR. For example, to incorporate expert opinion for apparent prevalence (*PA*), we elicited what the expert believed to be the most likely value. This served as a prior point estimate. Then, with a specifically high probability (usually 0.95 or 0.99), the expert provided a value that *PA* was believed to be greater than (or less than) if the prior mode was greater than (or less than) 0.5. The prior mode and outer percentile (95th or 99th) were inputed into the software, e.g. BetaBuster (http://www.epi.ucdavis.edu/diagnostictests/betabuster.html) and the values *a* and *b* were produced as output. When little was known of the prior testing history of the herd, a non-informative prior (i.e. beta(1,1)) could be used, which prescribed equal likelihood to every possible value of the prevalence. An example of a BetaBuster output is shown in Fig 3. The two parameters (μ, ϕ) have been modelled based on the truthful region results with beta(3.9968, 13.7759) derived from distribution for μ of prevalence distribution mode = 0.19, with a 95% certainty this value is lower than < 0.4 and gamma (4.524, 0.387) derived from prior distributions for ϕ (variability related parameter) using median of 95th percentile of prevalence distribution = 0.30 and 99% certainty this number is < 0.50. Based on prior information derived from NBRM, on the latest EFSA recommendations [38] [39] (2006, 2009) and in the study of Garippa [40] (2006), 3% of farms in Italy was expected to be infected with a one-side 95% limit of 8% (i.e. with 95% certainty, the proportion of CE infected farms was less than 0.08). BetaBuster yielded a beta(1.5385, 2.2565) prior for the tau parameter, representing *PP*_*T*_. Finally, the two parameters regarding the sensitivity and specificity of the diagnostic test were establish based on the last review proposed by Craig et al. in 2015 [11]: setting the most likely value of *Se* = 0.89, with 95% certainty that Se > 0.55 and the most likely value of *Sp* = 0.76, with 95% certainty that *Sp* < 0.82. The sensitivity and specificity were modelled using beta(6.835, 1.7212) and beta(79.0317, 25.6416), respectively. Bayesian modelling was implemented using R-software ‘BRugs’ [41] and OpenBUGS (version 3.2.3). The model code is reported in S1 Appendix. Inferences were based on 50000 iterations after a burn-in for convergence of 10000 iterations. Results of the posterior probability distributions are summarised by the median and the credibility intervals. All statistical analyses were performed using R-language v.3.4.1 and the libraries ‘lme4’ [42] and ‘BRugs’ [41].

**Table 2:** Descriptive analysis of baseline variables collected in Sardinian provinces from 2010 to 2015. Data are presented as mean (standard deviation [SD]); median (interquartile range [IQR]) per year.

VARIABLE COLLECTED	Cagliari	Sassari	Nuoro	Oristano
**CE slaughterhouse prevalence (%)**	39.46 [39.15–39.83]	9.21 [9.01–9.45]	14.98 [14.87–15.12]	15.72 [15.46–16.02]
**N. CE-cases**	345 (262); 335 [153–449]	48 (32); 55 [15–66]	115 (70); 130 [62–182]	165 (46); 181 [125–198]
**N. farms to slaughterhouse**	733 (258); 752 [698–779]	1355 (536); 1278 [1097–1456]	474 (167); 503 [426–596]	524 (226); 532 [487–570]
**N. farms**	4079 (590); 4413 [3301–4492]	4382 (320); 4509 [3931–4613]	4059 (33); 4063 [4023–4095]	3389 (260); 3262 [3216–3658]
**N. animals**	514330 (10877); 513090 [503645–526263]	900948 (37059); 902157 [857443–943019]	787099 (14139); 784360 [773903–793144]	560724 (14530); 554632 [547266–572325]
**Human cases**	29 (4.8); 28 [25–33]	23 (4.7); 26 [18–27]	81 (55); 68 [26–112]	90 (64); 76 [13–162]
**% female farms owner**	22 (3.7); 21.6 [19.5–25.7]	24 (1.5); 24.6 [22.8–25.7]	26 (1.9); 25.8 [24.2–27.5]	28 (1.3); 28.6 [26.9–29.1]
**Age of farmer**	52.4 (2.2); 52 [50–55]	48.1 (1.8); 48 [47–50]	56.6 (1.5); 56 [55–58]	57.2 (1.8); 57 [56–59]
**Burnt forests (Ind_255)**	3731 (2884); 2795 [1891–7806]	4087 (3160); 3062 [2071–8551]	4757 (3678); 3564 [2411–9952]	1076 (832); 806 [545–2251]
**Flood risk population (Ind_278)**	6.1 ab / km^2^	1.43 ab / km^2^	0.89 ab / km^2^	5.2 ab / km^2^
**Cultural demand (Ind_018)**	21.8 (7.9); 18.2 [17.6–28.4]	9.3 (0.85); 9.3 [8.7–10.3]	3.8 (2.4); 2.9 [2.7–3.4]	24.6 (11.5); 23.7 [10.8–36.1]
**Degree of promotion of the cultural offer of state institutions (Ind_024)**	308 (420); 198 [67–236]	45 (23); 38 [31–41]	73 (75); 40 [35–70]	838 (66); 941 [194–1232]
**Weight of cooperative society (Ind_120)**	6.9 (0.58); 6.8 [6.4–7.7]	2.5 (0.25); 2.5 [2.4–2.8]	3.3 (0.32); 3.2 [3.07–3.7]	9.2 (0.78); 8.9 [8.7–9.5]
**Air quality monitoring (Ind_265)**	3.4 (0.2); 3.4 [3.2–2.5]	3.2 (0.23); 3.3 [3–3.4]	3.5 (0.5); 3.2 [3.1–3.8]	1.9 (0.22); 1.8 [1.7–1.9]
**Enrollment rate in the business register (Ind_242)**	0.52 (0.31); 0.54 [0.31–0.63]	0.82 (0.34); 0.96 [0.44–1.1]	1.3 (2.03); 0.51 [0.20–1.2]	0.88 (0.34); 0.95 [0.49–1.2]
**Export index (Ind_168)**	4.8 (0.85); 5 [4.4–5.2]	30 (7.5), 26 [23–36]	37 (24); 45 [12–59]	6.8 (4.1); 6.4 [5.7–7.8]
**Unemployment rate (Ind_012)**	15 (2.8); 15.5 [12.5–17.7]	16.8 (2); 16.7 [15.9–18.7]	11 (1.9); 10 [9.9–11.7]	17 (2); 17.4 [15.1–19.7]
**Employment rate (Ind_013)**	51.7 (1.35); 52 [50.4–52.7]	50.1 (2.0); 51.2 [47.5–51.7]	51.3 (1.6); 50.7 [50.5–52.8]	50.1 (1.2); 50.4 [49–51]
**Difference between male and female employment rate (Ind_057)**	17.2 (1.9); 16.8 [15.1–18.3]	14.9 (2.5); 15.2 [11.9–17.1]	14.8 (3.9); 16.1 [12.3–18.5]	17.5 (2.3); 18.3 [16.2–19.1]
**Participation of the population in the labor market (Ind_108)**	61.1 (1.6); 60.3 [60–62.5]	60.4 (1.5); 59.6 [59.4–61.7]	57.8 (1.5); 57.7 [56.3–58.2]	60.5 (1.9); 61.2 [58.6–62.3]
**Rate of reported thefts (Ind_279)**	19 (1.5); 19 [17–20]	22 (1.6); 22 [21–24]	16 (1.4); 16 [14–17]	7.8 (0.69); 7.8 [7.2–8.1]
**Rate of reported robberies (Ind_280)**	0.37 (0.05); 0.37 [0.32–0.42]	0.33 (0.04); 0.33 [0.29–0.37]	0.38 (0.12); 0.38 [0.37–0.46]	0.11 (0.02); 0.11 [0.08–0.12]
**Homicides rate (Ind_281)**	0.9 (0.55); 0.89 [0.36–1.43]	1.2 (0.77); 0.91 [0.6–2.1]	5.1 (1.8); 5.6 [3.8–5.7]	1.04 (0.57); 1.21 [0.61–1.22]
**Micro criminalità index (Ind_134)**	5.3 (0.32); 5.2 [5.1–5.6]	4.1 (0.33); 4.0 [3.9–4.5]	3.0 (0.52); 2.9 [2.4–3.6]	1.1 (0.19); 1.0 [0.96–1.3]
**Funding risk (Ind_162)**	3.5 (1.1); 3.1 [2.4–4.6]	4.3 (2.2); 3.1 [2.7–6.6]	4.3 (1.3); 4.8 [2.8–5.4[	2.9 (0.96); 2.5 [2.4–2.8]
**Separate municipal waste (Ind_052)**	49.5 (2.4); 49.5 [46.6–52.1]	43.6 (5.6); 44 [37.7–49.4]	54.2 (5.9); 56.1 [49.9–60]	62.5 (2.3); 63 [60.9–64.9]
**Municipal waste (Ind_083)**	466 (26.6); 458 [438–492]	452 (28.4); 381 [368–394]	356 (28.9); 348 [329–379]	381 (12.3); 381 [369–393]
**Elderly in social assistance (Ind_415)**	1.9 (0.08); 1.9 [1.7–1.9]	2.25 (0.11); 2.2 [2.1–2.3]	2.9 (0.21); 2.8 [2.7–2.9]	3.68 (0.14); 3.7 [3.6–3.8]
**Childhood services (Ind_142)**	50 (5); 50.7 [45.1–51]	31.4 (3.9); 28.8 [27.8–36.4]	35.7 (3.8); 38.5 [32.7–38.5]	22 (2.1); 22.7 [20.5–23]
**Taking charge of all users of childcare services (Ind_414)**	13.6 (2); 13.1 [12.9–13.6]	16 (3.4); 14.5 [14–18.3]	15.2 (2.3); 14.3 [13.9–14.6]	11.6 (1.3); 10.8 [10–13.4]
**Hospital emigration (Ind_141)**	4 (0.24); 4 [3.8–4.3]	4.8 (0.17); 4.9 [4.7–5]	5.7 (0.56); 5.5 [5.2–6.3]	4.9 (0.42); 5.1 [4.4–5.3]
**Tourism in not-summer period (ind_165)**	0.98 (0.13); 0.94 [0.91–1.13]	1.03 (0.09); 1.01 [0.97–1.11]	0.92 (0.25); 0.87 [0.75–1.14]	0.71 (0.076); 0.71 [0.65–0.75]
**Tourism rate (Ind_105)**	4.9 (0.43); 4.8 [4.6–5.3]	5 (0.48); 4.9 [4.7–5.2]	6.4 (0.93); 6.6 [5.9–7.2]	2.8 (0.33); 2.6 [2.5–3.0]

## Results

**Fig 4:**
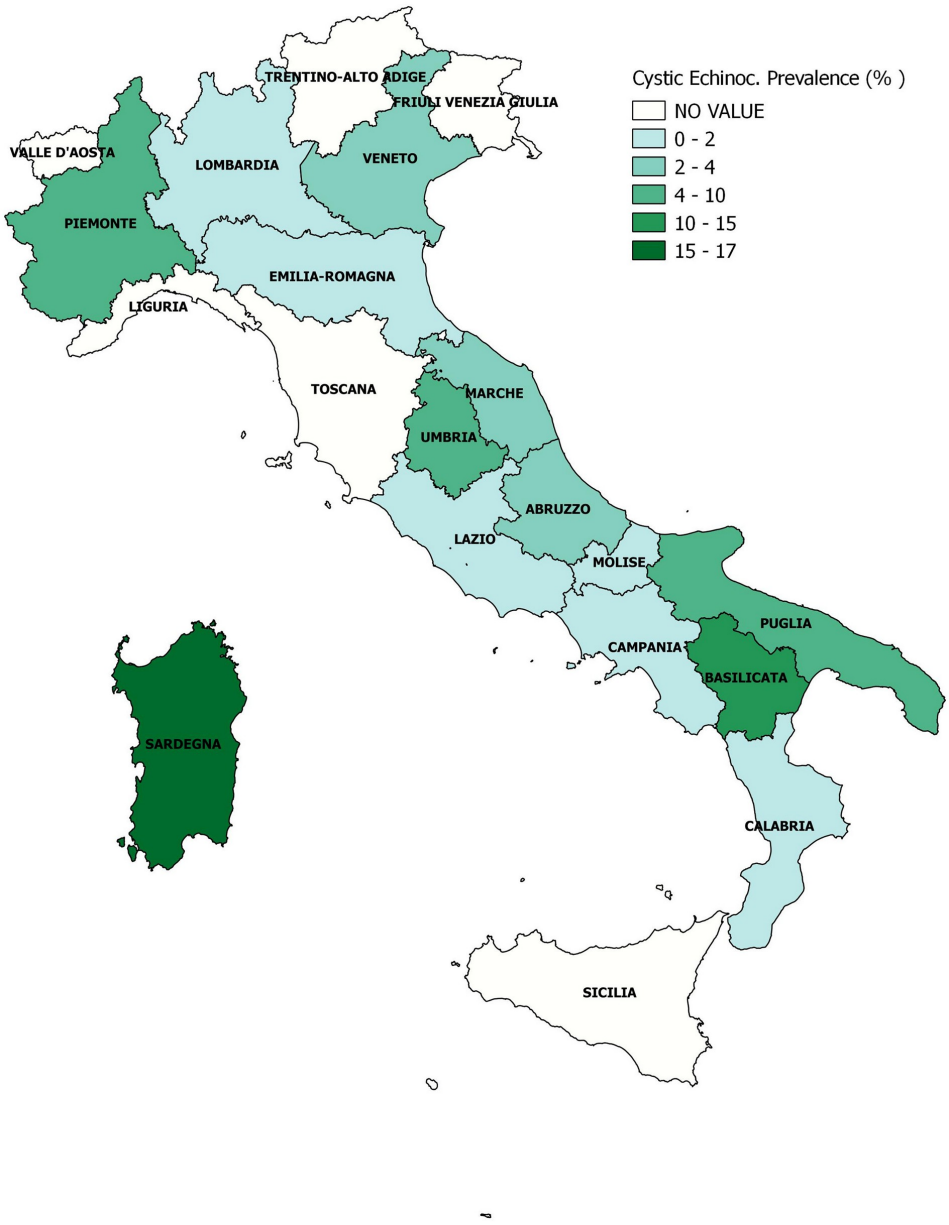
Choropleth map of average sample tau distribution and regional cystic echinococcosis (CE) prevalence estimation by Bayesian modelling.

**Fig 5:**
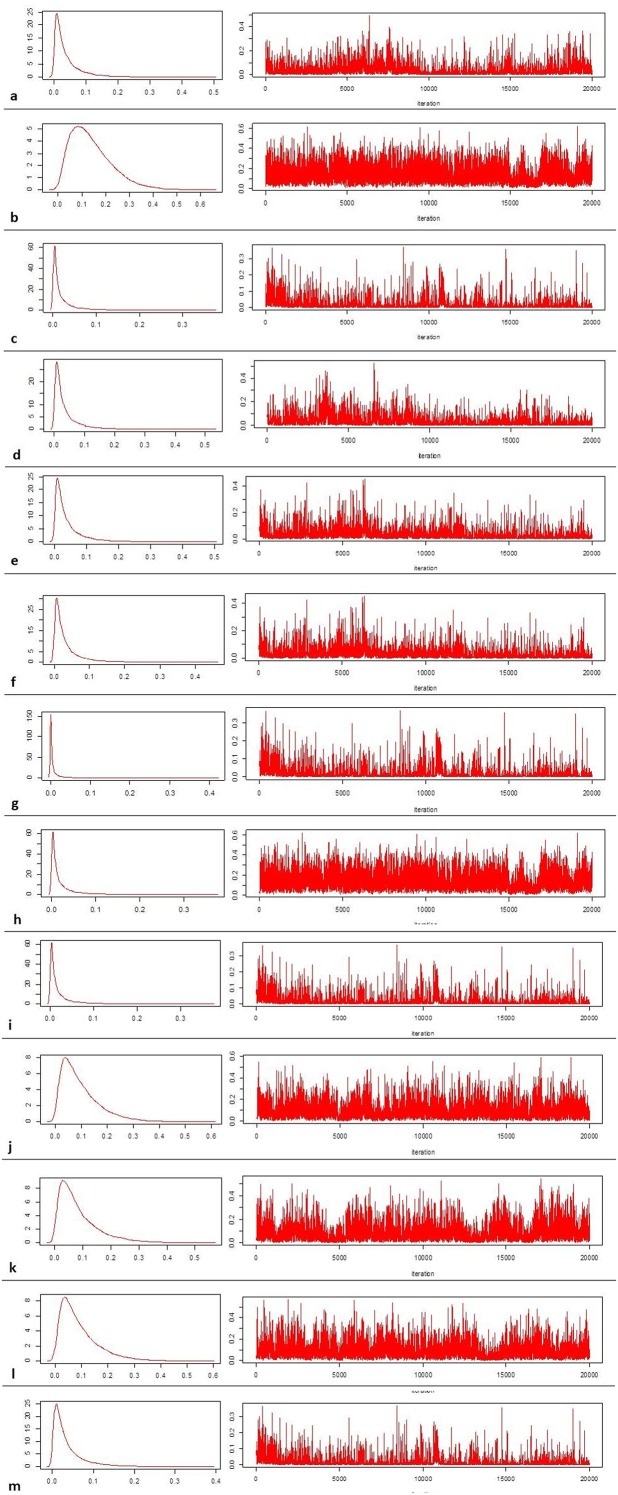
Plots of the median sample tau distribution and regional cystic echinococcosis (CE) prevalence estimation by Bayesian modelling. a. Abruzzo; b. Basilicata; c. Calabria; d. Campania; e. Emilia Romagna; f. Lazio; g. Lombardia; h. Marche; i. Molise; j. Piemonte; k. Puglia; l. Umbria; m. Veneto.

Slaughterhouse data were collected by 10 OEVRs from 2010 to 2015. S2 Table shows the contribution of each OEVR in providing data. Data regarding the number of farms that move ovine to slaughterhouses were available only for the Sardinian region and thus, were used to eliciting the prior distribution, and defining Sardinia as the ‘truthful region’. The baseline distribution of each collected variable in the Sardinian province is described in Table 2 and shown in Fig 2. The number of farms that move animals for slaughter was the highest in the Sassari province (mean = 1355, sd = 536) and the lowest in Nuoro (mean = 474, SD = 167). The largest number of CE infected farms was observed in the Cagliari province (median = 335, IQR [153–449]) and the smallest in the Sassari province (median = 55, IQR [15–66]), while the most frequent human CE cases were reported in the Oristano (median = 76, IQR [13–162]) and Nuoro provinces (median = 68, IQR [26–112]). The number of sheep farms was similar in all provinces, except for Oristano, which had fewer sheep farms. This result is not surprising since historically Oristano is more dedicated to bovine breeding. The number of female farm owners seemed to be greater in the Nuoro and Oristano provinces, while the youngest farm owner population was recorded in Cagliari and Sassari. It is worth noting that the Oristano province has the lowest burnt forest area average (median = 808, IQR [545–2251]), while in Cagliari the highest flood risk per population index was reported (6.1 ab/km^2^), as seen in Table 2 and represented in Fig 4. The Nuoro province presented the lowest cultural demand or cultural heritage, while Oristano and Cagliari had the highest values. Variables related to work seemed to be equally distributed all over the Sardinian region, with an overall slightly higher unemployment rate (11–17%), as compared to the national rate (10%). Data regarding legality and security were variable across provinces. Sassari was the province where theft reported rate was the highest, while Oristano has the lowest rate. Homicides were more frequently reported in Nuoro when compared to other province and, in general, the highest micro criminality rates were reported in the Cagliari province. Health services had a different but expected regional distribution, which matched the age distribution per province: the oldest population was recorded in the Oristano and Nuoro provinces, where elderly social assistance was high (3.7% and 2.9%, respectively), while childcare services were more common in the Cagliari and Sassari provinces, which are populated by younger people on average. In the multivariable analysis, a total of 31 variables were analysed. Some variables (i.e. degree of promotion of the cultural offer of state institutions (Ind_024), elderly in social assistance (Ind_415), municipal waste separation (Ind_052), rate of reported robberies (Ind_280), tourism outside the summer period (Ind_165) were excluded because their correlation coefficient was greater than 0.8. The results of the variable selection, including the probability of inclusion, HPM, MPM and final decision (inclusion/exclusion from the final NBRM), are reported in the supplementary file (S3 Table). Overall, 25 variables were considered for Bayesian variable selection, and 15 of them were included in the final regression model (Table 3). The number of CE infected animals was higher during 2010 and had the tendency to decrease on an annual basis although this result is borderline. The percentage of female farm owners, hospital emigrations and cultural demand were negatively associated with the number of CE cases. When the number of sheep per province increased, or when the density of susceptible species per province was high, the number of CE infections increased approximately by 0.9% and 0.1%, respectively. The age of the farmer seemed to be strictly correlated with the number of CE cases, which increased by 1% per additional year of age. All the provinces where the rate of flood risk, unemployment and amount of urban waste were higher, the incidence of CE infections increased by 2.4%, 0.3% and 1.9%, respectively. Microcriminality index and homicide rates seemed to present borderline risk factors for CE infection. Table 4 shows the model-based predicted risk value of CE for certain Italian regions, based on CE prevalence estimation values obtained by the final model. As reported in Table 2, the observed Sardinian CE prevalence ranged was from 39% (*P* = 39.89%, 95% CI [39.15–39.83]) in Cagliari to 9% in Sassari (*P* = 9.21%, 95% CI [9.01–9.45]). High CE prevalences were estimated and reported in Table 4 for Basilicata (*P* = 12.4%, 95% BCI: 7.49–18.88%), Piemonte (*P* = 7.64%, 95% BCI: 4.12–13.04%) and Umbria (*P* = 7.27%, 95% BCI: 3.91–12.59). Prevalence estimation of 6 farms over 100 checked farms was observed for Puglia (*P* = 6.32%, 95% BCI: 3.26–11.43%). All other regions had a CE prevalence lower than 3%, while in Marche, Abruzzo, and Veneto the disease prevalence was estimated near 2.5% and lower than 2% in Campania (*P* = 1.97%, 95% BCI: 0.88–4.41) and Lazio (*P* = 1.87%, 95% BCI: 0.83–4. 01). CE prevalence of approximately 0.83% was estimated for Calabria (95% BCI: 0.32–2.31%), 0.81% for the Emilia Romagna region (95% BCI: 0.27–2.50%), while the lowest prevalence was estimated in Lombardia (*P* = 0.27%, 95% BCI: 0.09–0.94). The median sample distributions of the tau parameter for all regions, as derived by the Bayesian model, were evaluated for model validation and are shown in Fig 5, in association with each diagnostic plot of the sample median distribution of tau.

**Table 3:** Coefficient estimation, confidence interval and *p*-value arising from negative binomial regression modelling (NBRM), fitted on Sardinian cystic echinococcosis (CE) data. Data are presented as β regression coefficient with 95% confidence interval, and *p*-value.

VARIABLE	β Coefficient	95% CI	p-value
**Year**	-0.122	[-0.243, -0.001]	0.048
**N. farms**	0.009	[0.001, 0.015]	0.002
**N. animals**	0.001	[0.0003, 0.0006]	< 0.0001
**Human cases**	0.006	[0.002, 0.011]	0.043
**Age of the farmer**	0.104	[0.306, 0.178]	0.006
**% female farm owner**	-0.302	[-0.349, -0.252]	< 0.0001
**Flood risk population (Ind_278)**	0.239	[0.122, 0.358]	< 0.0001
**Unemployment rate (Ind_012)**	0.031	[0.003, 0.065]	0.037
**Urban waste (Ind_083)**	0.187	[0.054, 0.321]	0.006
**Micro criminality Index (Ind_134)**	0.177	[-0.002, 0.357]	0.053
**Homicide rate (Ind_281)**	0.182	[0.362, 0.002]	0.047
**Hospital emigration (Ind_141)**	-0.392	[-0.758, -0.027]	0.035
**Cultural demand (Ind_018)**	-0.027	[-0.053, -0.001]	0.044

**Table 4:** Predicted prevalence of cystic echinococcosis in Italian farms stratified by region and presented as median with 95% Bayesian credible interval.

ITALIAN REGION	PREVALENCE (%)
**ABRUZZO**	2.27 (0.95–5.06)
**BASILICATA**	12.38 (7.49–18.88)
**CALABRIA**	0.83 (0.32–2.31)
**CAMPANIA**	1.97 (0.88–4.41)
**EMILIA-ROMAGNA**	0.81 (0.27–2.50)
**LAZIO**	1.87 (0.83–4.10)
**LOMBARDIA**	0.27 (0.092–0.94)
**MARCHE**	2.49 (1.15–5.20]
**MOLISE**	0.81 (0.030–2.29)
**PIEMONTE**	7.64 (4.12–13.04)
**PUGLIA**	6.32 (3.26–11.43)
**UMBRIA**	7.27 (3.91–12.59)
**VENETO**	2.28 (1.08–4.71)

## Discussion

Several OEVRs assisted on this project, over the years, and carried out territorial epidemiological investigations aimed at thoroughly investigating CE prevalence in Italian provinces. It has been well established that data collected from official platforms (i.e. slaughterhouse documents, data from OEVRs, Ministry of Health, EFSA, OIE) are incomplete and do not reflect the real disease distribution in Italy. The present work involved numerous Italian OEVRs to aid in generating an epidemiological picture of CE nationwide. From the collected epidemiological data in all regions, it is evident that CE still presents a significant socio-economic burden at a national level, with a prevalence that ranges in intermediate hosts (sheep) from 0.50 to 9.45%. It is crucial to underline that human CE incidence, here evaluated by HDRs, still remains high (national rate: 1.4 cases/100.000 inhabits; Sardinian region rate: 6.5 cases/100.000 inhabits) and should not be neglected. Other social factors collected by ISTAT that were included in the present analysis further confirmed the association between disease risk and critical rural conditions. Furthermore, the density of susceptible species increased the risk on CE cases. A strict correlation was observed between animal and human CE cases, reflecting the cycle of zoonotic diseases where the human are aberrant hosts. The present work confirms the national distribution of CE demonstrated in previous studies [13] [20] [40] [43], with a higher prevalence in the South of Italy and the islands. While CE research in human cases is possible through the use of administrative data, the limitations in animal research are clear and prominent. This can be appreciated by the difficulty to find up-to-date information in national databases, the limited number of instrumentation for *in vivo* diagnosis (i.e. serological analysis or ecography) and the clinical checks on slaughtered animals as the unique CE surveillance system. The negative spill over effect of this in CE prevalence estimation is the ability to estimate only the farm CE prevalence, since no animal-specific data from single farms is available. The present study, confirms the underestimation of animal CE prevalence in official controls and highlights the need to establish and organise a national CE epidemiological depository that will provide trusted data. The ability of CE to present in several species, should further underline the need of implementing a synchronous system, able to provide information not only on prevalence, but also on the risk of diffusion, for the species involved (humans, dogs, ruminants). CE is a multifactorial disease, and its control requires different approaches. According to the WHO/OIE manual (2001), control for CE can be defined as the ‘development of a program to limit the spread of a specific disease under the control of a recognised authority and on the basis of the law’ [1] [44]. In the present study, in order to overcome this lack of data, an informative Bayesian prior was used, based on the observation of the phenomenon in areas where its registration was more accurate (Sardinian region). Since the historically high CE prevalence in Sardinia over the past 50 years, three prevention campaigns have been implemented (1962, 1978, 1987), aimed at controlling CE and successfully reducing the prevalence of the disease in humans [45]. Disease awareness has been raised in the Sardinian population through sanitary educational programs that are currently in action. Considering that the national farm average is approximately 900–1000 farms by province, it is important to consider that a great number of sheep and goat farms are located in the Sardinian province. The results of this study revealed some critical issues. Firstly, the concept of farm prevalence as defined has several limitations: considering CE positive sheep as indicative of a positive farm, noting that CE prevalence was estimated as the sum of positive farms over the total number of farms (see Material and methods), it is clear that this method does not take into account the number of farms that do not lead ovine to slaughterhouses. In fact in Sardinia and in other regions, the practice of home slaughter for sheep is illegal but widespread and this contributes to the underestimation of prior prevalence distribution. Data regarding the annual number of ovine slaughtered in slaughterhouses is available, but no information on the number of farms is recorded. Furthermore, the number of ovine includes adult and young animals, and disease prevalence is really different depending on animal age, with lambs being free from disease, as the parasite does not have enough time to form cysts in various organs [44]. It is possible to estimate animal prevalence, as the number of CE positive animals per slaughterhouse over the number of animals slaughtered in the same year and province. However, this evaluation is not free of limitations since most slaughtered animals are young, while our CE data concern only adult ovine. For example, from Agri-ISTAT the approximate number of slaughtered ovine in 2015 was equal to 700.040 animals. Considering that the total ovine population in Sardinia is approximately 3.5 million, and that 20% (650.000) are restock ovine herds, 700.040 could be estimated as lambs with some adult animals. Potential limitations of the present study due to the use of Sardinia as prior should also be taken into consideration. Firstly, the higher sensitisation of the breeder population in Sardinia may have led to higher disease awareness. Meanwhile, the historically high CE incidence in the region may have led to increased expertise in *post-mortem* slaughterhouse inspection, as compared to the other regions. Even if the same method is used in each slaughterhouse, it is essentially based on the skill and experience of the operator so the sensitivity and specificity may be relatively different between slaughterhouses. Furthermore, estimations of prevalence leave a considerable margin for error, as compared to the periodical surveillance recording. Our next goal would be the estimation of the real number of farms that lead adult ovine to slaughterhouses and a re-evaluation of farm CE prevalence using this new estimation as denominator. The Bayesian model we developed in this study has taken into account up-to-date data CE for each Italian region involved, corrected against all possible risk factors for error minimisation. This modelled CE estimate provides a good starting point for the future development and implementation of novel health strategies for the epidemiological control of CE in Italy, especially in those regions where the prevalence observed by the slaughtering data is zero.

## Supporting information

S1 Appendix Bayesian Region-Prevalence estimation. (DOCX)

S1 Table List of Regional Veterinary Epidemiology Observatories (OEVRs) involved in the study, related to Italian region and province covered by collecting data of ovine cystic echinococcosis positive cases. (DOCX)

S2 Table Ad hoc database for Sardinian farms resulted CE positive, including year of observation, positive farm code (encripped), data of slaughter, province and Local Sanitary Agency code, species. (DOCX)

S3 Table Ad hoc database for Italian farms resulted CE positive, including year of observation, positive farm code (encripped), data of slaughter, province and Local Sanitary Agency code, species. (DOCX)

S4 Table Contribution of each Regional Veterinary Epidemiology Observatory (OEVR) in data collection, as number of the total farms by the national database and the number of cystic echinococcosis (CE) positive sheep and goats found in slaughterhouses. Data regarding the number of farms that move animals for slaughter were available only for the Sardinian region. All data are stratified by study year.
(DOCX)

S5 Table Posterior inclusion probabilities for the variables assessed in the Bayesian variable selection procedure. (DOCX)
