# Supplementary material for: Prevalence estimation of Italian ovine cystic echinococcosis in slaughterhouses: A retrospective Bayesian data analysis, 2010–2015
Source: PLoS One. 2019 Apr 1;14(4):e0214224. doi: 10.1371/journal.pone.0214224 (PMC6443144; doi:10.1371/journal.pone.0214224)
Supplement: S1 Appendix — (DOCX) [file pone.0214224.s001.docx]

APPENDIX 1. Bayesian Region-Prevalence estimation

| Model |  |
| --- | --- |
| { |  |
| for(i in 1:k) | #Command designating number of loops to be performed |
| { |  |
| y[i]~dbin(ap[i], n) | #Likelihood function for the ith province |
| ap[i] <- tp[i]*SeCE+(1-tp[i])*(1-SpCE) | #Function showing relationship between apparent and true prevalences for ith province |
| tp[i] <- tpstar[i]*pinf[i] | #Function showing relationship between true prevalence and  infection probability for ith province |
| pinf[i]~dbern(tau) | #Prior distribution of probability of infection for ith province |
| tpstar[i]~dbeta(alpha,beta) | #Prior distribution of true prevalence for ith province given  true prevalence >0 |
| } |  |
| SeCE~dbeta(6.835, 1.7212) | #Prior distribution of CE test sensitivity. Mode=0.89, 95% sure SeCE >0.55 |
| SpCE~dbeta(79.0317, 25.6416) | #Prior distribution of CE test speciﬁcity. Mode=0.76, 95% sure SpCE <0.82 |
| tau~dbeta(1.5385, 2.2565) | #Prior distribution for province probability of infection. Mode=0.03, 95% sure <0.08 |
| alpha <- mu*psi | #Relationship of distribution parameters to mean (mu) and psi (variance related parameter) |
| beta <- psi*(1-mu) |  |
| mu~dbeta(3.9968, 13.7759) | #Prior distribution for mean (mu) of prevalence distribution Mode=0.19, 95% sure <0.4, derived from Sardinian data |
| psi~dgamma(4.524, 0.387) | #Prior distribution for psi (variability related parameter). Uses median of 95th percentile of prevalence distribution=0.30 and 99% sure this number is <0.50, derived from Sardinian data. |
| P~dbern(tau) | #Prior distribution of probability of infection for randomly chosen province |
| tpstarP~dbeta(alpha,beta) | #Prior distribution of true prevalence for randomly chosen province given true prevalence >0 |
| tpP <-P*tpstarP | #Function showing relationship between true prevalence and infection probability for ith province |
| a1 <- 1-step(tpP-0.05) | #Probability that a randomly chosen province has true prevalence<0.05 |
| a2 <- 1-step(tpP-0.2) | #Probability that a randomly chosen province has true prevalence<0.20 |
| } |  |
