# Supplementary material for: Prevalence estimation of Italian ovine cystic echinococcosis in slaughterhouses: A retrospective Bayesian data analysis, 2010–2015
Source: PLoS One. 2019 Apr 1;14(4):e0214224. doi: 10.1371/journal.pone.0214224 (PMC6443144; doi:10.1371/journal.pone.0214224)
Supplement: S1 Table — (DOCX) [file pone.0214224.s002.docx]

**S1 Table.** **List of Regional Veterinary Epidemiology Observatories (OEVRs) involved in the study, related to Italian Region and Province covered by collecting data of ovine cystic echinococcosis positive cases.**

| **OEVRs** | **Region** | **Province** |
| --- | --- | --- |
| OEVR - ABRUZZO MOLISE | ABRUZZO | Chieti, L'Aquila, Pescara, Teramo |
|  | MOLISE | Campobasso, Isernia |
| OEVR - MEZZOGIORNO | BASILICATA | Matera, Potenza |
|  | CALABRIA | Catanzaro, Cosenza, Crotone, Reggio di Calabria, Vibo Valentia |
|  | CAMPANIA | Avellino, Benevento, Caserta, Napoli, Salerno |
|  | PUGLIA | Bari, Brindisi, Foggia, Lecce, Taranto |
| OEVR – EMILIA ROMAGNA | EMILIA ROMAGNA | Bologna, Ferrara, Forlì-Cesena, Modena, Parma, Piacenza, Ravenna, Reggio nell'Emilia, Rimini |
| OEVR – LAZIO E TOSCANA | LAZIO | Frosinone, Latina, Rieti, Roma, Viterbo |
| OEVR – LOMBARDIA | LOMBARDIA | Bergamo, Brescia, Como, Cremona, Lecco, Lodi, Mantova, Milano, Monza e Brianza, Pavia, Sondrio, Varese |
| OEVR – UMBRIA E MARCHE | MARCHE | Ancona, Ascoli Piceno, Macerata, Pesaro e Urbino |
|  | UMBRIA | Perugia, Terni |
| OEVR – PIEMONTE | PIEMONTE | Alessandria, Asti, Biella, Cuneo, Novara, Torino, Verbano-Cusio-Ossola, Vercelli |
| OEVR – SARDEGNA | SARDEGNA | Cagliari, Nuoro, Oristano, Sassari |
| OEVR - VENETO | VENETO | Belluno, Padova, Rovigo, Treviso, Venezia, Verona, Vicenza |
