# Supplementary material for: Prevalence estimation of Italian ovine cystic echinococcosis in slaughterhouses: A retrospective Bayesian data analysis, 2010–2015
Source: PLoS One. 2019 Apr 1;14(4):e0214224. doi: 10.1371/journal.pone.0214224 (PMC6443144; doi:10.1371/journal.pone.0214224)
Supplement: S2 Table — (DOCX) [file pone.0214224.s003.docx]

**S2 Table. Ad hoc database fOR Sardinian farms resulted CE positive, including year of observation, positive farm code (encripped), data of slaughter, province and LoCAl Sanitary Agency code, species.**

| **year** | **positive_farm_code** | **data_slaught** | **Province** | **ASL** | **specie** |
| --- | --- | --- | --- | --- | --- |
| 2010 | IT032CAXXX | 05/01/2010 | CA | ASL 8 CAGLIARI | ovicaprini |
| 2010 | IT052CAXXX | 07/01/2010 | CA | ASL 8 CAGLIARI | ovicaprini |
| 2010 | IT057CAXXX | 08/01/2010 | CA | ASL 8 CAGLIARI | ovicaprini |
| 2010 | IT057CAXXX | 08/01/2010 | CA | ASL 8 CAGLIARI | ovicaprini |
| 2010 | IT072CAXXX | 08/01/2010 | CA | ASL 8 CAGLIARI | ovicaprini |
| 2010 | IT057CAXXX | 08/01/2010 | CA | ASL 8 CAGLIARI | ovicaprini |
| 2010 | IT057CAXXX | 08/01/2010 | CA | ASL 8 CAGLIARI | ovicaprini |
| 2010 | IT057CAXXX | 11/01/2010 | CA | ASL 8 CAGLIARI | ovicaprini |
| 2010 | IT032CAXXX | 11/01/2010 | CA | ASL 8 CAGLIARI | ovicaprini |
| 2010 | IT057CAXXX | 11/01/2010 | CA | ASL 8 CAGLIARI | ovicaprini |
| 2010 | IT101CAXXX | 11/01/2010 | CA | ASL 8 CAGLIARI | ovicaprini |
| 2010 | IT032CAXXX | 11/01/2010 | CA | ASL 8 CAGLIARI | ovicaprini |
| 2010 | IT014CAXXX | 12/01/2010 | CA | ASL 8 CAGLIARI | ovicaprini |
| 2010 | IT057ORXXX | 12/01/2010 | OR | ASL ORISTANO | ovicaprini |
| 2010 | IT072CAXXX | 12/01/2010 | CA | ASL 8 CAGLIARI | ovicaprini |
| 2010 | IT072CAXXX | 12/01/2010 | CA | ASL 8 CAGLIARI | ovicaprini |
| 2010 | IT014CAXXX | 12/01/2010 | CA | ASL 8 CAGLIARI | ovicaprini |
| 2010 | IT057ORXXX | 12/01/2010 | OR | ASL ORISTANO | ovicaprini |
| 2010 | IT052CAXXX | 14/01/2010 | CA | ASL 8 CAGLIARI | ovicaprini |
| 2010 | IT052CAXXX | 14/01/2010 | CA | ASL 8 CAGLIARI | ovicaprini |
| 2010 | IT057CAXXX | 14/01/2010 | CA | ASL 8 CAGLIARI | ovicaprini |
| 2010 | IT001CAXXX | 18/01/2010 | CA | ASL 8 CAGLIARI | ovicaprini |
| 2010 | IT052SSXXX | 18/01/2010 | SS | ASL 1 SASSARI | ovicaprini |
| 2010 | IT052SSXXX | 18/01/2010 | SS | ASL 1 SASSARI | ovicaprini |
| 2010 | IT092CAXXX | 18/01/2010 | CA | ASL 8 CAGLIARI | ovicaprini |
| 2010 | IT001CAXXX | 18/01/2010 | CA | ASL 8 CAGLIARI | ovicaprini |
| 2010 | IT020ORXXX | 19/01/2010 | OR | ASL ORISTANO | ovicaprini |
| 2010 | IT057ORXXX | 19/01/2010 | OR | ASL ORISTANO | ovicaprini |
| 2010 | IT057ORXXX | 19/01/2010 | OR | ASL ORISTANO | ovicaprini |
| 2010 | IT052SSXXX | 19/01/2010 | SS | ASL 1 SASSARI | ovicaprini |
| 2010 | IT020ORXXX | 19/01/2010 | OR | ASL ORISTANO | ovicaprini |
| 2010 | IT057ORXXX | 19/01/2010 | OR | ASL ORISTANO | ovicaprini |
| 2010 | IT057ORXXX | 19/01/2010 | OR | ASL ORISTANO | ovicaprini |
| 2010 | IT047ORXXX | 22/01/2010 | OR | ASL ORISTANO | ovicaprini |
| 2010 | IT029CAXXX | 22/01/2010 | CA | ASL 8 CAGLIARI | ovicaprini |
| 2010 | IT047ORXXX | 22/01/2010 | OR | ASL ORISTANO | ovicaprini |
| 2010 | IT001CAXXX | 22/01/2010 | CA | ASL 8 CAGLIARI | ovicaprini |
| 2010 | IT001CAXXX | 25/01/2010 | CA | ASL 8 CAGLIARI | ovicaprini |
| 2010 | IT046ORXXX | 25/01/2010 | OR | ASL ORISTANO | ovicaprini |
| 2010 | IT032CAXXX | 25/01/2010 | CA | ASL 8 CAGLIARI | ovicaprini |
| 2010 | IT101CAXXX | 25/01/2010 | CA | ASL 8 CAGLIARI | ovicaprini |
| 2010 | IT069ORXXX | 25/01/2010 | OR | ASL ORISTANO | ovicaprini |
| 2010 | IT001CAXXX | 25/01/2010 | CA | ASL 8 CAGLIARI | ovicaprini |
| 2010 | IT001CAXXX | 25/01/2010 | CA | ASL 8 CAGLIARI | ovicaprini |
| 2010 | IT046ORXXX | 25/01/2010 | OR | ASL ORISTANO | ovicaprini |
| 2010 | IT032CAXXX | 25/01/2010 | CA | ASL 8 CAGLIARI | ovicaprini |
| 2010 | IT101CAXXX | 25/01/2010 | CA | ASL 8 CAGLIARI | ovicaprini |
| 2010 | IT069ORXXX | 25/01/2010 | OR | ASL ORISTANO | ovicaprini |
| 2010 | IT092CAXXX | 25/01/2010 | CA | ASL 8 CAGLIARI | ovicaprini |
| 2010 | IT039ORXXX | 26/01/2010 | OR | ASL ORISTANO | ovicaprini |
| 2010 | IT032CAXXX | 26/01/2010 | CA | ASL 8 CAGLIARI | ovicaprini |
| 2010 | IT032CAXXX | 26/01/2010 | CA | ASL 8 CAGLIARI | ovicaprini |
| 2010 | IT039ORXXX | 26/01/2010 | OR | ASL ORISTANO | ovicaprini |
| 2010 | IT032CAXXX | 26/01/2010 | CA | ASL 8 CAGLIARI | ovicaprini |
| 2010 | IT032CAXXX | 26/01/2010 | CA | ASL 8 CAGLIARI | ovicaprini |
| 2010 | IT078CAXXX | 27/01/2010 | CA | ASL 8 CAGLIARI | ovicaprini |
| 2010 | IT078CAXXX | 27/01/2010 | CA | ASL 8 CAGLIARI | ovicaprini |
| 2010 | IT052CAXXX | 29/01/2010 | CA | ASL 8 CAGLIARI | ovicaprini |
| 2010 | IT031CAXXX | 01/02/2010 | CA | ASL 8 CAGLIARI | ovicaprini |
| 2010 | IT057CAXXX | 01/02/2010 | CA | ASL 8 CAGLIARI | ovicaprini |
| 2010 | IT032CAXXX | 01/02/2010 | CA | ASL 8 CAGLIARI | ovicaprini |
| 2010 | IT092CAXXX | 01/02/2010 | CA | ASL 8 CAGLIARI | ovicaprini |
| 2010 | IT029CAXXX | 01/02/2010 | CA | ASL 8 CAGLIARI | ovicaprini |
| 2010 | IT057CAXXX | 01/02/2010 | CA | ASL 8 CAGLIARI | ovicaprini |
| 2010 | IT032CAXXX | 01/02/2010 | CA | ASL 8 CAGLIARI | ovicaprini |
| 2010 | IT092CAXXX | 01/02/2010 | CA | ASL 8 CAGLIARI | ovicaprini |
| 2010 | IT092CAXXX | 01/02/2010 | CA | ASL 8 CAGLIARI | ovicaprini |
| 2010 | IT029CAXXX | 01/02/2010 | CA | ASL 8 CAGLIARI | ovicaprini |
| 2010 | IT032CAXXX | 02/02/2010 | CA | ASL 8 CAGLIARI | ovicaprini |
| 2010 | IT028ORXXX | 02/02/2010 | OR | ASL ORISTANO | ovicaprini |
| 2010 | IT032CAXXX | 02/02/2010 | CA | ASL 8 CAGLIARI | ovicaprini |
| 2010 | IT032CAXXX | 02/02/2010 | CA | ASL 8 CAGLIARI | ovicaprini |
| 2010 | IT092CAXXX | 02/02/2010 | CA | ASL 8 CAGLIARI | ovicaprini |
| 2010 | IT028ORXXX | 02/02/2010 | OR | ASL ORISTANO | ovicaprini |
| 2010 | IT057ORXXX | 03/02/2010 | OR | ASL ORISTANO | ovicaprini |
| 2010 | IT057ORXXX | 03/02/2010 | OR | ASL ORISTANO | ovicaprini |
| 2010 | IT101CAXXX | 04/02/2010 | CA | ASL 8 CAGLIARI | ovicaprini |
| 2010 | IT057CAXXX | 05/02/2010 | CA | ASL 8 CAGLIARI | ovicaprini |
| 2010 | IT092CAXXX | 05/02/2010 | CA | ASL 8 CAGLIARI | ovicaprini |
| 2010 | IT057CAXXX | 08/02/2010 | CA | ASL 8 CAGLIARI | ovicaprini |
| 2010 | IT029CAXXX | 08/02/2010 | CA | ASL 8 CAGLIARI | ovicaprini |
| 2010 | IT057CAXXX | 08/02/2010 | CA | ASL 8 CAGLIARI | ovicaprini |
| 2010 | IT101CAXXX | 08/02/2010 | CA | ASL 8 CAGLIARI | ovicaprini |
| 2010 | IT092CAXXX | 08/02/2010 | CA | ASL 8 CAGLIARI | ovicaprini |
| 2010 | IT029CAXXX | 08/02/2010 | CA | ASL 8 CAGLIARI | ovicaprini |
| 2010 | IT029CAXXX | 08/02/2010 | CA | ASL 8 CAGLIARI | ovicaprini |
| 2010 | IT092CAXXX | 08/02/2010 | CA | ASL 8 CAGLIARI | ovicaprini |
| 2010 | IT057CAXXX | 09/02/2010 | CA | ASL 8 CAGLIARI | ovicaprini |
| 2010 | IT057ORXXX | 09/02/2010 | OR | ASL ORISTANO | ovicaprini |
| 2010 | IT006CAXXX | 09/02/2010 | CA | ASL 8 CAGLIARI | ovicaprini |
| 2010 | IT057CAXXX | 09/02/2010 | CA | ASL 8 CAGLIARI | ovicaprini |
| 2010 | IT057ORXXX | 09/02/2010 | OR | ASL ORISTANO | ovicaprini |
| 2010 | IT006CAXXX | 09/02/2010 | CA | ASL 8 CAGLIARI | ovicaprini |
| 2010 | IT092CAXXX | 09/02/2010 | CA | ASL 8 CAGLIARI | ovicaprini |
| 2010 | IT092CAXXX | 11/02/2010 | CA | ASL 8 CAGLIARI | ovicaprini |
| 2010 | IT057CAXXX | 11/02/2010 | CA | ASL 8 CAGLIARI | ovicaprini |
| 2010 | IT092CAXXX | 11/02/2010 | CA | ASL 8 CAGLIARI | ovicaprini |
| 2010 | IT057CAXXX | 11/02/2010 | CA | ASL 8 CAGLIARI | ovicaprini |
| 2010 | IT080CAXXX | 12/02/2010 | CA | ASL 8 CAGLIARI | ovicaprini |
| 2010 | IT012CAXXX | 12/02/2010 | CA | ASL 8 CAGLIARI | ovicaprini |
| 2010 | IT032CAXXX | 12/02/2010 | CA | ASL 8 CAGLIARI | ovicaprini |
| 2010 | IT072CAXXX | 12/02/2010 | CA | ASL 8 CAGLIARI | ovicaprini |
| 2010 | IT012CAXXX | 12/02/2010 | CA | ASL 8 CAGLIARI | ovicaprini |
| 2010 | IT012CAXXX | 12/02/2010 | CA | ASL 8 CAGLIARI | ovicaprini |
| 2010 | IT031CAXXX | 15/02/2010 | CA | ASL 8 CAGLIARI | ovicaprini |
| 2010 | IT092CAXXX | 15/02/2010 | CA | ASL 8 CAGLIARI | ovicaprini |
| 2010 | IT029CAXXX | 15/02/2010 | CA | ASL 8 CAGLIARI | ovicaprini |
| 2010 | IT092CAXXX | 15/02/2010 | CA | ASL 8 CAGLIARI | ovicaprini |
| 2010 | IT046CAXXX | 15/02/2010 | CA | ASL 8 CAGLIARI | ovicaprini |
| 2010 | IT001CAXXX | 15/02/2010 | CA | ASL 8 CAGLIARI | ovicaprini |
| 2010 | IT092CAXXX | 15/02/2010 | CA | ASL 8 CAGLIARI | ovicaprini |
| 2010 | IT029CAXXX | 15/02/2010 | CA | ASL 8 CAGLIARI | ovicaprini |
| 2010 | IT029CAXXX | 15/02/2010 | CA | ASL 8 CAGLIARI | ovicaprini |
| 2010 | IT092CAXXX | 15/02/2010 | CA | ASL 8 CAGLIARI | ovicaprini |
| 2010 | IT101CAXXX | 16/02/2010 | CA | ASL 8 CAGLIARI | ovicaprini |
| 2010 | IT086CAXXX | 16/02/2010 | CA | ASL 8 CAGLIARI | ovicaprini |
| 2010 | IT032CAXXX | 16/02/2010 | CA | ASL 8 CAGLIARI | ovicaprini |
| 2010 | IT091CAXXX | 16/02/2010 | CA | ASL 8 CAGLIARI | ovicaprini |
| 2010 | IT101CAXXX | 16/02/2010 | CA | ASL 8 CAGLIARI | ovicaprini |
| 2010 | IT020CAXXX | 16/02/2010 | CA | ASL 8 CAGLIARI | ovicaprini |
| 2010 | IT086CAXXX | 16/02/2010 | CA | ASL 8 CAGLIARI | ovicaprini |
| 2010 | IT086CAXXX | 16/02/2010 | CA | ASL 8 CAGLIARI | ovicaprini |
| 2010 | IT032CAXXX | 16/02/2010 | CA | ASL 8 CAGLIARI | ovicaprini |
| 2010 | IT091CAXXX | 16/02/2010 | CA | ASL 8 CAGLIARI | ovicaprini |
| 2010 | IT065CAXXX | 17/02/2010 | CA | ASL 8 CAGLIARI | ovicaprini |
| 2010 | IT069ORXXX | 17/02/2010 | OR | ASL ORISTANO | ovicaprini |
| 2010 | IT065CAXXX | 17/02/2010 | CA | ASL 8 CAGLIARI | ovicaprini |
| 2010 | IT069ORXXX | 17/02/2010 | OR | ASL ORISTANO | ovicaprini |
| 2010 | IT057CAXXX | 18/02/2010 | CA | ASL 8 CAGLIARI | ovicaprini |
| 2010 | IT032CAXXX | 19/02/2010 | CA | ASL 8 CAGLIARI | ovicaprini |
| 2010 | IT039ORXXX | 19/02/2010 | OR | ASL ORISTANO | ovicaprini |
| 2010 | IT057CAXXX | 19/02/2010 | CA | ASL 8 CAGLIARI | ovicaprini |
| 2010 | IT032CAXXX | 19/02/2010 | CA | ASL 8 CAGLIARI | ovicaprini |
| 2010 | IT032CAXXX | 19/02/2010 | CA | ASL 8 CAGLIARI | ovicaprini |
| 2010 | IT039ORXXX | 19/02/2010 | OR | ASL ORISTANO | ovicaprini |
| 2010 | IT092CAXXX | 19/02/2010 | CA | ASL 8 CAGLIARI | ovicaprini |
| 2010 | IT032CAXXX | 19/02/2010 | CA | ASL 8 CAGLIARI | ovicaprini |
| 2010 | IT045CAXXX | 19/02/2010 | CA | ASL 8 CAGLIARI | ovicaprini |
| 2010 | IT080NUXXX | 22/02/2010 | NU | ASL NUORO | ovicaprini |
| 2010 | IT055CAXXX | 22/02/2010 | CA | ASL 8 CAGLIARI | ovicaprini |
| 2010 | IT029CAXXX | 22/02/2010 | CA | ASL 8 CAGLIARI | ovicaprini |
| 2010 | IT032CAXXX | 22/02/2010 | CA | ASL 8 CAGLIARI | ovicaprini |
| 2010 | IT055CAXXX | 22/02/2010 | CA | ASL 8 CAGLIARI | ovicaprini |
| 2010 | IT029CAXXX | 22/02/2010 | CA | ASL 8 CAGLIARI | ovicaprini |
| 2010 | IT092CAXXX | 22/02/2010 | CA | ASL 8 CAGLIARI | ovicaprini |
| 2010 | IT057CAXXX | 23/02/2010 | CA | ASL 8 CAGLIARI | ovicaprini |
| 2010 | IT092CAXXX | 23/02/2010 | CA | ASL 8 CAGLIARI | ovicaprini |
| 2010 | IT057CAXXX | 23/02/2010 | CA | ASL 8 CAGLIARI | ovicaprini |
| 2010 | IT092CAXXX | 23/02/2010 | CA | ASL 8 CAGLIARI | ovicaprini |
| 2010 | IT092CAXXX | 23/02/2010 | CA | ASL 8 CAGLIARI | ovicaprini |
| 2010 | IT067CAXXX | 25/02/2010 | CA | ASL 8 CAGLIARI | ovicaprini |
| 2010 | IT067CAXXX | 25/02/2010 | CA | ASL 8 CAGLIARI | ovicaprini |
| 2010 | IT067CAXXX | 26/02/2010 | CA | ASL 8 CAGLIARI | ovicaprini |
| 2010 | IT039ORXXX | 26/02/2010 | OR | ASL ORISTANO | ovicaprini |
| 2010 | IT039ORXXX | 26/02/2010 | OR | ASL ORISTANO | ovicaprini |
| 2010 | IT057ORXXX | 26/02/2010 | OR | ASL ORISTANO | ovicaprini |
| 2010 | IT067CAXXX | 26/02/2010 | CA | ASL 8 CAGLIARI | ovicaprini |
| 2010 | IT039ORXXX | 26/02/2010 | OR | ASL ORISTANO | ovicaprini |
| 2010 | IT039ORXXX | 26/02/2010 | OR | ASL ORISTANO | ovicaprini |
| 2010 | IT057ORXXX | 26/02/2010 | OR | ASL ORISTANO | ovicaprini |
| 2010 | IT065CAXXX | 01/03/2010 | CA | ASL 8 CAGLIARI | ovicaprini |
| 2010 | IT101CAXXX | 01/03/2010 | CA | ASL 8 CAGLIARI | ovicaprini |
| 2010 | IT101CAXXX | 01/03/2010 | CA | ASL 8 CAGLIARI | ovicaprini |
| 2010 | IT092CAXXX | 01/03/2010 | CA | ASL 8 CAGLIARI | ovicaprini |
| 2010 | IT032CAXXX | 01/03/2010 | CA | ASL 8 CAGLIARI | ovicaprini |
| 2010 | IT012ORXXX | 02/03/2010 | OR | ASL ORISTANO | ovicaprini |
| 2010 | IT032CAXXX | 02/03/2010 | CA | ASL 8 CAGLIARI | ovicaprini |
| 2010 | IT069ORXXX | 02/03/2010 | OR | ASL ORISTANO | ovicaprini |
| 2010 | IT057ORXXX | 02/03/2010 | OR | ASL ORISTANO | ovicaprini |
| 2010 | IT016CAXXX | 02/03/2010 | CA | ASL 8 CAGLIARI | ovicaprini |
| 2010 | IT032CAXXX | 02/03/2010 | CA | ASL 8 CAGLIARI | ovicaprini |
| 2010 | IT012ORXXX | 02/03/2010 | OR | ASL ORISTANO | ovicaprini |
| 2010 | IT032CAXXX | 02/03/2010 | CA | ASL 8 CAGLIARI | ovicaprini |
| 2010 | IT069ORXXX | 02/03/2010 | OR | ASL ORISTANO | ovicaprini |
| 2010 | IT057ORXXX | 02/03/2010 | OR | ASL ORISTANO | ovicaprini |
| 2010 | IT053CAXXX | 04/03/2010 | CA | ASL 8 CAGLIARI | ovicaprini |
| 2010 | IT045CAXXX | 05/03/2010 | CA | ASL 8 CAGLIARI | ovicaprini |
| 2010 | IT045CAXXX | 05/03/2010 | CA | ASL 8 CAGLIARI | ovicaprini |
| 2010 | IT045CAXXX | 05/03/2010 | CA | ASL 8 CAGLIARI | ovicaprini |
| 2010 | IT001CAXXX | 05/03/2010 | CA | ASL 8 CAGLIARI | ovicaprini |
| 2010 | IT045CAXXX | 05/03/2010 | CA | ASL 8 CAGLIARI | ovicaprini |
| 2010 | IT045CAXXX | 05/03/2010 | CA | ASL 8 CAGLIARI | ovicaprini |
| 2010 | IT045CAXXX | 05/03/2010 | CA | ASL 8 CAGLIARI | ovicaprini |
| 2010 | IT001CAXXX | 05/03/2010 | CA | ASL 8 CAGLIARI | ovicaprini |
| 2010 | IT045CAXXX | 05/03/2010 | CA | ASL 8 CAGLIARI | ovicaprini |
| 2010 | IT045CAXXX | 05/03/2010 | CA | ASL 8 CAGLIARI | ovicaprini |
| 2010 | IT065CAXXX | 08/03/2010 | CA | ASL 8 CAGLIARI | ovicaprini |
| 2010 | IT101CAXXX | 08/03/2010 | CA | ASL 8 CAGLIARI | ovicaprini |
| 2010 | IT101CAXXX | 08/03/2010 | CA | ASL 8 CAGLIARI | ovicaprini |
| 2010 | IT101CAXXX | 08/03/2010 | CA | ASL 8 CAGLIARI | ovicaprini |
| 2010 | IT101CAXXX | 08/03/2010 | CA | ASL 8 CAGLIARI | ovicaprini |
| 2010 | IT101CAXXX | 08/03/2010 | CA | ASL 8 CAGLIARI | ovicaprini |
| 2010 | IT101CAXXX | 08/03/2010 | CA | ASL 8 CAGLIARI | ovicaprini |
| 2010 | IT093CAXXX | 09/03/2010 | CA | ASL 8 CAGLIARI | ovicaprini |
| 2010 | IT039ORXXX | 09/03/2010 | OR | ASL ORISTANO | ovicaprini |
| 2010 | IT032CAXXX | 09/03/2010 | CA | ASL 8 CAGLIARI | ovicaprini |
| 2010 | IT093CAXXX | 09/03/2010 | CA | ASL 8 CAGLIARI | ovicaprini |
| 2010 | IT039ORXXX | 09/03/2010 | OR | ASL ORISTANO | ovicaprini |
| 2010 | IT093CAXXX | 11/03/2010 | CA | ASL 8 CAGLIARI | ovicaprini |
| 2010 | IT093CAXXX | 11/03/2010 | CA | ASL 8 CAGLIARI | ovicaprini |
| 2010 | IT047ORXXX | 12/03/2010 | OR | ASL ORISTANO | ovicaprini |
| 2010 | IT032CAXXX | 12/03/2010 | CA | ASL 8 CAGLIARI | ovicaprini |
| 2010 | IT032CAXXX | 12/03/2010 | CA | ASL 8 CAGLIARI | ovicaprini |
| 2010 | IT057CAXXX | 12/03/2010 | CA | ASL 8 CAGLIARI | ovicaprini |
| 2010 | IT047ORXXX | 12/03/2010 | OR | ASL ORISTANO | ovicaprini |
| 2010 | IT032CAXXX | 12/03/2010 | CA | ASL 8 CAGLIARI | ovicaprini |
| 2010 | IT032CAXXX | 12/03/2010 | CA | ASL 8 CAGLIARI | ovicaprini |
| 2010 | IT032CAXXX | 12/03/2010 | CA | ASL 8 CAGLIARI | ovicaprini |
| 2010 | IT072CAXXX | 12/03/2010 | CA | ASL 8 CAGLIARI | ovicaprini |
| 2010 | IT093CAXXX | 15/03/2010 | CA | ASL 8 CAGLIARI | ovicaprini |
| 2010 | IT093CAXXX | 15/03/2010 | CA | ASL 8 CAGLIARI | ovicaprini |
| 2010 | IT057CAXXX | 15/03/2010 | CA | ASL 8 CAGLIARI | ovicaprini |
| 2010 | IT032CAXXX | 15/03/2010 | CA | ASL 8 CAGLIARI | ovicaprini |
| 2010 | IT092CAXXX | 15/03/2010 | CA | ASL 8 CAGLIARI | ovicaprini |
| 2010 | IT032CAXXX | 15/03/2010 | CA | ASL 8 CAGLIARI | ovicaprini |
| 2010 | IT032CAXXX | 15/03/2010 | CA | ASL 8 CAGLIARI | ovicaprini |
| 2010 | IT093CAXXX | 15/03/2010 | CA | ASL 8 CAGLIARI | ovicaprini |
| 2010 | IT093CAXXX | 15/03/2010 | CA | ASL 8 CAGLIARI | ovicaprini |
| 2010 | IT057CAXXX | 15/03/2010 | CA | ASL 8 CAGLIARI | ovicaprini |
| 2010 | IT032CAXXX | 15/03/2010 | CA | ASL 8 CAGLIARI | ovicaprini |
| 2010 | IT092CAXXX | 15/03/2010 | CA | ASL 8 CAGLIARI | ovicaprini |
| 2010 | IT092CAXXX | 15/03/2010 | CA | ASL 8 CAGLIARI | ovicaprini |
| 2010 | IT092CAXXX | 15/03/2010 | CA | ASL 8 CAGLIARI | ovicaprini |
| 2010 | IT032CAXXX | 15/03/2010 | CA | ASL 8 CAGLIARI | ovicaprini |
| 2010 | IT032CAXXX | 15/03/2010 | CA | ASL 8 CAGLIARI | ovicaprini |
| 2010 | IT039ORXXX | 16/03/2010 | OR | ASL ORISTANO | ovicaprini |
| 2010 | IT039ORXXX | 16/03/2010 | OR | ASL ORISTANO | ovicaprini |
| 2010 | IT093CAXXX | 18/03/2010 | CA | ASL 8 CAGLIARI | ovicaprini |
| 2010 | IT093CAXXX | 18/03/2010 | CA | ASL 8 CAGLIARI | ovicaprini |
| 2010 | IT093CAXXX | 19/03/2010 | CA | ASL 8 CAGLIARI | ovicaprini |
| 2010 | IT032CAXXX | 19/03/2010 | CA | ASL 8 CAGLIARI | ovicaprini |
| 2010 | IT065CAXXX | 19/03/2010 | CA | ASL 8 CAGLIARI | ovicaprini |
| 2010 | IT092CAXXX | 19/03/2010 | CA | ASL 8 CAGLIARI | ovicaprini |
| 2010 | IT093CAXXX | 19/03/2010 | CA | ASL 8 CAGLIARI | ovicaprini |
| 2010 | IT032CAXXX | 19/03/2010 | CA | ASL 8 CAGLIARI | ovicaprini |
| 2010 | IT032CAXXX | 19/03/2010 | CA | ASL 8 CAGLIARI | ovicaprini |
| 2010 | IT065CAXXX | 19/03/2010 | CA | ASL 8 CAGLIARI | ovicaprini |
| 2010 | IT092CAXXX | 19/03/2010 | CA | ASL 8 CAGLIARI | ovicaprini |
| 2010 | IT057CAXXX | 22/03/2010 | CA | ASL 8 CAGLIARI | ovicaprini |
| 2010 | IT092CAXXX | 22/03/2010 | CA | ASL 8 CAGLIARI | ovicaprini |
| 2010 | IT078CAXXX | 22/03/2010 | CA | ASL 8 CAGLIARI | ovicaprini |
| 2010 | IT032CAXXX | 22/03/2010 | CA | ASL 8 CAGLIARI | ovicaprini |
| 2010 | IT032CAXXX | 22/03/2010 | CA | ASL 8 CAGLIARI | ovicaprini |
| 2010 | IT032CAXXX | 22/03/2010 | CA | ASL 8 CAGLIARI | ovicaprini |
| 2010 | IT092CAXXX | 22/03/2010 | CA | ASL 8 CAGLIARI | ovicaprini |
| 2010 | IT078CAXXX | 22/03/2010 | CA | ASL 8 CAGLIARI | ovicaprini |
| 2010 | IT032CAXXX | 22/03/2010 | CA | ASL 8 CAGLIARI | ovicaprini |
| 2010 | IT078CAXXX | 23/03/2010 | CA | ASL 8 CAGLIARI | ovicaprini |
| 2010 | IT046CAXXX | 23/03/2010 | CA | ASL 8 CAGLIARI | ovicaprini |
| 2010 | IT091CAXXX | 23/03/2010 | CA | ASL 8 CAGLIARI | ovicaprini |
| 2010 | IT032CAXXX | 23/03/2010 | CA | ASL 8 CAGLIARI | ovicaprini |
| 2010 | IT078CAXXX | 23/03/2010 | CA | ASL 8 CAGLIARI | ovicaprini |
| 2010 | IT046CAXXX | 23/03/2010 | CA | ASL 8 CAGLIARI | ovicaprini |
| 2010 | IT091CAXXX | 23/03/2010 | CA | ASL 8 CAGLIARI | ovicaprini |
| 2010 | IT057ORXXX | 25/03/2010 | OR | ASL ORISTANO | ovicaprini |
| 2010 | IT053CAXXX | 25/03/2010 | CA | ASL 8 CAGLIARI | ovicaprini |
| 2010 | IT057ORXXX | 25/03/2010 | OR | ASL ORISTANO | ovicaprini |
| 2010 | IT029ORXXX | 26/03/2010 | OR | ASL ORISTANO | ovicaprini |
| 2010 | IT029ORXXX | 26/03/2010 | OR | ASL ORISTANO | ovicaprini |
| 2010 | IT001CAXXX | 26/03/2010 | CA | ASL 8 CAGLIARI | ovicaprini |
| 2010 | IT029ORXXX | 26/03/2010 | OR | ASL ORISTANO | ovicaprini |
| 2010 | IT029ORXXX | 26/03/2010 | OR | ASL ORISTANO | ovicaprini |
| 2010 | IT001CAXXX | 26/03/2010 | CA | ASL 8 CAGLIARI | ovicaprini |
| 2010 | IT078CAXXX | 28/03/2010 | CA | ASL 8 CAGLIARI | ovicaprini |
| 2010 | IT078CAXXX | 28/03/2010 | CA | ASL 8 CAGLIARI | ovicaprini |
| 2010 | IT085CAXXX | 29/03/2010 | CA | ASL 8 CAGLIARI | ovicaprini |
| 2010 | IT101CAXXX | 29/03/2010 | CA | ASL 8 CAGLIARI | ovicaprini |
| 2010 | IT085CAXXX | 29/03/2010 | CA | ASL 8 CAGLIARI | ovicaprini |
| 2010 | IT092CAXXX | 29/03/2010 | CA | ASL 8 CAGLIARI | ovicaprini |
| 2010 | IT101CAXXX | 29/03/2010 | CA | ASL 8 CAGLIARI | ovicaprini |
| 2010 | IT092CAXXX | 29/03/2010 | CA | ASL 8 CAGLIARI | ovicaprini |
| 2010 | IT049ORXXX | 30/03/2010 | OR | ASL ORISTANO | ovicaprini |
| 2010 | IT057ORXXX | 30/03/2010 | OR | ASL ORISTANO | ovicaprini |
| 2010 | IT049ORXXX | 30/03/2010 | OR | ASL ORISTANO | ovicaprini |
| 2010 | IT033ORXXX | 30/03/2010 | OR | ASL ORISTANO | ovicaprini |
| 2010 | IT057CAXXX | 30/03/2010 | CA | ASL 8 CAGLIARI | ovicaprini |
| 2010 | IT057ORXXX | 30/03/2010 | OR | ASL ORISTANO | ovicaprini |
| 2010 | IT029CAXXX | 06/04/2010 | CA | ASL 8 CAGLIARI | ovicaprini |
| 2010 | IT091CAXXX | 07/04/2010 | CA | ASL 8 CAGLIARI | ovicaprini |
| 2010 | IT072CAXXX | 08/04/2010 | CA | ASL 8 CAGLIARI | ovicaprini |
| 2010 | IT065CAXXX | 12/04/2010 | CA | ASL 8 CAGLIARI | ovicaprini |
| 2010 | IT057CAXXX | 12/04/2010 | CA | ASL 8 CAGLIARI | ovicaprini |
| 2010 | IT032CAXXX | 12/04/2010 | CA | ASL 8 CAGLIARI | ovicaprini |
| 2010 | IT078CAXXX | 12/04/2010 | CA | ASL 8 CAGLIARI | ovicaprini |
| 2010 | IT046ORXXX | 12/04/2010 | OR | ASL ORISTANO | ovicaprini |
| 2010 | IT096CAXXX | 12/04/2010 | CA | ASL 8 CAGLIARI | ovicaprini |
| 2010 | IT020CAXXX | 13/04/2010 | CA | ASL 8 CAGLIARI | ovicaprini |
| 2010 | IT057CAXXX | 13/04/2010 | CA | ASL 8 CAGLIARI | ovicaprini |
| 2010 | IT078CAXXX | 13/04/2010 | CA | ASL 8 CAGLIARI | ovicaprini |
| 2010 | IT028ORXXX | 13/04/2010 | OR | ASL ORISTANO | ovicaprini |
| 2010 | IT028ORXXX | 13/04/2010 | OR | ASL ORISTANO | ovicaprini |
| 2010 | IT094CAXXX | 14/04/2010 | CA | ASL 8 CAGLIARI | ovicaprini |
| 2010 | IT094CAXXX | 14/04/2010 | CA | ASL 8 CAGLIARI | ovicaprini |
| 2010 | IT094CAXXX | 14/04/2010 | CA | ASL 8 CAGLIARI | ovicaprini |
| 2010 | IT001CAXXX | 14/04/2010 | CA | ASL 8 CAGLIARI | ovicaprini |
| 2010 | IT020CAXXX | 15/04/2010 | CA | ASL 8 CAGLIARI | ovicaprini |
| 2010 | IT057CAXXX | 16/04/2010 | CA | ASL 8 CAGLIARI | ovicaprini |
| 2010 | IT028ORXXX | 16/04/2010 | OR | ASL ORISTANO | ovicaprini |
| 2010 | IT065CAXXX | 16/04/2010 | CA | ASL 8 CAGLIARI | ovicaprini |
| 2010 | IT034NUXXX | 16/04/2010 | NU | ASL NUORO | ovicaprini |
| 2010 | IT033CAXXX | 16/04/2010 | CA | ASL 8 CAGLIARI | ovicaprini |
| 2010 | IT033CAXXX | 16/04/2010 | CA | ASL 8 CAGLIARI | ovicaprini |
| 2010 | IT055CAXXX | 16/04/2010 | CA | ASL 8 CAGLIARI | ovicaprini |
| 2010 | IT069ORXXX | 16/04/2010 | OR | ASL ORISTANO | ovicaprini |
| 2010 | IT055CAXXX | 16/04/2010 | CA | ASL 8 CAGLIARI | ovicaprini |
| 2010 | IT069ORXXX | 16/04/2010 | OR | ASL ORISTANO | ovicaprini |
| 2010 | IT047ORXXX | 16/04/2010 | OR | ASL ORISTANO | ovicaprini |
| 2010 | IT070ORXXX | 16/04/2010 | OR | ASL ORISTANO | ovicaprini |
| 2010 | IT024NUXXX | 16/04/2010 | NU | ASL NUORO | ovicaprini |
| 2010 | IT066ORXXX | 16/04/2010 | OR | ASL ORISTANO | ovicaprini |
| 2010 | IT033CAXXX | 16/04/2010 | CA | ASL 8 CAGLIARI | ovicaprini |
| 2010 | IT057CAXXX | 19/04/2010 | CA | ASL 8 CAGLIARI | ovicaprini |
| 2010 | IT032CAXXX | 19/04/2010 | CA | ASL 8 CAGLIARI | ovicaprini |
| 2010 | IT032CAXXX | 19/04/2010 | CA | ASL 8 CAGLIARI | ovicaprini |
| 2010 | IT078CAXXX | 19/04/2010 | CA | ASL 8 CAGLIARI | ovicaprini |
| 2010 | IT029ORXXX | 19/04/2010 | OR | ASL ORISTANO | ovicaprini |
| 2010 | IT029ORXXX | 19/04/2010 | OR | ASL ORISTANO | ovicaprini |
| 2010 | IT055CAXXX | 19/04/2010 | CA | ASL 8 CAGLIARI | ovicaprini |
| 2010 | IT091CAXXX | 19/04/2010 | CA | ASL 8 CAGLIARI | ovicaprini |
| 2010 | IT031CAXXX | 19/04/2010 | CA | ASL 8 CAGLIARI | ovicaprini |
| 2010 | IT029CAXXX | 19/04/2010 | CA | ASL 8 CAGLIARI | ovicaprini |
| 2010 | IT047ORXXX | 19/04/2010 | OR | ASL ORISTANO | ovicaprini |
| 2010 | IT035CAXXX | 20/04/2010 | CA | ASL 8 CAGLIARI | ovicaprini |
| 2010 | IT032CAXXX | 20/04/2010 | CA | ASL 8 CAGLIARI | ovicaprini |
| 2010 | IT062ORXXX | 20/04/2010 | OR | ASL ORISTANO | ovicaprini |
| 2010 | IT062ORXXX | 20/04/2010 | OR | ASL ORISTANO | ovicaprini |
| 2010 | IT042ORXXX | 20/04/2010 | OR | ASL ORISTANO | ovicaprini |
| 2010 | IT069ORXXX | 23/04/2010 | OR | ASL ORISTANO | ovicaprini |
| 2010 | IT092CAXXX | 23/04/2010 | CA | ASL 8 CAGLIARI | ovicaprini |
| 2010 | IT057ORXXX | 23/04/2010 | OR | ASL ORISTANO | ovicaprini |
| 2010 | IT073ORXXX | 23/04/2010 | OR | ASL ORISTANO | ovicaprini |
| 2010 | IT004ORXXX | 23/04/2010 | OR | ASL ORISTANO | ovicaprini |
| 2010 | IT069ORXXX | 23/04/2010 | OR | ASL ORISTANO | ovicaprini |
| 2010 | IT069ORXXX | 23/04/2010 | OR | ASL ORISTANO | ovicaprini |
| 2010 | IT040CAXXX | 23/04/2010 | CA | ASL 8 CAGLIARI | ovicaprini |
| 2010 | IT012CAXXX | 23/04/2010 | CA | ASL 8 CAGLIARI | ovicaprini |
| 2010 | IT065CAXXX | 26/04/2010 | CA | ASL 8 CAGLIARI | ovicaprini |
| 2010 | IT057CAXXX | 26/04/2010 | CA | ASL 8 CAGLIARI | ovicaprini |
| 2010 | IT025CAXXX | 26/04/2010 | CA | ASL 8 CAGLIARI | ovicaprini |
| 2010 | IT030NUXXX | 26/04/2010 | NU | ASL NUORO | ovicaprini |
| 2010 | IT078CAXXX | 26/04/2010 | CA | ASL 8 CAGLIARI | ovicaprini |
| 2010 | IT032CAXXX | 26/04/2010 | CA | ASL 8 CAGLIARI | ovicaprini |
| 2010 | IT092CAXXX | 26/04/2010 | CA | ASL 8 CAGLIARI | ovicaprini |
| 2010 | IT012ORXXX | 26/04/2010 | OR | ASL ORISTANO | ovicaprini |
| 2010 | IT096CAXXX | 26/04/2010 | CA | ASL 8 CAGLIARI | ovicaprini |
| 2010 | IT078CAXXX | 27/04/2010 | CA | ASL 8 CAGLIARI | ovicaprini |
| 2010 | IT032CAXXX | 27/04/2010 | CA | ASL 8 CAGLIARI | ovicaprini |
| 2010 | IT092CAXXX | 28/04/2010 | CA | ASL 8 CAGLIARI | ovicaprini |
| 2010 | IT072CAXXX | 29/04/2010 | CA | ASL 8 CAGLIARI | ovicaprini |
| 2010 | IT057CAXXX | 30/04/2010 | CA | ASL 8 CAGLIARI | ovicaprini |
| 2010 | IT069ORXXX | 30/04/2010 | OR | ASL ORISTANO | ovicaprini |
| 2010 | IT033CAXXX | 30/04/2010 | CA | ASL 8 CAGLIARI | ovicaprini |
| 2010 | IT069ORXXX | 30/04/2010 | OR | ASL ORISTANO | ovicaprini |
| 2010 | IT046CAXXX | 03/05/2010 | CA | ASL 8 CAGLIARI | ovicaprini |
| 2010 | IT072CAXXX | 03/05/2010 | CA | ASL 8 CAGLIARI | ovicaprini |
| 2010 | 033CA10XXX | 03/05/2010 | CA | ASL 8 CAGLIARI | ovicaprini |
| 2010 | 033CA10XXX | 03/05/2010 | CA | ASL 8 CAGLIARI | ovicaprini |
| 2010 | 096CA00XXX | 03/05/2010 | CA | ASL 8 CAGLIARI | ovicaprini |
| 2010 | 012OR02XXX | 03/05/2010 | OR | ASL ORISTANO | ovicaprini |
| 2010 | 029CA07XXX | 03/05/2010 | CA | ASL 8 CAGLIARI | ovicaprini |
| 2010 | 055CA01XXX | 03/05/2010 | CA | ASL 8 CAGLIARI | ovicaprini |
| 2010 | 012CA22XXX | 03/05/2010 | CA | ASL 8 CAGLIARI | ovicaprini |
| 2010 | IT053CAXXX | 04/05/2010 | CA | ASL 8 CAGLIARI | ovicaprini |
| 2010 | IT092CAXXX | 04/05/2010 | CA | ASL 8 CAGLIARI | ovicaprini |
| 2010 | IT057CAXXX | 04/05/2010 | CA | ASL 8 CAGLIARI | ovicaprini |
| 2010 | 039OR12XXX | 04/05/2010 | OR | ASL ORISTANO | ovicaprini |
| 2010 | 016CA03XXX | 04/05/2010 | CA | ASL 8 CAGLIARI | ovicaprini |
| 2010 | IT046CAXXX | 05/05/2010 | CA | ASL 8 CAGLIARI | ovicaprini |
| 2010 | IT020CAXXX | 06/05/2010 | CA | ASL 8 CAGLIARI | ovicaprini |
| 2010 | IT072CAXXX | 06/05/2010 | CA | ASL 8 CAGLIARI | ovicaprini |
| 2010 | 036NU05XXX | 07/05/2010 | NU | ASL NUORO | ovicaprini |
| 2010 | 065CA02XXX | 07/05/2010 | CA | ASL 8 CAGLIARI | ovicaprini |
| 2010 | 065CA02XXX | 07/05/2010 | CA | ASL 8 CAGLIARI | ovicaprini |
| 2010 | IT065CAXXX | 10/05/2010 | CA | ASL 8 CAGLIARI | ovicaprini |
| 2010 | IT046CAXXX | 10/05/2010 | CA | ASL 8 CAGLIARI | ovicaprini |
| 2010 | IT072CAXXX | 10/05/2010 | CA | ASL 8 CAGLIARI | ovicaprini |
| 2010 | IT020CAXXX | 10/05/2010 | CA | ASL 8 CAGLIARI | ovicaprini |
| 2010 | IT025CAXXX | 10/05/2010 | CA | ASL 8 CAGLIARI | ovicaprini |
| 2010 | IT055CAXXX | 10/05/2010 | CA | ASL 8 CAGLIARI | ovicaprini |
| 2010 | 014CA01XXX | 10/05/2010 | CA | ASL 8 CAGLIARI | ovicaprini |
| 2010 | 024OR01XXX | 10/05/2010 | OR | ASL ORISTANO | ovicaprini |
| 2010 | 086CA01XXX | 10/05/2010 | CA | ASL 8 CAGLIARI | ovicaprini |
| 2010 | 096CA00XXX | 10/05/2010 | CA | ASL 8 CAGLIARI | ovicaprini |
| 2010 | 092CA06XXX | 10/05/2010 | CA | ASL 8 CAGLIARI | ovicaprini |
| 2010 | 029CA07XXX | 10/05/2010 | CA | ASL 8 CAGLIARI | ovicaprini |
| 2010 | 004OR01XXX | 11/05/2010 | OR | ASL ORISTANO | ovicaprini |
| 2010 | 004OR01XXX | 11/05/2010 | OR | ASL ORISTANO | ovicaprini |
| 2010 | IT046CAXXX | 12/05/2010 | CA | ASL 8 CAGLIARI | ovicaprini |
| 2010 | IT057CAXXX | 12/05/2010 | CA | ASL 8 CAGLIARI | ovicaprini |
| 2010 | 024OR00XXX | 12/05/2010 | OR | ASL ORISTANO | ovicaprini |
| 2010 | IT001CAXXX | 13/05/2010 | CA | ASL 8 CAGLIARI | ovicaprini |
| 2010 | IT019CAXXX | 13/05/2010 | CA | ASL 8 CAGLIARI | ovicaprini |
| 2010 | IT093CAXXX | 13/05/2010 | CA | ASL 8 CAGLIARI | ovicaprini |
| 2010 | IT032CAXXX | 14/05/2010 | CA | ASL 8 CAGLIARI | ovicaprini |
| 2010 | 016CA04XXX | 14/05/2010 | CA | ASL 8 CAGLIARI | ovicaprini |
| 2010 | 024NU05XXX | 14/05/2010 | NU | ASL NUORO | ovicaprini |
| 2010 | IT023CAXXX | 17/05/2010 | CA | ASL 8 CAGLIARI | ovicaprini |
| 2010 | IT057CAXXX | 17/05/2010 | CA | ASL 8 CAGLIARI | ovicaprini |
| 2010 | IT019CAXXX | 17/05/2010 | CA | ASL 8 CAGLIARI | ovicaprini |
| 2010 | 092CA12XXX | 17/05/2010 | CA | ASL 8 CAGLIARI | ovicaprini |
| 2010 | 055CA01XXX | 17/05/2010 | CA | ASL 8 CAGLIARI | ovicaprini |
| 2010 | 055CA00XXX | 17/05/2010 | CA | ASL 8 CAGLIARI | ovicaprini |
| 2010 | 029OR06XXX | 17/05/2010 | OR | ASL ORISTANO | ovicaprini |
| 2010 | 096CA00XXX | 17/05/2010 | CA | ASL 8 CAGLIARI | ovicaprini |
| 2010 | 065CA01XXX | 17/05/2010 | CA | ASL 8 CAGLIARI | ovicaprini |
| 2010 | 091CA00XXX | 17/05/2010 | CA | ASL 8 CAGLIARI | ovicaprini |
| 2010 | 029CA07XXX | 17/05/2010 | CA | ASL 8 CAGLIARI | ovicaprini |
| 2010 | 029CA07XXX | 17/05/2010 | CA | ASL 8 CAGLIARI | ovicaprini |
| 2010 | 037OR02XXX | 18/05/2010 | OR | ASL ORISTANO | ovicaprini |
| 2010 | 034CA02XXX | 18/05/2010 | CA | ASL 8 CAGLIARI | ovicaprini |
| 2010 | 085CA02XXX | 18/05/2010 | CA | ASL 8 CAGLIARI | ovicaprini |
| 2010 | IT023CAXXX | 19/05/2010 | CA | ASL 8 CAGLIARI | ovicaprini |
| 2010 | IT093CAXXX | 20/05/2010 | CA | ASL 8 CAGLIARI | ovicaprini |
| 2010 | IT053CAXXX | 20/05/2010 | CA | ASL 8 CAGLIARI | ovicaprini |
| 2010 | IT092CAXXX | 20/05/2010 | CA | ASL 8 CAGLIARI | ovicaprini |
| 2010 | IT001CAXXX | 21/05/2010 | CA | ASL 8 CAGLIARI | ovicaprini |
| 2010 | IT006ORXXX | 21/05/2010 | OR | ASL ORISTANO | ovicaprini |
| 2010 | IT092CAXXX | 21/05/2010 | CA | ASL 8 CAGLIARI | ovicaprini |
| 2010 | 033CA19XXX | 21/05/2010 | CA | ASL 8 CAGLIARI | ovicaprini |
| 2010 | 012CA00XXX | 21/05/2010 | CA | ASL 8 CAGLIARI | ovicaprini |
| 2010 | IT092CAXXX | 24/05/2010 | CA | ASL 8 CAGLIARI | ovicaprini |
| 2010 | IT093CAXXX | 24/05/2010 | CA | ASL 8 CAGLIARI | ovicaprini |
| 2010 | 092CA06XXX | 24/05/2010 | CA | ASL 8 CAGLIARI | ovicaprini |
| 2010 | 029CA07XXX | 24/05/2010 | CA | ASL 8 CAGLIARI | ovicaprini |
| 2010 | 029CA07XXX | 24/05/2010 | CA | ASL 8 CAGLIARI | ovicaprini |
| 2010 | 044OR06XXX | 24/05/2010 | OR | ASL ORISTANO | ovicaprini |
| 2010 | 057CA04XXX | 24/05/2010 | CA | ASL 8 CAGLIARI | ovicaprini |
| 2010 | 016CA00XXX | 24/05/2010 | CA | ASL 8 CAGLIARI | ovicaprini |
| 2010 | 096CA02XXX | 24/05/2010 | CA | ASL 8 CAGLIARI | ovicaprini |
| 2010 | 046OR07XXX | 24/05/2010 | OR | ASL ORISTANO | ovicaprini |
| 2010 | IT030NUXXX | 25/05/2010 | NU | ASL NUORO | ovicaprini |
| 2010 | IT030NUXXX | 25/05/2010 | NU | ASL NUORO | ovicaprini |
| 2010 | 039OR03XXX | 25/05/2010 | OR | ASL ORISTANO | ovicaprini |
| 2010 | IT046CAXXX | 26/05/2010 | CA | ASL 8 CAGLIARI | ovicaprini |
| 2010 | IT030NUXXX | 27/05/2010 | NU | ASL NUORO | ovicaprini |
| 2010 | IT046CAXXX | 28/05/2010 | CA | ASL 8 CAGLIARI | ovicaprini |
| 2010 | IT092CAXXX | 28/05/2010 | CA | ASL 8 CAGLIARI | ovicaprini |
| 2010 | 014CA01XXX | 28/05/2010 | CA | ASL 8 CAGLIARI | ovicaprini |
| 2010 | 012CA06XXX | 28/05/2010 | CA | ASL 8 CAGLIARI | ovicaprini |
| 2010 | 012OR02XXX | 28/05/2010 | OR | ASL ORISTANO | ovicaprini |
| 2010 | IT031CAXXX | 31/05/2010 | CA | ASL 8 CAGLIARI | ovicaprini |
| 2010 | IT046CAXXX | 31/05/2010 | CA | ASL 8 CAGLIARI | ovicaprini |
| 2010 | IT093CAXXX | 31/05/2010 | CA | ASL 8 CAGLIARI | ovicaprini |
| 2010 | IT022ORXXX | 31/05/2010 | OR | ASL ORISTANO | ovicaprini |
| 2010 | IT092CAXXX | 31/05/2010 | CA | ASL 8 CAGLIARI | ovicaprini |
| 2010 | 053CA01XXX | 31/05/2010 | CA | ASL 8 CAGLIARI | ovicaprini |
| 2010 | 032CA04XXX | 31/05/2010 | CA | ASL 8 CAGLIARI | ovicaprini |
| 2010 | 070OR00XXX | 31/05/2010 | OR | ASL ORISTANO | ovicaprini |
| 2010 | 012OR00XXX | 31/05/2010 | OR | ASL ORISTANO | ovicaprini |
| 2010 | 045CA03XXX | 31/05/2010 | CA | ASL 8 CAGLIARI | ovicaprini |
| 2010 | 091CA03XXX | 31/05/2010 | CA | ASL 8 CAGLIARI | ovicaprini |
| 2010 | IT061CAXXX | 01/06/2010 | CA | ASL 8 CAGLIARI | ovicaprini |
| 2010 | 055CA01XXX | 01/06/2010 | CA | ASL 8 CAGLIARI | ovicaprini |
| 2010 | IT031CAXXX | 07/06/2010 | CA | ASL 8 CAGLIARI | ovicaprini |
| 2010 | IT057CAXXX | 07/06/2010 | CA | ASL 8 CAGLIARI | ovicaprini |
| 2010 | IT052CAXXX | 07/06/2010 | CA | ASL 8 CAGLIARI | ovicaprini |
| 2010 | IT092CAXXX | 07/06/2010 | CA | ASL 8 CAGLIARI | ovicaprini |
| 2010 | IT092CAXXX | 07/06/2010 | CA | ASL 8 CAGLIARI | ovicaprini |
| 2010 | 045CA03XXX | 07/06/2010 | CA | ASL 8 CAGLIARI | ovicaprini |
| 2010 | 048OR06XXX | 07/06/2010 | OR | ASL ORISTANO | ovicaprini |
| 2010 | 091CA03XXX | 07/06/2010 | CA | ASL 8 CAGLIARI | ovicaprini |
| 2010 | 053CA00XXX | 07/06/2010 | CA | ASL 8 CAGLIARI | ovicaprini |
| 2010 | 032CA04XXX | 07/06/2010 | CA | ASL 8 CAGLIARI | ovicaprini |
| 2010 | 046OR07XXX | 07/06/2010 | OR | ASL ORISTANO | ovicaprini |
| 2010 | 055CA01XXX | 07/06/2010 | CA | ASL 8 CAGLIARI | ovicaprini |
| 2010 | 028CA04XXX | 07/06/2010 | CA | ASL 8 CAGLIARI | ovicaprini |
| 2010 | IT092CAXXX | 08/06/2010 | CA | ASL 8 CAGLIARI | ovicaprini |
| 2010 | IT032CAXXX | 08/06/2010 | CA | ASL 8 CAGLIARI | ovicaprini |
| 2010 | 091CA10XXX | 08/06/2010 | CA | ASL 8 CAGLIARI | ovicaprini |
| 2010 | IT057CAXXX | 09/06/2010 | CA | ASL 8 CAGLIARI | ovicaprini |
| 2010 | IT021CAXXX | 10/06/2010 | CA | ASL 8 CAGLIARI | ovicaprini |
| 2010 | IT046CAXXX | 11/06/2010 | CA | ASL 8 CAGLIARI | ovicaprini |
| 2010 | IT080NUXXX | 11/06/2010 | NU | ASL NUORO | ovicaprini |
| 2010 | IT057CAXXX | 11/06/2010 | CA | ASL 8 CAGLIARI | ovicaprini |
| 2010 | IT069CAXXX | 14/06/2010 | CA | ASL 8 CAGLIARI | ovicaprini |
| 2010 | IT008ORXXX | 14/06/2010 | OR | ASL ORISTANO | ovicaprini |
| 2010 | IT091CAXXX | 14/06/2010 | CA | ASL 8 CAGLIARI | ovicaprini |
| 2010 | IT092CAXXX | 14/06/2010 | CA | ASL 8 CAGLIARI | ovicaprini |
| 2010 | IT032CAXXX | 14/06/2010 | CA | ASL 8 CAGLIARI | ovicaprini |
| 2010 | 019CA00XXX | 14/06/2010 | CA | ASL 8 CAGLIARI | ovicaprini |
| 2010 | 019CA00XXX | 14/06/2010 | CA | ASL 8 CAGLIARI | ovicaprini |
| 2010 | 091CA12XXX | 14/06/2010 | CA | ASL 8 CAGLIARI | ovicaprini |
| 2010 | 045CA07XXX | 14/06/2010 | CA | ASL 8 CAGLIARI | ovicaprini |
| 2010 | 045CA07XXX | 14/06/2010 | CA | ASL 8 CAGLIARI | ovicaprini |
| 2010 | 045CA07XXX | 14/06/2010 | CA | ASL 8 CAGLIARI | ovicaprini |
| 2010 | 045CA03XXX | 14/06/2010 | CA | ASL 8 CAGLIARI | ovicaprini |
| 2010 | 058OR00XXX | 14/06/2010 | OR | ASL ORISTANO | ovicaprini |
| 2010 | 035CA00XXX | 14/06/2010 | CA | ASL 8 CAGLIARI | ovicaprini |
| 2010 | 069CA00XXX | 14/06/2010 | CA | ASL 8 CAGLIARI | ovicaprini |
| 2010 | IT057CAXXX | 15/06/2010 | CA | ASL 8 CAGLIARI | ovicaprini |
| 2010 | 039OR03XXX | 15/06/2010 | OR | ASL ORISTANO | ovicaprini |
| 2010 | 039OR12XXX | 15/06/2010 | OR | ASL ORISTANO | ovicaprini |
| 2010 | IT092CAXXX | 17/06/2010 | CA | ASL 8 CAGLIARI | ovicaprini |
| 2010 | IT057CAXXX | 17/06/2010 | CA | ASL 8 CAGLIARI | ovicaprini |
| 2010 | IT020CAXXX | 18/06/2010 | CA | ASL 8 CAGLIARI | ovicaprini |
| 2010 | IT033CAXXX | 18/06/2010 | CA | ASL 8 CAGLIARI | ovicaprini |
| 2010 | 019CA00XXX | 18/06/2010 | CA | ASL 8 CAGLIARI | ovicaprini |
| 2010 | 069OR00XXX | 18/06/2010 | OR | ASL ORISTANO | ovicaprini |
| 2010 | 039OR03XXX | 18/06/2010 | OR | ASL ORISTANO | ovicaprini |
| 2010 | 032CA04XXX | 18/06/2010 | CA | ASL 8 CAGLIARI | ovicaprini |
| 2010 | 032CA04XXX | 18/06/2010 | CA | ASL 8 CAGLIARI | ovicaprini |
| 2010 | 072CA03XXX | 18/06/2010 | CA | ASL 8 CAGLIARI | ovicaprini |
| 2010 | 029CA03XXX | 18/06/2010 | CA | ASL 8 CAGLIARI | ovicaprini |
| 2010 | 029CA03XXX | 18/06/2010 | CA | ASL 8 CAGLIARI | ovicaprini |
| 2010 | 040CA02XXX | 18/06/2010 | CA | ASL 8 CAGLIARI | ovicaprini |
| 2010 | 012CA11XXX | 18/06/2010 | CA | ASL 8 CAGLIARI | ovicaprini |
| 2010 | 069OR00XXX | 18/06/2010 | OR | ASL ORISTANO | ovicaprini |
| 2010 | 092CA00XXX | 18/06/2010 | CA | ASL 8 CAGLIARI | ovicaprini |
| 2010 | IT008ORXXX | 21/06/2010 | OR | ASL ORISTANO | ovicaprini |
| 2010 | IT057CAXXX | 21/06/2010 | CA | ASL 8 CAGLIARI | ovicaprini |
| 2010 | IT032CAXXX | 21/06/2010 | CA | ASL 8 CAGLIARI | ovicaprini |
| 2010 | IT020CAXXX | 21/06/2010 | CA | ASL 8 CAGLIARI | ovicaprini |
| 2010 | 092CA00XXX | 21/06/2010 | CA | ASL 8 CAGLIARI | ovicaprini |
| 2010 | 091CA02XXX | 21/06/2010 | CA | ASL 8 CAGLIARI | ovicaprini |
| 2010 | 061OR01XXX | 21/06/2010 | OR | ASL ORISTANO | ovicaprini |
| 2010 | 045CA00XXX | 21/06/2010 | CA | ASL 8 CAGLIARI | ovicaprini |
| 2010 | 025CA04XXX | 21/06/2010 | CA | ASL 8 CAGLIARI | ovicaprini |
| 2010 | 089CA00XXX | 21/06/2010 | CA | ASL 8 CAGLIARI | ovicaprini |
| 2010 | 069CA00XXX | 21/06/2010 | CA | ASL 8 CAGLIARI | ovicaprini |
| 2010 | 031CA00XXX | 21/06/2010 | CA | ASL 8 CAGLIARI | ovicaprini |
| 2010 | 055CA01XXX | 21/06/2010 | CA | ASL 8 CAGLIARI | ovicaprini |
| 2010 | IT057CAXXX | 22/06/2010 | CA | ASL 8 CAGLIARI | ovicaprini |
| 2010 | 092CA01XXX | 22/06/2010 | CA | ASL 8 CAGLIARI | ovicaprini |
| 2010 | IT086CAXXX | 23/06/2010 | CA | ASL 8 CAGLIARI | ovicaprini |
| 2010 | IT019CAXXX | 24/06/2010 | CA | ASL 8 CAGLIARI | ovicaprini |
| 2010 | IT053CAXXX | 24/06/2010 | CA | ASL 8 CAGLIARI | ovicaprini |
| 2010 | IT006CAXXX | 24/06/2010 | CA | ASL 8 CAGLIARI | ovicaprini |
| 2010 | IT006CAXXX | 24/06/2010 | CA | ASL 8 CAGLIARI | ovicaprini |
| 2010 | IT006CAXXX | 24/06/2010 | CA | ASL 8 CAGLIARI | ovicaprini |
| 2010 | IT086CAXXX | 25/06/2010 | CA | ASL 8 CAGLIARI | ovicaprini |
| 2010 | IT092CAXXX | 25/06/2010 | CA | ASL 8 CAGLIARI | ovicaprini |
| 2010 | IT065CAXXX | 25/06/2010 | CA | ASL 8 CAGLIARI | ovicaprini |
| 2010 | IT081ORXXX | 25/06/2010 | OR | ASL ORISTANO | ovicaprini |
| 2010 | 070OR03XXX | 25/06/2010 | OR | ASL ORISTANO | ovicaprini |
| 2010 | 028OR05XXX | 25/06/2010 | OR | ASL ORISTANO | ovicaprini |
| 2010 | 092CA00XXX | 25/06/2010 | CA | ASL 8 CAGLIARI | ovicaprini |
| 2010 | 028CA00XXX | 25/06/2010 | CA | ASL 8 CAGLIARI | ovicaprini |
| 2010 | 102CA00XXX | 25/06/2010 | CA | ASL 8 CAGLIARI | ovicaprini |
| 2010 | 026CA04XXX | 25/06/2010 | CA | ASL 8 CAGLIARI | ovicaprini |
| 2010 | IT057CAXXX | 28/06/2010 | CA | ASL 8 CAGLIARI | ovicaprini |
| 2010 | IT093CAXXX | 28/06/2010 | CA | ASL 8 CAGLIARI | ovicaprini |
| 2010 | 081OR00XXX | 28/06/2010 | OR | ASL ORISTANO | ovicaprini |
| 2010 | 092CA12XXX | 28/06/2010 | CA | ASL 8 CAGLIARI | ovicaprini |
| 2010 | 096CA03XXX | 28/06/2010 | CA | ASL 8 CAGLIARI | ovicaprini |
| 2010 | 091CA01XXX | 28/06/2010 | CA | ASL 8 CAGLIARI | ovicaprini |
| 2010 | 057CA04XXX | 28/06/2010 | CA | ASL 8 CAGLIARI | ovicaprini |
| 2010 | 008OR03XXX | 28/06/2010 | OR | ASL ORISTANO | ovicaprini |
| 2010 | IT057CAXXX | 29/06/2010 | CA | ASL 8 CAGLIARI | ovicaprini |
| 2010 | 004OR01XXX | 29/06/2010 | OR | ASL ORISTANO | ovicaprini |
| 2010 | 008OR03XXX | 29/06/2010 | OR | ASL ORISTANO | ovicaprini |
| 2010 | IT081ORXXX | 30/06/2010 | OR | ASL ORISTANO | ovicaprini |
| 2010 | IT067CAXXX | 01/07/2010 | CA | ASL 8 CAGLIARI | ovicaprini |
| 2010 | IT067CAXXX | 01/07/2010 | CA | ASL 8 CAGLIARI | ovicaprini |
| 2010 | IT069ORXXX | 02/07/2010 | OR | ASL ORISTANO | ovicaprini |
| 2010 | IT080NUXXX | 05/07/2010 | NU | ASL NUORO | ovicaprini |
| 2010 | IT081ORXXX | 05/07/2010 | OR | ASL ORISTANO | ovicaprini |
| 2010 | IT025CAXXX | 05/07/2010 | CA | ASL 8 CAGLIARI | ovicaprini |
| 2010 | IT091CAXXX | 05/07/2010 | CA | ASL 8 CAGLIARI | ovicaprini |
| 2010 | IT032CAXXX | 05/07/2010 | CA | ASL 8 CAGLIARI | ovicaprini |
| 2010 | IT032CAXXX | 05/07/2010 | CA | ASL 8 CAGLIARI | ovicaprini |
| 2010 | IT032CAXXX | 05/07/2010 | CA | ASL 8 CAGLIARI | ovicaprini |
| 2010 | IT001CAXXX | 05/07/2010 | CA | ASL 8 CAGLIARI | ovicaprini |
| 2010 | IT025CAXXX | 05/07/2010 | CA | ASL 8 CAGLIARI | ovicaprini |
| 2010 | IT091CAXXX | 05/07/2010 | CA | ASL 8 CAGLIARI | ovicaprini |
| 2010 | IT032CAXXX | 05/07/2010 | CA | ASL 8 CAGLIARI | ovicaprini |
| 2010 | IT032CAXXX | 05/07/2010 | CA | ASL 8 CAGLIARI | ovicaprini |
| 2010 | IT032CAXXX | 05/07/2010 | CA | ASL 8 CAGLIARI | ovicaprini |
| 2010 | IT001CAXXX | 05/07/2010 | CA | ASL 8 CAGLIARI | ovicaprini |
| 2010 | IT057CAXXX | 05/07/2010 | CA | ASL 8 CAGLIARI | ovicaprini |
| 2010 | IT092CAXXX | 05/07/2010 | CA | ASL 8 CAGLIARI | ovicaprini |
| 2010 | IT096CAXXX | 05/07/2010 | CA | ASL 8 CAGLIARI | ovicaprini |
| 2010 | IT091CAXXX | 05/07/2010 | CA | ASL 8 CAGLIARI | ovicaprini |
| 2010 | IT077ORXXX | 05/07/2010 | OR | ASL ORISTANO | ovicaprini |
| 2010 | IT069ORXXX | 05/07/2010 | OR | ASL ORISTANO | ovicaprini |
| 2010 | IT028ORXXX | 05/07/2010 | OR | ASL ORISTANO | ovicaprini |
| 2010 | IT057ORXXX | 06/07/2010 | OR | ASL ORISTANO | ovicaprini |
| 2010 | IT092CAXXX | 06/07/2010 | CA | ASL 8 CAGLIARI | ovicaprini |
| 2010 | IT057ORXXX | 06/07/2010 | OR | ASL ORISTANO | ovicaprini |
| 2010 | IT092CAXXX | 06/07/2010 | CA | ASL 8 CAGLIARI | ovicaprini |
| 2010 | IT096CAXXX | 06/07/2010 | CA | ASL 8 CAGLIARI | ovicaprini |
| 2010 | IT081ORXXX | 07/07/2010 | OR | ASL ORISTANO | ovicaprini |
| 2010 | IT072CAXXX | 08/07/2010 | CA | ASL 8 CAGLIARI | ovicaprini |
| 2010 | IT072CAXXX | 08/07/2010 | CA | ASL 8 CAGLIARI | ovicaprini |
| 2010 | IT057CAXXX | 08/07/2010 | CA | ASL 8 CAGLIARI | ovicaprini |
| 2010 | IT072CAXXX | 08/07/2010 | CA | ASL 8 CAGLIARI | ovicaprini |
| 2010 | IT072CAXXX | 08/07/2010 | CA | ASL 8 CAGLIARI | ovicaprini |
| 2010 | IT057CAXXX | 08/07/2010 | CA | ASL 8 CAGLIARI | ovicaprini |
| 2010 | IT092CAXXX | 09/07/2010 | CA | ASL 8 CAGLIARI | ovicaprini |
| 2010 | IT092CAXXX | 09/07/2010 | CA | ASL 8 CAGLIARI | ovicaprini |
| 2010 | IT036NUXXX | 09/07/2010 | NU | ASL NUORO | ovicaprini |
| 2010 | IT092CAXXX | 12/07/2010 | CA | ASL 8 CAGLIARI | ovicaprini |
| 2010 | IT092CAXXX | 12/07/2010 | CA | ASL 8 CAGLIARI | ovicaprini |
| 2010 | IT096CAXXX | 12/07/2010 | CA | ASL 8 CAGLIARI | ovicaprini |
| 2010 | IT069ORXXX | 12/07/2010 | OR | ASL ORISTANO | ovicaprini |
| 2010 | IT073ORXXX | 12/07/2010 | OR | ASL ORISTANO | ovicaprini |
| 2010 | IT028ORXXX | 12/07/2010 | OR | ASL ORISTANO | ovicaprini |
| 2010 | IT014CAXXX | 12/07/2010 | CA | ASL 8 CAGLIARI | ovicaprini |
| 2010 | IT028ORXXX | 12/07/2010 | OR | ASL ORISTANO | ovicaprini |
| 2010 | IT035CAXXX | 12/07/2010 | CA | ASL 8 CAGLIARI | ovicaprini |
| 2010 | IT091CAXXX | 12/07/2010 | CA | ASL 8 CAGLIARI | ovicaprini |
| 2010 | IT032CAXXX | 12/07/2010 | CA | ASL 8 CAGLIARI | ovicaprini |
| 2010 | IT032CAXXX | 12/07/2010 | CA | ASL 8 CAGLIARI | ovicaprini |
| 2010 | IT029ORXXX | 13/07/2010 | OR | ASL ORISTANO | ovicaprini |
| 2010 | IT057ORXXX | 15/07/2010 | OR | ASL ORISTANO | ovicaprini |
| 2010 | IT057ORXXX | 15/07/2010 | OR | ASL ORISTANO | ovicaprini |
| 2010 | IT069ORXXX | 16/07/2010 | OR | ASL ORISTANO | ovicaprini |
| 2010 | IT072CAXXX | 16/07/2010 | CA | ASL 8 CAGLIARI | ovicaprini |
| 2010 | IT024ORXXX | 20/07/2010 | OR | ASL ORISTANO | ovicaprini |
| 2010 | IT037ORXXX | 20/07/2010 | OR | ASL ORISTANO | ovicaprini |
| 2010 | IT096CAXXX | 20/07/2010 | CA | ASL 8 CAGLIARI | ovicaprini |
| 2010 | IT096CAXXX | 20/07/2010 | CA | ASL 8 CAGLIARI | ovicaprini |
| 2010 | IT046CAXXX | 20/07/2010 | CA | ASL 8 CAGLIARI | ovicaprini |
| 2010 | IT035CAXXX | 20/07/2010 | CA | ASL 8 CAGLIARI | ovicaprini |
| 2010 | IT069ORXXX | 20/07/2010 | OR | ASL ORISTANO | ovicaprini |
| 2010 | IT024ORXXX | 20/07/2010 | OR | ASL ORISTANO | ovicaprini |
| 2010 | IT091CAXXX | 20/07/2010 | CA | ASL 8 CAGLIARI | ovicaprini |
| 2010 | IT091CAXXX | 20/07/2010 | CA | ASL 8 CAGLIARI | ovicaprini |
| 2010 | IT057CAXXX | 22/07/2010 | CA | ASL 8 CAGLIARI | ovicaprini |
| 2010 | IT057CAXXX | 22/07/2010 | CA | ASL 8 CAGLIARI | ovicaprini |
| 2010 | IT065CAXXX | 26/07/2010 | CA | ASL 8 CAGLIARI | ovicaprini |
| 2010 | IT096CAXXX | 26/07/2010 | CA | ASL 8 CAGLIARI | ovicaprini |
| 2010 | IT026ORXXX | 26/07/2010 | OR | ASL ORISTANO | ovicaprini |
| 2010 | IT091CAXXX | 26/07/2010 | CA | ASL 8 CAGLIARI | ovicaprini |
| 2010 | IT035CAXXX | 26/07/2010 | CA | ASL 8 CAGLIARI | ovicaprini |
| 2010 | IT057ORXXX | 26/07/2010 | OR | ASL ORISTANO | ovicaprini |
| 2010 | IT042ORXXX | 26/07/2010 | OR | ASL ORISTANO | ovicaprini |
| 2010 | IT024ORXXX | 26/07/2010 | OR | ASL ORISTANO | ovicaprini |
| 2010 | IT019CAXXX | 27/07/2010 | CA | ASL 8 CAGLIARI | ovicaprini |
| 2010 | IT066CAXXX | 27/07/2010 | CA | ASL 8 CAGLIARI | ovicaprini |
| 2010 | IT019CAXXX | 27/07/2010 | CA | ASL 8 CAGLIARI | ovicaprini |
| 2010 | IT066CAXXX | 27/07/2010 | CA | ASL 8 CAGLIARI | ovicaprini |
| 2010 | IT087CAXXX | 27/07/2010 | CA | ASL 8 CAGLIARI | ovicaprini |
| 2010 | IT028ORXXX | 28/07/2010 | OR | ASL ORISTANO | ovicaprini |
| 2010 | IT091CAXXX | 30/07/2010 | CA | ASL 8 CAGLIARI | ovicaprini |
| 2010 | IT091CAXXX | 30/07/2010 | CA | ASL 8 CAGLIARI | ovicaprini |
| 2010 | IT065CAXXX | 02/08/2010 | CA | ASL 8 CAGLIARI | ovicaprini |
| 2010 | IT036CAXXX | 02/08/2010 | CA | ASL 8 CAGLIARI | ovicaprini |
| 2010 | IT032CAXXX | 02/08/2010 | CA | ASL 8 CAGLIARI | ovicaprini |
| 2010 | IT032CAXXX | 02/08/2010 | CA | ASL 8 CAGLIARI | ovicaprini |
| 2010 | IT092CAXXX | 02/08/2010 | CA | ASL 8 CAGLIARI | ovicaprini |
| 2010 | IT096CAXXX | 02/08/2010 | CA | ASL 8 CAGLIARI | ovicaprini |
| 2010 | IT012ORXXX | 02/08/2010 | OR | ASL ORISTANO | ovicaprini |
| 2010 | IT029ORXXX | 02/08/2010 | OR | ASL ORISTANO | ovicaprini |
| 2010 | IT035CAXXX | 02/08/2010 | CA | ASL 8 CAGLIARI | ovicaprini |
| 2010 | IT052CAXXX | 03/08/2010 | CA | ASL 8 CAGLIARI | ovicaprini |
| 2010 | IT057CAXXX | 03/08/2010 | CA | ASL 8 CAGLIARI | ovicaprini |
| 2010 | IT057CAXXX | 03/08/2010 | CA | ASL 8 CAGLIARI | ovicaprini |
| 2010 | IT057CAXXX | 03/08/2010 | CA | ASL 8 CAGLIARI | ovicaprini |
| 2010 | IT091CAXXX | 03/08/2010 | CA | ASL 8 CAGLIARI | ovicaprini |
| 2010 | IT052CAXXX | 03/08/2010 | CA | ASL 8 CAGLIARI | ovicaprini |
| 2010 | IT057CAXXX | 03/08/2010 | CA | ASL 8 CAGLIARI | ovicaprini |
| 2010 | IT057CAXXX | 03/08/2010 | CA | ASL 8 CAGLIARI | ovicaprini |
| 2010 | IT057CAXXX | 03/08/2010 | CA | ASL 8 CAGLIARI | ovicaprini |
| 2010 | IT091CAXXX | 03/08/2010 | CA | ASL 8 CAGLIARI | ovicaprini |
| 2010 | IT032CAXXX | 03/08/2010 | CA | ASL 8 CAGLIARI | ovicaprini |
| 2010 | IT070ORXXX | 03/08/2010 | OR | ASL ORISTANO | ovicaprini |
| 2010 | IT028ORXXX | 03/08/2010 | OR | ASL ORISTANO | ovicaprini |
| 2010 | IT028ORXXX | 03/08/2010 | OR | ASL ORISTANO | ovicaprini |
| 2010 | IT091CAXXX | 05/08/2010 | CA | ASL 8 CAGLIARI | ovicaprini |
| 2010 | IT091CAXXX | 05/08/2010 | CA | ASL 8 CAGLIARI | ovicaprini |
| 2010 | IT035CAXXX | 09/08/2010 | CA | ASL 8 CAGLIARI | ovicaprini |
| 2010 | IT096CAXXX | 09/08/2010 | CA | ASL 8 CAGLIARI | ovicaprini |
| 2010 | IT029ORXXX | 09/08/2010 | OR | ASL ORISTANO | ovicaprini |
| 2010 | IT080NUXXX | 10/08/2010 | NU | ASL NUORO | ovicaprini |
| 2010 | IT006CAXXX | 10/08/2010 | CA | ASL 8 CAGLIARI | ovicaprini |
| 2010 | IT006CAXXX | 10/08/2010 | CA | ASL 8 CAGLIARI | ovicaprini |
| 2010 | IT055CAXXX | 10/08/2010 | CA | ASL 8 CAGLIARI | ovicaprini |
| 2010 | IT091CAXXX | 10/08/2010 | CA | ASL 8 CAGLIARI | ovicaprini |
| 2010 | IT057CAXXX | 16/08/2010 | CA | ASL 8 CAGLIARI | ovicaprini |
| 2010 | IT035CAXXX | 16/08/2010 | CA | ASL 8 CAGLIARI | ovicaprini |
| 2010 | IT055CAXXX | 18/08/2010 | CA | ASL 8 CAGLIARI | ovicaprini |
| 2010 | IT032CAXXX | 19/08/2010 | CA | ASL 8 CAGLIARI | ovicaprini |
| 2010 | IT069CAXXX | 19/08/2010 | CA | ASL 8 CAGLIARI | ovicaprini |
| 2010 | IT032CAXXX | 19/08/2010 | CA | ASL 8 CAGLIARI | ovicaprini |
| 2010 | IT069CAXXX | 19/08/2010 | CA | ASL 8 CAGLIARI | ovicaprini |
| 2010 | IT036CAXXX | 20/08/2010 | CA | ASL 8 CAGLIARI | ovicaprini |
| 2010 | IT036CAXXX | 20/08/2010 | CA | ASL 8 CAGLIARI | ovicaprini |
| 2010 | IT065CAXXX | 23/08/2010 | CA | ASL 8 CAGLIARI | ovicaprini |
| 2010 | IT036CAXXX | 23/08/2010 | CA | ASL 8 CAGLIARI | ovicaprini |
| 2010 | IT036CAXXX | 23/08/2010 | CA | ASL 8 CAGLIARI | ovicaprini |
| 2010 | IT001CAXXX | 23/08/2010 | CA | ASL 8 CAGLIARI | ovicaprini |
| 2010 | IT029ORXXX | 23/08/2010 | OR | ASL ORISTANO | ovicaprini |
| 2010 | IT035CAXXX | 23/08/2010 | CA | ASL 8 CAGLIARI | ovicaprini |
| 2010 | IT046CAXXX | 24/08/2010 | CA | ASL 8 CAGLIARI | ovicaprini |
| 2010 | IT046CAXXX | 24/08/2010 | CA | ASL 8 CAGLIARI | ovicaprini |
| 2010 | IT046CAXXX | 24/08/2010 | CA | ASL 8 CAGLIARI | ovicaprini |
| 2010 | IT046CAXXX | 24/08/2010 | CA | ASL 8 CAGLIARI | ovicaprini |
| 2010 | IT046CAXXX | 24/08/2010 | CA | ASL 8 CAGLIARI | ovicaprini |
| 2010 | IT046CAXXX | 24/08/2010 | CA | ASL 8 CAGLIARI | ovicaprini |
| 2010 | IT046CAXXX | 24/08/2010 | CA | ASL 8 CAGLIARI | ovicaprini |
| 2010 | IT046CAXXX | 24/08/2010 | CA | ASL 8 CAGLIARI | ovicaprini |
| 2010 | IT036CAXXX | 25/08/2010 | CA | ASL 8 CAGLIARI | ovicaprini |
| 2010 | IT057CAXXX | 30/08/2010 | CA | ASL 8 CAGLIARI | ovicaprini |
| 2010 | IT036CAXXX | 30/08/2010 | CA | ASL 8 CAGLIARI | ovicaprini |
| 2010 | IT092CAXXX | 30/08/2010 | CA | ASL 8 CAGLIARI | ovicaprini |
| 2010 | IT092CAXXX | 30/08/2010 | CA | ASL 8 CAGLIARI | ovicaprini |
| 2010 | IT035CAXXX | 30/08/2010 | CA | ASL 8 CAGLIARI | ovicaprini |
| 2010 | IT055CAXXX | 01/09/2010 | CA | ASL 8 CAGLIARI | ovicaprini |
| 2010 | IT072CAXXX | 03/09/2010 | CA | ASL 8 CAGLIARI | ovicaprini |
| 2010 | IT072CAXXX | 03/09/2010 | CA | ASL 8 CAGLIARI | ovicaprini |
| 2010 | IT012ORXXX | 03/09/2010 | OR | ASL ORISTANO | ovicaprini |
| 2010 | IT057CAXXX | 06/09/2010 | CA | ASL 8 CAGLIARI | ovicaprini |
| 2010 | IT066CAXXX | 07/09/2010 | CA | ASL 8 CAGLIARI | ovicaprini |
| 2010 | IT091CAXXX | 07/09/2010 | CA | ASL 8 CAGLIARI | ovicaprini |
| 2010 | IT066CAXXX | 07/09/2010 | CA | ASL 8 CAGLIARI | ovicaprini |
| 2010 | IT091CAXXX | 07/09/2010 | CA | ASL 8 CAGLIARI | ovicaprini |
| 2010 | IT086CAXXX | 08/09/2010 | CA | ASL 8 CAGLIARI | ovicaprini |
| 2010 | IT016CAXXX | 09/09/2010 | CA | ASL 8 CAGLIARI | ovicaprini |
| 2010 | IT016CAXXX | 09/09/2010 | CA | ASL 8 CAGLIARI | ovicaprini |
| 2010 | IT091CAXXX | 09/09/2010 | CA | ASL 8 CAGLIARI | ovicaprini |
| 2010 | IT016CAXXX | 09/09/2010 | CA | ASL 8 CAGLIARI | ovicaprini |
| 2010 | IT016CAXXX | 09/09/2010 | CA | ASL 8 CAGLIARI | ovicaprini |
| 2010 | IT091CAXXX | 09/09/2010 | CA | ASL 8 CAGLIARI | ovicaprini |
| 2010 | IT026ORXXX | 13/09/2010 | OR | ASL ORISTANO | ovicaprini |
| 2010 | IT030ORXXX | 13/09/2010 | OR | ASL ORISTANO | ovicaprini |
| 2010 | IT101CAXXX | 13/09/2010 | CA | ASL 8 CAGLIARI | ovicaprini |
| 2010 | IT057CAXXX | 15/09/2010 | CA | ASL 8 CAGLIARI | ovicaprini |
| 2010 | IT012ORXXX | 20/09/2010 | OR | ASL ORISTANO | ovicaprini |
| 2010 | IT004ORXXX | 21/09/2010 | OR | ASL ORISTANO | ovicaprini |
| 2010 | IT004ORXXX | 21/09/2010 | OR | ASL ORISTANO | ovicaprini |
| 2010 | IT057CAXXX | 22/09/2010 | CA | ASL 8 CAGLIARI | ovicaprini |
| 2010 | IT032CAXXX | 27/09/2010 | CA | ASL 8 CAGLIARI | ovicaprini |
| 2010 | IT032CAXXX | 27/09/2010 | CA | ASL 8 CAGLIARI | ovicaprini |
| 2010 | IT091CAXXX | 27/09/2010 | CA | ASL 8 CAGLIARI | ovicaprini |
| 2010 | IT034NUXXX | 29/09/2010 | NU | ASL NUORO | ovicaprini |
| 2010 | IT057CAXXX | 04/10/2010 | CA | ASL 8 CAGLIARI | ovicaprini |
| 2010 | IT091CAXXX | 04/10/2010 | CA | ASL 8 CAGLIARI | ovicaprini |
| 2010 | IT089CAXXX | 04/10/2010 | CA | ASL 8 CAGLIARI | ovicaprini |
| 2010 | IT006CAXXX | 04/10/2010 | CA | ASL 8 CAGLIARI | ovicaprini |
| 2010 | IT072CAXXX | 05/10/2010 | CA | ASL 8 CAGLIARI | ovicaprini |
| 2010 | IT072CAXXX | 08/10/2010 | CA | ASL 8 CAGLIARI | ovicaprini |
| 2010 | IT028ORXXX | 11/10/2010 | OR | ASL ORISTANO | ovicaprini |
| 2010 | IT091CAXXX | 11/10/2010 | CA | ASL 8 CAGLIARI | ovicaprini |
| 2010 | IT034NUXXX | 13/10/2010 | NU | ASL NUORO | ovicaprini |
| 2010 | IT072CAXXX | 15/10/2010 | CA | ASL 8 CAGLIARI | ovicaprini |
| 2010 | IT010NUXXX | 22/10/2010 | NU | ASL NUORO | ovicaprini |
| 2010 | IT010NUXXX | 25/10/2010 | NU | ASL NUORO | ovicaprini |
| 2010 | IT010NUXXX | 26/10/2010 | NU | ASL NUORO | ovicaprini |
| 2010 | IT057ORXXX | 08/11/2010 | OR | ASL ORISTANO | ovicaprini |
| 2010 | IT057CAXXX | 10/11/2010 | CA | ASL 8 CAGLIARI | ovicaprini |
| 2010 | IT072CAXXX | 18/11/2010 | CA | ASL 8 CAGLIARI | ovicaprini |
| 2010 | IT055CAXXX | 19/11/2010 | CA | ASL 8 CAGLIARI | ovicaprini |
| 2010 | IT057ORXXX | 22/11/2010 | OR | ASL ORISTANO | ovicaprini |
| 2010 | IT057CAXXX | 25/11/2010 | CA | ASL 8 CAGLIARI | ovicaprini |
| 2010 | IT057ORXXX | 29/11/2010 | OR | ASL ORISTANO | ovicaprini |
| 2010 | IT053CAXXX | 02/12/2010 | CA | ASL 8 CAGLIARI | ovicaprini |
| 2011 | IT057CAXXX | 10/01/2011 | CA | ASL 8 CAGLIARI | ovicaprini |
| 2011 | IT034NUXXX | 19/01/2011 | NU | ASL NUORO | ovicaprini |
| 2011 | IT065CAXXX | 31/01/2011 | CA | ASL 8 CAGLIARI | ovicaprini |
| 2011 | IT065CAXXX | 07/02/2011 | CA | ASL 8 CAGLIARI | ovicaprini |
| 2011 | IT057CAXXX | 09/02/2011 | CA | ASL 8 CAGLIARI | ovicaprini |
| 2011 | IT077CAXXX | 02/03/2011 | CA | ASL 8 CAGLIARI | ovicaprini |
| 2011 | IT036NUXXX | 09/03/2011 | NU | ASL NUORO | ovicaprini |
| 2011 | IT065CAXXX | 21/03/2011 | CA | ASL 8 CAGLIARI | ovicaprini |
| 2011 | IT036NUXXX | 21/03/2011 | NU | ASL NUORO | ovicaprini |
| 2011 | IT094CAXXX | 23/03/2011 | CA | ASL 8 CAGLIARI | ovicaprini |
| 2011 | IT081ORXXX | 23/03/2011 | OR | ASL ORISTANO | ovicaprini |
| 2011 | IT081ORXXX | 28/03/2011 | OR | ASL ORISTANO | ovicaprini |
| 2011 | IT072CAXXX | 04/01/2011 | CA | ASL 8 CAGLIARI | ovicaprini |
| 2011 | IT016CAXXX | 07/01/2011 | CA | ASL 8 CAGLIARI | ovicaprini |
| 2011 | IT057CAXXX | 11/01/2011 | CA | ASL 8 CAGLIARI | ovicaprini |
| 2011 | IT052CAXXX | 13/01/2011 | CA | ASL 8 CAGLIARI | ovicaprini |
| 2011 | IT052CAXXX | 13/01/2011 | CA | ASL 8 CAGLIARI | ovicaprini |
| 2011 | IT072CAXXX | 13/01/2011 | CA | ASL 8 CAGLIARI | ovicaprini |
| 2011 | IT052CAXXX | 20/01/2011 | CA | ASL 8 CAGLIARI | ovicaprini |
| 2011 | IT093CAXXX | 24/01/2011 | CA | ASL 8 CAGLIARI | ovicaprini |
| 2011 | IT036CAXXX | 01/02/2011 | CA | ASL 8 CAGLIARI | ovicaprini |
| 2011 | IT067CAXXX | 07/02/2011 | CA | ASL 8 CAGLIARI | ovicaprini |
| 2011 | IT093CAXXX | 07/02/2011 | CA | ASL 8 CAGLIARI | ovicaprini |
| 2011 | IT032CAXXX | 08/02/2011 | CA | ASL 8 CAGLIARI | ovicaprini |
| 2011 | IT093CAXXX | 08/02/2011 | CA | ASL 8 CAGLIARI | ovicaprini |
| 2011 | IT052CAXXX | 10/02/2011 | CA | ASL 8 CAGLIARI | ovicaprini |
| 2011 | IT052CAXXX | 11/02/2011 | CA | ASL 8 CAGLIARI | ovicaprini |
| 2011 | IT032CAXXX | 11/02/2011 | CA | ASL 8 CAGLIARI | ovicaprini |
| 2011 | IT091CAXXX | 14/02/2011 | CA | ASL 8 CAGLIARI | ovicaprini |
| 2011 | IT091CAXXX | 14/02/2011 | CA | ASL 8 CAGLIARI | ovicaprini |
| 2011 | IT025CAXXX | 14/02/2011 | CA | ASL 8 CAGLIARI | ovicaprini |
| 2011 | IT057CAXXX | 17/02/2011 | CA | ASL 8 CAGLIARI | ovicaprini |
| 2011 | IT057CAXXX | 18/02/2011 | CA | ASL 8 CAGLIARI | ovicaprini |
| 2011 | IT072CAXXX | 21/02/2011 | CA | ASL 8 CAGLIARI | ovicaprini |
| 2011 | IT032CAXXX | 22/02/2011 | CA | ASL 8 CAGLIARI | ovicaprini |
| 2011 | IT032CAXXX | 24/02/2011 | CA | ASL 8 CAGLIARI | ovicaprini |
| 2011 | IT032CAXXX | 25/02/2011 | CA | ASL 8 CAGLIARI | ovicaprini |
| 2011 | IT053CAXXX | 28/02/2011 | CA | ASL 8 CAGLIARI | ovicaprini |
| 2011 | IT067CAXXX | 03/03/2011 | CA | ASL 8 CAGLIARI | ovicaprini |
| 2011 | IT012ORXXX | 04/03/2011 | OR | ASL ORISTANO | ovicaprini |
| 2011 | IT096CAXXX | 07/03/2011 | CA | ASL 8 CAGLIARI | ovicaprini |
| 2011 | IT025CAXXX | 07/03/2011 | CA | ASL 8 CAGLIARI | ovicaprini |
| 2011 | IT025CAXXX | 07/03/2011 | CA | ASL 8 CAGLIARI | ovicaprini |
| 2011 | IT032CAXXX | 08/03/2011 | CA | ASL 8 CAGLIARI | ovicaprini |
| 2011 | IT032CAXXX | 11/03/2011 | CA | ASL 8 CAGLIARI | ovicaprini |
| 2011 | IT094CAXXX | 15/03/2011 | CA | ASL 8 CAGLIARI | ovicaprini |
| 2011 | IT094CAXXX | 15/03/2011 | CA | ASL 8 CAGLIARI | ovicaprini |
| 2011 | IT052CAXXX | 21/03/2011 | CA | ASL 8 CAGLIARI | ovicaprini |
| 2011 | IT032CAXXX | 21/03/2011 | CA | ASL 8 CAGLIARI | ovicaprini |
| 2011 | IT029CAXXX | 22/03/2011 | CA | ASL 8 CAGLIARI | ovicaprini |
| 2011 | IT019CAXXX | 24/03/2011 | CA | ASL 8 CAGLIARI | ovicaprini |
| 2011 | IT032CAXXX | 31/03/2011 | CA | ASL 8 CAGLIARI | ovicaprini |
| 2011 | IT032CAXXX | 31/03/2011 | CA | ASL 8 CAGLIARI | ovicaprini |
| 2011 | IT016CAXXX | 31/03/2011 | CA | ASL 8 CAGLIARI | ovicaprini |
| 2011 | 032CA10XXX | 10/01/2011 | CA | ASL 8 CAGLIARI | ovicaprini |
| 2011 | 049OR03XXX | 11/01/2011 | OR | ASL ORISTANO | ovicaprini |
| 2011 | 049OR17XXX | 11/01/2011 | OR | ASL ORISTANO | ovicaprini |
| 2011 | 049OR00XXX | 11/01/2011 | OR | ASL ORISTANO | ovicaprini |
| 2011 | 028OR04XXX | 17/01/2011 | OR | ASL ORISTANO | ovicaprini |
| 2011 | 032CA02XXX | 24/01/2011 | CA | ASL 8 CAGLIARI | ovicaprini |
| 2011 | 092CA06XXX | 24/01/2011 | CA | ASL 8 CAGLIARI | ovicaprini |
| 2011 | 057OR00XXX | 24/01/2011 | OR | ASL ORISTANO | ovicaprini |
| 2011 | 053OR01XXX | 25/01/2011 | OR | ASL ORISTANO | ovicaprini |
| 2011 | 015OR09XXX | 25/01/2011 | OR | ASL ORISTANO | ovicaprini |
| 2011 | 092CA12XXX | 31/01/2011 | CA | ASL 8 CAGLIARI | ovicaprini |
| 2011 | 092CA06XXX | 31/01/2011 | CA | ASL 8 CAGLIARI | ovicaprini |
| 2011 | 032CA10XXX | 31/01/2011 | CA | ASL 8 CAGLIARI | ovicaprini |
| 2011 | 029OR06XXX | 31/01/2011 | OR | ASL ORISTANO | ovicaprini |
| 2011 | 049OR02XXX | 01/02/2011 | OR | ASL ORISTANO | ovicaprini |
| 2011 | 049OR02XXX | 01/02/2011 | OR | ASL ORISTANO | ovicaprini |
| 2011 | 049OR02XXX | 01/02/2011 | OR | ASL ORISTANO | ovicaprini |
| 2011 | 049OR02XXX | 01/02/2011 | OR | ASL ORISTANO | ovicaprini |
| 2011 | 049OR02XXX | 01/02/2011 | OR | ASL ORISTANO | ovicaprini |
| 2011 | 053OR01XXX | 01/02/2011 | OR | ASL ORISTANO | ovicaprini |
| 2011 | 049OR14XXX | 01/02/2011 | OR | ASL ORISTANO | ovicaprini |
| 2011 | 092CA12XXX | 07/02/2011 | CA | ASL 8 CAGLIARI | ovicaprini |
| 2011 | 057OR03XXX | 07/02/2011 | OR | ASL ORISTANO | ovicaprini |
| 2011 | 048OR06XXX | 07/02/2011 | OR | ASL ORISTANO | ovicaprini |
| 2011 | 092CA06XXX | 14/02/2011 | CA | ASL 8 CAGLIARI | ovicaprini |
| 2011 | 069OR03XXX | 14/02/2011 | OR | ASL ORISTANO | ovicaprini |
| 2011 | 049OR14XXX | 15/02/2011 | OR | ASL ORISTANO | ovicaprini |
| 2011 | 049OR12XXX | 15/02/2011 | OR | ASL ORISTANO | ovicaprini |
| 2011 | 049OR12XXX | 15/02/2011 | OR | ASL ORISTANO | ovicaprini |
| 2011 | 049OR12XXX | 15/02/2011 | OR | ASL ORISTANO | ovicaprini |
| 2011 | 049OR12XXX | 15/02/2011 | OR | ASL ORISTANO | ovicaprini |
| 2011 | 049OR03XXX | 15/02/2011 | OR | ASL ORISTANO | ovicaprini |
| 2011 | 049OR00XXX | 15/02/2011 | OR | ASL ORISTANO | ovicaprini |
| 2011 | 078CA03XXX | 15/02/2011 | CA | ASL 8 CAGLIARI | ovicaprini |
| 2011 | 031OR02XXX | 15/02/2011 | OR | ASL ORISTANO | ovicaprini |
| 2011 | 031OR02XXX | 15/02/2011 | OR | ASL ORISTANO | ovicaprini |
| 2011 | 027OR02XXX | 15/02/2011 | OR | ASL ORISTANO | ovicaprini |
| 2011 | 053OR11XXX | 15/02/2011 | OR | ASL ORISTANO | ovicaprini |
| 2011 | 053OR09XXX | 15/02/2011 | OR | ASL ORISTANO | ovicaprini |
| 2011 | 053OR18XXX | 15/02/2011 | OR | ASL ORISTANO | ovicaprini |
| 2011 | 032CA04XXX | 16/02/2011 | CA | ASL 8 CAGLIARI | ovicaprini |
| 2011 | IT086CAXXX | 21/02/2011 | CA | ASL 8 CAGLIARI | ovicaprini |
| 2011 | IT032CAXXX | 21/02/2011 | CA | ASL 8 CAGLIARI | ovicaprini |
| 2011 | IT029CAXXX | 21/02/2011 | CA | ASL 8 CAGLIARI | ovicaprini |
| 2011 | IT047ORXXX | 21/02/2011 | OR | ASL ORISTANO | ovicaprini |
| 2011 | IT032CAXXX | 22/02/2011 | CA | ASL 8 CAGLIARI | ovicaprini |
| 2011 | IT092CAXXX | 22/02/2011 | CA | ASL 8 CAGLIARI | ovicaprini |
| 2011 | IT031ORXXX | 22/02/2011 | OR | ASL ORISTANO | ovicaprini |
| 2011 | IT066ORXXX | 22/02/2011 | OR | ASL ORISTANO | ovicaprini |
| 2011 | IT092CAXXX | 28/02/2011 | CA | ASL 8 CAGLIARI | ovicaprini |
| 2011 | IT092CAXXX | 28/02/2011 | CA | ASL 8 CAGLIARI | ovicaprini |
| 2011 | IT029CAXXX | 28/02/2011 | CA | ASL 8 CAGLIARI | ovicaprini |
| 2011 | IT055CAXXX | 02/03/2011 | CA | ASL 8 CAGLIARI | ovicaprini |
| 2011 | IT091CAXXX | 07/03/2011 | CA | ASL 8 CAGLIARI | ovicaprini |
| 2011 | IT096CAXXX | 07/03/2011 | CA | ASL 8 CAGLIARI | ovicaprini |
| 2011 | IT029CAXXX | 07/03/2011 | CA | ASL 8 CAGLIARI | ovicaprini |
| 2011 | IT029ORXXX | 07/03/2011 | OR | ASL ORISTANO | ovicaprini |
| 2011 | IT065CAXXX | 07/03/2011 | CA | ASL 8 CAGLIARI | ovicaprini |
| 2011 | IT029ORXXX | 07/03/2011 | OR | ASL ORISTANO | ovicaprini |
| 2011 | IT032CAXXX | 07/03/2011 | CA | ASL 8 CAGLIARI | ovicaprini |
| 2011 | IT057ORXXX | 07/03/2011 | OR | ASL ORISTANO | ovicaprini |
| 2011 | IT057ORXXX | 07/03/2011 | OR | ASL ORISTANO | ovicaprini |
| 2011 | IT032CAXXX | 08/03/2011 | CA | ASL 8 CAGLIARI | ovicaprini |
| 2011 | IT032CAXXX | 09/03/2011 | CA | ASL 8 CAGLIARI | ovicaprini |
| 2011 | IT096CAXXX | 14/03/2011 | CA | ASL 8 CAGLIARI | ovicaprini |
| 2011 | IT029CAXXX | 14/03/2011 | CA | ASL 8 CAGLIARI | ovicaprini |
| 2011 | IT092CAXXX | 14/03/2011 | CA | ASL 8 CAGLIARI | ovicaprini |
| 2011 | IT092CAXXX | 14/03/2011 | CA | ASL 8 CAGLIARI | ovicaprini |
| 2011 | IT096CAXXX | 14/03/2011 | CA | ASL 8 CAGLIARI | ovicaprini |
| 2011 | IT038ORXXX | 15/03/2011 | OR | ASL ORISTANO | ovicaprini |
| 2011 | IT053ORXXX | 15/03/2011 | OR | ASL ORISTANO | ovicaprini |
| 2011 | IT053ORXXX | 15/03/2011 | OR | ASL ORISTANO | ovicaprini |
| 2011 | IT032CAXXX | 15/03/2011 | CA | ASL 8 CAGLIARI | ovicaprini |
| 2011 | IT032CAXXX | 21/03/2011 | CA | ASL 8 CAGLIARI | ovicaprini |
| 2011 | IT029ORXXX | 21/03/2011 | OR | ASL ORISTANO | ovicaprini |
| 2011 | IT031CAXXX | 21/03/2011 | CA | ASL 8 CAGLIARI | ovicaprini |
| 2011 | IT092CAXXX | 21/03/2011 | CA | ASL 8 CAGLIARI | ovicaprini |
| 2011 | IT027ORXXX | 22/03/2011 | OR | ASL ORISTANO | ovicaprini |
| 2011 | IT028NUXXX | 22/03/2011 | NU | ASL NUORO | ovicaprini |
| 2011 | IT049ORXXX | 22/03/2011 | OR | ASL ORISTANO | ovicaprini |
| 2011 | IT055CAXXX | 22/03/2011 | CA | ASL 8 CAGLIARI | ovicaprini |
| 2011 | IT049ORXXX | 22/03/2011 | OR | ASL ORISTANO | ovicaprini |
| 2011 | IT049ORXXX | 22/03/2011 | OR | ASL ORISTANO | ovicaprini |
| 2011 | IT015ORXXX | 22/03/2011 | OR | ASL ORISTANO | ovicaprini |
| 2011 | IT049ORXXX | 22/03/2011 | OR | ASL ORISTANO | ovicaprini |
| 2011 | IT049ORXXX | 25/03/2011 | OR | ASL ORISTANO | ovicaprini |
| 2011 | IT049ORXXX | 25/03/2011 | OR | ASL ORISTANO | ovicaprini |
| 2011 | IT049ORXXX | 25/03/2011 | OR | ASL ORISTANO | ovicaprini |
| 2011 | IT049ORXXX | 25/03/2011 | OR | ASL ORISTANO | ovicaprini |
| 2011 | IT049ORXXX | 25/03/2011 | OR | ASL ORISTANO | ovicaprini |
| 2011 | IT049ORXXX | 25/03/2011 | OR | ASL ORISTANO | ovicaprini |
| 2011 | IT015ORXXX | 25/03/2011 | OR | ASL ORISTANO | ovicaprini |
| 2011 | IT043ORXXX | 25/03/2011 | OR | ASL ORISTANO | ovicaprini |
| 2011 | IT049ORXXX | 25/03/2011 | OR | ASL ORISTANO | ovicaprini |
| 2011 | IT012CAXXX | 28/03/2011 | CA | ASL 8 CAGLIARI | ovicaprini |
| 2011 | IT032CAXXX | 28/03/2011 | CA | ASL 8 CAGLIARI | ovicaprini |
| 2011 | IT029CAXXX | 28/03/2011 | CA | ASL 8 CAGLIARI | ovicaprini |
| 2011 | IT096CAXXX | 28/03/2011 | CA | ASL 8 CAGLIARI | ovicaprini |
| 2011 | IT029CAXXX | 28/03/2011 | CA | ASL 8 CAGLIARI | ovicaprini |
| 2011 | IT014CAXXX | 28/03/2011 | CA | ASL 8 CAGLIARI | ovicaprini |
| 2011 | IT026ORXXX | 28/03/2011 | OR | ASL ORISTANO | ovicaprini |
| 2011 | IT038ORXXX | 30/03/2011 | OR | ASL ORISTANO | ovicaprini |
| 2011 | IT049ORXXX | 30/03/2011 | OR | ASL ORISTANO | ovicaprini |
| 2011 | IT050ORXXX | 30/03/2011 | OR | ASL ORISTANO | ovicaprini |
| 2011 | IT055CAXXX | 30/03/2011 | CA | ASL 8 CAGLIARI | ovicaprini |
| 2011 | IT078CAXXX | 30/03/2011 | CA | ASL 8 CAGLIARI | ovicaprini |
| 2011 | IT032CAXXX | 30/03/2011 | CA | ASL 8 CAGLIARI | ovicaprini |
| 2011 | IT053ORXXX | 30/03/2011 | OR | ASL ORISTANO | ovicaprini |
| 2011 | 032CA07XXX | 10/01/2011 | CA | ASL 8 CAGLIARI | ovicaprini |
| 2011 | 032CA07XXX | 20/01/2011 | CA | ASL 8 CAGLIARI | ovicaprini |
| 2011 | 032CA19XXX | 21/01/2011 | CA | ASL 8 CAGLIARI | ovicaprini |
| 2011 | 032CA07XXX | 04/02/2011 | CA | ASL 8 CAGLIARI | ovicaprini |
| 2011 | 078CA03XXX | 04/03/2011 | CA | ASL 8 CAGLIARI | ovicaprini |
| 2011 | 078CA05XXX | 11/03/2011 | CA | ASL 8 CAGLIARI | ovicaprini |
| 2011 | 034NU02XXX | 18/03/2011 | NU | ASL NUORO | ovicaprini |
| 2011 | 032CA07XXX | 24/03/2011 | CA | ASL 8 CAGLIARI | ovicaprini |
| 2011 | 032CA07XXX | 24/03/2011 | CA | ASL 8 CAGLIARI | ovicaprini |
| 2011 | 078CA03XXX | 25/03/2011 | CA | ASL 8 CAGLIARI | ovicaprini |
| 2011 | 078CA05XXX | 25/03/2011 | CA | ASL 8 CAGLIARI | ovicaprini |
| 2011 | IT057CAXXX | 04/04/2011 | CA | ASL 8 CAGLIARI | ovicaprini |
| 2011 | IT081ORXXX | 06/04/2011 | OR | ASL ORISTANO | ovicaprini |
| 2011 | IT047ORXXX | 11/04/2011 | OR | ASL ORISTANO | ovicaprini |
| 2011 | IT086CAXXX | 11/04/2011 | CA | ASL 8 CAGLIARI | ovicaprini |
| 2011 | IT047ORXXX | 13/04/2011 | OR | ASL ORISTANO | ovicaprini |
| 2011 | IT086CAXXX | 26/04/2011 | CA | ASL 8 CAGLIARI | ovicaprini |
| 2011 | IT047ORXXX | 26/04/2011 | OR | ASL ORISTANO | ovicaprini |
| 2011 | IT047ORXXX | 02/05/2011 | OR | ASL ORISTANO | ovicaprini |
| 2011 | IT065CAXXX | 09/05/2011 | CA | ASL 8 CAGLIARI | ovicaprini |
| 2011 | IT024CAXXX | 09/05/2011 | CA | ASL 8 CAGLIARI | ovicaprini |
| 2011 | IT047ORXXX | 09/05/2011 | OR | ASL ORISTANO | ovicaprini |
| 2011 | IT057CAXXX | 09/05/2011 | CA | ASL 8 CAGLIARI | ovicaprini |
| 2011 | IT024CAXXX | 16/05/2011 | CA | ASL 8 CAGLIARI | ovicaprini |
| 2011 | IT057CAXXX | 16/05/2011 | CA | ASL 8 CAGLIARI | ovicaprini |
| 2011 | IT057CAXXX | 18/05/2011 | CA | ASL 8 CAGLIARI | ovicaprini |
| 2011 | IT057CAXXX | 23/05/2011 | CA | ASL 8 CAGLIARI | ovicaprini |
| 2011 | IT057CAXXX | 23/05/2011 | CA | ASL 8 CAGLIARI | ovicaprini |
| 2011 | IT057CAXXX | 25/05/2011 | CA | ASL 8 CAGLIARI | ovicaprini |
| 2011 | IT091CAXXX | 30/05/2011 | CA | ASL 8 CAGLIARI | ovicaprini |
| 2011 | IT091CAXXX | 31/05/2011 | CA | ASL 8 CAGLIARI | ovicaprini |
| 2011 | IT044ORXXX | 06/06/2011 | OR | ASL ORISTANO | ovicaprini |
| 2011 | IT091CAXXX | 06/06/2011 | CA | ASL 8 CAGLIARI | ovicaprini |
| 2011 | IT044ORXXX | 08/06/2011 | OR | ASL ORISTANO | ovicaprini |
| 2011 | IT052CAXXX | 13/06/2011 | CA | ASL 8 CAGLIARI | ovicaprini |
| 2011 | IT052CAXXX | 15/06/2011 | CA | ASL 8 CAGLIARI | ovicaprini |
| 2011 | IT052CAXXX | 20/06/2011 | CA | ASL 8 CAGLIARI | ovicaprini |
| 2011 | IT057CAXXX | 20/06/2011 | CA | ASL 8 CAGLIARI | ovicaprini |
| 2011 | IT094CAXXX | 22/06/2011 | CA | ASL 8 CAGLIARI | ovicaprini |
| 2011 | IT057CAXXX | 27/06/2011 | CA | ASL 8 CAGLIARI | ovicaprini |
| 2011 | IT065CAXXX | 27/06/2011 | CA | ASL 8 CAGLIARI | ovicaprini |
| 2011 | IT094CAXXX | 29/06/2011 | CA | ASL 8 CAGLIARI | ovicaprini |
| 2011 | IT096CAXXX | 04/04/2011 | CA | ASL 8 CAGLIARI | ovicaprini |
| 2011 | IT032CAXXX | 04/04/2011 | CA | ASL 8 CAGLIARI | ovicaprini |
| 2011 | IT091CAXXX | 04/04/2011 | CA | ASL 8 CAGLIARI | ovicaprini |
| 2011 | IT053CAXXX | 04/04/2011 | CA | ASL 8 CAGLIARI | ovicaprini |
| 2011 | IT057CAXXX | 05/04/2011 | CA | ASL 8 CAGLIARI | ovicaprini |
| 2011 | IT032CAXXX | 05/04/2011 | CA | ASL 8 CAGLIARI | ovicaprini |
| 2011 | IT066CAXXX | 05/04/2011 | CA | ASL 8 CAGLIARI | ovicaprini |
| 2011 | IT072CAXXX | 07/04/2011 | CA | ASL 8 CAGLIARI | ovicaprini |
| 2011 | IT025CAXXX | 11/04/2011 | CA | ASL 8 CAGLIARI | ovicaprini |
| 2011 | IT046CAXXX | 12/04/2011 | CA | ASL 8 CAGLIARI | ovicaprini |
| 2011 | IT046CAXXX | 12/04/2011 | CA | ASL 8 CAGLIARI | ovicaprini |
| 2011 | IT092CAXXX | 14/04/2011 | CA | ASL 8 CAGLIARI | ovicaprini |
| 2011 | IT057CAXXX | 18/04/2011 | CA | ASL 8 CAGLIARI | ovicaprini |
| 2011 | IT096CAXXX | 21/04/2011 | CA | ASL 8 CAGLIARI | ovicaprini |
| 2011 | IT092CAXXX | 21/04/2011 | CA | ASL 8 CAGLIARI | ovicaprini |
| 2011 | IT092CAXXX | 22/04/2011 | CA | ASL 8 CAGLIARI | ovicaprini |
| 2011 | IT072CAXXX | 26/04/2011 | CA | ASL 8 CAGLIARI | ovicaprini |
| 2011 | IT057CAXXX | 28/04/2011 | CA | ASL 8 CAGLIARI | ovicaprini |
| 2011 | IT052CAXXX | 28/04/2011 | CA | ASL 8 CAGLIARI | ovicaprini |
| 2011 | IT057CAXXX | 02/05/2011 | CA | ASL 8 CAGLIARI | ovicaprini |
| 2011 | IT072CAXXX | 03/05/2011 | CA | ASL 8 CAGLIARI | ovicaprini |
| 2011 | IT012ORXXX | 05/05/2011 | OR | ASL ORISTANO | ovicaprini |
| 2011 | IT092CAXXX | 05/05/2011 | CA | ASL 8 CAGLIARI | ovicaprini |
| 2011 | IT020CAXXX | 05/05/2011 | CA | ASL 8 CAGLIARI | ovicaprini |
| 2011 | IT020CAXXX | 06/05/2011 | CA | ASL 8 CAGLIARI | ovicaprini |
| 2011 | IT025CAXXX | 09/05/2011 | CA | ASL 8 CAGLIARI | ovicaprini |
| 2011 | IT006CAXXX | 09/05/2011 | CA | ASL 8 CAGLIARI | ovicaprini |
| 2011 | IT016CAXXX | 09/05/2011 | CA | ASL 8 CAGLIARI | ovicaprini |
| 2011 | IT016CAXXX | 10/05/2011 | CA | ASL 8 CAGLIARI | ovicaprini |
| 2011 | IT072CAXXX | 12/05/2011 | CA | ASL 8 CAGLIARI | ovicaprini |
| 2011 | IT072CAXXX | 12/05/2011 | CA | ASL 8 CAGLIARI | ovicaprini |
| 2011 | IT020CAXXX | 16/05/2011 | CA | ASL 8 CAGLIARI | ovicaprini |
| 2011 | IT020CAXXX | 16/05/2011 | CA | ASL 8 CAGLIARI | ovicaprini |
| 2011 | IT092CAXXX | 16/05/2011 | CA | ASL 8 CAGLIARI | ovicaprini |
| 2011 | IT057CAXXX | 17/05/2011 | CA | ASL 8 CAGLIARI | ovicaprini |
| 2011 | IT057CAXXX | 17/05/2011 | CA | ASL 8 CAGLIARI | ovicaprini |
| 2011 | IT092CAXXX | 17/05/2011 | CA | ASL 8 CAGLIARI | ovicaprini |
| 2011 | IT092CAXXX | 19/05/2011 | CA | ASL 8 CAGLIARI | ovicaprini |
| 2011 | IT091CAXXX | 19/05/2011 | CA | ASL 8 CAGLIARI | ovicaprini |
| 2011 | IT091CAXXX | 20/05/2011 | CA | ASL 8 CAGLIARI | ovicaprini |
| 2011 | IT025CAXXX | 23/05/2011 | CA | ASL 8 CAGLIARI | ovicaprini |
| 2011 | IT091CAXXX | 23/05/2011 | CA | ASL 8 CAGLIARI | ovicaprini |
| 2011 | IT092CAXXX | 24/05/2011 | CA | ASL 8 CAGLIARI | ovicaprini |
| 2011 | IT053CAXXX | 26/05/2011 | CA | ASL 8 CAGLIARI | ovicaprini |
| 2011 | IT091CAXXX | 27/05/2011 | CA | ASL 8 CAGLIARI | ovicaprini |
| 2011 | IT072CAXXX | 27/05/2011 | CA | ASL 8 CAGLIARI | ovicaprini |
| 2011 | IT091CAXXX | 30/05/2011 | CA | ASL 8 CAGLIARI | ovicaprini |
| 2011 | IT057CAXXX | 30/05/2011 | CA | ASL 8 CAGLIARI | ovicaprini |
| 2011 | IT057CAXXX | 30/05/2011 | CA | ASL 8 CAGLIARI | ovicaprini |
| 2011 | IT012CAXXX | 31/05/2011 | CA | ASL 8 CAGLIARI | ovicaprini |
| 2011 | IT012ORXXX | 31/05/2011 | OR | ASL ORISTANO | ovicaprini |
| 2011 | IT019CAXXX | 03/06/2011 | CA | ASL 8 CAGLIARI | ovicaprini |
| 2011 | IT057CAXXX | 06/06/2011 | CA | ASL 8 CAGLIARI | ovicaprini |
| 2011 | IT016CAXXX | 06/06/2011 | CA | ASL 8 CAGLIARI | ovicaprini |
| 2011 | IT057CAXXX | 06/06/2011 | CA | ASL 8 CAGLIARI | ovicaprini |
| 2011 | IT087CAXXX | 07/06/2011 | CA | ASL 8 CAGLIARI | ovicaprini |
| 2011 | IT087CAXXX | 07/06/2011 | CA | ASL 8 CAGLIARI | ovicaprini |
| 2011 | IT072CAXXX | 09/06/2011 | CA | ASL 8 CAGLIARI | ovicaprini |
| 2011 | IT072CAXXX | 10/06/2011 | CA | ASL 8 CAGLIARI | ovicaprini |
| 2011 | IT072CAXXX | 10/06/2011 | CA | ASL 8 CAGLIARI | ovicaprini |
| 2011 | IT087CAXXX | 13/06/2011 | CA | ASL 8 CAGLIARI | ovicaprini |
| 2011 | IT012CAXXX | 14/06/2011 | CA | ASL 8 CAGLIARI | ovicaprini |
| 2011 | IT087CAXXX | 14/06/2011 | CA | ASL 8 CAGLIARI | ovicaprini |
| 2011 | IT092CAXXX | 16/06/2011 | CA | ASL 8 CAGLIARI | ovicaprini |
| 2011 | IT030NUXXX | 16/06/2011 | NU | ASL NUORO | ovicaprini |
| 2011 | IT030NUXXX | 17/06/2011 | NU | ASL NUORO | ovicaprini |
| 2011 | IT030NUXXX | 20/06/2011 | NU | ASL NUORO | ovicaprini |
| 2011 | IT055CAXXX | 20/06/2011 | CA | ASL 8 CAGLIARI | ovicaprini |
| 2011 | IT032CAXXX | 21/06/2011 | CA | ASL 8 CAGLIARI | ovicaprini |
| 2011 | IT072CAXXX | 23/06/2011 | CA | ASL 8 CAGLIARI | ovicaprini |
| 2011 | IT034NUXXX | 23/06/2011 | NU | ASL NUORO | ovicaprini |
| 2011 | IT057CAXXX | 27/06/2011 | CA | ASL 8 CAGLIARI | ovicaprini |
| 2011 | IT057CAXXX | 27/06/2011 | CA | ASL 8 CAGLIARI | ovicaprini |
| 2011 | IT093CAXXX | 27/06/2011 | CA | ASL 8 CAGLIARI | ovicaprini |
| 2011 | IT093CAXXX | 27/06/2011 | CA | ASL 8 CAGLIARI | ovicaprini |
| 2011 | IT032CAXXX | 28/06/2011 | CA | ASL 8 CAGLIARI | ovicaprini |
| 2011 | IT032CAXXX | 28/06/2011 | CA | ASL 8 CAGLIARI | ovicaprini |
| 2011 | IT016CAXXX | 30/06/2011 | CA | ASL 8 CAGLIARI | ovicaprini |
| 2011 | IT072CAXXX | 30/06/2011 | CA | ASL 8 CAGLIARI | ovicaprini |
| 2011 | IT077ORXXX | 01/04/2011 | OR | ASL ORISTANO | ovicaprini |
| 2011 | IT029ORXXX | 01/04/2011 | OR | ASL ORISTANO | ovicaprini |
| 2011 | IT029ORXXX | 01/04/2011 | OR | ASL ORISTANO | ovicaprini |
| 2011 | IT091CAXXX | 01/04/2011 | CA | ASL 8 CAGLIARI | ovicaprini |
| 2011 | IT044ORXXX | 01/04/2011 | OR | ASL ORISTANO | ovicaprini |
| 2011 | IT091CAXXX | 01/04/2011 | CA | ASL 8 CAGLIARI | ovicaprini |
| 2011 | IT029ORXXX | 01/04/2011 | OR | ASL ORISTANO | ovicaprini |
| 2011 | IT032CAXXX | 01/04/2011 | CA | ASL 8 CAGLIARI | ovicaprini |
| 2011 | IT032CAXXX | 04/04/2011 | CA | ASL 8 CAGLIARI | ovicaprini |
| 2011 | IT092CAXXX | 04/04/2011 | CA | ASL 8 CAGLIARI | ovicaprini |
| 2011 | IT026ORXXX | 04/04/2011 | OR | ASL ORISTANO | ovicaprini |
| 2011 | IT096CAXXX | 04/04/2011 | CA | ASL 8 CAGLIARI | ovicaprini |
| 2011 | IT066ORXXX | 05/04/2011 | OR | ASL ORISTANO | ovicaprini |
| 2011 | IT032CAXXX | 05/04/2011 | CA | ASL 8 CAGLIARI | ovicaprini |
| 2011 | IT032CAXXX | 06/04/2011 | CA | ASL 8 CAGLIARI | ovicaprini |
| 2011 | IT014CAXXX | 11/04/2011 | CA | ASL 8 CAGLIARI | ovicaprini |
| 2011 | IT014CAXXX | 11/04/2011 | CA | ASL 8 CAGLIARI | ovicaprini |
| 2011 | IT077ORXXX | 11/04/2011 | OR | ASL ORISTANO | ovicaprini |
| 2011 | IT032CAXXX | 11/04/2011 | CA | ASL 8 CAGLIARI | ovicaprini |
| 2011 | IT092CAXXX | 11/04/2011 | CA | ASL 8 CAGLIARI | ovicaprini |
| 2011 | IT039ORXXX | 12/04/2011 | OR | ASL ORISTANO | ovicaprini |
| 2011 | IT086CAXXX | 17/04/2011 | CA | ASL 8 CAGLIARI | ovicaprini |
| 2011 | IT096CAXXX | 18/04/2011 | CA | ASL 8 CAGLIARI | ovicaprini |
| 2011 | IT039ORXXX | 19/04/2011 | OR | ASL ORISTANO | ovicaprini |
| 2011 | IT001CAXXX | 20/04/2011 | CA | ASL 8 CAGLIARI | ovicaprini |
| 2011 | IT032CAXXX | 20/04/2011 | CA | ASL 8 CAGLIARI | ovicaprini |
| 2011 | IT055CAXXX | 26/04/2011 | CA | ASL 8 CAGLIARI | ovicaprini |
| 2011 | IT001CAXXX | 29/04/2011 | CA | ASL 8 CAGLIARI | ovicaprini |
| 2011 | IT065CAXXX | 29/04/2011 | CA | ASL 8 CAGLIARI | ovicaprini |
| 2011 | IT032CAXXX | 02/05/2011 | CA | ASL 8 CAGLIARI | ovicaprini |
| 2011 | IT032CAXXX | 02/05/2011 | CA | ASL 8 CAGLIARI | ovicaprini |
| 2011 | IT057ORXXX | 02/05/2011 | OR | ASL ORISTANO | ovicaprini |
| 2011 | IT092CAXXX | 02/05/2011 | CA | ASL 8 CAGLIARI | ovicaprini |
| 2011 | IT031CAXXX | 02/05/2011 | CA | ASL 8 CAGLIARI | ovicaprini |
| 2011 | IT045CAXXX | 02/05/2011 | CA | ASL 8 CAGLIARI | ovicaprini |
| 2011 | IT012ORXXX | 09/05/2011 | OR | ASL ORISTANO | ovicaprini |
| 2011 | IT046ORXXX | 09/05/2011 | OR | ASL ORISTANO | ovicaprini |
| 2011 | IT045CAXXX | 09/05/2011 | CA | ASL 8 CAGLIARI | ovicaprini |
| 2011 | IT012ORXXX | 09/05/2011 | OR | ASL ORISTANO | ovicaprini |
| 2011 | IT092CAXXX | 09/05/2011 | CA | ASL 8 CAGLIARI | ovicaprini |
| 2011 | IT031CAXXX | 09/05/2011 | CA | ASL 8 CAGLIARI | ovicaprini |
| 2011 | IT001CAXXX | 09/05/2011 | CA | ASL 8 CAGLIARI | ovicaprini |
| 2011 | IT056ORXXX | 10/05/2011 | OR | ASL ORISTANO | ovicaprini |
| 2011 | IT029ORXXX | 11/05/2011 | OR | ASL ORISTANO | ovicaprini |
| 2011 | IT091CAXXX | 16/05/2011 | CA | ASL 8 CAGLIARI | ovicaprini |
| 2011 | IT032CAXXX | 16/05/2011 | CA | ASL 8 CAGLIARI | ovicaprini |
| 2011 | IT031CAXXX | 16/05/2011 | CA | ASL 8 CAGLIARI | ovicaprini |
| 2011 | IT001CAXXX | 16/05/2011 | CA | ASL 8 CAGLIARI | ovicaprini |
| 2011 | IT045CAXXX | 16/05/2011 | CA | ASL 8 CAGLIARI | ovicaprini |
| 2011 | IT045CAXXX | 16/05/2011 | CA | ASL 8 CAGLIARI | ovicaprini |
| 2011 | IT045CAXXX | 16/05/2011 | CA | ASL 8 CAGLIARI | ovicaprini |
| 2011 | IT055CAXXX | 16/05/2011 | CA | ASL 8 CAGLIARI | ovicaprini |
| 2011 | IT045CAXXX | 16/05/2011 | CA | ASL 8 CAGLIARI | ovicaprini |
| 2011 | IT032CAXXX | 16/05/2011 | CA | ASL 8 CAGLIARI | ovicaprini |
| 2011 | IT057ORXXX | 17/05/2011 | OR | ASL ORISTANO | ovicaprini |
| 2011 | IT032CAXXX | 23/05/2011 | CA | ASL 8 CAGLIARI | ovicaprini |
| 2011 | IT092CAXXX | 23/05/2011 | CA | ASL 8 CAGLIARI | ovicaprini |
| 2011 | IT045CAXXX | 23/05/2011 | CA | ASL 8 CAGLIARI | ovicaprini |
| 2011 | IT069CAXXX | 23/05/2011 | CA | ASL 8 CAGLIARI | ovicaprini |
| 2011 | IT042ORXXX | 23/05/2011 | OR | ASL ORISTANO | ovicaprini |
| 2011 | IT029ORXXX | 23/05/2011 | OR | ASL ORISTANO | ovicaprini |
| 2011 | IT039ORXXX | 24/05/2011 | OR | ASL ORISTANO | ovicaprini |
| 2011 | IT029CAXXX | 25/05/2011 | CA | ASL 8 CAGLIARI | ovicaprini |
| 2011 | IT078CAXXX | 27/05/2011 | CA | ASL 8 CAGLIARI | ovicaprini |
| 2011 | IT094CAXXX | 27/05/2011 | CA | ASL 8 CAGLIARI | ovicaprini |
| 2011 | IT101CAXXX | 27/05/2011 | CA | ASL 8 CAGLIARI | ovicaprini |
| 2011 | IT094CAXXX | 27/05/2011 | CA | ASL 8 CAGLIARI | ovicaprini |
| 2011 | IT055CAXXX | 30/05/2011 | CA | ASL 8 CAGLIARI | ovicaprini |
| 2011 | IT045CAXXX | 30/05/2011 | CA | ASL 8 CAGLIARI | ovicaprini |
| 2011 | IT069CAXXX | 30/05/2011 | CA | ASL 8 CAGLIARI | ovicaprini |
| 2011 | IT091CAXXX | 30/05/2011 | CA | ASL 8 CAGLIARI | ovicaprini |
| 2011 | IT014CAXXX | 30/05/2011 | CA | ASL 8 CAGLIARI | ovicaprini |
| 2011 | IT071ORXXX | 31/05/2011 | OR | ASL ORISTANO | ovicaprini |
| 2011 | IT039ORXXX | 31/05/2011 | OR | ASL ORISTANO | ovicaprini |
| 2011 | IT046ORXXX | 01/06/2011 | OR | ASL ORISTANO | ovicaprini |
| 2011 | IT006CAXXX | 01/06/2011 | CA | ASL 8 CAGLIARI | ovicaprini |
| 2011 | IT006CAXXX | 01/06/2011 | CA | ASL 8 CAGLIARI | ovicaprini |
| 2011 | IT006CAXXX | 01/06/2011 | CA | ASL 8 CAGLIARI | ovicaprini |
| 2011 | IT069CAXXX | 06/06/2011 | CA | ASL 8 CAGLIARI | ovicaprini |
| 2011 | IT045CAXXX | 06/06/2011 | CA | ASL 8 CAGLIARI | ovicaprini |
| 2011 | IT091CAXXX | 06/06/2011 | CA | ASL 8 CAGLIARI | ovicaprini |
| 2011 | IT043ORXXX | 06/06/2011 | OR | ASL ORISTANO | ovicaprini |
| 2011 | IT032CAXXX | 07/06/2011 | CA | ASL 8 CAGLIARI | ovicaprini |
| 2011 | IT032CAXXX | 07/06/2011 | CA | ASL 8 CAGLIARI | ovicaprini |
| 2011 | IT086CAXXX | 13/06/2011 | CA | ASL 8 CAGLIARI | ovicaprini |
| 2011 | IT091CAXXX | 13/06/2011 | CA | ASL 8 CAGLIARI | ovicaprini |
| 2011 | IT086CAXXX | 13/06/2011 | CA | ASL 8 CAGLIARI | ovicaprini |
| 2011 | IT045CAXXX | 13/06/2011 | CA | ASL 8 CAGLIARI | ovicaprini |
| 2011 | IT029ORXXX | 13/06/2011 | OR | ASL ORISTANO | ovicaprini |
| 2011 | IT096CAXXX | 13/06/2011 | CA | ASL 8 CAGLIARI | ovicaprini |
| 2011 | IT004ORXXX | 14/06/2011 | OR | ASL ORISTANO | ovicaprini |
| 2011 | IT032CAXXX | 15/06/2011 | CA | ASL 8 CAGLIARI | ovicaprini |
| 2011 | IT057ORXXX | 20/06/2011 | OR | ASL ORISTANO | ovicaprini |
| 2011 | IT057CAXXX | 20/06/2011 | CA | ASL 8 CAGLIARI | ovicaprini |
| 2011 | IT069CAXXX | 20/06/2011 | CA | ASL 8 CAGLIARI | ovicaprini |
| 2011 | IT065CAXXX | 20/06/2011 | CA | ASL 8 CAGLIARI | ovicaprini |
| 2011 | IT091CAXXX | 20/06/2011 | CA | ASL 8 CAGLIARI | ovicaprini |
| 2011 | IT046ORXXX | 20/06/2011 | OR | ASL ORISTANO | ovicaprini |
| 2011 | IT042ORXXX | 20/06/2011 | OR | ASL ORISTANO | ovicaprini |
| 2011 | IT057ORXXX | 20/06/2011 | OR | ASL ORISTANO | ovicaprini |
| 2011 | IT069CAXXX | 27/06/2011 | CA | ASL 8 CAGLIARI | ovicaprini |
| 2011 | IT091CAXXX | 27/06/2011 | CA | ASL 8 CAGLIARI | ovicaprini |
| 2011 | IT042ORXXX | 27/06/2011 | OR | ASL ORISTANO | ovicaprini |
| 2011 | IT014CAXXX | 27/06/2011 | CA | ASL 8 CAGLIARI | ovicaprini |
| 2011 | IT057CAXXX | 27/06/2011 | CA | ASL 8 CAGLIARI | ovicaprini |
| 2011 | IT029ORXXX | 27/06/2011 | OR | ASL ORISTANO | ovicaprini |
| 2011 | IT001CAXXX | 01/04/2011 | CA | ASL 8 CAGLIARI | ovicaprini |
| 2011 | IT001CAXXX | 04/04/2011 | CA | ASL 8 CAGLIARI | ovicaprini |
| 2011 | IT029CAXXX | 19/04/2011 | CA | ASL 8 CAGLIARI | ovicaprini |
| 2011 | IT078CAXXX | 20/05/2011 | CA | ASL 8 CAGLIARI | ovicaprini |
| 2011 | IT060CAXXX | 27/05/2011 | CA | ASL 8 CAGLIARI | ovicaprini |
| 2011 | IT092CAXXX | 27/05/2011 | CA | ASL 8 CAGLIARI | ovicaprini |
| 2011 | IT001CAXXX | 27/05/2011 | CA | ASL 8 CAGLIARI | ovicaprini |
| 2011 | IT001CAXXX | 27/05/2011 | CA | ASL 8 CAGLIARI | ovicaprini |
| 2011 | IT060CAXXX | 03/06/2011 | CA | ASL 8 CAGLIARI | ovicaprini |
| 2011 | IT057CAXXX | 17/06/2011 | CA | ASL 8 CAGLIARI | ovicaprini |
| 2011 | IT057CAXXX | 17/06/2011 | CA | ASL 8 CAGLIARI | ovicaprini |
| 2011 | IT032CAXXX | 17/06/2011 | CA | ASL 8 CAGLIARI | ovicaprini |
| 2011 | IT032CAXXX | 17/06/2011 | CA | ASL 8 CAGLIARI | ovicaprini |
| 2011 | IT001CAXXX | 24/06/2011 | CA | ASL 8 CAGLIARI | ovicaprini |
| 2011 | IT065CAXXX | 04/07/2011 | CA | ASL 8 CAGLIARI | ovicaprini |
| 2011 | IT057CAXXX | 06/07/2011 | CA | ASL 8 CAGLIARI | ovicaprini |
| 2011 | IT065CAXXX | 11/07/2011 | CA | ASL 8 CAGLIARI | ovicaprini |
| 2011 | IT047ORXXX | 18/07/2011 | OR | ASL ORISTANO | ovicaprini |
| 2011 | IT057CAXXX | 18/07/2011 | CA | ASL 8 CAGLIARI | ovicaprini |
| 2011 | IT024CAXXX | 25/07/2011 | CA | ASL 8 CAGLIARI | ovicaprini |
| 2011 | IT001CAXXX | 25/07/2011 | CA | ASL 8 CAGLIARI | ovicaprini |
| 2011 | IT095CAXXX | 27/07/2011 | CA | ASL 8 CAGLIARI | ovicaprini |
| 2011 | IT077CAXXX | 27/07/2011 | CA | ASL 8 CAGLIARI | ovicaprini |
| 2011 | IT077CAXXX | 27/07/2011 | CA | ASL 8 CAGLIARI | ovicaprini |
| 2011 | IT024CAXXX | 01/08/2011 | CA | ASL 8 CAGLIARI | ovicaprini |
| 2011 | IT057CAXXX | 03/08/2011 | CA | ASL 8 CAGLIARI | ovicaprini |
| 2011 | IT024CAXXX | 08/08/2011 | CA | ASL 8 CAGLIARI | ovicaprini |
| 2011 | IT057CAXXX | 08/08/2011 | CA | ASL 8 CAGLIARI | ovicaprini |
| 2011 | IT057CAXXX | 08/08/2011 | CA | ASL 8 CAGLIARI | ovicaprini |
| 2011 | IT077CAXXX | 10/08/2011 | CA | ASL 8 CAGLIARI | ovicaprini |
| 2011 | IT077CAXXX | 16/08/2011 | CA | ASL 8 CAGLIARI | ovicaprini |
| 2011 | IT024CAXXX | 16/08/2011 | CA | ASL 8 CAGLIARI | ovicaprini |
| 2011 | IT057CAXXX | 16/08/2011 | CA | ASL 8 CAGLIARI | ovicaprini |
| 2011 | IT057CAXXX | 17/08/2011 | CA | ASL 8 CAGLIARI | ovicaprini |
| 2011 | IT057CAXXX | 22/08/2011 | CA | ASL 8 CAGLIARI | ovicaprini |
| 2011 | IT001CAXXX | 29/08/2011 | CA | ASL 8 CAGLIARI | ovicaprini |
| 2011 | IT035CAXXX | 31/08/2011 | CA | ASL 8 CAGLIARI | ovicaprini |
| 2011 | IT065CAXXX | 05/09/2011 | CA | ASL 8 CAGLIARI | ovicaprini |
| 2011 | IT086CAXXX | 07/09/2011 | CA | ASL 8 CAGLIARI | ovicaprini |
| 2011 | IT072CAXXX | 01/07/2011 | CA | ASL 8 CAGLIARI | ovicaprini |
| 2011 | IT025CAXXX | 04/07/2011 | CA | ASL 8 CAGLIARI | ovicaprini |
| 2011 | IT072CAXXX | 04/07/2011 | CA | ASL 8 CAGLIARI | ovicaprini |
| 2011 | IT073CAXXX | 04/07/2011 | CA | ASL 8 CAGLIARI | ovicaprini |
| 2011 | IT052CAXXX | 05/07/2011 | CA | ASL 8 CAGLIARI | ovicaprini |
| 2011 | IT052CAXXX | 05/07/2011 | CA | ASL 8 CAGLIARI | ovicaprini |
| 2011 | IT016CAXXX | 07/07/2011 | CA | ASL 8 CAGLIARI | ovicaprini |
| 2011 | IT016CAXXX | 07/07/2011 | CA | ASL 8 CAGLIARI | ovicaprini |
| 2011 | IT019CAXXX | 07/07/2011 | CA | ASL 8 CAGLIARI | ovicaprini |
| 2011 | IT096CAXXX | 08/07/2011 | CA | ASL 8 CAGLIARI | ovicaprini |
| 2011 | IT052CAXXX | 11/07/2011 | CA | ASL 8 CAGLIARI | ovicaprini |
| 2011 | IT072CAXXX | 11/07/2011 | CA | ASL 8 CAGLIARI | ovicaprini |
| 2011 | IT072CAXXX | 11/07/2011 | CA | ASL 8 CAGLIARI | ovicaprini |
| 2011 | IT034NUXXX | 12/07/2011 | NU | ASL NUORO | ovicaprini |
| 2011 | IT057CAXXX | 12/07/2011 | CA | ASL 8 CAGLIARI | ovicaprini |
| 2011 | IT057CAXXX | 12/07/2011 | CA | ASL 8 CAGLIARI | ovicaprini |
| 2011 | IT057CAXXX | 12/07/2011 | CA | ASL 8 CAGLIARI | ovicaprini |
| 2011 | IT072CAXXX | 14/07/2011 | CA | ASL 8 CAGLIARI | ovicaprini |
| 2011 | IT025CAXXX | 18/07/2011 | CA | ASL 8 CAGLIARI | ovicaprini |
| 2011 | IT078CAXXX | 18/07/2011 | CA | ASL 8 CAGLIARI | ovicaprini |
| 2011 | IT078CAXXX | 19/07/2011 | CA | ASL 8 CAGLIARI | ovicaprini |
| 2011 | IT078CAXXX | 19/07/2011 | CA | ASL 8 CAGLIARI | ovicaprini |
| 2011 | IT078CAXXX | 21/07/2011 | CA | ASL 8 CAGLIARI | ovicaprini |
| 2011 | IT009ORXXX | 21/07/2011 | OR | ASL ORISTANO | ovicaprini |
| 2011 | IT069CAXXX | 21/07/2011 | CA | ASL 8 CAGLIARI | ovicaprini |
| 2011 | IT069CAXXX | 22/07/2011 | CA | ASL 8 CAGLIARI | ovicaprini |
| 2011 | IT069CAXXX | 25/07/2011 | CA | ASL 8 CAGLIARI | ovicaprini |
| 2011 | IT016CAXXX | 25/07/2011 | CA | ASL 8 CAGLIARI | ovicaprini |
| 2011 | IT016CAXXX | 25/07/2011 | CA | ASL 8 CAGLIARI | ovicaprini |
| 2011 | IT016CAXXX | 26/07/2011 | CA | ASL 8 CAGLIARI | ovicaprini |
| 2011 | IT057CAXXX | 29/07/2011 | CA | ASL 8 CAGLIARI | ovicaprini |
| 2011 | IT057CAXXX | 01/08/2011 | CA | ASL 8 CAGLIARI | ovicaprini |
| 2011 | IT016CAXXX | 02/08/2011 | CA | ASL 8 CAGLIARI | ovicaprini |
| 2011 | IT016CAXXX | 04/08/2011 | CA | ASL 8 CAGLIARI | ovicaprini |
| 2011 | IT091CAXXX | 05/08/2011 | CA | ASL 8 CAGLIARI | ovicaprini |
| 2011 | IT091CAXXX | 08/08/2011 | CA | ASL 8 CAGLIARI | ovicaprini |
| 2011 | IT006CAXXX | 08/08/2011 | CA | ASL 8 CAGLIARI | ovicaprini |
| 2011 | IT066CAXXX | 09/08/2011 | CA | ASL 8 CAGLIARI | ovicaprini |
| 2011 | IT006CAXXX | 09/08/2011 | CA | ASL 8 CAGLIARI | ovicaprini |
| 2011 | IT091CAXXX | 16/08/2011 | CA | ASL 8 CAGLIARI | ovicaprini |
| 2011 | IT091CAXXX | 16/08/2011 | CA | ASL 8 CAGLIARI | ovicaprini |
| 2011 | IT012ORXXX | 17/08/2011 | OR | ASL ORISTANO | ovicaprini |
| 2011 | IT040CAXXX | 22/08/2011 | CA | ASL 8 CAGLIARI | ovicaprini |
| 2011 | IT094CAXXX | 22/08/2011 | CA | ASL 8 CAGLIARI | ovicaprini |
| 2011 | IT094CAXXX | 23/08/2011 | CA | ASL 8 CAGLIARI | ovicaprini |
| 2011 | IT057CAXXX | 25/08/2011 | CA | ASL 8 CAGLIARI | ovicaprini |
| 2011 | IT032CAXXX | 25/08/2011 | CA | ASL 8 CAGLIARI | ovicaprini |
| 2011 | IT046CAXXX | 25/08/2011 | CA | ASL 8 CAGLIARI | ovicaprini |
| 2011 | IT046CAXXX | 25/08/2011 | CA | ASL 8 CAGLIARI | ovicaprini |
| 2011 | IT046CAXXX | 25/08/2011 | CA | ASL 8 CAGLIARI | ovicaprini |
| 2011 | IT046CAXXX | 26/08/2011 | CA | ASL 8 CAGLIARI | ovicaprini |
| 2011 | IT032CAXXX | 26/08/2011 | CA | ASL 8 CAGLIARI | ovicaprini |
| 2011 | IT012CAXXX | 01/09/2011 | CA | ASL 8 CAGLIARI | ovicaprini |
| 2011 | IT069CAXXX | 05/09/2011 | CA | ASL 8 CAGLIARI | ovicaprini |
| 2011 | IT096CAXXX | 15/09/2011 | CA | ASL 8 CAGLIARI | ovicaprini |
| 2011 | IT073CAXXX | 16/09/2011 | CA | ASL 8 CAGLIARI | ovicaprini |
| 2011 | IT087CAXXX | 19/09/2011 | CA | ASL 8 CAGLIARI | ovicaprini |
| 2011 | IT087CAXXX | 19/09/2011 | CA | ASL 8 CAGLIARI | ovicaprini |
| 2011 | IT057CAXXX | 26/09/2011 | CA | ASL 8 CAGLIARI | ovicaprini |
| 2011 | IT029ORXXX | 04/07/2011 | OR | ASL ORISTANO | ovicaprini |
| 2011 | IT032CAXXX | 04/07/2011 | CA | ASL 8 CAGLIARI | ovicaprini |
| 2011 | IT057CAXXX | 04/07/2011 | CA | ASL 8 CAGLIARI | ovicaprini |
| 2011 | IT012ORXXX | 04/07/2011 | OR | ASL ORISTANO | ovicaprini |
| 2011 | IT069CAXXX | 04/07/2011 | CA | ASL 8 CAGLIARI | ovicaprini |
| 2011 | IT011ORXXX | 06/07/2011 | OR | ASL ORISTANO | ovicaprini |
| 2011 | IT032CAXXX | 08/07/2011 | CA | ASL 8 CAGLIARI | ovicaprini |
| 2011 | IT069CAXXX | 11/07/2011 | CA | ASL 8 CAGLIARI | ovicaprini |
| 2011 | IT092CAXXX | 11/07/2011 | CA | ASL 8 CAGLIARI | ovicaprini |
| 2011 | IT057CAXXX | 11/07/2011 | CA | ASL 8 CAGLIARI | ovicaprini |
| 2011 | IT032CAXXX | 11/07/2011 | CA | ASL 8 CAGLIARI | ovicaprini |
| 2011 | IT032CAXXX | 11/07/2011 | CA | ASL 8 CAGLIARI | ovicaprini |
| 2011 | IT001CAXXX | 11/07/2011 | CA | ASL 8 CAGLIARI | ovicaprini |
| 2011 | IT069CAXXX | 18/07/2011 | CA | ASL 8 CAGLIARI | ovicaprini |
| 2011 | IT057CAXXX | 18/07/2011 | CA | ASL 8 CAGLIARI | ovicaprini |
| 2011 | IT032CAXXX | 18/07/2011 | CA | ASL 8 CAGLIARI | ovicaprini |
| 2011 | IT060ORXXX | 18/07/2011 | OR | ASL ORISTANO | ovicaprini |
| 2011 | IT014CAXXX | 18/07/2011 | CA | ASL 8 CAGLIARI | ovicaprini |
| 2011 | IT091CAXXX | 18/07/2011 | CA | ASL 8 CAGLIARI | ovicaprini |
| 2011 | IT086CAXXX | 18/07/2011 | CA | ASL 8 CAGLIARI | ovicaprini |
| 2011 | IT014CAXXX | 18/07/2011 | CA | ASL 8 CAGLIARI | ovicaprini |
| 2011 | IT028ORXXX | 19/07/2011 | OR | ASL ORISTANO | ovicaprini |
| 2011 | IT032CAXXX | 19/07/2011 | CA | ASL 8 CAGLIARI | ovicaprini |
| 2011 | IT001CAXXX | 25/07/2011 | CA | ASL 8 CAGLIARI | ovicaprini |
| 2011 | IT057CAXXX | 25/07/2011 | CA | ASL 8 CAGLIARI | ovicaprini |
| 2011 | IT089CAXXX | 25/07/2011 | CA | ASL 8 CAGLIARI | ovicaprini |
| 2011 | IT070CAXXX | 25/07/2011 | CA | ASL 8 CAGLIARI | ovicaprini |
| 2011 | IT012ORXXX | 25/07/2011 | OR | ASL ORISTANO | ovicaprini |
| 2011 | IT091CAXXX | 25/07/2011 | CA | ASL 8 CAGLIARI | ovicaprini |
| 2011 | IT091CAXXX | 25/07/2011 | CA | ASL 8 CAGLIARI | ovicaprini |
| 2011 | IT035CAXXX | 25/07/2011 | CA | ASL 8 CAGLIARI | ovicaprini |
| 2011 | IT086CAXXX | 01/08/2011 | CA | ASL 8 CAGLIARI | ovicaprini |
| 2011 | IT057CAXXX | 01/08/2011 | CA | ASL 8 CAGLIARI | ovicaprini |
| 2011 | IT048CAXXX | 01/08/2011 | CA | ASL 8 CAGLIARI | ovicaprini |
| 2011 | IT069CAXXX | 01/08/2011 | CA | ASL 8 CAGLIARI | ovicaprini |
| 2011 | IT077CAXXX | 01/08/2011 | CA | ASL 8 CAGLIARI | ovicaprini |
| 2011 | IT092CAXXX | 01/08/2011 | CA | ASL 8 CAGLIARI | ovicaprini |
| 2011 | IT077CAXXX | 01/08/2011 | CA | ASL 8 CAGLIARI | ovicaprini |
| 2011 | IT032CAXXX | 02/08/2011 | CA | ASL 8 CAGLIARI | ovicaprini |
| 2011 | IT057CAXXX | 09/08/2011 | CA | ASL 8 CAGLIARI | ovicaprini |
| 2011 | IT057CAXXX | 09/08/2011 | CA | ASL 8 CAGLIARI | ovicaprini |
| 2011 | IT039ORXXX | 09/08/2011 | OR | ASL ORISTANO | ovicaprini |
| 2011 | IT001CAXXX | 09/08/2011 | CA | ASL 8 CAGLIARI | ovicaprini |
| 2011 | IT032CAXXX | 09/08/2011 | CA | ASL 8 CAGLIARI | ovicaprini |
| 2011 | IT055CAXXX | 09/08/2011 | CA | ASL 8 CAGLIARI | ovicaprini |
| 2011 | IT057CAXXX | 09/08/2011 | CA | ASL 8 CAGLIARI | ovicaprini |
| 2011 | IT057CAXXX | 16/08/2011 | CA | ASL 8 CAGLIARI | ovicaprini |
| 2011 | IT039ORXXX | 16/08/2011 | OR | ASL ORISTANO | ovicaprini |
| 2011 | IT070CAXXX | 16/08/2011 | CA | ASL 8 CAGLIARI | ovicaprini |
| 2011 | IT029ORXXX | 16/08/2011 | OR | ASL ORISTANO | ovicaprini |
| 2011 | IT029ORXXX | 16/08/2011 | OR | ASL ORISTANO | ovicaprini |
| 2011 | IT067NUXXX | 22/08/2011 | NU | ASL NUORO | ovicaprini |
| 2011 | IT012ORXXX | 22/08/2011 | OR | ASL ORISTANO | ovicaprini |
| 2011 | IT029ORXXX | 22/08/2011 | OR | ASL ORISTANO | ovicaprini |
| 2011 | IT096CAXXX | 23/08/2011 | CA | ASL 8 CAGLIARI | ovicaprini |
| 2011 | IT096CAXXX | 23/08/2011 | CA | ASL 8 CAGLIARI | ovicaprini |
| 2011 | IT042ORXXX | 29/08/2011 | OR | ASL ORISTANO | ovicaprini |
| 2011 | IT032CAXXX | 31/08/2011 | CA | ASL 8 CAGLIARI | ovicaprini |
| 2011 | IT086CAXXX | 12/09/2011 | CA | ASL 8 CAGLIARI | ovicaprini |
| 2011 | IT029ORXXX | 19/09/2011 | OR | ASL ORISTANO | ovicaprini |
| 2011 | IT086CAXXX | 26/09/2011 | CA | ASL 8 CAGLIARI | ovicaprini |
| 2011 | IT080NUXXX | 26/09/2011 | NU | ASL NUORO | ovicaprini |
| 2011 | IT012ORXXX | 26/09/2011 | OR | ASL ORISTANO | ovicaprini |
| 2011 | IT033CAXXX | 01/07/2011 | CA | ASL 8 CAGLIARI | ovicaprini |
| 2011 | IT001CAXXX | 08/07/2011 | CA | ASL 8 CAGLIARI | ovicaprini |
| 2011 | IT092CAXXX | 08/07/2011 | CA | ASL 8 CAGLIARI | ovicaprini |
| 2011 | IT001CAXXX | 08/07/2011 | CA | ASL 8 CAGLIARI | ovicaprini |
| 2011 | IT001CAXXX | 29/07/2011 | CA | ASL 8 CAGLIARI | ovicaprini |
| 2011 | IT001CAXXX | 26/08/2011 | CA | ASL 8 CAGLIARI | ovicaprini |
| 2011 | IT001CAXXX | 09/09/2011 | CA | ASL 8 CAGLIARI | ovicaprini |
| 2011 | IT069ORXXX | 09/09/2011 | OR | ASL ORISTANO | ovicaprini |
| 2011 | IT057CAXXX | 17/10/2011 | CA | ASL 8 CAGLIARI | ovicaprini |
| 2011 | IT057CAXXX | 28/10/2011 | CA | ASL 8 CAGLIARI | ovicaprini |
| 2011 | IT057CAXXX | 14/11/2011 | CA | ASL 8 CAGLIARI | ovicaprini |
| 2011 | IT057CAXXX | 07/12/2011 | CA | ASL 8 CAGLIARI | ovicaprini |
| 2011 | IT092CAXXX | 13/10/2011 | CA | ASL 8 CAGLIARI | ovicaprini |
| 2011 | IT032CAXXX | 25/10/2011 | CA | ASL 8 CAGLIARI | ovicaprini |
| 2011 | IT032CAXXX | 27/10/2011 | CA | ASL 8 CAGLIARI | ovicaprini |
| 2011 | IT032CAXXX | 28/10/2011 | CA | ASL 8 CAGLIARI | ovicaprini |
| 2011 | IT032CAXXX | 10/11/2011 | CA | ASL 8 CAGLIARI | ovicaprini |
| 2011 | IT032CAXXX | 11/11/2011 | CA | ASL 8 CAGLIARI | ovicaprini |
| 2011 | IT020CAXXX | 15/11/2011 | CA | ASL 8 CAGLIARI | ovicaprini |
| 2011 | IT072CAXXX | 14/12/2011 | CA | ASL 8 CAGLIARI | ovicaprini |
| 2011 | IT072CAXXX | 28/12/2011 | CA | ASL 8 CAGLIARI | ovicaprini |
| 2011 | IT023ORXXX | 04/10/2011 | OR | ASL ORISTANO | ovicaprini |
| 2011 | IT032CAXXX | 10/10/2011 | CA | ASL 8 CAGLIARI | ovicaprini |
| 2011 | IT032CAXXX | 10/10/2011 | CA | ASL 8 CAGLIARI | ovicaprini |
| 2011 | IT029ORXXX | 14/10/2011 | OR | ASL ORISTANO | ovicaprini |
| 2011 | IT006CAXXX | 17/10/2011 | CA | ASL 8 CAGLIARI | ovicaprini |
| 2011 | IT032CAXXX | 09/11/2011 | CA | ASL 8 CAGLIARI | ovicaprini |
| 2011 | 009NU13XXX | 02/05/2011 | NU | ASL NUORO | ovicaprini |
| 2011 | 073NU01XXX | 04/05/2011 | NU | ASL NUORO | ovicaprini |
| 2011 | 018SS06XXX | 06/05/2011 | SS | ASL 1 SASSARI | ovicaprini |
| 2011 | 036NU17XXX | 08/05/2011 | NU | ASL NUORO | ovicaprini |
| 2011 | 036NU17XXX | 10/05/2011 | NU | ASL NUORO | ovicaprini |
| 2011 | 036NU17XXX | 12/05/2011 | NU | ASL NUORO | ovicaprini |
| 2011 | 036NU17XXX | 14/05/2011 | NU | ASL NUORO | ovicaprini |
| 2011 | 036NU17XXX | 16/05/2011 | NU | ASL NUORO | ovicaprini |
| 2011 | 036NU17XXX | 18/05/2011 | NU | ASL NUORO | ovicaprini |
| 2011 | 036NU17XXX | 20/05/2011 | NU | ASL NUORO | ovicaprini |
| 2011 | 012NU12XXX | 22/05/2011 | NU | ASL NUORO | ovicaprini |
| 2011 | 049OR02XXX | 24/05/2011 | OR | ASL ORISTANO | ovicaprini |
| 2011 | 049SS08XXX | 26/05/2011 | SS | ASL 1 SASSARI | ovicaprini |
| 2011 | 071SS08XXX | 28/05/2011 | SS | ASL 1 SASSARI | ovicaprini |
| 2011 | 055SS27XXX | 30/05/2011 | SS | ASL 1 SASSARI | ovicaprini |
| 2011 | 044NU17XXX | 01/06/2011 | NU | ASL NUORO | ovicaprini |
| 2011 | 024SS12XXX | 03/06/2011 | SS | ASL 1 SASSARI | ovicaprini |
| 2011 | 045SS03XXX | 05/06/2011 | SS | ASL 1 SASSARI | ovicaprini |
| 2011 | 045SS03XXX | 07/06/2011 | SS | ASL 1 SASSARI | ovicaprini |
| 2011 | 045SS03XXX | 09/06/2011 | SS | ASL 1 SASSARI | ovicaprini |
| 2011 | 017NU19XXX | 11/06/2011 | NU | ASL NUORO | ovicaprini |
| 2011 | 027NU08XXX | 13/06/2011 | NU | ASL NUORO | ovicaprini |
| 2011 | 017NU11XXX | 15/06/2011 | NU | ASL NUORO | ovicaprini |
| 2011 | 027NU13XXX | 17/06/2011 | NU | ASL NUORO | ovicaprini |
| 2011 | 067NU11XXX | 19/06/2011 | NU | ASL NUORO | ovicaprini |
| 2011 | 061NU02XXX | 21/06/2011 | NU | ASL NUORO | ovicaprini |
| 2011 | 077NU05XXX | 23/06/2011 | NU | ASL NUORO | ovicaprini |
| 2011 | 052OR17XXX | 25/06/2011 | OR | ASL ORISTANO | ovicaprini |
| 2011 | 016SS00XXX | 27/06/2011 | SS | ASL 1 SASSARI | ovicaprini |
| 2011 | 052OR18XXX | 29/06/2011 | OR | ASL ORISTANO | ovicaprini |
| 2011 | 077NU05XXX | 01/07/2011 | NU | ASL NUORO | ovicaprini |
| 2011 | 077NU00XXX | 03/07/2011 | NU | ASL NUORO | ovicaprini |
| 2011 | 052OR11XXX | 05/07/2011 | OR | ASL ORISTANO | ovicaprini |
| 2011 | 010NU01XXX | 07/07/2011 | NU | ASL NUORO | ovicaprini |
| 2011 | 046NU00XXX | 09/07/2011 | NU | ASL NUORO | ovicaprini |
| 2011 | 061NU11XXX | 11/07/2011 | NU | ASL NUORO | ovicaprini |
| 2011 | 057NU01XXX | 13/07/2011 | NU | ASL NUORO | ovicaprini |
| 2011 | 057NU01XXX | 15/07/2011 | NU | ASL NUORO | ovicaprini |
| 2011 | 077NU05XXX | 17/07/2011 | NU | ASL NUORO | ovicaprini |
| 2011 | 052OR17XXX | 19/07/2011 | OR | ASL ORISTANO | ovicaprini |
| 2011 | 077NU05XXX | 21/07/2011 | NU | ASL NUORO | ovicaprini |
| 2011 | 010NU01XXX | 23/07/2011 | NU | ASL NUORO | ovicaprini |
| 2011 | 010NU01XXX | 25/07/2011 | NU | ASL NUORO | ovicaprini |
| 2011 | 077NU01XXX | 27/07/2011 | NU | ASL NUORO | ovicaprini |
| 2011 | 052OR07XXX | 29/07/2011 | OR | ASL ORISTANO | ovicaprini |
| 2011 | 052OR15XXX | 31/07/2011 | OR | ASL ORISTANO | ovicaprini |
| 2011 | 077NU05XXX | 02/08/2011 | NU | ASL NUORO | ovicaprini |
| 2011 | 046NU00XXX | 04/08/2011 | NU | ASL NUORO | ovicaprini |
| 2011 | 055SS02XXX | 06/08/2011 | SS | ASL 1 SASSARI | ovicaprini |
| 2011 | 055SS02XXX | 08/08/2011 | SS | ASL 1 SASSARI | ovicaprini |
| 2011 | 052OR18XXX | 10/08/2011 | OR | ASL ORISTANO | ovicaprini |
| 2011 | 052OR17XXX | 12/08/2011 | OR | ASL ORISTANO | ovicaprini |
| 2011 | 062NU19XXX | 14/08/2011 | NU | ASL NUORO | ovicaprini |
| 2011 | 061NU02XXX | 16/08/2011 | NU | ASL NUORO | ovicaprini |
| 2011 | 061NU02XXX | 18/08/2011 | NU | ASL NUORO | ovicaprini |
| 2011 | 077NU09XXX | 20/08/2011 | NU | ASL NUORO | ovicaprini |
| 2011 | 052OR17XXX | 22/08/2011 | OR | ASL ORISTANO | ovicaprini |
| 2011 | 077NU00XXX | 24/08/2011 | NU | ASL NUORO | ovicaprini |
| 2011 | 077NU00XXX | 26/08/2011 | NU | ASL NUORO | ovicaprini |
| 2011 | 062NU19XXX | 28/08/2011 | NU | ASL NUORO | ovicaprini |
| 2011 | 046NU00XXX | 30/08/2011 | NU | ASL NUORO | ovicaprini |
| 2011 | 008SS05XXX | 01/09/2011 | SS | ASL 1 SASSARI | ovicaprini |
| 2011 | 008SS05XXX | 03/09/2011 | SS | ASL 1 SASSARI | ovicaprini |
| 2011 | 052OR09XXX | 05/09/2011 | OR | ASL ORISTANO | ovicaprini |
| 2011 | 024SS02XXX | 07/09/2011 | SS | ASL 1 SASSARI | ovicaprini |
| 2011 | 018SS07XXX | 09/09/2011 | SS | ASL 1 SASSARI | ovicaprini |
| 2011 | 018SS07XXX | 11/09/2011 | SS | ASL 1 SASSARI | ovicaprini |
| 2011 | 008SS07XXX | 13/09/2011 | SS | ASL 1 SASSARI | ovicaprini |
| 2011 | 044NU04XXX | 15/09/2011 | NU | ASL NUORO | ovicaprini |
| 2011 | 044NU04XXX | 17/09/2011 | NU | ASL NUORO | ovicaprini |
| 2011 | 044NU04XXX | 19/09/2011 | NU | ASL NUORO | ovicaprini |
| 2011 | 024SS02XXX | 21/09/2011 | SS | ASL 1 SASSARI | ovicaprini |
| 2011 | 024SS02XXX | 23/09/2011 | SS | ASL 1 SASSARI | ovicaprini |
| 2011 | 008SS05XXX | 25/09/2011 | SS | ASL 1 SASSARI | ovicaprini |
| 2011 | 077NU06XXX | 27/09/2011 | NU | ASL NUORO | ovicaprini |
| 2011 | 077NU06XXX | 29/09/2011 | NU | ASL NUORO | ovicaprini |
| 2011 | 077NU06XXX | 01/10/2011 | NU | ASL NUORO | ovicaprini |
| 2011 | 077NU00XXX | 03/10/2011 | NU | ASL NUORO | ovicaprini |
| 2011 | 077NU00XXX | 05/10/2011 | NU | ASL NUORO | ovicaprini |
| 2011 | 046NU00XXX | 07/10/2011 | NU | ASL NUORO | ovicaprini |
| 2011 | 062NU02XXX | 09/10/2011 | NU | ASL NUORO | ovicaprini |
| 2011 | 062NU19XXX | 11/10/2011 | NU | ASL NUORO | ovicaprini |
| 2011 | 077NU06XXX | 13/10/2011 | NU | ASL NUORO | ovicaprini |
| 2011 | 077NU06XXX | 15/10/2011 | NU | ASL NUORO | ovicaprini |
| 2011 | 052OR15XXX | 17/10/2011 | OR | ASL ORISTANO | ovicaprini |
| 2011 | 077NU00XXX | 19/10/2011 | NU | ASL NUORO | ovicaprini |
| 2011 | 077NU00XXX | 21/10/2011 | NU | ASL NUORO | ovicaprini |
| 2011 | 062NU02XXX | 23/10/2011 | NU | ASL NUORO | ovicaprini |
| 2011 | 052OR11XXX | 25/10/2011 | OR | ASL ORISTANO | ovicaprini |
| 2011 | 052OR11XXX | 27/10/2011 | OR | ASL ORISTANO | ovicaprini |
| 2011 | 046NU00XXX | 29/10/2011 | NU | ASL NUORO | ovicaprini |
| 2011 | 061NU10XXX | 31/10/2011 | NU | ASL NUORO | ovicaprini |
| 2011 | 052OR15XXX | 02/11/2011 | OR | ASL ORISTANO | ovicaprini |
| 2011 | 077NU05XXX | 04/11/2011 | NU | ASL NUORO | ovicaprini |
| 2011 | 024NU00XXX | 06/11/2011 | NU | ASL NUORO | ovicaprini |
| 2011 | 077NU00XXX | 08/11/2011 | NU | ASL NUORO | ovicaprini |
| 2011 | 077NU00XXX | 10/11/2011 | NU | ASL NUORO | ovicaprini |
| 2011 | 062NU14XXX | 12/11/2011 | NU | ASL NUORO | ovicaprini |
| 2011 | 062NU11XXX | 14/11/2011 | NU | ASL NUORO | ovicaprini |
| 2011 | 052OR11XXX | 16/11/2011 | OR | ASL ORISTANO | ovicaprini |
| 2011 | 052OR11XXX | 18/11/2011 | OR | ASL ORISTANO | ovicaprini |
| 2011 | 046NU00XXX | 20/11/2011 | NU | ASL NUORO | ovicaprini |
| 2011 | 077NU10XXX | 22/11/2011 | NU | ASL NUORO | ovicaprini |
| 2011 | 016SS00XXX | 24/11/2011 | SS | ASL 1 SASSARI | ovicaprini |
| 2011 | 061NU09XXX | 26/11/2011 | NU | ASL NUORO | ovicaprini |
| 2011 | 041OR26XXX | 28/11/2011 | OR | ASL ORISTANO | ovicaprini |
| 2011 | 061NU14XXX | 30/11/2011 | NU | ASL NUORO | ovicaprini |
| 2011 | 052OR00XXX | 02/12/2011 | OR | ASL ORISTANO | ovicaprini |
| 2011 | 046NU00XXX | 04/12/2011 | NU | ASL NUORO | ovicaprini |
| 2011 | 024NU05XXX | 06/12/2011 | NU | ASL NUORO | ovicaprini |
| 2011 | 012NU02XXX | 08/12/2011 | NU | ASL NUORO | ovicaprini |
| 2011 | 061NU06XXX | 10/12/2011 | NU | ASL NUORO | ovicaprini |
| 2011 | 062NU14XXX | 12/12/2011 | NU | ASL NUORO | ovicaprini |
| 2011 | 077NU05XXX | 14/12/2011 | NU | ASL NUORO | ovicaprini |
| 2011 | 077NU00XXX | 16/12/2011 | NU | ASL NUORO | ovicaprini |
| 2011 | 049OR0XXX | 18/12/2011 | OR | ASL ORISTANO | ovicaprini |
| 2011 | 024SS02XXX | 20/12/2011 | SS | ASL 1 SASSARI | ovicaprini |
| 2011 | 052OR02XXX | 22/12/2011 | OR | ASL ORISTANO | ovicaprini |
| 2011 | 042SS02XXX | 24/12/2011 | SS | ASL 1 SASSARI | ovicaprini |
| 2011 | 053SS05XXX | 26/12/2011 | SS | ASL 1 SASSARI | ovicaprini |
| 2011 | 055SS05XXX | 28/12/2011 | SS | ASL 1 SASSARI | ovicaprini |
| 2011 | 077NU00XXX | 30/12/2011 | NU | ASL NUORO | ovicaprini |
| 2011 | 061NU16XXX | 01/01/2011 | NU | ASL NUORO | ovicaprini |
| 2011 | 062NU36XXX | 03/01/2011 | NU | ASL NUORO | ovicaprini |
| 2011 | 061NU02XXX | 05/01/2011 | NU | ASL NUORO | ovicaprini |
| 2011 | 061NU07XXX | 07/01/2011 | NU | ASL NUORO | ovicaprini |
| 2011 | 061NU02XXX | 09/01/2011 | NU | ASL NUORO | ovicaprini |
| 2011 | 077NU06XXX | 11/01/2011 | NU | ASL NUORO | ovicaprini |
| 2011 | 077NU01XXX | 13/01/2011 | NU | ASL NUORO | ovicaprini |
| 2011 | 052OR17XXX | 15/01/2011 | OR | ASL ORISTANO | ovicaprini |
| 2011 | 052OR17XXX | 17/01/2011 | OR | ASL ORISTANO | ovicaprini |
| 2011 | 046NU00XXX | 19/01/2011 | NU | ASL NUORO | ovicaprini |
| 2011 | 077NU05XXX | 21/01/2011 | NU | ASL NUORO | ovicaprini |
| 2011 | 061NU14XXX | 23/01/2011 | NU | ASL NUORO | ovicaprini |
| 2011 | 077NU00XXX | 25/01/2011 | NU | ASL NUORO | ovicaprini |
| 2011 | 024NU22XXX | 27/01/2011 | NU | ASL NUORO | ovicaprini |
| 2011 | 052OR17XXX | 29/01/2011 | OR | ASL ORISTANO | ovicaprini |
| 2011 | 052OR17XXX | 31/01/2011 | OR | ASL ORISTANO | ovicaprini |
| 2011 | 062NU02XXX | 02/02/2011 | NU | ASL NUORO | ovicaprini |
| 2011 | 062NU13XXX | 04/02/2011 | NU | ASL NUORO | ovicaprini |
| 2011 | 061NU15XXX | 06/02/2011 | NU | ASL NUORO | ovicaprini |
| 2011 | 057NU01XXX | 08/02/2011 | NU | ASL NUORO | ovicaprini |
| 2011 | 06INU04XXX | 10/02/2011 | NU | ASL NUORO | ovicaprini |
| 2011 | 077NU06XXX | 12/02/2011 | NU | ASL NUORO | ovicaprini |
| 2011 | 052OR17XXX | 14/02/2011 | OR | ASL ORISTANO | ovicaprini |
| 2011 | 077NU00XXX | 16/02/2011 | NU | ASL NUORO | ovicaprini |
| 2011 | 024NU22XXX | 18/02/2011 | NU | ASL NUORO | ovicaprini |
| 2011 | 062NU13XXX | 20/02/2011 | NU | ASL NUORO | ovicaprini |
| 2011 | 046NU00XXX | 22/02/2011 | NU | ASL NUORO | ovicaprini |
| 2011 | 061NU14XXX | 24/02/2011 | NU | ASL NUORO | ovicaprini |
| 2011 | 077NU06XXX | 26/02/2011 | NU | ASL NUORO | ovicaprini |
| 2011 | 077NU05XXX | 28/02/2011 | NU | ASL NUORO | ovicaprini |
| 2011 | 062NU13XXX | 02/03/2011 | NU | ASL NUORO | ovicaprini |
| 2011 | 062NU02XXX | 04/03/2011 | NU | ASL NUORO | ovicaprini |
| 2011 | 046NU00XXX | 06/03/2011 | NU | ASL NUORO | ovicaprini |
| 2011 | 024NU22XXX | 08/03/2011 | NU | ASL NUORO | ovicaprini |
| 2011 | 077NU00XXX | 10/03/2011 | NU | ASL NUORO | ovicaprini |
| 2011 | 024NU18XXX | 12/03/2011 | NU | ASL NUORO | ovicaprini |
| 2011 | 052OR11XXX | 14/03/2011 | OR | ASL ORISTANO | ovicaprini |
| 2011 | 052OR11XXX | 16/03/2011 | OR | ASL ORISTANO | ovicaprini |
| 2011 | 008SS05XXX | 18/03/2011 | SS | ASL 1 SASSARI | ovicaprini |
| 2011 | 021OR10XXX | 20/03/2011 | OR | ASL ORISTANO | ovicaprini |
| 2011 | 021OR10XXX | 22/03/2011 | OR | ASL ORISTANO | ovicaprini |
| 2011 | 021OR10XXX | 24/03/2011 | OR | ASL ORISTANO | ovicaprini |
| 2011 | 021OR00XXX | 26/03/2011 | OR | ASL ORISTANO | ovicaprini |
| 2011 | 045SS03XXX | 28/03/2011 | SS | ASL 1 SASSARI | ovicaprini |
| 2011 | 024SS02XXX | 30/03/2011 | SS | ASL 1 SASSARI | ovicaprini |
| 2011 | 044NU17XXX | 01/04/2011 | NU | ASL NUORO | ovicaprini |
| 2011 | 044NU17XXX | 03/04/2011 | NU | ASL NUORO | ovicaprini |
| 2011 | 061NU14XXX | 05/04/2011 | NU | ASL NUORO | ovicaprini |
| 2011 | 061NU10XXX | 07/04/2011 | NU | ASL NUORO | ovicaprini |
| 2011 | 024NU14XXX | 09/04/2011 | NU | ASL NUORO | ovicaprini |
| 2011 | 077NU06XXX | 11/04/2011 | NU | ASL NUORO | ovicaprini |
| 2011 | 024NU22XXX | 13/04/2011 | NU | ASL NUORO | ovicaprini |
| 2011 | 077NU04XXX | 15/04/2011 | NU | ASL NUORO | ovicaprini |
| 2011 | 062NU12XXX | 17/04/2011 | NU | ASL NUORO | ovicaprini |
| 2011 | 062NU13XXX | 19/04/2011 | NU | ASL NUORO | ovicaprini |
| 2011 | 061NU02XXX | 21/04/2011 | NU | ASL NUORO | ovicaprini |
| 2011 | 061NU07XXX | 23/04/2011 | NU | ASL NUORO | ovicaprini |
| 2011 | 008NU00XXX | 25/04/2011 | NU | ASL NUORO | ovicaprini |
| 2011 | 024NU14XXX | 27/04/2011 | NU | ASL NUORO | ovicaprini |
| 2011 | 024NU08XXX | 29/04/2011 | NU | ASL NUORO | ovicaprini |
| 2011 | 077NU00XXX | 01/05/2011 | NU | ASL NUORO | ovicaprini |
| 2011 | 024NU02XXX | 03/05/2011 | NU | ASL NUORO | ovicaprini |
| 2011 | 024NU11XXX | 05/05/2011 | NU | ASL NUORO | ovicaprini |
| 2011 | 062NU13XXX | 07/05/2011 | NU | ASL NUORO | ovicaprini |
| 2011 | 046NU00XXX | 09/05/2011 | NU | ASL NUORO | ovicaprini |
| 2011 | 009NU00XXX | 11/05/2011 | NU | ASL NUORO | ovicaprini |
| 2011 | 061NU07XXX | 13/05/2011 | NU | ASL NUORO | ovicaprini |
| 2011 | 077NU00XXX | 15/05/2011 | NU | ASL NUORO | ovicaprini |
| 2011 | 077NU03XXX | 17/05/2011 | NU | ASL NUORO | ovicaprini |
| 2011 | 062NU38XXX | 19/05/2011 | NU | ASL NUORO | ovicaprini |
| 2011 | 061NU15XXX | 21/05/2011 | NU | ASL NUORO | ovicaprini |
| 2011 | 024NU01XXX | 23/05/2011 | NU | ASL NUORO | ovicaprini |
| 2011 | 061NU03XXX | 25/05/2011 | NU | ASL NUORO | ovicaprini |
| 2011 | 061NU10XXX | 27/05/2011 | NU | ASL NUORO | ovicaprini |
| 2011 | 061NU03XXX | 29/05/2011 | NU | ASL NUORO | ovicaprini |
| 2011 | 061NU07XXX | 31/05/2011 | NU | ASL NUORO | ovicaprini |
| 2011 | 046NU00XXX | 02/06/2011 | NU | ASL NUORO | ovicaprini |
| 2011 | 024NU22XXX | 04/06/2011 | NU | ASL NUORO | ovicaprini |
| 2011 | 077NU06XXX | 06/06/2011 | NU | ASL NUORO | ovicaprini |
| 2011 | 077NU09XXX | 08/06/2011 | NU | ASL NUORO | ovicaprini |
| 2011 | 077NU07XXX | 10/06/2011 | NU | ASL NUORO | ovicaprini |
| 2011 | 077NU07XXX | 12/06/2011 | NU | ASL NUORO | ovicaprini |
| 2011 | 077NU07XXX | 14/06/2011 | NU | ASL NUORO | ovicaprini |
| 2011 | 077NU06XXX | 16/06/2011 | NU | ASL NUORO | ovicaprini |
| 2011 | 008NU00XXX | 18/06/2011 | NU | ASL NUORO | ovicaprini |
| 2011 | 062NU02XXX | 20/06/2011 | NU | ASL NUORO | ovicaprini |
| 2011 | 027NU03XXX | 22/06/2011 | NU | ASL NUORO | ovicaprini |
| 2011 | 061NU02XXX | 24/06/2011 | NU | ASL NUORO | ovicaprini |
| 2011 | 061NU02XXX | 26/06/2011 | NU | ASL NUORO | ovicaprini |
| 2011 | 008NU00XXX | 28/06/2011 | NU | ASL NUORO | ovicaprini |
| 2011 | 062NU39XXX | 30/06/2011 | NU | ASL NUORO | ovicaprini |
| 2011 | 062NU14XXX | 02/07/2011 | NU | ASL NUORO | ovicaprini |
| 2011 | 024NU22XXX | 04/07/2011 | NU | ASL NUORO | ovicaprini |
| 2011 | 024NU22XXX | 06/07/2011 | NU | ASL NUORO | ovicaprini |
| 2011 | 077NU00XXX | 08/07/2011 | NU | ASL NUORO | ovicaprini |
| 2011 | 061NU02XXX | 10/07/2011 | NU | ASL NUORO | ovicaprini |
| 2011 | 024NU08XXX | 12/07/2011 | NU | ASL NUORO | ovicaprini |
| 2011 | 024NU14XXX | 14/07/2011 | NU | ASL NUORO | ovicaprini |
| 2011 | 024NU22XXX | 16/07/2011 | NU | ASL NUORO | ovicaprini |
| 2011 | 024NU22XXX | 18/07/2011 | NU | ASL NUORO | ovicaprini |
| 2011 | 052OR17XXX | 20/07/2011 | OR | ASL ORISTANO | ovicaprini |
| 2011 | 021OR10XXX | 22/07/2011 | OR | ASL ORISTANO | ovicaprini |
| 2011 | 062NU14XXX | 24/07/2011 | NU | ASL NUORO | ovicaprini |
| 2011 | 062NU39XXX | 26/07/2011 | NU | ASL NUORO | ovicaprini |
| 2011 | 061NU02XXX | 28/07/2011 | NU | ASL NUORO | ovicaprini |
| 2011 | 062NU02XXX | 30/07/2011 | NU | ASL NUORO | ovicaprini |
| 2011 | 024NU14XXX | 01/08/2011 | NU | ASL NUORO | ovicaprini |
| 2011 | 024NU22XXX | 03/08/2011 | NU | ASL NUORO | ovicaprini |
| 2011 | 062NU39XXX | 05/08/2011 | NU | ASL NUORO | ovicaprini |
| 2011 | 004SS04XXX | 07/08/2011 | SS | ASL 1 SASSARI | ovicaprini |
| 2011 | 071SS12XXX | 09/08/2011 | SS | ASL 1 SASSARI | ovicaprini |
| 2011 | 055SS05XXX | 11/08/2011 | SS | ASL 1 SASSARI | ovicaprini |
| 2012 | IT020CAXXX | 09/01/2012 | CA | ASL 8 CAGLIARI | ovicaprini |
| 2012 | IT032CAXXX | 09/01/2012 | CA | ASL 8 CAGLIARI | ovicaprini |
| 2012 | IT032CAXXX | 11/01/2012 | CA | ASL 8 CAGLIARI | ovicaprini |
| 2012 | IT057CAXXX | 12/01/2012 | CA | ASL 8 CAGLIARI | ovicaprini |
| 2012 | IT033SSXXX | 13/01/2012 | SS | ASL 1 SASSARI | ovicaprini |
| 2012 | IT033SSXXX | 13/01/2012 | SS | ASL 1 SASSARI | ovicaprini |
| 2012 | IT033SSXXX | 13/01/2012 | SS | ASL 1 SASSARI | ovicaprini |
| 2012 | IT033SSXXX | 13/01/2012 | SS | ASL 1 SASSARI | ovicaprini |
| 2012 | IT078CAXXX | 16/01/2012 | CA | ASL 8 CAGLIARI | ovicaprini |
| 2012 | IT092CAXXX | 16/01/2012 | CA | ASL 8 CAGLIARI | ovicaprini |
| 2012 | IT001CAXXX | 16/01/2012 | CA | ASL 8 CAGLIARI | ovicaprini |
| 2012 | IT029CAXXX | 16/01/2012 | CA | ASL 8 CAGLIARI | ovicaprini |
| 2012 | IT032CAXXX | 17/01/2012 | CA | ASL 8 CAGLIARI | ovicaprini |
| 2012 | IT029CAXXX | 17/01/2012 | CA | ASL 8 CAGLIARI | ovicaprini |
| 2012 | IT078CAXXX | 18/01/2012 | CA | ASL 8 CAGLIARI | ovicaprini |
| 2012 | IT033CAXXX | 18/01/2012 | CA | ASL 8 CAGLIARI | ovicaprini |
| 2012 | IT052CAXXX | 18/01/2012 | CA | ASL 8 CAGLIARI | ovicaprini |
| 2012 | IT078CAXXX | 19/01/2012 | CA | ASL 8 CAGLIARI | ovicaprini |
| 2012 | IT065CAXXX | 23/01/2012 | CA | ASL 8 CAGLIARI | ovicaprini |
| 2012 | IT043ORXXX | 23/01/2012 | OR | ASL ORISTANO | ovicaprini |
| 2012 | IT001CAXXX | 23/01/2012 | CA | ASL 8 CAGLIARI | ovicaprini |
| 2012 | IT092CAXXX | 23/01/2012 | CA | ASL 8 CAGLIARI | ovicaprini |
| 2012 | IT032CAXXX | 23/01/2012 | CA | ASL 8 CAGLIARI | ovicaprini |
| 2012 | IT093CAXXX | 30/01/2012 | CA | ASL 8 CAGLIARI | ovicaprini |
| 2012 | IT032CAXXX | 30/01/2012 | CA | ASL 8 CAGLIARI | ovicaprini |
| 2012 | IT055CAXXX | 30/01/2012 | CA | ASL 8 CAGLIARI | ovicaprini |
| 2012 | IT001CAXXX | 30/01/2012 | CA | ASL 8 CAGLIARI | ovicaprini |
| 2012 | IT057ORXXX | 31/01/2012 | OR | ASL ORISTANO | ovicaprini |
| 2012 | IT047ORXXX | 31/01/2012 | OR | ASL ORISTANO | ovicaprini |
| 2012 | IT024CAXXX | 01/02/2012 | CA | ASL 8 CAGLIARI | ovicaprini |
| 2012 | IT024CAXXX | 01/02/2012 | CA | ASL 8 CAGLIARI | ovicaprini |
| 2012 | IT041CAXXX | 03/02/2012 | CA | ASL 8 CAGLIARI | ovicaprini |
| 2012 | IT039ORXXX | 03/02/2012 | OR | ASL ORISTANO | ovicaprini |
| 2012 | IT039ORXXX | 03/02/2012 | OR | ASL ORISTANO | ovicaprini |
| 2012 | IT101CAXXX | 06/02/2012 | CA | ASL 8 CAGLIARI | ovicaprini |
| 2012 | IT032CAXXX | 06/02/2012 | CA | ASL 8 CAGLIARI | ovicaprini |
| 2012 | IT092CAXXX | 06/02/2012 | CA | ASL 8 CAGLIARI | ovicaprini |
| 2012 | IT029CAXXX | 07/02/2012 | CA | ASL 8 CAGLIARI | ovicaprini |
| 2012 | IT027ORXXX | 08/02/2012 | OR | ASL ORISTANO | ovicaprini |
| 2012 | IT032CAXXX | 08/02/2012 | CA | ASL 8 CAGLIARI | ovicaprini |
| 2012 | IT024CAXXX | 13/02/2012 | CA | ASL 8 CAGLIARI | ovicaprini |
| 2012 | IT065CAXXX | 13/02/2012 | CA | ASL 8 CAGLIARI | ovicaprini |
| 2012 | IT091CAXXX | 13/02/2012 | CA | ASL 8 CAGLIARI | ovicaprini |
| 2012 | IT031ORXXX | 13/02/2012 | OR | ASL ORISTANO | ovicaprini |
| 2012 | IT049ORXXX | 13/02/2012 | OR | ASL ORISTANO | ovicaprini |
| 2012 | IT092CAXXX | 13/02/2012 | CA | ASL 8 CAGLIARI | ovicaprini |
| 2012 | IT092CAXXX | 13/02/2012 | CA | ASL 8 CAGLIARI | ovicaprini |
| 2012 | IT092CAXXX | 13/02/2012 | CA | ASL 8 CAGLIARI | ovicaprini |
| 2012 | IT057CAXXX | 14/02/2012 | CA | ASL 8 CAGLIARI | ovicaprini |
| 2012 | IT052CAXXX | 14/02/2012 | CA | ASL 8 CAGLIARI | ovicaprini |
| 2012 | IT086CAXXX | 14/02/2012 | CA | ASL 8 CAGLIARI | ovicaprini |
| 2012 | IT065CAXXX | 15/02/2012 | CA | ASL 8 CAGLIARI | ovicaprini |
| 2012 | IT034NUXXX | 15/02/2012 | NU | ASL NUORO | ovicaprini |
| 2012 | IT080NUXXX | 15/02/2012 | NU | ASL NUORO | ovicaprini |
| 2012 | IT065CAXXX | 20/02/2012 | CA | ASL 8 CAGLIARI | ovicaprini |
| 2012 | IT065CAXXX | 20/02/2012 | CA | ASL 8 CAGLIARI | ovicaprini |
| 2012 | IT066CAXXX | 20/02/2012 | CA | ASL 8 CAGLIARI | ovicaprini |
| 2012 | IT055CAXXX | 20/02/2012 | CA | ASL 8 CAGLIARI | ovicaprini |
| 2012 | IT045CAXXX | 20/02/2012 | CA | ASL 8 CAGLIARI | ovicaprini |
| 2012 | IT029CAXXX | 20/02/2012 | CA | ASL 8 CAGLIARI | ovicaprini |
| 2012 | IT042ORXXX | 21/02/2012 | OR | ASL ORISTANO | ovicaprini |
| 2012 | IT011ORXXX | 21/02/2012 | OR | ASL ORISTANO | ovicaprini |
| 2012 | IT029ORXXX | 21/02/2012 | OR | ASL ORISTANO | ovicaprini |
| 2012 | IT011ORXXX | 21/02/2012 | OR | ASL ORISTANO | ovicaprini |
| 2012 | IT092CAXXX | 21/02/2012 | CA | ASL 8 CAGLIARI | ovicaprini |
| 2012 | IT080NUXXX | 24/02/2012 | NU | ASL NUORO | ovicaprini |
| 2012 | IT078CAXXX | 27/02/2012 | CA | ASL 8 CAGLIARI | ovicaprini |
| 2012 | IT029CAXXX | 27/02/2012 | CA | ASL 8 CAGLIARI | ovicaprini |
| 2012 | IT043ORXXX | 27/02/2012 | OR | ASL ORISTANO | ovicaprini |
| 2012 | IT043ORXXX | 27/02/2012 | OR | ASL ORISTANO | ovicaprini |
| 2012 | IT032CAXXX | 27/02/2012 | CA | ASL 8 CAGLIARI | ovicaprini |
| 2012 | IT029CAXXX | 27/02/2012 | CA | ASL 8 CAGLIARI | ovicaprini |
| 2012 | IT078CAXXX | 28/02/2012 | CA | ASL 8 CAGLIARI | ovicaprini |
| 2012 | IT057ORXXX | 28/02/2012 | OR | ASL ORISTANO | ovicaprini |
| 2012 | IT046CAXXX | 28/02/2012 | CA | ASL 8 CAGLIARI | ovicaprini |
| 2012 | IT092CAXXX | 28/02/2012 | CA | ASL 8 CAGLIARI | ovicaprini |
| 2012 | IT101CAXXX | 01/03/2012 | CA | ASL 8 CAGLIARI | ovicaprini |
| 2012 | IT072CAXXX | 02/03/2012 | CA | ASL 8 CAGLIARI | ovicaprini |
| 2012 | IT065CAXXX | 05/03/2012 | CA | ASL 8 CAGLIARI | ovicaprini |
| 2012 | IT031CAXXX | 05/03/2012 | CA | ASL 8 CAGLIARI | ovicaprini |
| 2012 | IT087CAXXX | 05/03/2012 | CA | ASL 8 CAGLIARI | ovicaprini |
| 2012 | IT094CAXXX | 05/03/2012 | CA | ASL 8 CAGLIARI | ovicaprini |
| 2012 | IT029ORXXX | 05/03/2012 | OR | ASL ORISTANO | ovicaprini |
| 2012 | IT029ORXXX | 05/03/2012 | OR | ASL ORISTANO | ovicaprini |
| 2012 | IT092CAXXX | 05/03/2012 | CA | ASL 8 CAGLIARI | ovicaprini |
| 2012 | IT045CAXXX | 05/03/2012 | CA | ASL 8 CAGLIARI | ovicaprini |
| 2012 | IT014CAXXX | 05/03/2012 | CA | ASL 8 CAGLIARI | ovicaprini |
| 2012 | IT091CAXXX | 06/03/2012 | CA | ASL 8 CAGLIARI | ovicaprini |
| 2012 | IT091CAXXX | 06/03/2012 | CA | ASL 8 CAGLIARI | ovicaprini |
| 2012 | IT032CAXXX | 06/03/2012 | CA | ASL 8 CAGLIARI | ovicaprini |
| 2012 | IT055CAXXX | 06/03/2012 | CA | ASL 8 CAGLIARI | ovicaprini |
| 2012 | IT045CAXXX | 06/03/2012 | CA | ASL 8 CAGLIARI | ovicaprini |
| 2012 | IT045CAXXX | 06/03/2012 | CA | ASL 8 CAGLIARI | ovicaprini |
| 2012 | IT055CAXXX | 06/03/2012 | CA | ASL 8 CAGLIARI | ovicaprini |
| 2012 | IT032CAXXX | 06/03/2012 | CA | ASL 8 CAGLIARI | ovicaprini |
| 2012 | IT057CAXXX | 08/03/2012 | CA | ASL 8 CAGLIARI | ovicaprini |
| 2012 | IT065CAXXX | 12/03/2012 | CA | ASL 8 CAGLIARI | ovicaprini |
| 2012 | IT094CAXXX | 12/03/2012 | CA | ASL 8 CAGLIARI | ovicaprini |
| 2012 | IT012ORXXX | 12/03/2012 | OR | ASL ORISTANO | ovicaprini |
| 2012 | IT045CAXXX | 12/03/2012 | CA | ASL 8 CAGLIARI | ovicaprini |
| 2012 | IT055CAXXX | 12/03/2012 | CA | ASL 8 CAGLIARI | ovicaprini |
| 2012 | IT101CAXXX | 12/03/2012 | CA | ASL 8 CAGLIARI | ovicaprini |
| 2012 | IT055CAXXX | 13/03/2012 | CA | ASL 8 CAGLIARI | ovicaprini |
| 2012 | IT039ORXXX | 14/03/2012 | OR | ASL ORISTANO | ovicaprini |
| 2012 | IT055CAXXX | 14/03/2012 | CA | ASL 8 CAGLIARI | ovicaprini |
| 2012 | IT091CAXXX | 15/03/2012 | CA | ASL 8 CAGLIARI | ovicaprini |
| 2012 | IT057CAXXX | 19/03/2012 | CA | ASL 8 CAGLIARI | ovicaprini |
| 2012 | IT101CAXXX | 19/03/2012 | CA | ASL 8 CAGLIARI | ovicaprini |
| 2012 | IT029ORXXX | 19/03/2012 | OR | ASL ORISTANO | ovicaprini |
| 2012 | IT012ORXXX | 19/03/2012 | OR | ASL ORISTANO | ovicaprini |
| 2012 | IT029ORXXX | 19/03/2012 | OR | ASL ORISTANO | ovicaprini |
| 2012 | IT092CAXXX | 19/03/2012 | CA | ASL 8 CAGLIARI | ovicaprini |
| 2012 | IT033CAXXX | 20/03/2012 | CA | ASL 8 CAGLIARI | ovicaprini |
| 2012 | IT057CAXXX | 20/03/2012 | CA | ASL 8 CAGLIARI | ovicaprini |
| 2012 | IT029ORXXX | 20/03/2012 | OR | ASL ORISTANO | ovicaprini |
| 2012 | IT040CAXXX | 21/03/2012 | CA | ASL 8 CAGLIARI | ovicaprini |
| 2012 | IT029CAXXX | 21/03/2012 | CA | ASL 8 CAGLIARI | ovicaprini |
| 2012 | IT032CAXXX | 21/03/2012 | CA | ASL 8 CAGLIARI | ovicaprini |
| 2012 | IT057CAXXX | 22/03/2012 | CA | ASL 8 CAGLIARI | ovicaprini |
| 2012 | IT057CAXXX | 23/03/2012 | CA | ASL 8 CAGLIARI | ovicaprini |
| 2012 | IT065CAXXX | 26/03/2012 | CA | ASL 8 CAGLIARI | ovicaprini |
| 2012 | IT057CAXXX | 26/03/2012 | CA | ASL 8 CAGLIARI | ovicaprini |
| 2012 | IT072CAXXX | 26/03/2012 | CA | ASL 8 CAGLIARI | ovicaprini |
| 2012 | IT055CAXXX | 26/03/2012 | CA | ASL 8 CAGLIARI | ovicaprini |
| 2012 | IT059SSXXX | 26/03/2012 | SS | ASL 1 SASSARI | ovicaprini |
| 2012 | IT029CAXXX | 26/03/2012 | CA | ASL 8 CAGLIARI | ovicaprini |
| 2012 | IT101CAXXX | 26/03/2012 | CA | ASL 8 CAGLIARI | ovicaprini |
| 2012 | IT045CAXXX | 26/03/2012 | CA | ASL 8 CAGLIARI | ovicaprini |
| 2012 | IT011ORXXX | 26/03/2012 | OR | ASL ORISTANO | ovicaprini |
| 2012 | IT039ORXXX | 27/03/2012 | OR | ASL ORISTANO | ovicaprini |
| 2012 | IT001CAXXX | 31/03/2012 | CA | ASL 8 CAGLIARI | ovicaprini |
| 2012 | IT065CAXXX | 02/04/2012 | CA | ASL 8 CAGLIARI | ovicaprini |
| 2012 | IT024CAXXX | 02/04/2012 | CA | ASL 8 CAGLIARI | ovicaprini |
| 2012 | IT057CAXXX | 02/04/2012 | CA | ASL 8 CAGLIARI | ovicaprini |
| 2012 | IT057CAXXX | 02/04/2012 | CA | ASL 8 CAGLIARI | ovicaprini |
| 2012 | IT094CAXXX | 11/04/2012 | CA | ASL 8 CAGLIARI | ovicaprini |
| 2012 | IT057CAXXX | 11/04/2012 | CA | ASL 8 CAGLIARI | ovicaprini |
| 2012 | IT024CAXXX | 16/04/2012 | CA | ASL 8 CAGLIARI | ovicaprini |
| 2012 | IT057CAXXX | 16/04/2012 | CA | ASL 8 CAGLIARI | ovicaprini |
| 2012 | IT031CAXXX | 16/04/2012 | CA | ASL 8 CAGLIARI | ovicaprini |
| 2012 | IT065CAXXX | 16/04/2012 | CA | ASL 8 CAGLIARI | ovicaprini |
| 2012 | IT051CAXXX | 18/04/2012 | CA | ASL 8 CAGLIARI | ovicaprini |
| 2012 | IT065CAXXX | 23/04/2012 | CA | ASL 8 CAGLIARI | ovicaprini |
| 2012 | IT051CAXXX | 23/04/2012 | CA | ASL 8 CAGLIARI | ovicaprini |
| 2012 | IT057CAXXX | 23/04/2012 | CA | ASL 8 CAGLIARI | ovicaprini |
| 2012 | IT031CAXXX | 23/04/2012 | CA | ASL 8 CAGLIARI | ovicaprini |
| 2012 | IT094CAXXX | 24/04/2012 | CA | ASL 8 CAGLIARI | ovicaprini |
| 2012 | IT051CAXXX | 24/04/2012 | CA | ASL 8 CAGLIARI | ovicaprini |
| 2012 | IT031CAXXX | 30/04/2012 | CA | ASL 8 CAGLIARI | ovicaprini |
| 2012 | IT065CAXXX | 30/04/2012 | CA | ASL 8 CAGLIARI | ovicaprini |
| 2012 | IT057CAXXX | 02/05/2012 | CA | ASL 8 CAGLIARI | ovicaprini |
| 2012 | IT057CAXXX | 02/05/2012 | CA | ASL 8 CAGLIARI | ovicaprini |
| 2012 | IT065CAXXX | 07/05/2012 | CA | ASL 8 CAGLIARI | ovicaprini |
| 2012 | IT031CAXXX | 07/05/2012 | CA | ASL 8 CAGLIARI | ovicaprini |
| 2012 | IT024CAXXX | 07/05/2012 | CA | ASL 8 CAGLIARI | ovicaprini |
| 2012 | IT057CAXXX | 07/05/2012 | CA | ASL 8 CAGLIARI | ovicaprini |
| 2012 | IT057CAXXX | 07/05/2012 | CA | ASL 8 CAGLIARI | ovicaprini |
| 2012 | IT057CAXXX | 08/05/2012 | CA | ASL 8 CAGLIARI | ovicaprini |
| 2012 | IT031CAXXX | 14/05/2012 | CA | ASL 8 CAGLIARI | ovicaprini |
| 2012 | IT065CAXXX | 14/05/2012 | CA | ASL 8 CAGLIARI | ovicaprini |
| 2012 | IT057CAXXX | 16/05/2012 | CA | ASL 8 CAGLIARI | ovicaprini |
| 2012 | IT024CAXXX | 16/05/2012 | CA | ASL 8 CAGLIARI | ovicaprini |
| 2012 | IT031CAXXX | 21/05/2012 | CA | ASL 8 CAGLIARI | ovicaprini |
| 2012 | IT065CAXXX | 21/05/2012 | CA | ASL 8 CAGLIARI | ovicaprini |
| 2012 | IT057CAXXX | 21/05/2012 | CA | ASL 8 CAGLIARI | ovicaprini |
| 2012 | IT024CAXXX | 23/05/2012 | CA | ASL 8 CAGLIARI | ovicaprini |
| 2012 | IT057CAXXX | 23/05/2012 | CA | ASL 8 CAGLIARI | ovicaprini |
| 2012 | IT057CAXXX | 28/05/2012 | CA | ASL 8 CAGLIARI | ovicaprini |
| 2012 | IT031CAXXX | 28/05/2012 | CA | ASL 8 CAGLIARI | ovicaprini |
| 2012 | IT065CAXXX | 28/05/2012 | CA | ASL 8 CAGLIARI | ovicaprini |
| 2012 | IT057CAXXX | 30/05/2012 | CA | ASL 8 CAGLIARI | ovicaprini |
| 2012 | IT065CAXXX | 04/06/2012 | CA | ASL 8 CAGLIARI | ovicaprini |
| 2012 | IT031CAXXX | 04/06/2012 | CA | ASL 8 CAGLIARI | ovicaprini |
| 2012 | IT057CAXXX | 04/06/2012 | CA | ASL 8 CAGLIARI | ovicaprini |
| 2012 | IT057CAXXX | 06/06/2012 | CA | ASL 8 CAGLIARI | ovicaprini |
| 2012 | IT065CAXXX | 11/06/2012 | CA | ASL 8 CAGLIARI | ovicaprini |
| 2012 | IT031CAXXX | 11/06/2012 | CA | ASL 8 CAGLIARI | ovicaprini |
| 2012 | IT057CAXXX | 13/06/2012 | CA | ASL 8 CAGLIARI | ovicaprini |
| 2012 | IT065CAXXX | 18/06/2012 | CA | ASL 8 CAGLIARI | ovicaprini |
| 2012 | IT094CAXXX | 18/06/2012 | CA | ASL 8 CAGLIARI | ovicaprini |
| 2012 | IT025CAXXX | 20/06/2012 | CA | ASL 8 CAGLIARI | ovicaprini |
| 2012 | IT006CAXXX | 20/06/2012 | CA | ASL 8 CAGLIARI | ovicaprini |
| 2012 | IT094CAXXX | 20/06/2012 | CA | ASL 8 CAGLIARI | ovicaprini |
| 2012 | IT094CAXXX | 20/06/2012 | CA | ASL 8 CAGLIARI | ovicaprini |
| 2012 | IT065CAXXX | 25/06/2012 | CA | ASL 8 CAGLIARI | ovicaprini |
| 2012 | IT001CAXXX | 25/06/2012 | CA | ASL 8 CAGLIARI | ovicaprini |
| 2012 | IT057CAXXX | 25/06/2012 | CA | ASL 8 CAGLIARI | ovicaprini |
| 2012 | IT094CAXXX | 25/06/2012 | CA | ASL 8 CAGLIARI | ovicaprini |
| 2012 | IT094CAXXX | 27/06/2012 | CA | ASL 8 CAGLIARI | ovicaprini |
| 2012 | IT091CAXXX | 02/04/2012 | CA | ASL 8 CAGLIARI | ovicaprini |
| 2012 | IT012ORXXX | 04/04/2012 | OR | ASL ORISTANO | ovicaprini |
| 2012 | IT072CAXXX | 05/04/2012 | CA | ASL 8 CAGLIARI | ovicaprini |
| 2012 | IT072CAXXX | 10/04/2012 | CA | ASL 8 CAGLIARI | ovicaprini |
| 2012 | IT032CAXXX | 10/04/2012 | CA | ASL 8 CAGLIARI | ovicaprini |
| 2012 | IT032CAXXX | 10/04/2012 | CA | ASL 8 CAGLIARI | ovicaprini |
| 2012 | IT101CAXXX | 12/04/2012 | CA | ASL 8 CAGLIARI | ovicaprini |
| 2012 | IT032CAXXX | 12/04/2012 | CA | ASL 8 CAGLIARI | ovicaprini |
| 2012 | IT032CAXXX | 12/04/2012 | CA | ASL 8 CAGLIARI | ovicaprini |
| 2012 | IT092CAXXX | 13/04/2012 | CA | ASL 8 CAGLIARI | ovicaprini |
| 2012 | IT092CAXXX | 13/04/2012 | CA | ASL 8 CAGLIARI | ovicaprini |
| 2012 | IT072CAXXX | 13/04/2012 | CA | ASL 8 CAGLIARI | ovicaprini |
| 2012 | IT072CAXXX | 13/04/2012 | CA | ASL 8 CAGLIARI | ovicaprini |
| 2012 | IT072CAXXX | 16/04/2012 | CA | ASL 8 CAGLIARI | ovicaprini |
| 2012 | IT072CAXXX | 16/04/2012 | CA | ASL 8 CAGLIARI | ovicaprini |
| 2012 | IT072CAXXX | 17/04/2012 | CA | ASL 8 CAGLIARI | ovicaprini |
| 2012 | IT057CAXXX | 17/04/2012 | CA | ASL 8 CAGLIARI | ovicaprini |
| 2012 | IT032CAXXX | 17/04/2012 | CA | ASL 8 CAGLIARI | ovicaprini |
| 2012 | IT032CAXXX | 17/04/2012 | CA | ASL 8 CAGLIARI | ovicaprini |
| 2012 | IT032CAXXX | 17/04/2012 | CA | ASL 8 CAGLIARI | ovicaprini |
| 2012 | IT057CAXXX | 17/04/2012 | CA | ASL 8 CAGLIARI | ovicaprini |
| 2012 | IT032CAXXX | 17/04/2012 | CA | ASL 8 CAGLIARI | ovicaprini |
| 2012 | IT019CAXXX | 19/04/2012 | CA | ASL 8 CAGLIARI | ovicaprini |
| 2012 | IT019CAXXX | 20/04/2012 | CA | ASL 8 CAGLIARI | ovicaprini |
| 2012 | IT033CAXXX | 20/04/2012 | CA | ASL 8 CAGLIARI | ovicaprini |
| 2012 | IT072CAXXX | 23/04/2012 | CA | ASL 8 CAGLIARI | ovicaprini |
| 2012 | IT025CAXXX | 23/04/2012 | CA | ASL 8 CAGLIARI | ovicaprini |
| 2012 | IT057CAXXX | 23/04/2012 | CA | ASL 8 CAGLIARI | ovicaprini |
| 2012 | IT040CAXXX | 24/04/2012 | CA | ASL 8 CAGLIARI | ovicaprini |
| 2012 | IT040CAXXX | 24/04/2012 | CA | ASL 8 CAGLIARI | ovicaprini |
| 2012 | IT078CAXXX | 26/04/2012 | CA | ASL 8 CAGLIARI | ovicaprini |
| 2012 | IT001CAXXX | 26/04/2012 | CA | ASL 8 CAGLIARI | ovicaprini |
| 2012 | IT0078CXXX | 27/04/2012 | CA | ASL 8 CAGLIARI | ovicaprini |
| 2012 | IT029CAXXX | 27/04/2012 | CA | ASL 8 CAGLIARI | ovicaprini |
| 2012 | IT055CAXXX | 30/04/2012 | CA | ASL 8 CAGLIARI | ovicaprini |
| 2012 | IT078CAXXX | 30/04/2012 | CA | ASL 8 CAGLIARI | ovicaprini |
| 2012 | IT020CAXXX | 30/04/2012 | CA | ASL 8 CAGLIARI | ovicaprini |
| 2012 | IT052CAXXX | 03/05/2012 | CA | ASL 8 CAGLIARI | ovicaprini |
| 2012 | IT020CAXXX | 03/05/2012 | CA | ASL 8 CAGLIARI | ovicaprini |
| 2012 | IT072CAXXX | 03/05/2012 | CA | ASL 8 CAGLIARI | ovicaprini |
| 2012 | IT020CAXXX | 03/05/2012 | CA | ASL 8 CAGLIARI | ovicaprini |
| 2012 | IT096CAXXX | 07/05/2012 | CA | ASL 8 CAGLIARI | ovicaprini |
| 2012 | IT016CAXXX | 07/05/2012 | CA | ASL 8 CAGLIARI | ovicaprini |
| 2012 | IT016CAXXX | 08/05/2012 | CA | ASL 8 CAGLIARI | ovicaprini |
| 2012 | IT057CAXXX | 08/05/2012 | CA | ASL 8 CAGLIARI | ovicaprini |
| 2012 | IT032CAXXX | 08/05/2012 | CA | ASL 8 CAGLIARI | ovicaprini |
| 2012 | IT092CAXXX | 10/05/2012 | CA | ASL 8 CAGLIARI | ovicaprini |
| 2012 | IT001CAXXX | 10/05/2012 | CA | ASL 8 CAGLIARI | ovicaprini |
| 2012 | IT016CAXXX | 10/05/2012 | CA | ASL 8 CAGLIARI | ovicaprini |
| 2012 | IT101CAXXX | 10/05/2012 | CA | ASL 8 CAGLIARI | ovicaprini |
| 2012 | IT040CAXXX | 10/05/2012 | CA | ASL 8 CAGLIARI | ovicaprini |
| 2012 | IT092CAXXX | 11/05/2012 | CA | ASL 8 CAGLIARI | ovicaprini |
| 2012 | IT016CAXXX | 11/05/2012 | CA | ASL 8 CAGLIARI | ovicaprini |
| 2012 | IT016CAXXX | 14/05/2012 | CA | ASL 8 CAGLIARI | ovicaprini |
| 2012 | IT032CAXXX | 14/05/2012 | CA | ASL 8 CAGLIARI | ovicaprini |
| 2012 | IT057CAXXX | 14/05/2012 | CA | ASL 8 CAGLIARI | ovicaprini |
| 2012 | IT072CAXXX | 14/05/2012 | CA | ASL 8 CAGLIARI | ovicaprini |
| 2012 | IT012ORXXX | 18/05/2012 | OR | ASL ORISTANO | ovicaprini |
| 2012 | IT092CAXXX | 18/05/2012 | CA | ASL 8 CAGLIARI | ovicaprini |
| 2012 | IT012ORXXX | 21/05/2012 | OR | ASL ORISTANO | ovicaprini |
| 2012 | IT072CAXXX | 21/05/2012 | CA | ASL 8 CAGLIARI | ovicaprini |
| 2012 | IT032CAXXX | 21/05/2012 | CA | ASL 8 CAGLIARI | ovicaprini |
| 2012 | IT032CAXXX | 22/05/2012 | CA | ASL 8 CAGLIARI | ovicaprini |
| 2012 | IT092CAXXX | 22/05/2012 | CA | ASL 8 CAGLIARI | ovicaprini |
| 2012 | IT072CAXXX | 22/05/2012 | CA | ASL 8 CAGLIARI | ovicaprini |
| 2012 | IT092CAXXX | 24/05/2012 | CA | ASL 8 CAGLIARI | ovicaprini |
| 2012 | IT091CAXXX | 24/05/2012 | CA | ASL 8 CAGLIARI | ovicaprini |
| 2012 | IT092CAXXX | 24/05/2012 | CA | ASL 8 CAGLIARI | ovicaprini |
| 2012 | IT092CAXXX | 24/05/2012 | CA | ASL 8 CAGLIARI | ovicaprini |
| 2012 | IT096CAXXX | 25/05/2012 | CA | ASL 8 CAGLIARI | ovicaprini |
| 2012 | IT092CAXXX | 25/05/2012 | CA | ASL 8 CAGLIARI | ovicaprini |
| 2012 | IT091CAXXX | 25/05/2012 | CA | ASL 8 CAGLIARI | ovicaprini |
| 2012 | IT096CAXXX | 28/05/2012 | CA | ASL 8 CAGLIARI | ovicaprini |
| 2012 | IT091CAXXX | 28/05/2012 | CA | ASL 8 CAGLIARI | ovicaprini |
| 2012 | IT032CAXXX | 29/05/2012 | CA | ASL 8 CAGLIARI | ovicaprini |
| 2012 | IT032CAXXX | 29/05/2012 | CA | ASL 8 CAGLIARI | ovicaprini |
| 2012 | IT036NUXXX | 31/05/2012 | NU | ASL NUORO | ovicaprini |
| 2012 | IT092CAXXX | 31/05/2012 | CA | ASL 8 CAGLIARI | ovicaprini |
| 2012 | IT057CAXXX | 31/05/2012 | CA | ASL 8 CAGLIARI | ovicaprini |
| 2012 | IT078CAXXX | 31/05/2012 | CA | ASL 8 CAGLIARI | ovicaprini |
| 2012 | IT078CAXXX | 31/05/2012 | CA | ASL 8 CAGLIARI | ovicaprini |
| 2012 | IT096CAXXX | 04/06/2012 | CA | ASL 8 CAGLIARI | ovicaprini |
| 2012 | IT020CAXXX | 04/06/2012 | CA | ASL 8 CAGLIARI | ovicaprini |
| 2012 | IT091CAXXX | 04/06/2012 | CA | ASL 8 CAGLIARI | ovicaprini |
| 2012 | IT040CAXXX | 05/06/2012 | CA | ASL 8 CAGLIARI | ovicaprini |
| 2012 | IT092CAXXX | 05/06/2012 | CA | ASL 8 CAGLIARI | ovicaprini |
| 2012 | IT093CAXXX | 05/06/2012 | CA | ASL 8 CAGLIARI | ovicaprini |
| 2012 | IT016CAXXX | 07/06/2012 | CA | ASL 8 CAGLIARI | ovicaprini |
| 2012 | IT092CAXXX | 07/06/2012 | CA | ASL 8 CAGLIARI | ovicaprini |
| 2012 | IT093CAXXX | 07/06/2012 | CA | ASL 8 CAGLIARI | ovicaprini |
| 2012 | IT016CAXXX | 08/06/2012 | CA | ASL 8 CAGLIARI | ovicaprini |
| 2012 | IT016CAXXX | 11/06/2012 | CA | ASL 8 CAGLIARI | ovicaprini |
| 2012 | IT016CAXXX | 11/06/2012 | CA | ASL 8 CAGLIARI | ovicaprini |
| 2012 | IT057CAXXX | 11/06/2012 | CA | ASL 8 CAGLIARI | ovicaprini |
| 2012 | IT016CAXXX | 11/06/2012 | CA | ASL 8 CAGLIARI | ovicaprini |
| 2012 | IT091CAXXX | 11/06/2012 | CA | ASL 8 CAGLIARI | ovicaprini |
| 2012 | IT091CAXXX | 11/06/2012 | CA | ASL 8 CAGLIARI | ovicaprini |
| 2012 | IT019CAXXX | 12/06/2012 | CA | ASL 8 CAGLIARI | ovicaprini |
| 2012 | IT040CAXXX | 12/06/2012 | CA | ASL 8 CAGLIARI | ovicaprini |
| 2012 | IT036NUXXX | 14/06/2012 | NU | ASL NUORO | ovicaprini |
| 2012 | IT092CAXXX | 14/06/2012 | CA | ASL 8 CAGLIARI | ovicaprini |
| 2012 | IT033CAXXX | 14/06/2012 | CA | ASL 8 CAGLIARI | ovicaprini |
| 2012 | IT033CAXXX | 15/06/2012 | CA | ASL 8 CAGLIARI | ovicaprini |
| 2012 | IT057CAXXX | 18/06/2012 | CA | ASL 8 CAGLIARI | ovicaprini |
| 2012 | IT057CAXXX | 18/06/2012 | CA | ASL 8 CAGLIARI | ovicaprini |
| 2012 | IT025CAXXX | 18/06/2012 | CA | ASL 8 CAGLIARI | ovicaprini |
| 2012 | IT033CAXXX | 18/06/2012 | CA | ASL 8 CAGLIARI | ovicaprini |
| 2012 | IT052CAXXX | 18/06/2012 | CA | ASL 8 CAGLIARI | ovicaprini |
| 2012 | IT092CAXXX | 19/06/2012 | CA | ASL 8 CAGLIARI | ovicaprini |
| 2012 | IT092CAXXX | 21/06/2012 | CA | ASL 8 CAGLIARI | ovicaprini |
| 2012 | IT094CAXXX | 21/06/2012 | CA | ASL 8 CAGLIARI | ovicaprini |
| 2012 | IT092CAXXX | 21/06/2012 | CA | ASL 8 CAGLIARI | ovicaprini |
| 2012 | IT016CAXXX | 25/06/2012 | CA | ASL 8 CAGLIARI | ovicaprini |
| 2012 | IT040CAXXX | 26/06/2012 | CA | ASL 8 CAGLIARI | ovicaprini |
| 2012 | IT033CAXXX | 26/06/2012 | CA | ASL 8 CAGLIARI | ovicaprini |
| 2012 | IT019CAXXX | 26/06/2012 | CA | ASL 8 CAGLIARI | ovicaprini |
| 2012 | IT019CAXXX | 26/06/2012 | CA | ASL 8 CAGLIARI | ovicaprini |
| 2012 | IT072CAXXX | 26/06/2012 | CA | ASL 8 CAGLIARI | ovicaprini |
| 2012 | IT091CAXXX | 28/06/2012 | CA | ASL 8 CAGLIARI | ovicaprini |
| 2012 | IT091CAXXX | 28/06/2012 | CA | ASL 8 CAGLIARI | ovicaprini |
| 2012 | IT052CAXXX | 29/06/2012 | CA | ASL 8 CAGLIARI | ovicaprini |
| 2012 | IT091CAXXX | 29/06/2012 | CA | ASL 8 CAGLIARI | ovicaprini |
| 2012 | IT092CAXXX | 29/06/2012 | CA | ASL 8 CAGLIARI | ovicaprini |
| 2012 | IT101CAXXX | 02/04/2012 | CA | ASL 8 CAGLIARI | ovicaprini |
| 2012 | IT057ORXXX | 02/04/2012 | OR | ASL ORISTANO | ovicaprini |
| 2012 | IT045CAXXX | 02/04/2012 | CA | ASL 8 CAGLIARI | ovicaprini |
| 2012 | IT048ORXXX | 10/04/2012 | OR | ASL ORISTANO | ovicaprini |
| 2012 | IT045CAXXX | 10/04/2012 | CA | ASL 8 CAGLIARI | ovicaprini |
| 2012 | IT044ORXXX | 11/04/2012 | OR | ASL ORISTANO | ovicaprini |
| 2012 | IT032CAXXX | 16/04/2012 | CA | ASL 8 CAGLIARI | ovicaprini |
| 2012 | IT029CAXXX | 16/04/2012 | CA | ASL 8 CAGLIARI | ovicaprini |
| 2012 | IT028ORXXX | 16/04/2012 | OR | ASL ORISTANO | ovicaprini |
| 2012 | IT101CAXXX | 16/04/2012 | CA | ASL 8 CAGLIARI | ovicaprini |
| 2012 | IT023CAXXX | 16/04/2012 | CA | ASL 8 CAGLIARI | ovicaprini |
| 2012 | IT023CAXXX | 16/04/2012 | CA | ASL 8 CAGLIARI | ovicaprini |
| 2012 | IT045CAXXX | 16/04/2012 | CA | ASL 8 CAGLIARI | ovicaprini |
| 2012 | IT044ORXXX | 17/04/2012 | OR | ASL ORISTANO | ovicaprini |
| 2012 | IT047ORXXX | 17/04/2012 | OR | ASL ORISTANO | ovicaprini |
| 2012 | IT006CAXXX | 18/04/2012 | CA | ASL 8 CAGLIARI | ovicaprini |
| 2012 | IT006CAXXX | 18/04/2012 | CA | ASL 8 CAGLIARI | ovicaprini |
| 2012 | IT006CAXXX | 18/04/2012 | CA | ASL 8 CAGLIARI | ovicaprini |
| 2012 | IT059ORXXX | 18/04/2012 | OR | ASL ORISTANO | ovicaprini |
| 2012 | IT086CAXXX | 20/04/2012 | CA | ASL 8 CAGLIARI | ovicaprini |
| 2012 | IT086CAXXX | 20/04/2012 | CA | ASL 8 CAGLIARI | ovicaprini |
| 2012 | IT069ORXXX | 23/04/2012 | OR | ASL ORISTANO | ovicaprini |
| 2012 | IT101CAXXX | 23/04/2012 | CA | ASL 8 CAGLIARI | ovicaprini |
| 2012 | IT032CAXXX | 23/04/2012 | CA | ASL 8 CAGLIARI | ovicaprini |
| 2012 | IT078CAXXX | 23/04/2012 | CA | ASL 8 CAGLIARI | ovicaprini |
| 2012 | IT078CAXXX | 23/04/2012 | CA | ASL 8 CAGLIARI | ovicaprini |
| 2012 | IT045CAXXX | 23/04/2012 | CA | ASL 8 CAGLIARI | ovicaprini |
| 2012 | IT046CAXXX | 24/04/2012 | CA | ASL 8 CAGLIARI | ovicaprini |
| 2012 | IT065CAXXX | 26/04/2012 | CA | ASL 8 CAGLIARI | ovicaprini |
| 2012 | IT012ORXXX | 30/04/2012 | OR | ASL ORISTANO | ovicaprini |
| 2012 | IT029CAXXX | 30/04/2012 | CA | ASL 8 CAGLIARI | ovicaprini |
| 2012 | IT029CAXXX | 30/04/2012 | CA | ASL 8 CAGLIARI | ovicaprini |
| 2012 | IT086CAXXX | 30/04/2012 | CA | ASL 8 CAGLIARI | ovicaprini |
| 2012 | IT055CAXXX | 07/05/2012 | CA | ASL 8 CAGLIARI | ovicaprini |
| 2012 | IT055CAXXX | 07/05/2012 | CA | ASL 8 CAGLIARI | ovicaprini |
| 2012 | IT092CAXXX | 07/05/2012 | CA | ASL 8 CAGLIARI | ovicaprini |
| 2012 | IT055CAXXX | 07/05/2012 | CA | ASL 8 CAGLIARI | ovicaprini |
| 2012 | IT012ORXXX | 07/05/2012 | OR | ASL ORISTANO | ovicaprini |
| 2012 | IT032CAXXX | 07/05/2012 | CA | ASL 8 CAGLIARI | ovicaprini |
| 2012 | IT045CAXXX | 07/05/2012 | CA | ASL 8 CAGLIARI | ovicaprini |
| 2012 | IT029ORXXX | 07/05/2012 | OR | ASL ORISTANO | ovicaprini |
| 2012 | IT092CAXXX | 08/05/2012 | CA | ASL 8 CAGLIARI | ovicaprini |
| 2012 | IT044ORXXX | 08/05/2012 | OR | ASL ORISTANO | ovicaprini |
| 2012 | IT032CAXXX | 09/05/2012 | CA | ASL 8 CAGLIARI | ovicaprini |
| 2012 | IT032CAXXX | 11/05/2012 | CA | ASL 8 CAGLIARI | ovicaprini |
| 2012 | IT032CAXXX | 11/05/2012 | CA | ASL 8 CAGLIARI | ovicaprini |
| 2012 | IT045CAXXX | 14/05/2012 | CA | ASL 8 CAGLIARI | ovicaprini |
| 2012 | IT032CAXXX | 14/05/2012 | CA | ASL 8 CAGLIARI | ovicaprini |
| 2012 | IT055CAXXX | 14/05/2012 | CA | ASL 8 CAGLIARI | ovicaprini |
| 2012 | IT046CAXXX | 14/05/2012 | CA | ASL 8 CAGLIARI | ovicaprini |
| 2012 | IT032CAXXX | 14/05/2012 | CA | ASL 8 CAGLIARI | ovicaprini |
| 2012 | IT045CAXXX | 14/05/2012 | CA | ASL 8 CAGLIARI | ovicaprini |
| 2012 | IT045CAXXX | 14/05/2012 | CA | ASL 8 CAGLIARI | ovicaprini |
| 2012 | IT044CAXXX | 15/05/2012 | CA | ASL 8 CAGLIARI | ovicaprini |
| 2012 | IT065CAXXX | 18/05/2012 | CA | ASL 8 CAGLIARI | ovicaprini |
| 2012 | IT006CAXXX | 18/05/2012 | CA | ASL 8 CAGLIARI | ovicaprini |
| 2012 | IT006CAXXX | 18/05/2012 | CA | ASL 8 CAGLIARI | ovicaprini |
| 2012 | IT101CAXXX | 18/05/2012 | CA | ASL 8 CAGLIARI | ovicaprini |
| 2012 | IT029ORXXX | 21/05/2012 | OR | ASL ORISTANO | ovicaprini |
| 2012 | IT077ORXXX | 21/05/2012 | OR | ASL ORISTANO | ovicaprini |
| 2012 | IT029ORXXX | 21/05/2012 | OR | ASL ORISTANO | ovicaprini |
| 2012 | IT032CAXXX | 21/05/2012 | CA | ASL 8 CAGLIARI | ovicaprini |
| 2012 | IT092CAXXX | 21/05/2012 | CA | ASL 8 CAGLIARI | ovicaprini |
| 2012 | IT045CAXXX | 21/05/2012 | CA | ASL 8 CAGLIARI | ovicaprini |
| 2012 | IT094CAXXX | 21/05/2012 | CA | ASL 8 CAGLIARI | ovicaprini |
| 2012 | IT029ORXXX | 21/05/2012 | OR | ASL ORISTANO | ovicaprini |
| 2012 | IT029ORXXX | 21/05/2012 | OR | ASL ORISTANO | ovicaprini |
| 2012 | IT012CAXXX | 21/05/2012 | CA | ASL 8 CAGLIARI | ovicaprini |
| 2012 | IT094CAXXX | 21/05/2012 | CA | ASL 8 CAGLIARI | ovicaprini |
| 2012 | IT055CAXXX | 22/05/2012 | CA | ASL 8 CAGLIARI | ovicaprini |
| 2012 | IT006CAXXX | 23/05/2012 | CA | ASL 8 CAGLIARI | ovicaprini |
| 2012 | IT078CAXXX | 23/05/2012 | CA | ASL 8 CAGLIARI | ovicaprini |
| 2012 | IT044ORXXX | 28/05/2012 | OR | ASL ORISTANO | ovicaprini |
| 2012 | IT045CAXXX | 28/05/2012 | CA | ASL 8 CAGLIARI | ovicaprini |
| 2012 | IT042ORXXX | 28/05/2012 | OR | ASL ORISTANO | ovicaprini |
| 2012 | IT092CAXXX | 28/05/2012 | CA | ASL 8 CAGLIARI | ovicaprini |
| 2012 | IT026ORXXX | 28/05/2012 | OR | ASL ORISTANO | ovicaprini |
| 2012 | IT028ORXXX | 28/05/2012 | OR | ASL ORISTANO | ovicaprini |
| 2012 | IT039ORXXX | 29/05/2012 | OR | ASL ORISTANO | ovicaprini |
| 2012 | IT065CAXXX | 30/05/2012 | CA | ASL 8 CAGLIARI | ovicaprini |
| 2012 | IT065CAXXX | 30/05/2012 | CA | ASL 8 CAGLIARI | ovicaprini |
| 2012 | IT032CAXXX | 30/05/2012 | CA | ASL 8 CAGLIARI | ovicaprini |
| 2012 | IT092CAXXX | 04/06/2012 | CA | ASL 8 CAGLIARI | ovicaprini |
| 2012 | IT043ORXXX | 04/06/2012 | OR | ASL ORISTANO | ovicaprini |
| 2012 | IT029CAXXX | 04/06/2012 | CA | ASL 8 CAGLIARI | ovicaprini |
| 2012 | IT045CAXXX | 04/06/2012 | CA | ASL 8 CAGLIARI | ovicaprini |
| 2012 | IT065CAXXX | 04/06/2012 | CA | ASL 8 CAGLIARI | ovicaprini |
| 2012 | IT039ORXXX | 05/06/2012 | OR | ASL ORISTANO | ovicaprini |
| 2012 | IT046ORXXX | 05/06/2012 | OR | ASL ORISTANO | ovicaprini |
| 2012 | IT055CAXXX | 11/06/2012 | CA | ASL 8 CAGLIARI | ovicaprini |
| 2012 | IT086CAXXX | 11/06/2012 | CA | ASL 8 CAGLIARI | ovicaprini |
| 2012 | IT091CAXXX | 11/06/2012 | CA | ASL 8 CAGLIARI | ovicaprini |
| 2012 | IT043ORXXX | 11/06/2012 | OR | ASL ORISTANO | ovicaprini |
| 2012 | IT045CAXXX | 11/06/2012 | CA | ASL 8 CAGLIARI | ovicaprini |
| 2012 | IT086CAXXX | 11/06/2012 | CA | ASL 8 CAGLIARI | ovicaprini |
| 2012 | IT043ORXXX | 11/06/2012 | OR | ASL ORISTANO | ovicaprini |
| 2012 | IT004ORXXX | 12/06/2012 | OR | ASL ORISTANO | ovicaprini |
| 2012 | IT045CAXXX | 18/06/2012 | CA | ASL 8 CAGLIARI | ovicaprini |
| 2012 | IT006CAXXX | 18/06/2012 | CA | ASL 8 CAGLIARI | ovicaprini |
| 2012 | IT012ORXXX | 18/06/2012 | OR | ASL ORISTANO | ovicaprini |
| 2012 | IT086CAXXX | 18/06/2012 | CA | ASL 8 CAGLIARI | ovicaprini |
| 2012 | IT022CAXXX | 19/06/2012 | CA | ASL 8 CAGLIARI | ovicaprini |
| 2012 | IT004ORXXX | 19/06/2012 | OR | ASL ORISTANO | ovicaprini |
| 2012 | IT055CAXXX | 20/06/2012 | CA | ASL 8 CAGLIARI | ovicaprini |
| 2012 | IT055CAXXX | 20/06/2012 | CA | ASL 8 CAGLIARI | ovicaprini |
| 2012 | IT046CAXXX | 25/06/2012 | CA | ASL 8 CAGLIARI | ovicaprini |
| 2012 | IT046CAXXX | 25/06/2012 | CA | ASL 8 CAGLIARI | ovicaprini |
| 2012 | IT029CAXXX | 25/06/2012 | CA | ASL 8 CAGLIARI | ovicaprini |
| 2012 | IT078CAXXX | 25/06/2012 | CA | ASL 8 CAGLIARI | ovicaprini |
| 2012 | IT012ORXXX | 25/06/2012 | OR | ASL ORISTANO | ovicaprini |
| 2012 | IT029CAXXX | 25/06/2012 | CA | ASL 8 CAGLIARI | ovicaprini |
| 2012 | IT012ORXXX | 25/06/2012 | OR | ASL ORISTANO | ovicaprini |
| 2012 | IT057CAXXX | 25/06/2012 | CA | ASL 8 CAGLIARI | ovicaprini |
| 2012 | IT004ORXXX | 26/06/2012 | OR | ASL ORISTANO | ovicaprini |
| 2012 | IT033CAXXX | 13/04/2012 | CA | ASL 8 CAGLIARI | ovicaprini |
| 2012 | IT029CAXXX | 23/04/2012 | CA | ASL 8 CAGLIARI | ovicaprini |
| 2012 | IT092CAXXX | 27/04/2012 | CA | ASL 8 CAGLIARI | ovicaprini |
| 2012 | IT096CAXXX | 04/05/2012 | CA | ASL 8 CAGLIARI | ovicaprini |
| 2012 | IT029CAXXX | 07/05/2012 | CA | ASL 8 CAGLIARI | ovicaprini |
| 2012 | IT033CAXXX | 11/05/2012 | CA | ASL 8 CAGLIARI | ovicaprini |
| 2012 | IT096CAXXX | 11/05/2012 | CA | ASL 8 CAGLIARI | ovicaprini |
| 2012 | IT032CAXXX | 18/05/2012 | CA | ASL 8 CAGLIARI | ovicaprini |
| 2012 | IT032CAXXX | 18/05/2012 | CA | ASL 8 CAGLIARI | ovicaprini |
| 2012 | IT053CAXXX | 18/05/2012 | CA | ASL 8 CAGLIARI | ovicaprini |
| 2012 | IT031CAXXX | 25/05/2012 | CA | ASL 8 CAGLIARI | ovicaprini |
| 2012 | IT096CAXXX | 01/06/2012 | CA | ASL 8 CAGLIARI | ovicaprini |
| 2012 | IT060CAXXX | 01/06/2012 | CA | ASL 8 CAGLIARI | ovicaprini |
| 2012 | IT070CAXXX | 04/06/2012 | CA | ASL 8 CAGLIARI | ovicaprini |
| 2012 | IT060CAXXX | 08/06/2012 | CA | ASL 8 CAGLIARI | ovicaprini |
| 2012 | IT092CAXXX | 08/06/2012 | CA | ASL 8 CAGLIARI | ovicaprini |
| 2012 | IT060CAXXX | 15/06/2012 | CA | ASL 8 CAGLIARI | ovicaprini |
| 2012 | IT053CAXXX | 18/06/2012 | CA | ASL 8 CAGLIARI | ovicaprini |
| 2012 | IT032CAXXX | 22/06/2012 | CA | ASL 8 CAGLIARI | ovicaprini |
| 2012 | IT032CAXXX | 22/06/2012 | CA | ASL 8 CAGLIARI | ovicaprini |
| 2012 | IT070CAXXX | 25/06/2012 | CA | ASL 8 CAGLIARI | ovicaprini |
| 2012 | IT092CAXXX | 29/06/2012 | CA | ASL 8 CAGLIARI | ovicaprini |
| 2012 | IT069ORXXX | 02/07/2012 | OR | ASL ORISTANO | ovicaprini |
| 2012 | IT069ORXXX | 02/07/2012 | OR | ASL ORISTANO | ovicaprini |
| 2012 | IT001CAXXX | 02/07/2012 | CA | ASL 8 CAGLIARI | ovicaprini |
| 2012 | IT055CAXXX | 02/07/2012 | CA | ASL 8 CAGLIARI | ovicaprini |
| 2012 | IT042ORXXX | 02/07/2012 | OR | ASL ORISTANO | ovicaprini |
| 2012 | IT078CAXXX | 02/07/2012 | CA | ASL 8 CAGLIARI | ovicaprini |
| 2012 | IT029CAXXX | 09/07/2012 | CA | ASL 8 CAGLIARI | ovicaprini |
| 2012 | IT010ORXXX | 09/07/2012 | OR | ASL ORISTANO | ovicaprini |
| 2012 | IT078CAXXX | 09/07/2012 | CA | ASL 8 CAGLIARI | ovicaprini |
| 2012 | IT046ORXXX | 09/07/2012 | OR | ASL ORISTANO | ovicaprini |
| 2012 | IT069ORXXX | 09/07/2012 | OR | ASL ORISTANO | ovicaprini |
| 2012 | IT010ORXXX | 10/07/2012 | OR | ASL ORISTANO | ovicaprini |
| 2012 | IT032CAXXX | 11/07/2012 | CA | ASL 8 CAGLIARI | ovicaprini |
| 2012 | IT046ORXXX | 13/07/2012 | OR | ASL ORISTANO | ovicaprini |
| 2012 | IT069ORXXX | 16/07/2012 | OR | ASL ORISTANO | ovicaprini |
| 2012 | IT001CAXXX | 16/07/2012 | CA | ASL 8 CAGLIARI | ovicaprini |
| 2012 | IT078CAXXX | 16/07/2012 | CA | ASL 8 CAGLIARI | ovicaprini |
| 2012 | IT029CAXXX | 16/07/2012 | CA | ASL 8 CAGLIARI | ovicaprini |
| 2012 | IT034NUXXX | 16/07/2012 | NU | ASL NUORO | ovicaprini |
| 2012 | IT012ORXXX | 17/07/2012 | OR | ASL ORISTANO | ovicaprini |
| 2012 | IT032CAXXX | 23/07/2012 | CA | ASL 8 CAGLIARI | ovicaprini |
| 2012 | IT032CAXXX | 23/07/2012 | CA | ASL 8 CAGLIARI | ovicaprini |
| 2012 | IT077ORXXX | 23/07/2012 | OR | ASL ORISTANO | ovicaprini |
| 2012 | IT001CAXXX | 23/07/2012 | CA | ASL 8 CAGLIARI | ovicaprini |
| 2012 | IT029CAXXX | 23/07/2012 | CA | ASL 8 CAGLIARI | ovicaprini |
| 2012 | IT046ORXXX | 24/07/2012 | OR | ASL ORISTANO | ovicaprini |
| 2012 | IT006CAXXX | 30/07/2012 | CA | ASL 8 CAGLIARI | ovicaprini |
| 2012 | IT029CAXXX | 30/07/2012 | CA | ASL 8 CAGLIARI | ovicaprini |
| 2012 | IT023CAXXX | 30/07/2012 | CA | ASL 8 CAGLIARI | ovicaprini |
| 2012 | IT032CAXXX | 01/08/2012 | CA | ASL 8 CAGLIARI | ovicaprini |
| 2012 | IT032CAXXX | 01/08/2012 | CA | ASL 8 CAGLIARI | ovicaprini |
| 2012 | IT023CAXXX | 01/08/2012 | CA | ASL 8 CAGLIARI | ovicaprini |
| 2012 | IT023CAXXX | 01/08/2012 | CA | ASL 8 CAGLIARI | ovicaprini |
| 2012 | IT044ORXXX | 03/08/2012 | OR | ASL ORISTANO | ovicaprini |
| 2012 | IT077CAXXX | 03/08/2012 | CA | ASL 8 CAGLIARI | ovicaprini |
| 2012 | IT086CAXXX | 03/08/2012 | CA | ASL 8 CAGLIARI | ovicaprini |
| 2012 | IT086CAXXX | 03/08/2012 | CA | ASL 8 CAGLIARI | ovicaprini |
| 2012 | IT052NUXXX | 06/08/2012 | NU | ASL NUORO | ovicaprini |
| 2012 | IT052NUXXX | 06/08/2012 | NU | ASL NUORO | ovicaprini |
| 2012 | IT055CAXXX | 06/08/2012 | CA | ASL 8 CAGLIARI | ovicaprini |
| 2012 | IT029CAXXX | 06/08/2012 | CA | ASL 8 CAGLIARI | ovicaprini |
| 2012 | IT042ORXXX | 07/08/2012 | OR | ASL ORISTANO | ovicaprini |
| 2012 | IT104CAXXX | 07/08/2012 | CA | ASL 8 CAGLIARI | ovicaprini |
| 2012 | IT039ORXXX | 09/08/2012 | OR | ASL ORISTANO | ovicaprini |
| 2012 | IT091CAXXX | 10/08/2012 | CA | ASL 8 CAGLIARI | ovicaprini |
| 2012 | IT087CAXXX | 17/08/2012 | CA | ASL 8 CAGLIARI | ovicaprini |
| 2012 | IT087CAXXX | 17/08/2012 | CA | ASL 8 CAGLIARI | ovicaprini |
| 2012 | IT087CAXXX | 17/08/2012 | CA | ASL 8 CAGLIARI | ovicaprini |
| 2012 | IT029ORXXX | 20/08/2012 | OR | ASL ORISTANO | ovicaprini |
| 2012 | IT060ORXXX | 20/08/2012 | OR | ASL ORISTANO | ovicaprini |
| 2012 | IT104CAXXX | 21/08/2012 | CA | ASL 8 CAGLIARI | ovicaprini |
| 2012 | IT032CAXXX | 22/08/2012 | CA | ASL 8 CAGLIARI | ovicaprini |
| 2012 | IT012ORXXX | 27/08/2012 | OR | ASL ORISTANO | ovicaprini |
| 2012 | IT030ORXXX | 27/08/2012 | OR | ASL ORISTANO | ovicaprini |
| 2012 | IT030ORXXX | 27/08/2012 | OR | ASL ORISTANO | ovicaprini |
| 2012 | IT032CAXXX | 05/09/2012 | CA | ASL 8 CAGLIARI | ovicaprini |
| 2012 | IT057CAXXX | 07/09/2012 | CA | ASL 8 CAGLIARI | ovicaprini |
| 2012 | IT035CAXXX | 07/09/2012 | CA | ASL 8 CAGLIARI | ovicaprini |
| 2012 | IT035CAXXX | 07/09/2012 | CA | ASL 8 CAGLIARI | ovicaprini |
| 2012 | IT012ORXXX | 12/09/2012 | OR | ASL ORISTANO | ovicaprini |
| 2012 | IT091CAXXX | 14/09/2012 | CA | ASL 8 CAGLIARI | ovicaprini |
| 2012 | IT029CAXXX | 24/09/2012 | CA | ASL 8 CAGLIARI | ovicaprini |
| 2012 | IT091CAXXX | 24/09/2012 | CA | ASL 8 CAGLIARI | ovicaprini |
| 2012 | IT096CAXXX | 02/07/2012 | CA | ASL 8 CAGLIARI | ovicaprini |
| 2012 | IT060CAXXX | 06/07/2012 | CA | ASL 8 CAGLIARI | ovicaprini |
| 2012 | IT096CAXXX | 09/07/2012 | CA | ASL 8 CAGLIARI | ovicaprini |
| 2012 | IT096CAXXX | 16/07/2012 | CA | ASL 8 CAGLIARI | ovicaprini |
| 2012 | IT078CAXXX | 23/07/2012 | CA | ASL 8 CAGLIARI | ovicaprini |
| 2012 | IT053CAXXX | 23/07/2012 | CA | ASL 8 CAGLIARI | ovicaprini |
| 2012 | IT060CAXXX | 26/07/2012 | CA | ASL 8 CAGLIARI | ovicaprini |
| 2012 | IT078CAXXX | 30/07/2012 | CA | ASL 8 CAGLIARI | ovicaprini |
| 2012 | IT069CAXXX | 06/08/2012 | CA | ASL 8 CAGLIARI | ovicaprini |
| 2012 | IT031CAXXX | 06/08/2012 | CA | ASL 8 CAGLIARI | ovicaprini |
| 2012 | IT078CAXXX | 06/08/2012 | CA | ASL 8 CAGLIARI | ovicaprini |
| 2012 | IT060CAXXX | 09/08/2012 | CA | ASL 8 CAGLIARI | ovicaprini |
| 2012 | IT078CAXXX | 20/08/2012 | CA | ASL 8 CAGLIARI | ovicaprini |
| 2012 | IT069CAXXX | 20/08/2012 | CA | ASL 8 CAGLIARI | ovicaprini |
| 2012 | IT069CAXXX | 27/08/2012 | CA | ASL 8 CAGLIARI | ovicaprini |
| 2012 | IT091CAXXX | 03/09/2012 | CA | ASL 8 CAGLIARI | ovicaprini |
| 2012 | IT091CAXXX | 10/09/2012 | CA | ASL 8 CAGLIARI | ovicaprini |
| 2012 | IT069CAXXX | 10/09/2012 | CA | ASL 8 CAGLIARI | ovicaprini |
| 2012 | IT065CAXXX | 23/01/2012 | CA | ASL 8 CAGLIARI | ovicaprini |
| 2012 | IT024CAXXX | 01/02/2012 | CA | ASL 8 CAGLIARI | ovicaprini |
| 2012 | IT024CAXXX | 01/02/2012 | CA | ASL 8 CAGLIARI | ovicaprini |
| 2012 | IT024CAXXX | 13/02/2012 | CA | ASL 8 CAGLIARI | ovicaprini |
| 2012 | IT065CAXXX | 13/02/2012 | CA | ASL 8 CAGLIARI | ovicaprini |
| 2012 | IT065CAXXX | 15/02/2012 | CA | ASL 8 CAGLIARI | ovicaprini |
| 2012 | IT034NUXXX | 15/02/2012 | NU | ASL NUORO | ovicaprini |
| 2012 | IT080NUXXX | 15/02/2012 | NU | ASL NUORO | ovicaprini |
| 2012 | IT065CAXXX | 20/02/2012 | CA | ASL 8 CAGLIARI | ovicaprini |
| 2012 | IT065CAXXX | 20/02/2012 | CA | ASL 8 CAGLIARI | ovicaprini |
| 2012 | IT080NUXXX | 24/02/2012 | NU | ASL NUORO | ovicaprini |
| 2012 | IT065CAXXX | 05/03/2012 | CA | ASL 8 CAGLIARI | ovicaprini |
| 2012 | IT031CAXXX | 05/03/2012 | CA | ASL 8 CAGLIARI | ovicaprini |
| 2012 | IT087CAXXX | 05/03/2012 | CA | ASL 8 CAGLIARI | ovicaprini |
| 2012 | IT094CAXXX | 05/03/2012 | CA | ASL 8 CAGLIARI | ovicaprini |
| 2012 | IT065CAXXX | 12/03/2012 | CA | ASL 8 CAGLIARI | ovicaprini |
| 2012 | IT094CAXXX | 12/03/2012 | CA | ASL 8 CAGLIARI | ovicaprini |
| 2012 | IT065CAXXX | 26/03/2012 | CA | ASL 8 CAGLIARI | ovicaprini |
| 2012 | IT057CAXXX | 26/03/2012 | CA | ASL 8 CAGLIARI | ovicaprini |
| 2012 | IT020CAXXX | 09/01/2012 | CA | ASL 8 CAGLIARI | ovicaprini |
| 2012 | IT057CAXXX | 12/01/2012 | CA | ASL 8 CAGLIARI | ovicaprini |
| 2012 | IT078CAXXX | 16/01/2012 | CA | ASL 8 CAGLIARI | ovicaprini |
| 2012 | IT078CAXXX | 19/01/2012 | CA | ASL 8 CAGLIARI | ovicaprini |
| 2012 | IT093CAXXX | 30/01/2012 | CA | ASL 8 CAGLIARI | ovicaprini |
| 2012 | IT032CAXXX | 30/01/2012 | CA | ASL 8 CAGLIARI | ovicaprini |
| 2012 | IT101CAXXX | 06/02/2012 | CA | ASL 8 CAGLIARI | ovicaprini |
| 2012 | IT091CAXXX | 13/02/2012 | CA | ASL 8 CAGLIARI | ovicaprini |
| 2012 | IT057CAXXX | 14/02/2012 | CA | ASL 8 CAGLIARI | ovicaprini |
| 2012 | IT066CAXXX | 20/02/2012 | CA | ASL 8 CAGLIARI | ovicaprini |
| 2012 | IT078CAXXX | 27/02/2012 | CA | ASL 8 CAGLIARI | ovicaprini |
| 2012 | IT078CAXXX | 28/02/2012 | CA | ASL 8 CAGLIARI | ovicaprini |
| 2012 | IT101CAXXX | 01/03/2012 | CA | ASL 8 CAGLIARI | ovicaprini |
| 2012 | IT072CAXXX | 02/03/2012 | CA | ASL 8 CAGLIARI | ovicaprini |
| 2012 | IT091CAXXX | 06/03/2012 | CA | ASL 8 CAGLIARI | ovicaprini |
| 2012 | IT091CAXXX | 06/03/2012 | CA | ASL 8 CAGLIARI | ovicaprini |
| 2012 | IT032CAXXX | 06/03/2012 | CA | ASL 8 CAGLIARI | ovicaprini |
| 2012 | IT057CAXXX | 08/03/2012 | CA | ASL 8 CAGLIARI | ovicaprini |
| 2012 | IT091CAXXX | 15/03/2012 | CA | ASL 8 CAGLIARI | ovicaprini |
| 2012 | IT057CAXXX | 19/03/2012 | CA | ASL 8 CAGLIARI | ovicaprini |
| 2012 | IT033CAXXX | 20/03/2012 | CA | ASL 8 CAGLIARI | ovicaprini |
| 2012 | IT057CAXXX | 20/03/2012 | CA | ASL 8 CAGLIARI | ovicaprini |
| 2012 | IT057CAXXX | 22/03/2012 | CA | ASL 8 CAGLIARI | ovicaprini |
| 2012 | IT057CAXXX | 23/03/2012 | CA | ASL 8 CAGLIARI | ovicaprini |
| 2012 | IT072CAXXX | 26/03/2012 | CA | ASL 8 CAGLIARI | ovicaprini |
| 2012 | IT032CAXXX | 09/01/2012 | CA | ASL 8 CAGLIARI | ovicaprini |
| 2012 | IT032CAXXX | 11/01/2012 | CA | ASL 8 CAGLIARI | ovicaprini |
| 2012 | IT033SSXXX | 13/01/2012 | SS | ASL 1 SASSARI | ovicaprini |
| 2012 | IT033SSXXX | 13/01/2012 | SS | ASL 1 SASSARI | ovicaprini |
| 2012 | IT033SSXXX | 13/01/2012 | SS | ASL 1 SASSARI | ovicaprini |
| 2012 | IT033SSXXX | 13/01/2012 | SS | ASL 1 SASSARI | ovicaprini |
| 2012 | IT092CAXXX | 16/01/2012 | CA | ASL 8 CAGLIARI | ovicaprini |
| 2012 | IT001CAXXX | 16/01/2012 | CA | ASL 8 CAGLIARI | ovicaprini |
| 2012 | IT029CAXXX | 16/01/2012 | CA | ASL 8 CAGLIARI | ovicaprini |
| 2012 | IT032CAXXX | 17/01/2012 | CA | ASL 8 CAGLIARI | ovicaprini |
| 2012 | IT029CAXXX | 17/01/2012 | CA | ASL 8 CAGLIARI | ovicaprini |
| 2012 | IT078CAXXX | 18/01/2012 | CA | ASL 8 CAGLIARI | ovicaprini |
| 2012 | IT033CAXXX | 18/01/2012 | CA | ASL 8 CAGLIARI | ovicaprini |
| 2012 | IT052CAXXX | 18/01/2012 | CA | ASL 8 CAGLIARI | ovicaprini |
| 2012 | IT043ORXXX | 23/01/2012 | OR | ASL ORISTANO | ovicaprini |
| 2012 | IT001CAXXX | 23/01/2012 | CA | ASL 8 CAGLIARI | ovicaprini |
| 2012 | IT092CAXXX | 23/01/2012 | CA | ASL 8 CAGLIARI | ovicaprini |
| 2012 | IT032CAXXX | 23/01/2012 | CA | ASL 8 CAGLIARI | ovicaprini |
| 2012 | IT055CAXXX | 30/01/2012 | CA | ASL 8 CAGLIARI | ovicaprini |
| 2012 | IT001CAXXX | 30/01/2012 | CA | ASL 8 CAGLIARI | ovicaprini |
| 2012 | IT057ORXXX | 31/01/2012 | OR | ASL ORISTANO | ovicaprini |
| 2012 | IT047ORXXX | 31/01/2012 | OR | ASL ORISTANO | ovicaprini |
| 2012 | IT041CAXXX | 03/02/2012 | CA | ASL 8 CAGLIARI | ovicaprini |
| 2012 | IT039ORXXX | 03/02/2012 | OR | ASL ORISTANO | ovicaprini |
| 2012 | IT039ORXXX | 03/02/2012 | OR | ASL ORISTANO | ovicaprini |
| 2012 | IT032CAXXX | 06/02/2012 | CA | ASL 8 CAGLIARI | ovicaprini |
| 2012 | IT092CAXXX | 06/02/2012 | CA | ASL 8 CAGLIARI | ovicaprini |
| 2012 | IT029CAXXX | 07/02/2012 | CA | ASL 8 CAGLIARI | ovicaprini |
| 2012 | IT027ORXXX | 08/02/2012 | OR | ASL ORISTANO | ovicaprini |
| 2012 | IT032CAXXX | 08/02/2012 | CA | ASL 8 CAGLIARI | ovicaprini |
| 2012 | IT031ORXXX | 13/02/2012 | OR | ASL ORISTANO | ovicaprini |
| 2012 | IT049ORXXX | 13/02/2012 | OR | ASL ORISTANO | ovicaprini |
| 2012 | IT092CAXXX | 13/02/2012 | CA | ASL 8 CAGLIARI | ovicaprini |
| 2012 | IT092CAXXX | 13/02/2012 | CA | ASL 8 CAGLIARI | ovicaprini |
| 2012 | IT052CAXXX | 14/02/2012 | CA | ASL 8 CAGLIARI | ovicaprini |
| 2012 | IT086CAXXX | 14/02/2012 | CA | ASL 8 CAGLIARI | ovicaprini |
| 2012 | IT055CAXXX | 20/02/2012 | CA | ASL 8 CAGLIARI | ovicaprini |
| 2012 | IT045CAXXX | 20/02/2012 | CA | ASL 8 CAGLIARI | ovicaprini |
| 2012 | IT042ORXXX | 21/02/2012 | OR | ASL ORISTANO | ovicaprini |
| 2012 | IT011ORXXX | 21/02/2012 | OR | ASL ORISTANO | ovicaprini |
| 2012 | IT029ORXXX | 21/02/2012 | OR | ASL ORISTANO | ovicaprini |
| 2012 | IT011ORXXX | 21/02/2012 | OR | ASL ORISTANO | ovicaprini |
| 2012 | IT092CAXXX | 21/02/2012 | CA | ASL 8 CAGLIARI | ovicaprini |
| 2012 | IT029CAXXX | 27/02/2012 | CA | ASL 8 CAGLIARI | ovicaprini |
| 2012 | IT043ORXXX | 27/02/2012 | OR | ASL ORISTANO | ovicaprini |
| 2012 | IT043ORXXX | 27/02/2012 | OR | ASL ORISTANO | ovicaprini |
| 2012 | IT032CAXXX | 27/02/2012 | CA | ASL 8 CAGLIARI | ovicaprini |
| 2012 | IT029CAXXX | 27/02/2012 | CA | ASL 8 CAGLIARI | ovicaprini |
| 2012 | IT057ORXXX | 28/02/2012 | OR | ASL ORISTANO | ovicaprini |
| 2012 | IT046CAXXX | 28/02/2012 | CA | ASL 8 CAGLIARI | ovicaprini |
| 2012 | IT092CAXXX | 28/02/2012 | CA | ASL 8 CAGLIARI | ovicaprini |
| 2012 | IT029ORXXX | 05/03/2012 | OR | ASL ORISTANO | ovicaprini |
| 2012 | IT029ORXXX | 05/03/2012 | OR | ASL ORISTANO | ovicaprini |
| 2012 | IT092CAXXX | 05/03/2012 | CA | ASL 8 CAGLIARI | ovicaprini |
| 2012 | IT045CAXXX | 05/03/2012 | CA | ASL 8 CAGLIARI | ovicaprini |
| 2012 | IT014CAXXX | 05/03/2012 | CA | ASL 8 CAGLIARI | ovicaprini |
| 2012 | IT055CAXXX | 06/03/2012 | CA | ASL 8 CAGLIARI | ovicaprini |
| 2012 | IT045CAXXX | 06/03/2012 | CA | ASL 8 CAGLIARI | ovicaprini |
| 2012 | IT045CAXXX | 06/03/2012 | CA | ASL 8 CAGLIARI | ovicaprini |
| 2012 | IT055CAXXX | 06/03/2012 | CA | ASL 8 CAGLIARI | ovicaprini |
| 2012 | IT032CAXXX | 06/03/2012 | CA | ASL 8 CAGLIARI | ovicaprini |
| 2012 | IT012ORXXX | 12/03/2012 | OR | ASL ORISTANO | ovicaprini |
| 2012 | IT045CAXXX | 12/03/2012 | CA | ASL 8 CAGLIARI | ovicaprini |
| 2012 | IT055CAXXX | 12/03/2012 | CA | ASL 8 CAGLIARI | ovicaprini |
| 2012 | IT101CAXXX | 12/03/2012 | CA | ASL 8 CAGLIARI | ovicaprini |
| 2012 | IT055CAXXX | 13/03/2012 | CA | ASL 8 CAGLIARI | ovicaprini |
| 2012 | IT039ORXXX | 14/03/2012 | OR | ASL ORISTANO | ovicaprini |
| 2012 | IT055CAXXX | 14/03/2012 | CA | ASL 8 CAGLIARI | ovicaprini |
| 2012 | IT101CAXXX | 19/03/2012 | CA | ASL 8 CAGLIARI | ovicaprini |
| 2012 | IT029ORXXX | 19/03/2012 | OR | ASL ORISTANO | ovicaprini |
| 2012 | IT012ORXXX | 19/03/2012 | OR | ASL ORISTANO | ovicaprini |
| 2012 | IT029ORXXX | 19/03/2012 | OR | ASL ORISTANO | ovicaprini |
| 2012 | IT029ORXXX | 20/03/2012 | OR | ASL ORISTANO | ovicaprini |
| 2012 | IT040CAXXX | 21/03/2012 | CA | ASL 8 CAGLIARI | ovicaprini |
| 2012 | IT029CAXXX | 21/03/2012 | CA | ASL 8 CAGLIARI | ovicaprini |
| 2012 | IT032CAXXX | 21/03/2012 | CA | ASL 8 CAGLIARI | ovicaprini |
| 2012 | IT055CAXXX | 26/03/2012 | CA | ASL 8 CAGLIARI | ovicaprini |
| 2012 | IT059SSXXX | 26/03/2012 | SS | ASL 1 SASSARI | ovicaprini |
| 2012 | IT029CAXXX | 26/03/2012 | CA | ASL 8 CAGLIARI | ovicaprini |
| 2012 | IT101CAXXX | 26/03/2012 | CA | ASL 8 CAGLIARI | ovicaprini |
| 2012 | IT045CAXXX | 26/03/2012 | CA | ASL 8 CAGLIARI | ovicaprini |
| 2012 | IT011ORXXX | 26/03/2012 | OR | ASL ORISTANO | ovicaprini |
| 2012 | IT039ORXXX | 27/03/2012 | OR | ASL ORISTANO | ovicaprini |
| 2012 | IT001CAXXX | 31/03/2012 | CA | ASL 8 CAGLIARI | ovicaprini |
| 2012 | IT092CAXXX | 13/02/2012 | CA | ASL 8 CAGLIARI | ovicaprini |
| 2012 | IT029CAXXX | 20/02/2012 | CA | ASL 8 CAGLIARI | ovicaprini |
| 2012 | IT092CAXXX | 19/03/2012 | CA | ASL 8 CAGLIARI | ovicaprini |
| 2012 | IT091CAXXX | 02/04/2012 | CA | ASL 8 CAGLIARI | ovicaprini |
| 2012 | IT101CAXXX | 02/04/2012 | CA | ASL 8 CAGLIARI | ovicaprini |
| 2012 | IT057ORXXX | 02/04/2012 | OR | ASL ORISTANO | ovicaprini |
| 2012 | IT045CAXXX | 02/04/2012 | CA | ASL 8 CAGLIARI | ovicaprini |
| 2012 | IT012ORXXX | 04/04/2012 | OR | ASL ORISTANO | ovicaprini |
| 2012 | IT072CAXXX | 05/04/2012 | CA | ASL 8 CAGLIARI | ovicaprini |
| 2012 | IT072CAXXX | 10/04/2012 | CA | ASL 8 CAGLIARI | ovicaprini |
| 2012 | IT032CAXXX | 10/04/2012 | CA | ASL 8 CAGLIARI | ovicaprini |
| 2012 | IT032CAXXX | 10/04/2012 | CA | ASL 8 CAGLIARI | ovicaprini |
| 2012 | IT048ORXXX | 10/04/2012 | OR | ASL ORISTANO | ovicaprini |
| 2012 | IT045CAXXX | 10/04/2012 | CA | ASL 8 CAGLIARI | ovicaprini |
| 2012 | IT044ORXXX | 11/04/2012 | OR | ASL ORISTANO | ovicaprini |
| 2012 | IT101CAXXX | 12/04/2012 | CA | ASL 8 CAGLIARI | ovicaprini |
| 2012 | IT032CAXXX | 12/04/2012 | CA | ASL 8 CAGLIARI | ovicaprini |
| 2012 | IT032CAXXX | 12/04/2012 | CA | ASL 8 CAGLIARI | ovicaprini |
| 2012 | IT092CAXXX | 13/04/2012 | CA | ASL 8 CAGLIARI | ovicaprini |
| 2012 | IT092CAXXX | 13/04/2012 | CA | ASL 8 CAGLIARI | ovicaprini |
| 2012 | IT072CAXXX | 13/04/2012 | CA | ASL 8 CAGLIARI | ovicaprini |
| 2012 | IT072CAXXX | 13/04/2012 | CA | ASL 8 CAGLIARI | ovicaprini |
| 2012 | IT072CAXXX | 16/04/2012 | CA | ASL 8 CAGLIARI | ovicaprini |
| 2012 | IT072CAXXX | 16/04/2012 | CA | ASL 8 CAGLIARI | ovicaprini |
| 2012 | IT032CAXXX | 16/04/2012 | CA | ASL 8 CAGLIARI | ovicaprini |
| 2012 | IT029CAXXX | 16/04/2012 | CA | ASL 8 CAGLIARI | ovicaprini |
| 2012 | IT028ORXXX | 16/04/2012 | OR | ASL ORISTANO | ovicaprini |
| 2012 | IT101CAXXX | 16/04/2012 | CA | ASL 8 CAGLIARI | ovicaprini |
| 2012 | IT023CAXXX | 16/04/2012 | CA | ASL 8 CAGLIARI | ovicaprini |
| 2012 | IT023CAXXX | 16/04/2012 | CA | ASL 8 CAGLIARI | ovicaprini |
| 2012 | IT045CAXXX | 16/04/2012 | CA | ASL 8 CAGLIARI | ovicaprini |
| 2012 | IT072CAXXX | 17/04/2012 | CA | ASL 8 CAGLIARI | ovicaprini |
| 2012 | IT057CAXXX | 17/04/2012 | CA | ASL 8 CAGLIARI | ovicaprini |
| 2012 | IT032CAXXX | 17/04/2012 | CA | ASL 8 CAGLIARI | ovicaprini |
| 2012 | IT032CAXXX | 17/04/2012 | CA | ASL 8 CAGLIARI | ovicaprini |
| 2012 | IT032CAXXX | 17/04/2012 | CA | ASL 8 CAGLIARI | ovicaprini |
| 2012 | IT057CAXXX | 17/04/2012 | CA | ASL 8 CAGLIARI | ovicaprini |
| 2012 | IT032CAXXX | 17/04/2012 | CA | ASL 8 CAGLIARI | ovicaprini |
| 2012 | IT044ORXXX | 17/04/2012 | OR | ASL ORISTANO | ovicaprini |
| 2012 | IT047ORXXX | 17/04/2012 | OR | ASL ORISTANO | ovicaprini |
| 2012 | IT006CAXXX | 18/04/2012 | CA | ASL 8 CAGLIARI | ovicaprini |
| 2012 | IT006CAXXX | 18/04/2012 | CA | ASL 8 CAGLIARI | ovicaprini |
| 2012 | IT006CAXXX | 18/04/2012 | CA | ASL 8 CAGLIARI | ovicaprini |
| 2012 | IT059ORXXX | 18/04/2012 | OR | ASL ORISTANO | ovicaprini |
| 2012 | IT019CAXXX | 19/04/2012 | CA | ASL 8 CAGLIARI | ovicaprini |
| 2012 | IT019CAXXX | 20/04/2012 | CA | ASL 8 CAGLIARI | ovicaprini |
| 2012 | IT033CAXXX | 20/04/2012 | CA | ASL 8 CAGLIARI | ovicaprini |
| 2012 | IT086CAXXX | 20/04/2012 | CA | ASL 8 CAGLIARI | ovicaprini |
| 2012 | IT086CAXXX | 20/04/2012 | CA | ASL 8 CAGLIARI | ovicaprini |
| 2012 | IT072CAXXX | 23/04/2012 | CA | ASL 8 CAGLIARI | ovicaprini |
| 2012 | IT025CAXXX | 23/04/2012 | CA | ASL 8 CAGLIARI | ovicaprini |
| 2012 | IT057CAXXX | 23/04/2012 | CA | ASL 8 CAGLIARI | ovicaprini |
| 2012 | IT069ORXXX | 23/04/2012 | OR | ASL ORISTANO | ovicaprini |
| 2012 | IT101CAXXX | 23/04/2012 | CA | ASL 8 CAGLIARI | ovicaprini |
| 2012 | IT032CAXXX | 23/04/2012 | CA | ASL 8 CAGLIARI | ovicaprini |
| 2012 | IT078CAXXX | 23/04/2012 | CA | ASL 8 CAGLIARI | ovicaprini |
| 2012 | IT078CAXXX | 23/04/2012 | CA | ASL 8 CAGLIARI | ovicaprini |
| 2012 | IT045CAXXX | 23/04/2012 | CA | ASL 8 CAGLIARI | ovicaprini |
| 2012 | IT040CAXXX | 24/04/2012 | CA | ASL 8 CAGLIARI | ovicaprini |
| 2012 | IT040CAXXX | 24/04/2012 | CA | ASL 8 CAGLIARI | ovicaprini |
| 2012 | IT046CAXXX | 24/04/2012 | CA | ASL 8 CAGLIARI | ovicaprini |
| 2012 | IT078CAXXX | 26/04/2012 | CA | ASL 8 CAGLIARI | ovicaprini |
| 2012 | IT001CAXXX | 26/04/2012 | CA | ASL 8 CAGLIARI | ovicaprini |
| 2012 | IT065CAXXX | 26/04/2012 | CA | ASL 8 CAGLIARI | ovicaprini |
| 2012 | IT0078CXXX | 27/04/2012 | CA | ASL 8 CAGLIARI | ovicaprini |
| 2012 | IT029CAXXX | 27/04/2012 | CA | ASL 8 CAGLIARI | ovicaprini |
| 2012 | IT055CAXXX | 30/04/2012 | CA | ASL 8 CAGLIARI | ovicaprini |
| 2012 | IT078CAXXX | 30/04/2012 | CA | ASL 8 CAGLIARI | ovicaprini |
| 2012 | IT020CAXXX | 30/04/2012 | CA | ASL 8 CAGLIARI | ovicaprini |
| 2012 | IT012ORXXX | 30/04/2012 | OR | ASL ORISTANO | ovicaprini |
| 2012 | IT029CAXXX | 30/04/2012 | CA | ASL 8 CAGLIARI | ovicaprini |
| 2012 | IT029CAXXX | 30/04/2012 | CA | ASL 8 CAGLIARI | ovicaprini |
| 2012 | IT086CAXXX | 30/04/2012 | CA | ASL 8 CAGLIARI | ovicaprini |
| 2012 | IT052CAXXX | 03/05/2012 | CA | ASL 8 CAGLIARI | ovicaprini |
| 2012 | IT072CAXXX | 03/05/2012 | CA | ASL 8 CAGLIARI | ovicaprini |
| 2012 | IT020CAXXX | 03/05/2012 | CA | ASL 8 CAGLIARI | ovicaprini |
| 2012 | IT096CAXXX | 07/05/2012 | CA | ASL 8 CAGLIARI | ovicaprini |
| 2012 | IT016CAXXX | 07/05/2012 | CA | ASL 8 CAGLIARI | ovicaprini |
| 2012 | IT055CAXXX | 07/05/2012 | CA | ASL 8 CAGLIARI | ovicaprini |
| 2012 | IT055CAXXX | 07/05/2012 | CA | ASL 8 CAGLIARI | ovicaprini |
| 2012 | IT092CAXXX | 07/05/2012 | CA | ASL 8 CAGLIARI | ovicaprini |
| 2012 | IT055CAXXX | 07/05/2012 | CA | ASL 8 CAGLIARI | ovicaprini |
| 2012 | IT012ORXXX | 07/05/2012 | OR | ASL ORISTANO | ovicaprini |
| 2012 | IT032CAXXX | 07/05/2012 | CA | ASL 8 CAGLIARI | ovicaprini |
| 2012 | IT045CAXXX | 07/05/2012 | CA | ASL 8 CAGLIARI | ovicaprini |
| 2012 | IT029ORXXX | 07/05/2012 | OR | ASL ORISTANO | ovicaprini |
| 2012 | IT016CAXXX | 08/05/2012 | CA | ASL 8 CAGLIARI | ovicaprini |
| 2012 | IT057CAXXX | 08/05/2012 | CA | ASL 8 CAGLIARI | ovicaprini |
| 2012 | IT032CAXXX | 08/05/2012 | CA | ASL 8 CAGLIARI | ovicaprini |
| 2012 | IT092CAXXX | 08/05/2012 | CA | ASL 8 CAGLIARI | ovicaprini |
| 2012 | IT044ORXXX | 08/05/2012 | OR | ASL ORISTANO | ovicaprini |
| 2012 | IT032CAXXX | 09/05/2012 | CA | ASL 8 CAGLIARI | ovicaprini |
| 2012 | IT092CAXXX | 10/05/2012 | CA | ASL 8 CAGLIARI | ovicaprini |
| 2012 | IT001CAXXX | 10/05/2012 | CA | ASL 8 CAGLIARI | ovicaprini |
| 2012 | IT016CAXXX | 10/05/2012 | CA | ASL 8 CAGLIARI | ovicaprini |
| 2012 | IT101CAXXX | 10/05/2012 | CA | ASL 8 CAGLIARI | ovicaprini |
| 2012 | IT040CAXXX | 10/05/2012 | CA | ASL 8 CAGLIARI | ovicaprini |
| 2012 | IT092CAXXX | 11/05/2012 | CA | ASL 8 CAGLIARI | ovicaprini |
| 2012 | IT016CAXXX | 11/05/2012 | CA | ASL 8 CAGLIARI | ovicaprini |
| 2012 | IT032CAXXX | 11/05/2012 | CA | ASL 8 CAGLIARI | ovicaprini |
| 2012 | IT032CAXXX | 11/05/2012 | CA | ASL 8 CAGLIARI | ovicaprini |
| 2012 | IT016CAXXX | 14/05/2012 | CA | ASL 8 CAGLIARI | ovicaprini |
| 2012 | IT032CAXXX | 14/05/2012 | CA | ASL 8 CAGLIARI | ovicaprini |
| 2012 | IT057CAXXX | 14/05/2012 | CA | ASL 8 CAGLIARI | ovicaprini |
| 2012 | IT072CAXXX | 14/05/2012 | CA | ASL 8 CAGLIARI | ovicaprini |
| 2012 | IT045CAXXX | 14/05/2012 | CA | ASL 8 CAGLIARI | ovicaprini |
| 2012 | IT032CAXXX | 14/05/2012 | CA | ASL 8 CAGLIARI | ovicaprini |
| 2012 | IT055CAXXX | 14/05/2012 | CA | ASL 8 CAGLIARI | ovicaprini |
| 2012 | IT046CAXXX | 14/05/2012 | CA | ASL 8 CAGLIARI | ovicaprini |
| 2012 | IT032CAXXX | 14/05/2012 | CA | ASL 8 CAGLIARI | ovicaprini |
| 2012 | IT045CAXXX | 14/05/2012 | CA | ASL 8 CAGLIARI | ovicaprini |
| 2012 | IT045CAXXX | 14/05/2012 | CA | ASL 8 CAGLIARI | ovicaprini |
| 2012 | IT044CAXXX | 15/05/2012 | CA | ASL 8 CAGLIARI | ovicaprini |
| 2012 | IT012ORXXX | 18/05/2012 | OR | ASL ORISTANO | ovicaprini |
| 2012 | IT092CAXXX | 18/05/2012 | CA | ASL 8 CAGLIARI | ovicaprini |
| 2012 | IT065CAXXX | 18/05/2012 | CA | ASL 8 CAGLIARI | ovicaprini |
| 2012 | IT006CAXXX | 18/05/2012 | CA | ASL 8 CAGLIARI | ovicaprini |
| 2012 | IT006CAXXX | 18/05/2012 | CA | ASL 8 CAGLIARI | ovicaprini |
| 2012 | IT101CAXXX | 18/05/2012 | CA | ASL 8 CAGLIARI | ovicaprini |
| 2012 | IT012ORXXX | 21/05/2012 | OR | ASL ORISTANO | ovicaprini |
| 2012 | IT072CAXXX | 21/05/2012 | CA | ASL 8 CAGLIARI | ovicaprini |
| 2012 | IT032CAXXX | 21/05/2012 | CA | ASL 8 CAGLIARI | ovicaprini |
| 2012 | IT029ORXXX | 21/05/2012 | OR | ASL ORISTANO | ovicaprini |
| 2012 | IT077ORXXX | 21/05/2012 | OR | ASL ORISTANO | ovicaprini |
| 2012 | IT029ORXXX | 21/05/2012 | OR | ASL ORISTANO | ovicaprini |
| 2012 | IT032CAXXX | 21/05/2012 | CA | ASL 8 CAGLIARI | ovicaprini |
| 2012 | IT092CAXXX | 21/05/2012 | CA | ASL 8 CAGLIARI | ovicaprini |
| 2012 | IT045CAXXX | 21/05/2012 | CA | ASL 8 CAGLIARI | ovicaprini |
| 2012 | IT094CAXXX | 21/05/2012 | CA | ASL 8 CAGLIARI | ovicaprini |
| 2012 | IT029ORXXX | 21/05/2012 | OR | ASL ORISTANO | ovicaprini |
| 2012 | IT029ORXXX | 21/05/2012 | OR | ASL ORISTANO | ovicaprini |
| 2012 | IT012CAXXX | 21/05/2012 | CA | ASL 8 CAGLIARI | ovicaprini |
| 2012 | IT094CAXXX | 21/05/2012 | CA | ASL 8 CAGLIARI | ovicaprini |
| 2012 | IT032CAXXX | 22/05/2012 | CA | ASL 8 CAGLIARI | ovicaprini |
| 2012 | IT092CAXXX | 22/05/2012 | CA | ASL 8 CAGLIARI | ovicaprini |
| 2012 | IT072CAXXX | 22/05/2012 | CA | ASL 8 CAGLIARI | ovicaprini |
| 2012 | IT055CAXXX | 22/05/2012 | CA | ASL 8 CAGLIARI | ovicaprini |
| 2012 | IT006CAXXX | 23/05/2012 | CA | ASL 8 CAGLIARI | ovicaprini |
| 2012 | IT078CAXXX | 23/05/2012 | CA | ASL 8 CAGLIARI | ovicaprini |
| 2012 | IT092CAXXX | 24/05/2012 | CA | ASL 8 CAGLIARI | ovicaprini |
| 2012 | IT091CAXXX | 24/05/2012 | CA | ASL 8 CAGLIARI | ovicaprini |
| 2012 | IT092CAXXX | 24/05/2012 | CA | ASL 8 CAGLIARI | ovicaprini |
| 2012 | IT092CAXXX | 24/05/2012 | CA | ASL 8 CAGLIARI | ovicaprini |
| 2012 | IT096CAXXX | 25/05/2012 | CA | ASL 8 CAGLIARI | ovicaprini |
| 2012 | IT092CAXXX | 25/05/2012 | CA | ASL 8 CAGLIARI | ovicaprini |
| 2012 | IT091CAXXX | 25/05/2012 | CA | ASL 8 CAGLIARI | ovicaprini |
| 2012 | IT096CAXXX | 28/05/2012 | CA | ASL 8 CAGLIARI | ovicaprini |
| 2012 | IT091CAXXX | 28/05/2012 | CA | ASL 8 CAGLIARI | ovicaprini |
| 2012 | IT044ORXXX | 28/05/2012 | OR | ASL ORISTANO | ovicaprini |
| 2012 | IT045CAXXX | 28/05/2012 | CA | ASL 8 CAGLIARI | ovicaprini |
| 2012 | IT042ORXXX | 28/05/2012 | OR | ASL ORISTANO | ovicaprini |
| 2012 | IT092CAXXX | 28/05/2012 | CA | ASL 8 CAGLIARI | ovicaprini |
| 2012 | IT026ORXXX | 28/05/2012 | OR | ASL ORISTANO | ovicaprini |
| 2012 | IT028ORXXX | 28/05/2012 | OR | ASL ORISTANO | ovicaprini |
| 2012 | IT032CAXXX | 29/05/2012 | CA | ASL 8 CAGLIARI | ovicaprini |
| 2012 | IT032CAXXX | 29/05/2012 | CA | ASL 8 CAGLIARI | ovicaprini |
| 2012 | IT039ORXXX | 29/05/2012 | OR | ASL ORISTANO | ovicaprini |
| 2012 | IT065CAXXX | 30/05/2012 | CA | ASL 8 CAGLIARI | ovicaprini |
| 2012 | IT065CAXXX | 30/05/2012 | CA | ASL 8 CAGLIARI | ovicaprini |
| 2012 | IT032CAXXX | 30/05/2012 | CA | ASL 8 CAGLIARI | ovicaprini |
| 2012 | IT036NUXXX | 31/05/2012 | NU | ASL NUORO | ovicaprini |
| 2012 | IT092CAXXX | 31/05/2012 | CA | ASL 8 CAGLIARI | ovicaprini |
| 2012 | IT057CAXXX | 31/05/2012 | CA | ASL 8 CAGLIARI | ovicaprini |
| 2012 | IT078CAXXX | 31/05/2012 | CA | ASL 8 CAGLIARI | ovicaprini |
| 2012 | IT078CAXXX | 01/06/2012 | CA | ASL 8 CAGLIARI | ovicaprini |
| 2012 | IT096CAXXX | 04/06/2012 | CA | ASL 8 CAGLIARI | ovicaprini |
| 2012 | IT020CAXXX | 04/06/2012 | CA | ASL 8 CAGLIARI | ovicaprini |
| 2012 | IT091CAXXX | 04/06/2012 | CA | ASL 8 CAGLIARI | ovicaprini |
| 2012 | IT092CAXXX | 04/06/2012 | CA | ASL 8 CAGLIARI | ovicaprini |
| 2012 | IT043ORXXX | 04/06/2012 | OR | ASL ORISTANO | ovicaprini |
| 2012 | IT029CAXXX | 04/06/2012 | CA | ASL 8 CAGLIARI | ovicaprini |
| 2012 | IT045CAXXX | 04/06/2012 | CA | ASL 8 CAGLIARI | ovicaprini |
| 2012 | IT065CAXXX | 04/06/2012 | CA | ASL 8 CAGLIARI | ovicaprini |
| 2012 | IT040CAXXX | 05/06/2012 | CA | ASL 8 CAGLIARI | ovicaprini |
| 2012 | IT092CAXXX | 05/06/2012 | CA | ASL 8 CAGLIARI | ovicaprini |
| 2012 | IT093CAXXX | 05/06/2012 | CA | ASL 8 CAGLIARI | ovicaprini |
| 2012 | IT039ORXXX | 05/06/2012 | OR | ASL ORISTANO | ovicaprini |
| 2012 | IT046ORXXX | 05/06/2012 | OR | ASL ORISTANO | ovicaprini |
| 2012 | IT016CAXXX | 07/06/2012 | CA | ASL 8 CAGLIARI | ovicaprini |
| 2012 | IT092CAXXX | 07/06/2012 | CA | ASL 8 CAGLIARI | ovicaprini |
| 2012 | IT093CAXXX | 07/06/2012 | CA | ASL 8 CAGLIARI | ovicaprini |
| 2012 | IT016CAXXX | 08/06/2012 | CA | ASL 8 CAGLIARI | ovicaprini |
| 2012 | IT016CAXXX | 11/06/2012 | CA | ASL 8 CAGLIARI | ovicaprini |
| 2012 | IT016CAXXX | 11/06/2012 | CA | ASL 8 CAGLIARI | ovicaprini |
| 2012 | IT057CAXXX | 11/06/2012 | CA | ASL 8 CAGLIARI | ovicaprini |
| 2012 | IT016CAXXX | 11/06/2012 | CA | ASL 8 CAGLIARI | ovicaprini |
| 2012 | IT091CAXXX | 11/06/2012 | CA | ASL 8 CAGLIARI | ovicaprini |
| 2012 | IT091CAXXX | 11/06/2012 | CA | ASL 8 CAGLIARI | ovicaprini |
| 2012 | IT055CAXXX | 11/06/2012 | CA | ASL 8 CAGLIARI | ovicaprini |
| 2012 | IT086CAXXX | 11/06/2012 | CA | ASL 8 CAGLIARI | ovicaprini |
| 2012 | IT091CAXXX | 11/06/2012 | CA | ASL 8 CAGLIARI | ovicaprini |
| 2012 | IT043ORXXX | 11/06/2012 | OR | ASL ORISTANO | ovicaprini |
| 2012 | IT045CAXXX | 11/06/2012 | CA | ASL 8 CAGLIARI | ovicaprini |
| 2012 | IT086CAXXX | 11/06/2012 | CA | ASL 8 CAGLIARI | ovicaprini |
| 2012 | IT043ORXXX | 11/06/2012 | OR | ASL ORISTANO | ovicaprini |
| 2012 | IT019CAXXX | 12/06/2012 | CA | ASL 8 CAGLIARI | ovicaprini |
| 2012 | IT040CAXXX | 12/06/2012 | CA | ASL 8 CAGLIARI | ovicaprini |
| 2012 | IT004ORXXX | 12/06/2012 | OR | ASL ORISTANO | ovicaprini |
| 2012 | IT036NUXXX | 14/06/2012 | NU | ASL NUORO | ovicaprini |
| 2012 | IT092CAXXX | 14/06/2012 | CA | ASL 8 CAGLIARI | ovicaprini |
| 2012 | IT033CAXXX | 14/06/2012 | CA | ASL 8 CAGLIARI | ovicaprini |
| 2012 | IT033CAXXX | 15/06/2012 | CA | ASL 8 CAGLIARI | ovicaprini |
| 2012 | IT057CAXXX | 18/06/2012 | CA | ASL 8 CAGLIARI | ovicaprini |
| 2012 | IT057CAXXX | 18/06/2012 | CA | ASL 8 CAGLIARI | ovicaprini |
| 2012 | IT025CAXXX | 18/06/2012 | CA | ASL 8 CAGLIARI | ovicaprini |
| 2012 | IT033CAXXX | 18/06/2012 | CA | ASL 8 CAGLIARI | ovicaprini |
| 2012 | IT052CAXXX | 18/06/2012 | CA | ASL 8 CAGLIARI | ovicaprini |
| 2012 | IT045CAXXX | 18/06/2012 | CA | ASL 8 CAGLIARI | ovicaprini |
| 2012 | IT006CAXXX | 18/06/2012 | CA | ASL 8 CAGLIARI | ovicaprini |
| 2012 | IT012ORXXX | 18/06/2012 | OR | ASL ORISTANO | ovicaprini |
| 2012 | IT086CAXXX | 18/06/2012 | CA | ASL 8 CAGLIARI | ovicaprini |
| 2012 | IT092CAXXX | 19/06/2012 | CA | ASL 8 CAGLIARI | ovicaprini |
| 2012 | IT022CAXXX | 19/06/2012 | CA | ASL 8 CAGLIARI | ovicaprini |
| 2012 | IT004ORXXX | 19/06/2012 | OR | ASL ORISTANO | ovicaprini |
| 2012 | IT055CAXXX | 20/06/2012 | CA | ASL 8 CAGLIARI | ovicaprini |
| 2012 | IT055CAXXX | 20/06/2012 | CA | ASL 8 CAGLIARI | ovicaprini |
| 2012 | IT092CAXXX | 21/06/2012 | CA | ASL 8 CAGLIARI | ovicaprini |
| 2012 | IT094CAXXX | 21/06/2012 | CA | ASL 8 CAGLIARI | ovicaprini |
| 2012 | IT092CAXXX | 21/06/2012 | CA | ASL 8 CAGLIARI | ovicaprini |
| 2012 | IT016CAXXX | 25/06/2012 | CA | ASL 8 CAGLIARI | ovicaprini |
| 2012 | IT046CAXXX | 25/06/2012 | CA | ASL 8 CAGLIARI | ovicaprini |
| 2012 | IT046CAXXX | 25/06/2012 | CA | ASL 8 CAGLIARI | ovicaprini |
| 2012 | IT029CAXXX | 25/06/2012 | CA | ASL 8 CAGLIARI | ovicaprini |
| 2012 | IT078CAXXX | 25/06/2012 | CA | ASL 8 CAGLIARI | ovicaprini |
| 2012 | IT012ORXXX | 25/06/2012 | OR | ASL ORISTANO | ovicaprini |
| 2012 | IT029CAXXX | 25/06/2012 | CA | ASL 8 CAGLIARI | ovicaprini |
| 2012 | IT012ORXXX | 25/06/2012 | OR | ASL ORISTANO | ovicaprini |
| 2012 | IT057CAXXX | 25/06/2012 | CA | ASL 8 CAGLIARI | ovicaprini |
| 2012 | IT040CAXXX | 26/06/2012 | CA | ASL 8 CAGLIARI | ovicaprini |
| 2012 | IT033CAXXX | 26/06/2012 | CA | ASL 8 CAGLIARI | ovicaprini |
| 2012 | IT019CAXXX | 26/06/2012 | CA | ASL 8 CAGLIARI | ovicaprini |
| 2012 | IT019CAXXX | 26/06/2012 | CA | ASL 8 CAGLIARI | ovicaprini |
| 2012 | IT072CAXXX | 26/06/2012 | CA | ASL 8 CAGLIARI | ovicaprini |
| 2012 | IT004ORXXX | 26/06/2012 | OR | ASL ORISTANO | ovicaprini |
| 2012 | IT091CAXXX | 28/06/2012 | CA | ASL 8 CAGLIARI | ovicaprini |
| 2012 | IT091CAXXX | 28/06/2012 | CA | ASL 8 CAGLIARI | ovicaprini |
| 2012 | IT052CAXXX | 29/06/2012 | CA | ASL 8 CAGLIARI | ovicaprini |
| 2012 | IT091CAXXX | 29/06/2012 | CA | ASL 8 CAGLIARI | ovicaprini |
| 2012 | IT092CAXXX | 29/06/2012 | CA | ASL 8 CAGLIARI | ovicaprini |
| 2012 | IT020CAXXX | 03/05/2012 | CA | ASL 8 CAGLIARI | ovicaprini |
| 2012 | IT069ORXXX | 02/07/2012 | OR | ASL ORISTANO | ovicaprini |
| 2012 | IT069ORXXX | 02/07/2012 | OR | ASL ORISTANO | ovicaprini |
| 2012 | IT001CAXXX | 02/07/2012 | CA | ASL 8 CAGLIARI | ovicaprini |
| 2012 | IT055CAXXX | 02/07/2012 | CA | ASL 8 CAGLIARI | ovicaprini |
| 2012 | IT042ORXXX | 02/07/2012 | OR | ASL ORISTANO | ovicaprini |
| 2012 | IT078CAXXX | 02/07/2012 | CA | ASL 8 CAGLIARI | ovicaprini |
| 2012 | IT029CAXXX | 09/07/2012 | CA | ASL 8 CAGLIARI | ovicaprini |
| 2012 | IT010ORXXX | 09/07/2012 | OR | ASL ORISTANO | ovicaprini |
| 2012 | IT078CAXXX | 09/07/2012 | CA | ASL 8 CAGLIARI | ovicaprini |
| 2012 | IT046ORXXX | 09/07/2012 | OR | ASL ORISTANO | ovicaprini |
| 2012 | IT069ORXXX | 09/07/2012 | OR | ASL ORISTANO | ovicaprini |
| 2012 | IT010ORXXX | 10/07/2012 | OR | ASL ORISTANO | ovicaprini |
| 2012 | IT032CAXXX | 11/07/2012 | CA | ASL 8 CAGLIARI | ovicaprini |
| 2012 | IT046ORXXX | 13/07/2012 | OR | ASL ORISTANO | ovicaprini |
| 2012 | IT069ORXXX | 16/07/2012 | OR | ASL ORISTANO | ovicaprini |
| 2012 | IT001CAXXX | 16/07/2012 | CA | ASL 8 CAGLIARI | ovicaprini |
| 2012 | IT078CAXXX | 16/07/2012 | CA | ASL 8 CAGLIARI | ovicaprini |
| 2012 | IT029CAXXX | 16/07/2012 | CA | ASL 8 CAGLIARI | ovicaprini |
| 2012 | IT034NUXXX | 16/07/2012 | NU | ASL NUORO | ovicaprini |
| 2012 | IT012ORXXX | 17/07/2012 | OR | ASL ORISTANO | ovicaprini |
| 2012 | IT032CAXXX | 23/07/2012 | CA | ASL 8 CAGLIARI | ovicaprini |
| 2012 | IT032CAXXX | 23/07/2012 | CA | ASL 8 CAGLIARI | ovicaprini |
| 2012 | IT077ORXXX | 23/07/2012 | OR | ASL ORISTANO | ovicaprini |
| 2012 | IT001CAXXX | 23/07/2012 | CA | ASL 8 CAGLIARI | ovicaprini |
| 2012 | IT029CAXXX | 23/07/2012 | CA | ASL 8 CAGLIARI | ovicaprini |
| 2012 | IT046ORXXX | 24/07/2012 | OR | ASL ORISTANO | ovicaprini |
| 2012 | IT006CAXXX | 30/07/2012 | CA | ASL 8 CAGLIARI | ovicaprini |
| 2012 | IT029CAXXX | 30/07/2012 | CA | ASL 8 CAGLIARI | ovicaprini |
| 2012 | IT023CAXXX | 30/07/2012 | CA | ASL 8 CAGLIARI | ovicaprini |
| 2012 | IT032CAXXX | 01/08/2012 | CA | ASL 8 CAGLIARI | ovicaprini |
| 2012 | IT032CAXXX | 01/08/2012 | CA | ASL 8 CAGLIARI | ovicaprini |
| 2012 | IT023CAXXX | 01/08/2012 | CA | ASL 8 CAGLIARI | ovicaprini |
| 2012 | IT023CAXXX | 01/08/2012 | CA | ASL 8 CAGLIARI | ovicaprini |
| 2012 | IT044ORXXX | 03/08/2012 | OR | ASL ORISTANO | ovicaprini |
| 2012 | IT077CAXXX | 03/08/2012 | CA | ASL 8 CAGLIARI | ovicaprini |
| 2012 | IT086CAXXX | 03/08/2012 | CA | ASL 8 CAGLIARI | ovicaprini |
| 2012 | IT086CAXXX | 03/08/2012 | CA | ASL 8 CAGLIARI | ovicaprini |
| 2012 | IT052NUXXX | 06/08/2012 | NU | ASL NUORO | ovicaprini |
| 2012 | IT052NUXXX | 06/08/2012 | NU | ASL NUORO | ovicaprini |
| 2012 | IT055CAXXX | 06/08/2012 | CA | ASL 8 CAGLIARI | ovicaprini |
| 2012 | IT029CAXXX | 06/08/2012 | CA | ASL 8 CAGLIARI | ovicaprini |
| 2012 | IT042ORXXX | 07/08/2012 | OR | ASL ORISTANO | ovicaprini |
| 2012 | IT104CAXXX | 07/08/2012 | CA | ASL 8 CAGLIARI | ovicaprini |
| 2012 | IT039ORXXX | 09/08/2012 | OR | ASL ORISTANO | ovicaprini |
| 2012 | IT091CAXXX | 10/08/2012 | CA | ASL 8 CAGLIARI | ovicaprini |
| 2012 | IT087CAXXX | 17/08/2012 | CA | ASL 8 CAGLIARI | ovicaprini |
| 2012 | IT087CAXXX | 17/08/2012 | CA | ASL 8 CAGLIARI | ovicaprini |
| 2012 | IT087CAXXX | 17/08/2012 | CA | ASL 8 CAGLIARI | ovicaprini |
| 2012 | IT029ORXXX | 20/08/2012 | OR | ASL ORISTANO | ovicaprini |
| 2012 | IT060ORXXX | 20/08/2012 | OR | ASL ORISTANO | ovicaprini |
| 2012 | IT104CAXXX | 21/08/2012 | CA | ASL 8 CAGLIARI | ovicaprini |
| 2012 | IT032CAXXX | 22/08/2012 | CA | ASL 8 CAGLIARI | ovicaprini |
| 2012 | IT012ORXXX | 27/08/2012 | OR | ASL ORISTANO | ovicaprini |
| 2012 | IT030ORXXX | 27/08/2012 | OR | ASL ORISTANO | ovicaprini |
| 2012 | IT030ORXXX | 27/08/2012 | OR | ASL ORISTANO | ovicaprini |
| 2012 | IT032CAXXX | 05/09/2012 | CA | ASL 8 CAGLIARI | ovicaprini |
| 2012 | IT057CAXXX | 07/09/2012 | CA | ASL 8 CAGLIARI | ovicaprini |
| 2012 | IT035CAXXX | 07/09/2012 | CA | ASL 8 CAGLIARI | ovicaprini |
| 2012 | IT035CAXXX | 07/09/2012 | CA | ASL 8 CAGLIARI | ovicaprini |
| 2012 | IT012ORXXX | 12/09/2012 | OR | ASL ORISTANO | ovicaprini |
| 2012 | IT091CAXXX | 14/09/2012 | CA | ASL 8 CAGLIARI | ovicaprini |
| 2012 | IT029CAXXX | 24/09/2012 | CA | ASL 8 CAGLIARI | ovicaprini |
| 2012 | IT091CAXXX | 24/09/2012 | CA | ASL 8 CAGLIARI | ovicaprini |
| 2012 | IT096CAXXX | 02/07/2012 | CA | ASL 8 CAGLIARI | ovicaprini |
| 2012 | IT060CAXXX | 06/07/2012 | CA | ASL 8 CAGLIARI | ovicaprini |
| 2012 | IT096CAXXX | 09/07/2012 | CA | ASL 8 CAGLIARI | ovicaprini |
| 2012 | IT096CAXXX | 16/07/2012 | CA | ASL 8 CAGLIARI | ovicaprini |
| 2012 | IT078CAXXX | 23/07/2012 | CA | ASL 8 CAGLIARI | ovicaprini |
| 2012 | IT053CAXXX | 23/07/2012 | CA | ASL 8 CAGLIARI | ovicaprini |
| 2012 | IT060CAXXX | 26/07/2012 | CA | ASL 8 CAGLIARI | ovicaprini |
| 2012 | IT078CAXXX | 30/07/2012 | CA | ASL 8 CAGLIARI | ovicaprini |
| 2012 | IT069CAXXX | 06/08/2012 | CA | ASL 8 CAGLIARI | ovicaprini |
| 2012 | IT031CAXXX | 06/08/2012 | CA | ASL 8 CAGLIARI | ovicaprini |
| 2012 | IT078CAXXX | 06/08/2012 | CA | ASL 8 CAGLIARI | ovicaprini |
| 2012 | IT060CAXXX | 09/08/2012 | CA | ASL 8 CAGLIARI | ovicaprini |
| 2012 | IT078CAXXX | 20/08/2012 | CA | ASL 8 CAGLIARI | ovicaprini |
| 2012 | IT069CAXXX | 20/08/2012 | CA | ASL 8 CAGLIARI | ovicaprini |
| 2012 | IT069CAXXX | 27/08/2012 | CA | ASL 8 CAGLIARI | ovicaprini |
| 2012 | IT091CAXXX | 03/09/2012 | CA | ASL 8 CAGLIARI | ovicaprini |
| 2012 | IT091CAXXX | 10/09/2012 | CA | ASL 8 CAGLIARI | ovicaprini |
| 2012 | IT069CAXXX | 10/09/2012 | CA | ASL 8 CAGLIARI | ovicaprini |
| 2012 | IT086CAXXX | 03/10/2012 | CA | ASL 8 CAGLIARI | ovicaprini |
| 2012 | IT066CAXXX | 01/10/2012 | CA | ASL 8 CAGLIARI | ovicaprini |
| 2012 | IT093CAXXX | 01/10/2012 | CA | ASL 8 CAGLIARI | ovicaprini |
| 2012 | IT091CAXXX | 01/10/2012 | CA | ASL 8 CAGLIARI | ovicaprini |
| 2012 | IT066CAXXX | 04/10/2012 | CA | ASL 8 CAGLIARI | ovicaprini |
| 2012 | IT066CAXXX | 05/10/2012 | CA | ASL 8 CAGLIARI | ovicaprini |
| 2012 | IT033CAXXX | 08/10/2012 | CA | ASL 8 CAGLIARI | ovicaprini |
| 2012 | IT093CAXXX | 08/10/2012 | CA | ASL 8 CAGLIARI | ovicaprini |
| 2012 | IT032CAXXX | 08/10/2012 | CA | ASL 8 CAGLIARI | ovicaprini |
| 2012 | IT032CAXXX | 08/10/2012 | CA | ASL 8 CAGLIARI | ovicaprini |
| 2012 | IT093CAXXX | 09/10/2012 | CA | ASL 8 CAGLIARI | ovicaprini |
| 2012 | IT032CAXXX | 11/10/2012 | CA | ASL 8 CAGLIARI | ovicaprini |
| 2012 | IT092CAXXX | 12/10/2012 | CA | ASL 8 CAGLIARI | ovicaprini |
| 2012 | IT033CAXXX | 15/10/2012 | CA | ASL 8 CAGLIARI | ovicaprini |
| 2012 | IT069CAXXX | 15/10/2012 | CA | ASL 8 CAGLIARI | ovicaprini |
| 2012 | IT072CAXXX | 16/10/2012 | CA | ASL 8 CAGLIARI | ovicaprini |
| 2012 | IT033CAXXX | 16/10/2012 | CA | ASL 8 CAGLIARI | ovicaprini |
| 2012 | IT066CAXXX | 18/10/2012 | CA | ASL 8 CAGLIARI | ovicaprini |
| 2012 | IT066CAXXX | 19/10/2012 | CA | ASL 8 CAGLIARI | ovicaprini |
| 2012 | IT091CAXXX | 22/10/2012 | CA | ASL 8 CAGLIARI | ovicaprini |
| 2012 | IT072CAXXX | 25/10/2012 | CA | ASL 8 CAGLIARI | ovicaprini |
| 2012 | IT091CAXXX | 29/10/2012 | CA | ASL 8 CAGLIARI | ovicaprini |
| 2012 | IT077CAXXX | 02/11/2012 | CA | ASL 8 CAGLIARI | ovicaprini |
| 2012 | IT091CAXXX | 05/11/2012 | CA | ASL 8 CAGLIARI | ovicaprini |
| 2012 | IT072CAXXX | 08/11/2012 | CA | ASL 8 CAGLIARI | ovicaprini |
| 2012 | IT057ORXXX | 13/11/2012 | OR | ASL ORISTANO | ovicaprini |
| 2012 | IT057CAXXX | 15/11/2012 | CA | ASL 8 CAGLIARI | ovicaprini |
| 2012 | IT069CAXXX | 19/11/2012 | CA | ASL 8 CAGLIARI | ovicaprini |
| 2012 | IT072CAXXX | 20/11/2012 | CA | ASL 8 CAGLIARI | ovicaprini |
| 2012 | IT092CAXXX | 23/11/2012 | CA | ASL 8 CAGLIARI | ovicaprini |
| 2012 | IT072CAXXX | 26/11/2012 | CA | ASL 8 CAGLIARI | ovicaprini |
| 2012 | IT057CAXXX | 27/11/2012 | CA | ASL 8 CAGLIARI | ovicaprini |
| 2012 | IT032CAXXX | 04/12/2012 | CA | ASL 8 CAGLIARI | ovicaprini |
| 2012 | IT032CAXXX | 06/12/2012 | CA | ASL 8 CAGLIARI | ovicaprini |
| 2012 | IT029CAXXX | 14/12/2012 | CA | ASL 8 CAGLIARI | ovicaprini |
| 2012 | IT001CAXXX | 17/12/2012 | CA | ASL 8 CAGLIARI | ovicaprini |
| 2012 | IT032CAXXX | 17/12/2012 | CA | ASL 8 CAGLIARI | ovicaprini |
| 2012 | IT029CAXXX | 17/12/2012 | CA | ASL 8 CAGLIARI | ovicaprini |
| 2012 | IT072CAXXX | 18/12/2012 | CA | ASL 8 CAGLIARI | ovicaprini |
| 2012 | IT001CAXXX | 20/12/2012 | CA | ASL 8 CAGLIARI | ovicaprini |
| 2012 | IT032CAXXX | 20/12/2012 | CA | ASL 8 CAGLIARI | ovicaprini |
| 2012 | IT029CAXXX | 27/12/2012 | CA | ASL 8 CAGLIARI | ovicaprini |
| 2012 | IT072CAXXX | 31/12/2012 | CA | ASL 8 CAGLIARI | ovicaprini |
| 2012 | 077NU03XXX | 01/03/2012 | NU | ASL NUORO | ovicaprini |
| 2012 | 024NU03XXX | 04/03/2012 | NU | ASL NUORO | ovicaprini |
| 2012 | 046NU00XXX | 07/03/2012 | NU | ASL NUORO | ovicaprini |
| 2012 | 062NU20XXX | 10/03/2012 | NU | ASL NUORO | ovicaprini |
| 2012 | 061NU02XXX | 13/03/2012 | NU | ASL NUORO | ovicaprini |
| 2012 | 062NU02XXX | 16/03/2012 | NU | ASL NUORO | ovicaprini |
| 2012 | 077NU00XXX | 19/03/2012 | NU | ASL NUORO | ovicaprini |
| 2012 | 062NU45XXX | 22/03/2012 | NU | ASL NUORO | ovicaprini |
| 2012 | 062NU45XXX | 25/03/2012 | NU | ASL NUORO | ovicaprini |
| 2012 | 077NU01XXX | 28/03/2012 | NU | ASL NUORO | ovicaprini |
| 2012 | 061NU17XXX | 31/03/2012 | NU | ASL NUORO | ovicaprini |
| 2012 | 061NU12XXX | 03/04/2012 | NU | ASL NUORO | ovicaprini |
| 2012 | 061NU11XXX | 06/04/2012 | NU | ASL NUORO | ovicaprini |
| 2012 | 077NU03XXX | 09/04/2012 | NU | ASL NUORO | ovicaprini |
| 2012 | 024NU22XXX | 12/04/2012 | NU | ASL NUORO | ovicaprini |
| 2012 | 062NU20XXX | 15/04/2012 | NU | ASL NUORO | ovicaprini |
| 2012 | 002NU20XXX | 18/04/2012 | NU | ASL NUORO | ovicaprini |
| 2012 | 055NU04XXX | 21/04/2012 | NU | ASL NUORO | ovicaprini |
| 2012 | 062NU02XXX | 24/04/2012 | NU | ASL NUORO | ovicaprini |
| 2012 | 024NU24XXX | 27/04/2012 | NU | ASL NUORO | ovicaprini |
| 2012 | 077NU00XXX | 30/04/2012 | NU | ASL NUORO | ovicaprini |
| 2012 | 061NU14XXX | 03/05/2012 | NU | ASL NUORO | ovicaprini |
| 2012 | 077NU03XXX | 06/05/2012 | NU | ASL NUORO | ovicaprini |
| 2012 | 062NU13XXX | 09/05/2012 | NU | ASL NUORO | ovicaprini |
| 2012 | 061NU14XXX | 12/05/2012 | NU | ASL NUORO | ovicaprini |
| 2012 | 062NU20XXX | 15/05/2012 | NU | ASL NUORO | ovicaprini |
| 2012 | 012NU10XXX | 18/05/2012 | NU | ASL NUORO | ovicaprini |
| 2012 | 012NU12XXX | 21/05/2012 | NU | ASL NUORO | ovicaprini |
| 2012 | 049OR01XXX | 24/05/2012 | OR | ASL ORISTANO | ovicaprini |
| 2012 | 012NU10XXX | 27/05/2012 | NU | ASL NUORO | ovicaprini |
| 2012 | 046NU03XXX | 30/05/2012 | NU | ASL NUORO | ovicaprini |
| 2012 | 062NU45XXX | 02/06/2012 | NU | ASL NUORO | ovicaprini |
| 2012 | 046NU00XXX | 05/06/2012 | NU | ASL NUORO | ovicaprini |
| 2012 | 062NU39XXX | 08/06/2012 | NU | ASL NUORO | ovicaprini |
| 2012 | 083NU19XXX | 11/06/2012 | NU | ASL NUORO | ovicaprini |
| 2012 | 049OR01XXX | 14/06/2012 | OR | ASL ORISTANO | ovicaprini |
| 2012 | 012NU04XXX | 17/06/2012 | NU | ASL NUORO | ovicaprini |
| 2012 | 024NU23XXX | 20/06/2012 | NU | ASL NUORO | ovicaprini |
| 2012 | 061SS03XXX | 23/06/2012 | SS | ASL 1 SASSARI | ovicaprini |
| 2012 | 043NU14XXX | 26/06/2012 | NU | ASL NUORO | ovicaprini |
| 2012 | 045SS05XXX | 29/06/2012 | SS | ASL 1 SASSARI | ovicaprini |
| 2012 | 061NU07XXX | 02/07/2012 | NU | ASL NUORO | ovicaprini |
| 2012 | 067NU04XXX | 05/07/2012 | NU | ASL NUORO | ovicaprini |
| 2012 | 009NU09XXX | 08/07/2012 | NU | ASL NUORO | ovicaprini |
| 2012 | 026SS05XXX | 11/07/2012 | SS | ASL 1 SASSARI | ovicaprini |
| 2012 | 059NU02XXX | 14/07/2012 | NU | ASL NUORO | ovicaprini |
| 2012 | 009NU09XXX | 17/07/2012 | NU | ASL NUORO | ovicaprini |
| 2012 | 097NU02XXX | 20/07/2012 | NU | ASL NUORO | ovicaprini |
| 2012 | 043NU14XXX | 23/07/2012 | NU | ASL NUORO | ovicaprini |
| 2012 | 012SS00XXX | 26/07/2012 | SS | ASL 1 SASSARI | ovicaprini |
| 2012 | 009SS17XXX | 29/07/2012 | SS | ASL 1 SASSARI | ovicaprini |
| 2012 | 094NU14XXX | 01/08/2012 | NU | ASL NUORO | ovicaprini |
| 2012 | 085NU12XXX | 04/08/2012 | NU | ASL NUORO | ovicaprini |
| 2012 | 028NU02XXX | 07/08/2012 | NU | ASL NUORO | ovicaprini |
| 2012 | 040NU05XXX | 10/08/2012 | NU | ASL NUORO | ovicaprini |
| 2012 | 043NU02XXX | 13/08/2012 | NU | ASL NUORO | ovicaprini |
| 2012 | 043NU02XXX | 16/08/2012 | NU | ASL NUORO | ovicaprini |
| 2012 | 009NU09XXX | 19/08/2012 | NU | ASL NUORO | ovicaprini |
| 2012 | 041OR30XXX | 22/08/2012 | OR | ASL ORISTANO | ovicaprini |
| 2012 | 052OR09XXX | 25/08/2012 | OR | ASL ORISTANO | ovicaprini |
| 2013 | IT033CAXXX | 04/01/2013 | CA | ASL 8 CAGLIARI | ovicaprini |
| 2013 | IT029CAXXX | 11/01/2013 | CA | ASL 8 CAGLIARI | ovicaprini |
| 2013 | IT031CAXXX | 14/01/2013 | CA | ASL 8 CAGLIARI | ovicaprini |
| 2013 | IT096CAXXX | 14/01/2013 | CA | ASL 8 CAGLIARI | ovicaprini |
| 2013 | IT016CAXXX | 14/01/2013 | CA | ASL 8 CAGLIARI | ovicaprini |
| 2013 | IT032CAXXX | 15/01/2013 | CA | ASL 8 CAGLIARI | ovicaprini |
| 2013 | IT040ORXXX | 15/01/2013 | OR | ASL ORISTANO | ovicaprini |
| 2013 | IT040CAXXX | 15/01/2013 | CA | ASL 8 CAGLIARI | ovicaprini |
| 2013 | IT055CAXXX | 15/01/2013 | CA | ASL 8 CAGLIARI | ovicaprini |
| 2013 | IT016CAXXX | 17/01/2013 | CA | ASL 8 CAGLIARI | ovicaprini |
| 2013 | IT066CAXXX | 17/01/2013 | CA | ASL 8 CAGLIARI | ovicaprini |
| 2013 | IT066CAXXX | 18/01/2013 | CA | ASL 8 CAGLIARI | ovicaprini |
| 2013 | IT016CAXXX | 18/01/2013 | CA | ASL 8 CAGLIARI | ovicaprini |
| 2013 | IT047ORXXX | 18/01/2013 | OR | ASL ORISTANO | ovicaprini |
| 2013 | IT020CAXXX | 21/01/2013 | CA | ASL 8 CAGLIARI | ovicaprini |
| 2013 | IT066CAXXX | 21/01/2013 | CA | ASL 8 CAGLIARI | ovicaprini |
| 2013 | IT047ORXXX | 21/01/2013 | OR | ASL ORISTANO | ovicaprini |
| 2013 | IT055CAXXX | 21/01/2013 | CA | ASL 8 CAGLIARI | ovicaprini |
| 2013 | IT026ORXXX | 21/01/2013 | OR | ASL ORISTANO | ovicaprini |
| 2013 | IT032CAXXX | 21/01/2013 | CA | ASL 8 CAGLIARI | ovicaprini |
| 2013 | IT072CAXXX | 24/01/2013 | CA | ASL 8 CAGLIARI | ovicaprini |
| 2013 | IT077ORXXX | 25/01/2013 | OR | ASL ORISTANO | ovicaprini |
| 2013 | IT029CAXXX | 28/01/2013 | CA | ASL 8 CAGLIARI | ovicaprini |
| 2013 | IT092CAXXX | 28/01/2013 | CA | ASL 8 CAGLIARI | ovicaprini |
| 2013 | IT032CAXXX | 29/01/2013 | CA | ASL 8 CAGLIARI | ovicaprini |
| 2013 | IT057ORXXX | 29/01/2013 | OR | ASL ORISTANO | ovicaprini |
| 2013 | IT039ORXXX | 01/02/2013 | OR | ASL ORISTANO | ovicaprini |
| 2013 | IT020CAXXX | 04/02/2013 | CA | ASL 8 CAGLIARI | ovicaprini |
| 2013 | IT057CAXXX | 04/02/2013 | CA | ASL 8 CAGLIARI | ovicaprini |
| 2013 | IT086CAXXX | 04/02/2013 | CA | ASL 8 CAGLIARI | ovicaprini |
| 2013 | IT092CAXXX | 04/02/2013 | CA | ASL 8 CAGLIARI | ovicaprini |
| 2013 | IT057CAXXX | 05/02/2013 | CA | ASL 8 CAGLIARI | ovicaprini |
| 2013 | IT032CAXXX | 05/02/2013 | CA | ASL 8 CAGLIARI | ovicaprini |
| 2013 | IT044ORXXX | 05/02/2013 | OR | ASL ORISTANO | ovicaprini |
| 2013 | IT035CAXXX | 05/02/2013 | CA | ASL 8 CAGLIARI | ovicaprini |
| 2013 | IT086CAXXX | 05/02/2013 | CA | ASL 8 CAGLIARI | ovicaprini |
| 2013 | IT077ORXXX | 08/02/2013 | OR | ASL ORISTANO | ovicaprini |
| 2013 | IT034CAXXX | 08/02/2013 | CA | ASL 8 CAGLIARI | ovicaprini |
| 2013 | IT089CAXXX | 08/02/2013 | CA | ASL 8 CAGLIARI | ovicaprini |
| 2013 | IT092CAXXX | 11/02/2013 | CA | ASL 8 CAGLIARI | ovicaprini |
| 2013 | IT029CAXXX | 11/02/2013 | CA | ASL 8 CAGLIARI | ovicaprini |
| 2013 | IT057CAXXX | 11/02/2013 | CA | ASL 8 CAGLIARI | ovicaprini |
| 2013 | IT015ORXXX | 11/02/2013 | OR | ASL ORISTANO | ovicaprini |
| 2013 | IT032CAXXX | 11/02/2013 | CA | ASL 8 CAGLIARI | ovicaprini |
| 2013 | IT092CAXXX | 11/02/2013 | CA | ASL 8 CAGLIARI | ovicaprini |
| 2013 | IT072CAXXX | 12/02/2013 | CA | ASL 8 CAGLIARI | ovicaprini |
| 2013 | IT029CAXXX | 15/02/2013 | CA | ASL 8 CAGLIARI | ovicaprini |
| 2013 | IT078CAXXX | 15/02/2013 | CA | ASL 8 CAGLIARI | ovicaprini |
| 2013 | IT087CAXXX | 15/02/2013 | CA | ASL 8 CAGLIARI | ovicaprini |
| 2013 | IT029CAXXX | 15/02/2013 | CA | ASL 8 CAGLIARI | ovicaprini |
| 2013 | IT096CAXXX | 18/02/2013 | CA | ASL 8 CAGLIARI | ovicaprini |
| 2013 | IT025CAXXX | 18/02/2013 | CA | ASL 8 CAGLIARI | ovicaprini |
| 2013 | IT091CAXXX | 18/02/2013 | CA | ASL 8 CAGLIARI | ovicaprini |
| 2013 | IT029CAXXX | 18/02/2013 | CA | ASL 8 CAGLIARI | ovicaprini |
| 2013 | IT092CAXXX | 18/02/2013 | CA | ASL 8 CAGLIARI | ovicaprini |
| 2013 | IT078CAXXX | 19/02/2013 | CA | ASL 8 CAGLIARI | ovicaprini |
| 2013 | IT096CAXXX | 19/02/2013 | CA | ASL 8 CAGLIARI | ovicaprini |
| 2013 | IT076ORXXX | 22/02/2013 | OR | ASL ORISTANO | ovicaprini |
| 2013 | IT036NUXXX | 22/02/2013 | NU | ASL NUORO | ovicaprini |
| 2013 | IT077ORXXX | 22/02/2013 | OR | ASL ORISTANO | ovicaprini |
| 2013 | IT057ORXXX | 25/02/2013 | OR | ASL ORISTANO | ovicaprini |
| 2013 | IT101CAXXX | 25/02/2013 | CA | ASL 8 CAGLIARI | ovicaprini |
| 2013 | IT057CAXXX | 25/02/2013 | CA | ASL 8 CAGLIARI | ovicaprini |
| 2013 | IT076ORXXX | 25/02/2013 | OR | ASL ORISTANO | ovicaprini |
| 2013 | IT057ORXXX | 25/02/2013 | OR | ASL ORISTANO | ovicaprini |
| 2013 | IT029CAXXX | 25/02/2013 | CA | ASL 8 CAGLIARI | ovicaprini |
| 2013 | IT031CAXXX | 25/02/2013 | CA | ASL 8 CAGLIARI | ovicaprini |
| 2013 | IT046ORXXX | 27/02/2013 | OR | ASL ORISTANO | ovicaprini |
| 2013 | IT101CAXXX | 01/03/2013 | CA | ASL 8 CAGLIARI | ovicaprini |
| 2013 | IT101CAXXX | 01/03/2013 | CA | ASL 8 CAGLIARI | ovicaprini |
| 2013 | IT006CAXXX | 01/03/2013 | CA | ASL 8 CAGLIARI | ovicaprini |
| 2013 | IT006CAXXX | 01/03/2013 | CA | ASL 8 CAGLIARI | ovicaprini |
| 2013 | IT101CAXXX | 01/03/2013 | CA | ASL 8 CAGLIARI | ovicaprini |
| 2013 | IT016CAXXX | 04/03/2013 | CA | ASL 8 CAGLIARI | ovicaprini |
| 2013 | IT027ORXXX | 04/03/2013 | OR | ASL ORISTANO | ovicaprini |
| 2013 | IT029CAXXX | 04/03/2013 | CA | ASL 8 CAGLIARI | ovicaprini |
| 2013 | IT044ORXXX | 04/03/2013 | OR | ASL ORISTANO | ovicaprini |
| 2013 | IT092CAXXX | 04/03/2013 | CA | ASL 8 CAGLIARI | ovicaprini |
| 2013 | IT012ORXXX | 07/03/2013 | OR | ASL ORISTANO | ovicaprini |
| 2013 | IT078CAXXX | 08/03/2013 | CA | ASL 8 CAGLIARI | ovicaprini |
| 2013 | IT078CAXXX | 08/03/2013 | CA | ASL 8 CAGLIARI | ovicaprini |
| 2013 | IT010CAXXX | 08/03/2013 | CA | ASL 8 CAGLIARI | ovicaprini |
| 2013 | IT010CAXXX | 08/03/2013 | CA | ASL 8 CAGLIARI | ovicaprini |
| 2013 | IT086CAXXX | 08/03/2013 | CA | ASL 8 CAGLIARI | ovicaprini |
| 2013 | IT034CAXXX | 08/03/2013 | CA | ASL 8 CAGLIARI | ovicaprini |
| 2013 | IT093CAXXX | 08/03/2013 | CA | ASL 8 CAGLIARI | ovicaprini |
| 2013 | IT046CAXXX | 08/03/2013 | CA | ASL 8 CAGLIARI | ovicaprini |
| 2013 | IT046CAXXX | 08/03/2013 | CA | ASL 8 CAGLIARI | ovicaprini |
| 2013 | IT016CAXXX | 11/03/2013 | CA | ASL 8 CAGLIARI | ovicaprini |
| 2013 | IT046CAXXX | 11/03/2013 | CA | ASL 8 CAGLIARI | ovicaprini |
| 2013 | IT091CAXXX | 11/03/2013 | CA | ASL 8 CAGLIARI | ovicaprini |
| 2013 | IT032CAXXX | 11/03/2013 | CA | ASL 8 CAGLIARI | ovicaprini |
| 2013 | IT029CAXXX | 11/03/2013 | CA | ASL 8 CAGLIARI | ovicaprini |
| 2013 | IT001CAXXX | 11/03/2013 | CA | ASL 8 CAGLIARI | ovicaprini |
| 2013 | IT006CAXXX | 11/03/2013 | CA | ASL 8 CAGLIARI | ovicaprini |
| 2013 | IT006CAXXX | 11/03/2013 | CA | ASL 8 CAGLIARI | ovicaprini |
| 2013 | IT078CAXXX | 11/03/2013 | CA | ASL 8 CAGLIARI | ovicaprini |
| 2013 | IT031CAXXX | 11/03/2013 | CA | ASL 8 CAGLIARI | ovicaprini |
| 2013 | IT066CAXXX | 12/03/2013 | CA | ASL 8 CAGLIARI | ovicaprini |
| 2013 | IT086CAXXX | 15/03/2013 | CA | ASL 8 CAGLIARI | ovicaprini |
| 2013 | IT012CAXXX | 15/03/2013 | CA | ASL 8 CAGLIARI | ovicaprini |
| 2013 | IT077ORXXX | 15/03/2013 | OR | ASL ORISTANO | ovicaprini |
| 2013 | IT031ORXXX | 15/03/2013 | OR | ASL ORISTANO | ovicaprini |
| 2013 | IT023CAXXX | 15/03/2013 | CA | ASL 8 CAGLIARI | ovicaprini |
| 2013 | IT006CAXXX | 15/03/2013 | CA | ASL 8 CAGLIARI | ovicaprini |
| 2013 | IT032CAXXX | 18/03/2013 | CA | ASL 8 CAGLIARI | ovicaprini |
| 2013 | IT047CAXXX | 18/03/2013 | CA | ASL 8 CAGLIARI | ovicaprini |
| 2013 | IT029ORXXX | 18/03/2013 | OR | ASL ORISTANO | ovicaprini |
| 2013 | IT029CAXXX | 18/03/2013 | CA | ASL 8 CAGLIARI | ovicaprini |
| 2013 | IT029CAXXX | 18/03/2013 | CA | ASL 8 CAGLIARI | ovicaprini |
| 2013 | IT012ORXXX | 18/03/2013 | OR | ASL ORISTANO | ovicaprini |
| 2013 | IT086CAXXX | 18/03/2013 | CA | ASL 8 CAGLIARI | ovicaprini |
| 2013 | IT060CAXXX | 18/03/2013 | CA | ASL 8 CAGLIARI | ovicaprini |
| 2013 | IT060CAXXX | 18/03/2013 | CA | ASL 8 CAGLIARI | ovicaprini |
| 2013 | IT096CAXXX | 18/03/2013 | CA | ASL 8 CAGLIARI | ovicaprini |
| 2013 | IT096CAXXX | 18/03/2013 | CA | ASL 8 CAGLIARI | ovicaprini |
| 2013 | IT092CAXXX | 18/03/2013 | CA | ASL 8 CAGLIARI | ovicaprini |
| 2013 | IT057ORXXX | 19/03/2013 | OR | ASL ORISTANO | ovicaprini |
| 2013 | IT057CAXXX | 19/03/2013 | CA | ASL 8 CAGLIARI | ovicaprini |
| 2013 | IT094CAXXX | 20/03/2013 | CA | ASL 8 CAGLIARI | ovicaprini |
| 2013 | IT055CAXXX | 22/03/2013 | CA | ASL 8 CAGLIARI | ovicaprini |
| 2013 | IT096CAXXX | 22/03/2013 | CA | ASL 8 CAGLIARI | ovicaprini |
| 2013 | IT001CAXXX | 23/03/2013 | CA | ASL 8 CAGLIARI | ovicaprini |
| 2013 | IT057ORXXX | 26/03/2013 | OR | ASL ORISTANO | ovicaprini |
| 2013 | IT039ORXXX | 26/03/2013 | OR | ASL ORISTANO | ovicaprini |
| 2013 | IT096CAXXX | 04/04/2013 | CA | ASL 8 CAGLIARI | ovicaprini |
| 2013 | IT096CAXXX | 04/04/2013 | CA | ASL 8 CAGLIARI | ovicaprini |
| 2013 | IT072CAXXX | 05/04/2013 | CA | ASL 8 CAGLIARI | ovicaprini |
| 2013 | IT078CAXXX | 05/04/2013 | CA | ASL 8 CAGLIARI | ovicaprini |
| 2013 | IT086CAXXX | 08/04/2013 | CA | ASL 8 CAGLIARI | ovicaprini |
| 2013 | IT092CAXXX | 08/04/2013 | CA | ASL 8 CAGLIARI | ovicaprini |
| 2013 | IT029CAXXX | 08/04/2013 | CA | ASL 8 CAGLIARI | ovicaprini |
| 2013 | IT086CAXXX | 08/04/2013 | CA | ASL 8 CAGLIARI | ovicaprini |
| 2013 | IT029CAXXX | 08/04/2013 | CA | ASL 8 CAGLIARI | ovicaprini |
| 2013 | IT055CAXXX | 09/04/2013 | CA | ASL 8 CAGLIARI | ovicaprini |
| 2013 | IT057CAXXX | 10/04/2013 | CA | ASL 8 CAGLIARI | ovicaprini |
| 2013 | IT057CAXXX | 10/04/2013 | CA | ASL 8 CAGLIARI | ovicaprini |
| 2013 | IT102NUXXX | 12/04/2013 | NU | ASL NUORO | ovicaprini |
| 2013 | IT047ORXXX | 12/04/2013 | OR | ASL ORISTANO | ovicaprini |
| 2013 | IT027ORXXX | 12/04/2013 | OR | ASL ORISTANO | ovicaprini |
| 2013 | IT012CAXXX | 12/04/2013 | CA | ASL 8 CAGLIARI | ovicaprini |
| 2013 | IT033CAXXX | 12/04/2013 | CA | ASL 8 CAGLIARI | ovicaprini |
| 2013 | IT010CAXXX | 12/04/2013 | CA | ASL 8 CAGLIARI | ovicaprini |
| 2013 | IT087CAXXX | 12/04/2013 | CA | ASL 8 CAGLIARI | ovicaprini |
| 2013 | IT077CAXXX | 12/04/2013 | CA | ASL 8 CAGLIARI | ovicaprini |
| 2013 | IT032CAXXX | 12/04/2013 | CA | ASL 8 CAGLIARI | ovicaprini |
| 2013 | IT052NUXXX | 12/04/2013 | NU | ASL NUORO | ovicaprini |
| 2013 | IT086CAXXX | 12/04/2013 | CA | ASL 8 CAGLIARI | ovicaprini |
| 2013 | IT095CAXXX | 12/04/2013 | CA | ASL 8 CAGLIARI | ovicaprini |
| 2013 | IT078CAXXX | 12/04/2013 | CA | ASL 8 CAGLIARI | ovicaprini |
| 2013 | IT046CAXXX | 12/04/2013 | CA | ASL 8 CAGLIARI | ovicaprini |
| 2013 | IT070CAXXX | 12/04/2013 | CA | ASL 8 CAGLIARI | ovicaprini |
| 2013 | IT020CAXXX | 15/04/2013 | CA | ASL 8 CAGLIARI | ovicaprini |
| 2013 | IT092CAXXX | 15/04/2013 | CA | ASL 8 CAGLIARI | ovicaprini |
| 2013 | IT057CAXXX | 15/04/2013 | CA | ASL 8 CAGLIARI | ovicaprini |
| 2013 | IT028ORXXX | 15/04/2013 | OR | ASL ORISTANO | ovicaprini |
| 2013 | IT029CAXXX | 15/04/2013 | CA | ASL 8 CAGLIARI | ovicaprini |
| 2013 | IT032CAXXX | 15/04/2013 | CA | ASL 8 CAGLIARI | ovicaprini |
| 2013 | IT029CAXXX | 15/04/2013 | CA | ASL 8 CAGLIARI | ovicaprini |
| 2013 | IT029CAXXX | 15/04/2013 | CA | ASL 8 CAGLIARI | ovicaprini |
| 2013 | IT057ORXXX | 15/04/2013 | OR | ASL ORISTANO | ovicaprini |
| 2013 | IT029CAXXX | 16/04/2013 | CA | ASL 8 CAGLIARI | ovicaprini |
| 2013 | IT094CAXXX | 16/04/2013 | CA | ASL 8 CAGLIARI | ovicaprini |
| 2013 | IT094CAXXX | 16/04/2013 | CA | ASL 8 CAGLIARI | ovicaprini |
| 2013 | IT023CAXXX | 16/04/2013 | CA | ASL 8 CAGLIARI | ovicaprini |
| 2013 | IT023CAXXX | 16/04/2013 | CA | ASL 8 CAGLIARI | ovicaprini |
| 2013 | IT032CAXXX | 16/04/2013 | CA | ASL 8 CAGLIARI | ovicaprini |
| 2013 | IT057CAXXX | 16/04/2013 | CA | ASL 8 CAGLIARI | ovicaprini |
| 2013 | IT076ORXXX | 16/04/2013 | OR | ASL ORISTANO | ovicaprini |
| 2013 | IT029CAXXX | 17/04/2013 | CA | ASL 8 CAGLIARI | ovicaprini |
| 2013 | IT055CAXXX | 19/04/2013 | CA | ASL 8 CAGLIARI | ovicaprini |
| 2013 | IT086CAXXX | 19/04/2013 | CA | ASL 8 CAGLIARI | ovicaprini |
| 2013 | IT083CAXXX | 19/04/2013 | CA | ASL 8 CAGLIARI | ovicaprini |
| 2013 | IT083CAXXX | 19/04/2013 | CA | ASL 8 CAGLIARI | ovicaprini |
| 2013 | IT046CAXXX | 19/04/2013 | CA | ASL 8 CAGLIARI | ovicaprini |
| 2013 | IT065CAXXX | 19/04/2013 | CA | ASL 8 CAGLIARI | ovicaprini |
| 2013 | IT094CAXXX | 19/04/2013 | CA | ASL 8 CAGLIARI | ovicaprini |
| 2013 | IT094CAXXX | 19/04/2013 | CA | ASL 8 CAGLIARI | ovicaprini |
| 2013 | IT084CAXXX | 19/04/2013 | CA | ASL 8 CAGLIARI | ovicaprini |
| 2013 | IT060CAXXX | 19/04/2013 | CA | ASL 8 CAGLIARI | ovicaprini |
| 2013 | IT060CAXXX | 19/04/2013 | CA | ASL 8 CAGLIARI | ovicaprini |
| 2013 | IT057CAXXX | 22/04/2013 | CA | ASL 8 CAGLIARI | ovicaprini |
| 2013 | IT057CAXXX | 22/04/2013 | CA | ASL 8 CAGLIARI | ovicaprini |
| 2013 | IT031CAXXX | 22/04/2013 | CA | ASL 8 CAGLIARI | ovicaprini |
| 2013 | IT096CAXXX | 22/04/2013 | CA | ASL 8 CAGLIARI | ovicaprini |
| 2013 | IT045CAXXX | 22/04/2013 | CA | ASL 8 CAGLIARI | ovicaprini |
| 2013 | IT059ORXXX | 22/04/2013 | OR | ASL ORISTANO | ovicaprini |
| 2013 | IT078CAXXX | 22/04/2013 | CA | ASL 8 CAGLIARI | ovicaprini |
| 2013 | IT029ORXXX | 22/04/2013 | OR | ASL ORISTANO | ovicaprini |
| 2013 | IT092CAXXX | 22/04/2013 | CA | ASL 8 CAGLIARI | ovicaprini |
| 2013 | IT092CAXXX | 22/04/2013 | CA | ASL 8 CAGLIARI | ovicaprini |
| 2013 | IT048CAXXX | 22/04/2013 | CA | ASL 8 CAGLIARI | ovicaprini |
| 2013 | IT096CAXXX | 22/04/2013 | CA | ASL 8 CAGLIARI | ovicaprini |
| 2013 | IT033CAXXX | 23/04/2013 | CA | ASL 8 CAGLIARI | ovicaprini |
| 2013 | IT078CAXXX | 23/04/2013 | CA | ASL 8 CAGLIARI | ovicaprini |
| 2013 | IT096CAXXX | 23/04/2013 | CA | ASL 8 CAGLIARI | ovicaprini |
| 2013 | IT101CAXXX | 23/04/2013 | CA | ASL 8 CAGLIARI | ovicaprini |
| 2013 | IT057CAXXX | 24/04/2013 | CA | ASL 8 CAGLIARI | ovicaprini |
| 2013 | IT101CAXXX | 24/04/2013 | CA | ASL 8 CAGLIARI | ovicaprini |
| 2013 | IT034CAXXX | 24/04/2013 | CA | ASL 8 CAGLIARI | ovicaprini |
| 2013 | IT039ORXXX | 26/04/2013 | OR | ASL ORISTANO | ovicaprini |
| 2013 | IT022CAXXX | 26/04/2013 | CA | ASL 8 CAGLIARI | ovicaprini |
| 2013 | IT025CAXXX | 29/04/2013 | CA | ASL 8 CAGLIARI | ovicaprini |
| 2013 | IT087CAXXX | 29/04/2013 | CA | ASL 8 CAGLIARI | ovicaprini |
| 2013 | IT087CAXXX | 29/04/2013 | CA | ASL 8 CAGLIARI | ovicaprini |
| 2013 | IT045CAXXX | 29/04/2013 | CA | ASL 8 CAGLIARI | ovicaprini |
| 2013 | IT101CAXXX | 29/04/2013 | CA | ASL 8 CAGLIARI | ovicaprini |
| 2013 | IT092CAXXX | 29/04/2013 | CA | ASL 8 CAGLIARI | ovicaprini |
| 2013 | IT086CAXXX | 29/04/2013 | CA | ASL 8 CAGLIARI | ovicaprini |
| 2013 | IT031CAXXX | 29/04/2013 | CA | ASL 8 CAGLIARI | ovicaprini |
| 2013 | IT048CAXXX | 29/04/2013 | CA | ASL 8 CAGLIARI | ovicaprini |
| 2013 | IT092CAXXX | 29/04/2013 | CA | ASL 8 CAGLIARI | ovicaprini |
| 2013 | IT012ORXXX | 30/04/2013 | OR | ASL ORISTANO | ovicaprini |
| 2013 | IT057CAXXX | 30/04/2013 | CA | ASL 8 CAGLIARI | ovicaprini |
| 2013 | IT078CAXXX | 02/05/2013 | CA | ASL 8 CAGLIARI | ovicaprini |
| 2013 | IT078CAXXX | 03/05/2013 | CA | ASL 8 CAGLIARI | ovicaprini |
| 2013 | IT036NUXXX | 06/05/2013 | NU | ASL NUORO | ovicaprini |
| 2013 | IT055CAXXX | 06/05/2013 | CA | ASL 8 CAGLIARI | ovicaprini |
| 2013 | IT029CAXXX | 06/05/2013 | CA | ASL 8 CAGLIARI | ovicaprini |
| 2013 | IT029CAXXX | 06/05/2013 | CA | ASL 8 CAGLIARI | ovicaprini |
| 2013 | IT092CAXXX | 06/05/2013 | CA | ASL 8 CAGLIARI | ovicaprini |
| 2013 | IT012ORXXX | 06/05/2013 | OR | ASL ORISTANO | ovicaprini |
| 2013 | IT086CAXXX | 06/05/2013 | CA | ASL 8 CAGLIARI | ovicaprini |
| 2013 | IT092CAXXX | 06/05/2013 | CA | ASL 8 CAGLIARI | ovicaprini |
| 2013 | IT055CAXXX | 06/05/2013 | CA | ASL 8 CAGLIARI | ovicaprini |
| 2013 | IT086CAXXX | 06/05/2013 | CA | ASL 8 CAGLIARI | ovicaprini |
| 2013 | IT029CAXXX | 06/05/2013 | CA | ASL 8 CAGLIARI | ovicaprini |
| 2013 | IT029CAXXX | 06/05/2013 | CA | ASL 8 CAGLIARI | ovicaprini |
| 2013 | IT029CAXXX | 06/05/2013 | CA | ASL 8 CAGLIARI | ovicaprini |
| 2013 | IT031CAXXX | 06/05/2013 | CA | ASL 8 CAGLIARI | ovicaprini |
| 2013 | IT001CAXXX | 06/05/2013 | CA | ASL 8 CAGLIARI | ovicaprini |
| 2013 | IT029CAXXX | 06/05/2013 | CA | ASL 8 CAGLIARI | ovicaprini |
| 2013 | IT091CAXXX | 07/05/2013 | CA | ASL 8 CAGLIARI | ovicaprini |
| 2013 | IT086CAXXX | 07/05/2013 | CA | ASL 8 CAGLIARI | ovicaprini |
| 2013 | IT055CAXXX | 07/05/2013 | CA | ASL 8 CAGLIARI | ovicaprini |
| 2013 | IT001CAXXX | 08/05/2013 | CA | ASL 8 CAGLIARI | ovicaprini |
| 2013 | IT057CAXXX | 08/05/2013 | CA | ASL 8 CAGLIARI | ovicaprini |
| 2013 | IT057CAXXX | 08/05/2013 | CA | ASL 8 CAGLIARI | ovicaprini |
| 2013 | IT055CAXXX | 10/05/2013 | CA | ASL 8 CAGLIARI | ovicaprini |
| 2013 | IT037ORXXX | 10/05/2013 | OR | ASL ORISTANO | ovicaprini |
| 2013 | IT092CAXXX | 13/05/2013 | CA | ASL 8 CAGLIARI | ovicaprini |
| 2013 | IT001CAXXX | 13/05/2013 | CA | ASL 8 CAGLIARI | ovicaprini |
| 2013 | IT012CAXXX | 13/05/2013 | CA | ASL 8 CAGLIARI | ovicaprini |
| 2013 | IT029CAXXX | 13/05/2013 | CA | ASL 8 CAGLIARI | ovicaprini |
| 2013 | IT045CAXXX | 13/05/2013 | CA | ASL 8 CAGLIARI | ovicaprini |
| 2013 | IT012CAXXX | 13/05/2013 | CA | ASL 8 CAGLIARI | ovicaprini |
| 2013 | IT012ORXXX | 13/05/2013 | OR | ASL ORISTANO | ovicaprini |
| 2013 | IT101CAXXX | 13/05/2013 | CA | ASL 8 CAGLIARI | ovicaprini |
| 2013 | IT029ORXXX | 13/05/2013 | OR | ASL ORISTANO | ovicaprini |
| 2013 | IT031CAXXX | 13/05/2013 | CA | ASL 8 CAGLIARI | ovicaprini |
| 2013 | IT060CAXXX | 13/05/2013 | CA | ASL 8 CAGLIARI | ovicaprini |
| 2013 | IT060CAXXX | 13/05/2013 | CA | ASL 8 CAGLIARI | ovicaprini |
| 2013 | IT078CAXXX | 14/05/2013 | CA | ASL 8 CAGLIARI | ovicaprini |
| 2013 | IT101CAXXX | 14/05/2013 | CA | ASL 8 CAGLIARI | ovicaprini |
| 2013 | IT101CAXXX | 14/05/2013 | CA | ASL 8 CAGLIARI | ovicaprini |
| 2013 | IT101CAXXX | 14/05/2013 | CA | ASL 8 CAGLIARI | ovicaprini |
| 2013 | IT055CAXXX | 14/05/2013 | CA | ASL 8 CAGLIARI | ovicaprini |
| 2013 | IT055CAXXX | 14/05/2013 | CA | ASL 8 CAGLIARI | ovicaprini |
| 2013 | IT055CAXXX | 14/05/2013 | CA | ASL 8 CAGLIARI | ovicaprini |
| 2013 | IT040ORXXX | 14/05/2013 | OR | ASL ORISTANO | ovicaprini |
| 2013 | IT029CAXXX | 14/05/2013 | CA | ASL 8 CAGLIARI | ovicaprini |
| 2013 | IT055CAXXX | 14/05/2013 | CA | ASL 8 CAGLIARI | ovicaprini |
| 2013 | IT057CAXXX | 15/05/2013 | CA | ASL 8 CAGLIARI | ovicaprini |
| 2013 | IT029CAXXX | 15/05/2013 | CA | ASL 8 CAGLIARI | ovicaprini |
| 2013 | IT047ORXXX | 15/05/2013 | OR | ASL ORISTANO | ovicaprini |
| 2013 | IT072ORXXX | 15/05/2013 | OR | ASL ORISTANO | ovicaprini |
| 2013 | IT078CAXXX | 16/05/2013 | CA | ASL 8 CAGLIARI | ovicaprini |
| 2013 | IT078CAXXX | 17/05/2013 | CA | ASL 8 CAGLIARI | ovicaprini |
| 2013 | IT012ORXXX | 17/05/2013 | OR | ASL ORISTANO | ovicaprini |
| 2013 | IT033CAXXX | 17/05/2013 | CA | ASL 8 CAGLIARI | ovicaprini |
| 2013 | IT033CAXXX | 17/05/2013 | CA | ASL 8 CAGLIARI | ovicaprini |
| 2013 | IT031CAXXX | 20/05/2013 | CA | ASL 8 CAGLIARI | ovicaprini |
| 2013 | IT065CAXXX | 20/05/2013 | CA | ASL 8 CAGLIARI | ovicaprini |
| 2013 | IT012ORXXX | 20/05/2013 | OR | ASL ORISTANO | ovicaprini |
| 2013 | IT094CAXXX | 20/05/2013 | CA | ASL 8 CAGLIARI | ovicaprini |
| 2013 | IT092CAXXX | 20/05/2013 | CA | ASL 8 CAGLIARI | ovicaprini |
| 2013 | IT029ORXXX | 20/05/2013 | OR | ASL ORISTANO | ovicaprini |
| 2013 | IT057ORXXX | 20/05/2013 | OR | ASL ORISTANO | ovicaprini |
| 2013 | IT029CAXXX | 20/05/2013 | CA | ASL 8 CAGLIARI | ovicaprini |
| 2013 | IT042ORXXX | 20/05/2013 | OR | ASL ORISTANO | ovicaprini |
| 2013 | IT032CAXXX | 20/05/2013 | CA | ASL 8 CAGLIARI | ovicaprini |
| 2013 | IT101CAXXX | 20/05/2013 | CA | ASL 8 CAGLIARI | ovicaprini |
| 2013 | IT029ORXXX | 20/05/2013 | OR | ASL ORISTANO | ovicaprini |
| 2013 | IT094CAXXX | 21/05/2013 | CA | ASL 8 CAGLIARI | ovicaprini |
| 2013 | IT001CAXXX | 22/05/2013 | CA | ASL 8 CAGLIARI | ovicaprini |
| 2013 | IT032CAXXX | 23/05/2013 | CA | ASL 8 CAGLIARI | ovicaprini |
| 2013 | IT012CAXXX | 24/05/2013 | CA | ASL 8 CAGLIARI | ovicaprini |
| 2013 | IT025CAXXX | 27/05/2013 | CA | ASL 8 CAGLIARI | ovicaprini |
| 2013 | IT019CAXXX | 27/05/2013 | CA | ASL 8 CAGLIARI | ovicaprini |
| 2013 | IT016CAXXX | 27/05/2013 | CA | ASL 8 CAGLIARI | ovicaprini |
| 2013 | IT044ORXXX | 27/05/2013 | OR | ASL ORISTANO | ovicaprini |
| 2013 | IT052NUXXX | 27/05/2013 | NU | ASL NUORO | ovicaprini |
| 2013 | IT029CAXXX | 27/05/2013 | CA | ASL 8 CAGLIARI | ovicaprini |
| 2013 | IT046CAXXX | 27/05/2013 | CA | ASL 8 CAGLIARI | ovicaprini |
| 2013 | IT045CAXXX | 27/05/2013 | CA | ASL 8 CAGLIARI | ovicaprini |
| 2013 | IT029CAXXX | 27/05/2013 | CA | ASL 8 CAGLIARI | ovicaprini |
| 2013 | IT001CAXXX | 27/05/2013 | CA | ASL 8 CAGLIARI | ovicaprini |
| 2013 | IT053CAXXX | 27/05/2013 | CA | ASL 8 CAGLIARI | ovicaprini |
| 2013 | IT078CAXXX | 28/05/2013 | CA | ASL 8 CAGLIARI | ovicaprini |
| 2013 | IT010CAXXX | 29/05/2013 | CA | ASL 8 CAGLIARI | ovicaprini |
| 2013 | IT055CAXXX | 30/05/2013 | CA | ASL 8 CAGLIARI | ovicaprini |
| 2013 | IT078CAXXX | 31/05/2013 | CA | ASL 8 CAGLIARI | ovicaprini |
| 2013 | IT016CAXXX | 31/05/2013 | CA | ASL 8 CAGLIARI | ovicaprini |
| 2013 | IT030NUXXX | 03/06/2013 | NU | ASL NUORO | ovicaprini |
| 2013 | IT086CAXXX | 03/06/2013 | CA | ASL 8 CAGLIARI | ovicaprini |
| 2013 | IT032CAXXX | 03/06/2013 | CA | ASL 8 CAGLIARI | ovicaprini |
| 2013 | IT029CAXXX | 03/06/2013 | CA | ASL 8 CAGLIARI | ovicaprini |
| 2013 | IT045CAXXX | 03/06/2013 | CA | ASL 8 CAGLIARI | ovicaprini |
| 2013 | IT078CAXXX | 03/06/2013 | CA | ASL 8 CAGLIARI | ovicaprini |
| 2013 | IT092CAXXX | 03/06/2013 | CA | ASL 8 CAGLIARI | ovicaprini |
| 2013 | IT047ORXXX | 03/06/2013 | OR | ASL ORISTANO | ovicaprini |
| 2013 | IT036ORXXX | 03/06/2013 | OR | ASL ORISTANO | ovicaprini |
| 2013 | IT029ORXXX | 03/06/2013 | OR | ASL ORISTANO | ovicaprini |
| 2013 | IT046ORXXX | 03/06/2013 | OR | ASL ORISTANO | ovicaprini |
| 2013 | IT053CAXXX | 03/06/2013 | CA | ASL 8 CAGLIARI | ovicaprini |
| 2013 | IT012CAXXX | 04/06/2013 | CA | ASL 8 CAGLIARI | ovicaprini |
| 2013 | IT057CAXXX | 05/06/2013 | CA | ASL 8 CAGLIARI | ovicaprini |
| 2013 | IT029CAXXX | 05/06/2013 | CA | ASL 8 CAGLIARI | ovicaprini |
| 2013 | IT033CAXXX | 06/06/2013 | CA | ASL 8 CAGLIARI | ovicaprini |
| 2013 | IT032CAXXX | 06/06/2013 | CA | ASL 8 CAGLIARI | ovicaprini |
| 2013 | IT060CAXXX | 06/06/2013 | CA | ASL 8 CAGLIARI | ovicaprini |
| 2013 | IT091CAXXX | 10/06/2013 | CA | ASL 8 CAGLIARI | ovicaprini |
| 2013 | IT057CAXXX | 10/06/2013 | CA | ASL 8 CAGLIARI | ovicaprini |
| 2013 | IT087CAXXX | 10/06/2013 | CA | ASL 8 CAGLIARI | ovicaprini |
| 2013 | IT039ORXXX | 10/06/2013 | OR | ASL ORISTANO | ovicaprini |
| 2013 | IT091CAXXX | 10/06/2013 | CA | ASL 8 CAGLIARI | ovicaprini |
| 2013 | IT095CAXXX | 10/06/2013 | CA | ASL 8 CAGLIARI | ovicaprini |
| 2013 | IT045CAXXX | 10/06/2013 | CA | ASL 8 CAGLIARI | ovicaprini |
| 2013 | IT089CAXXX | 10/06/2013 | CA | ASL 8 CAGLIARI | ovicaprini |
| 2013 | IT092CAXXX | 10/06/2013 | CA | ASL 8 CAGLIARI | ovicaprini |
| 2013 | IT092CAXXX | 10/06/2013 | CA | ASL 8 CAGLIARI | ovicaprini |
| 2013 | IT001CAXXX | 10/06/2013 | CA | ASL 8 CAGLIARI | ovicaprini |
| 2013 | IT092CAXXX | 10/06/2013 | CA | ASL 8 CAGLIARI | ovicaprini |
| 2013 | IT031CAXXX | 10/06/2013 | CA | ASL 8 CAGLIARI | ovicaprini |
| 2013 | IT095CAXXX | 11/06/2013 | CA | ASL 8 CAGLIARI | ovicaprini |
| 2013 | IT052CAXXX | 13/06/2013 | CA | ASL 8 CAGLIARI | ovicaprini |
| 2013 | IT053NUXXX | 13/06/2013 | NU | ASL NUORO | ovicaprini |
| 2013 | IT072CAXXX | 13/06/2013 | CA | ASL 8 CAGLIARI | ovicaprini |
| 2013 | IT091CAXXX | 13/06/2013 | CA | ASL 8 CAGLIARI | ovicaprini |
| 2013 | IT072CAXXX | 14/06/2013 | CA | ASL 8 CAGLIARI | ovicaprini |
| 2013 | IT078CAXXX | 14/06/2013 | CA | ASL 8 CAGLIARI | ovicaprini |
| 2013 | IT016CAXXX | 14/06/2013 | CA | ASL 8 CAGLIARI | ovicaprini |
| 2013 | IT091CAXXX | 14/06/2013 | CA | ASL 8 CAGLIARI | ovicaprini |
| 2013 | IT030NUXXX | 17/06/2013 | NU | ASL NUORO | ovicaprini |
| 2013 | IT057CAXXX | 17/06/2013 | CA | ASL 8 CAGLIARI | ovicaprini |
| 2013 | IT012CAXXX | 17/06/2013 | CA | ASL 8 CAGLIARI | ovicaprini |
| 2013 | IT077ORXXX | 17/06/2013 | OR | ASL ORISTANO | ovicaprini |
| 2013 | IT001CAXXX | 17/06/2013 | CA | ASL 8 CAGLIARI | ovicaprini |
| 2013 | IT032CAXXX | 17/06/2013 | CA | ASL 8 CAGLIARI | ovicaprini |
| 2013 | IT057CAXXX | 17/06/2013 | CA | ASL 8 CAGLIARI | ovicaprini |
| 2013 | IT092CAXXX | 17/06/2013 | CA | ASL 8 CAGLIARI | ovicaprini |
| 2013 | IT045CAXXX | 17/06/2013 | CA | ASL 8 CAGLIARI | ovicaprini |
| 2013 | IT031CAXXX | 17/06/2013 | CA | ASL 8 CAGLIARI | ovicaprini |
| 2013 | IT029ORXXX | 18/06/2013 | OR | ASL ORISTANO | ovicaprini |
| 2013 | IT029CAXXX | 19/06/2013 | CA | ASL 8 CAGLIARI | ovicaprini |
| 2013 | IT033CAXXX | 20/06/2013 | CA | ASL 8 CAGLIARI | ovicaprini |
| 2013 | IT033CAXXX | 20/06/2013 | CA | ASL 8 CAGLIARI | ovicaprini |
| 2013 | IT019CAXXX | 24/06/2013 | CA | ASL 8 CAGLIARI | ovicaprini |
| 2013 | IT045CAXXX | 24/06/2013 | CA | ASL 8 CAGLIARI | ovicaprini |
| 2013 | IT006CAXXX | 24/06/2013 | CA | ASL 8 CAGLIARI | ovicaprini |
| 2013 | IT048ORXXX | 24/06/2013 | OR | ASL ORISTANO | ovicaprini |
| 2013 | IT077ORXXX | 24/06/2013 | OR | ASL ORISTANO | ovicaprini |
| 2013 | IT092CAXXX | 24/06/2013 | CA | ASL 8 CAGLIARI | ovicaprini |
| 2013 | IT033CAXXX | 25/06/2013 | CA | ASL 8 CAGLIARI | ovicaprini |
| 2013 | IT019CAXXX | 25/06/2013 | CA | ASL 8 CAGLIARI | ovicaprini |
| 2013 | IT012CAXXX | 27/06/2013 | CA | ASL 8 CAGLIARI | ovicaprini |
| 2013 | IT029CAXXX | 27/06/2013 | CA | ASL 8 CAGLIARI | ovicaprini |
| 2013 | IT060CAXXX | 27/06/2013 | CA | ASL 8 CAGLIARI | ovicaprini |
| 2013 | IT072CAXXX | 28/06/2013 | CA | ASL 8 CAGLIARI | ovicaprini |
| 2013 | IT078CAXXX | 28/06/2013 | CA | ASL 8 CAGLIARI | ovicaprini |
| 2013 | IT034NUXXX | 01/07/2013 | NU | ASL NUORO | ovicaprini |
| 2013 | IT030NUXXX | 01/07/2013 | NU | ASL NUORO | ovicaprini |
| 2013 | IT048ORXXX | 01/07/2013 | OR | ASL ORISTANO | ovicaprini |
| 2013 | IT045CAXXX | 01/07/2013 | CA | ASL 8 CAGLIARI | ovicaprini |
| 2013 | IT045CAXXX | 01/07/2013 | CA | ASL 8 CAGLIARI | ovicaprini |
| 2013 | IT045CAXXX | 01/07/2013 | CA | ASL 8 CAGLIARI | ovicaprini |
| 2013 | IT048ORXXX | 01/07/2013 | OR | ASL ORISTANO | ovicaprini |
| 2013 | IT029CAXXX | 01/07/2013 | CA | ASL 8 CAGLIARI | ovicaprini |
| 2013 | IT070CAXXX | 01/07/2013 | CA | ASL 8 CAGLIARI | ovicaprini |
| 2013 | IT033CAXXX | 02/07/2013 | CA | ASL 8 CAGLIARI | ovicaprini |
| 2013 | IT033CAXXX | 04/07/2013 | CA | ASL 8 CAGLIARI | ovicaprini |
| 2013 | IT033CAXXX | 04/07/2013 | CA | ASL 8 CAGLIARI | ovicaprini |
| 2013 | IT031CAXXX | 08/07/2013 | CA | ASL 8 CAGLIARI | ovicaprini |
| 2013 | IT065CAXXX | 08/07/2013 | CA | ASL 8 CAGLIARI | ovicaprini |
| 2013 | IT065CAXXX | 08/07/2013 | CA | ASL 8 CAGLIARI | ovicaprini |
| 2013 | IT033CAXXX | 08/07/2013 | CA | ASL 8 CAGLIARI | ovicaprini |
| 2013 | IT065CAXXX | 08/07/2013 | CA | ASL 8 CAGLIARI | ovicaprini |
| 2013 | IT069ORXXX | 08/07/2013 | OR | ASL ORISTANO | ovicaprini |
| 2013 | IT092CAXXX | 08/07/2013 | CA | ASL 8 CAGLIARI | ovicaprini |
| 2013 | IT091CAXXX | 08/07/2013 | CA | ASL 8 CAGLIARI | ovicaprini |
| 2013 | IT032CAXXX | 08/07/2013 | CA | ASL 8 CAGLIARI | ovicaprini |
| 2013 | IT035CAXXX | 08/07/2013 | CA | ASL 8 CAGLIARI | ovicaprini |
| 2013 | IT095CAXXX | 08/07/2013 | CA | ASL 8 CAGLIARI | ovicaprini |
| 2013 | IT092CAXXX | 08/07/2013 | CA | ASL 8 CAGLIARI | ovicaprini |
| 2013 | IT033CAXXX | 09/07/2013 | CA | ASL 8 CAGLIARI | ovicaprini |
| 2013 | IT080NUXXX | 10/07/2013 | NU | ASL NUORO | ovicaprini |
| 2013 | IT057CAXXX | 10/07/2013 | CA | ASL 8 CAGLIARI | ovicaprini |
| 2013 | IT012ORXXX | 11/07/2013 | OR | ASL ORISTANO | ovicaprini |
| 2013 | IT035CAXXX | 15/07/2013 | CA | ASL 8 CAGLIARI | ovicaprini |
| 2013 | IT057CAXXX | 15/07/2013 | CA | ASL 8 CAGLIARI | ovicaprini |
| 2013 | IT021CAXXX | 15/07/2013 | CA | ASL 8 CAGLIARI | ovicaprini |
| 2013 | IT091CAXXX | 15/07/2013 | CA | ASL 8 CAGLIARI | ovicaprini |
| 2013 | IT032CAXXX | 15/07/2013 | CA | ASL 8 CAGLIARI | ovicaprini |
| 2013 | IT001CAXXX | 15/07/2013 | CA | ASL 8 CAGLIARI | ovicaprini |
| 2013 | IT035CAXXX | 15/07/2013 | CA | ASL 8 CAGLIARI | ovicaprini |
| 2013 | IT077CAXXX | 15/07/2013 | CA | ASL 8 CAGLIARI | ovicaprini |
| 2013 | IT077CAXXX | 15/07/2013 | CA | ASL 8 CAGLIARI | ovicaprini |
| 2013 | IT034CAXXX | 15/07/2013 | CA | ASL 8 CAGLIARI | ovicaprini |
| 2013 | IT035CAXXX | 15/07/2013 | CA | ASL 8 CAGLIARI | ovicaprini |
| 2013 | IT086CAXXX | 15/07/2013 | CA | ASL 8 CAGLIARI | ovicaprini |
| 2013 | IT086CAXXX | 15/07/2013 | CA | ASL 8 CAGLIARI | ovicaprini |
| 2013 | IT086CAXXX | 15/07/2013 | CA | ASL 8 CAGLIARI | ovicaprini |
| 2013 | IT021CAXXX | 16/07/2013 | CA | ASL 8 CAGLIARI | ovicaprini |
| 2013 | IT091CAXXX | 16/07/2013 | CA | ASL 8 CAGLIARI | ovicaprini |
| 2013 | IT029CAXXX | 17/07/2013 | CA | ASL 8 CAGLIARI | ovicaprini |
| 2013 | IT066CAXXX | 18/07/2013 | CA | ASL 8 CAGLIARI | ovicaprini |
| 2013 | IT066CAXXX | 19/07/2013 | CA | ASL 8 CAGLIARI | ovicaprini |
| 2013 | IT031CAXXX | 22/07/2013 | CA | ASL 8 CAGLIARI | ovicaprini |
| 2013 | IT001CAXXX | 22/07/2013 | CA | ASL 8 CAGLIARI | ovicaprini |
| 2013 | IT096CAXXX | 22/07/2013 | CA | ASL 8 CAGLIARI | ovicaprini |
| 2013 | IT035CAXXX | 22/07/2013 | CA | ASL 8 CAGLIARI | ovicaprini |
| 2013 | IT057ORXXX | 22/07/2013 | OR | ASL ORISTANO | ovicaprini |
| 2013 | IT029ORXXX | 22/07/2013 | OR | ASL ORISTANO | ovicaprini |
| 2013 | IT035CAXXX | 22/07/2013 | CA | ASL 8 CAGLIARI | ovicaprini |
| 2013 | IT035CAXXX | 22/07/2013 | CA | ASL 8 CAGLIARI | ovicaprini |
| 2013 | IT086CAXXX | 22/07/2013 | CA | ASL 8 CAGLIARI | ovicaprini |
| 2013 | IT039ORXXX | 22/07/2013 | OR | ASL ORISTANO | ovicaprini |
| 2013 | IT072CAXXX | 23/07/2013 | CA | ASL 8 CAGLIARI | ovicaprini |
| 2013 | IT019CAXXX | 23/07/2013 | CA | ASL 8 CAGLIARI | ovicaprini |
| 2013 | IT020CAXXX | 23/07/2013 | CA | ASL 8 CAGLIARI | ovicaprini |
| 2013 | IT020CAXXX | 23/07/2013 | CA | ASL 8 CAGLIARI | ovicaprini |
| 2013 | IT094CAXXX | 23/07/2013 | CA | ASL 8 CAGLIARI | ovicaprini |
| 2013 | IT053NUXXX | 24/07/2013 | NU | ASL NUORO | ovicaprini |
| 2013 | IT036CAXXX | 24/07/2013 | CA | ASL 8 CAGLIARI | ovicaprini |
| 2013 | IT057CAXXX | 24/07/2013 | CA | ASL 8 CAGLIARI | ovicaprini |
| 2013 | IT029CAXXX | 24/07/2013 | CA | ASL 8 CAGLIARI | ovicaprini |
| 2013 | IT052CAXXX | 25/07/2013 | CA | ASL 8 CAGLIARI | ovicaprini |
| 2013 | IT072CAXXX | 25/07/2013 | CA | ASL 8 CAGLIARI | ovicaprini |
| 2013 | IT072CAXXX | 25/07/2013 | CA | ASL 8 CAGLIARI | ovicaprini |
| 2013 | IT072CAXXX | 25/07/2013 | CA | ASL 8 CAGLIARI | ovicaprini |
| 2013 | IT020CAXXX | 25/07/2013 | CA | ASL 8 CAGLIARI | ovicaprini |
| 2013 | IT065CAXXX | 29/07/2013 | CA | ASL 8 CAGLIARI | ovicaprini |
| 2013 | IT035CAXXX | 29/07/2013 | CA | ASL 8 CAGLIARI | ovicaprini |
| 2013 | IT086CAXXX | 29/07/2013 | CA | ASL 8 CAGLIARI | ovicaprini |
| 2013 | IT029ORXXX | 29/07/2013 | OR | ASL ORISTANO | ovicaprini |
| 2013 | IT087CAXXX | 29/07/2013 | CA | ASL 8 CAGLIARI | ovicaprini |
| 2013 | IT069ORXXX | 29/07/2013 | OR | ASL ORISTANO | ovicaprini |
| 2013 | IT091CAXXX | 02/08/2013 | CA | ASL 8 CAGLIARI | ovicaprini |
| 2013 | IT091CAXXX | 05/08/2013 | CA | ASL 8 CAGLIARI | ovicaprini |
| 2013 | IT069ORXXX | 05/08/2013 | OR | ASL ORISTANO | ovicaprini |
| 2013 | IT077CAXXX | 05/08/2013 | CA | ASL 8 CAGLIARI | ovicaprini |
| 2013 | IT090CAXXX | 05/08/2013 | CA | ASL 8 CAGLIARI | ovicaprini |
| 2013 | IT035CAXXX | 05/08/2013 | CA | ASL 8 CAGLIARI | ovicaprini |
| 2013 | IT012CAXXX | 06/08/2013 | CA | ASL 8 CAGLIARI | ovicaprini |
| 2013 | IT035CAXXX | 07/08/2013 | CA | ASL 8 CAGLIARI | ovicaprini |
| 2013 | IT077ORXXX | 07/08/2013 | OR | ASL ORISTANO | ovicaprini |
| 2013 | IT016CAXXX | 08/08/2013 | CA | ASL 8 CAGLIARI | ovicaprini |
| 2013 | IT057ORXXX | 08/08/2013 | OR | ASL ORISTANO | ovicaprini |
| 2013 | IT036CAXXX | 09/08/2013 | CA | ASL 8 CAGLIARI | ovicaprini |
| 2013 | IT029CAXXX | 09/08/2013 | CA | ASL 8 CAGLIARI | ovicaprini |
| 2013 | IT039ORXXX | 09/08/2013 | OR | ASL ORISTANO | ovicaprini |
| 2013 | IT028CAXXX | 12/08/2013 | CA | ASL 8 CAGLIARI | ovicaprini |
| 2013 | IT028CAXXX | 12/08/2013 | CA | ASL 8 CAGLIARI | ovicaprini |
| 2013 | IT092CAXXX | 12/08/2013 | CA | ASL 8 CAGLIARI | ovicaprini |
| 2013 | IT092CAXXX | 12/08/2013 | CA | ASL 8 CAGLIARI | ovicaprini |
| 2013 | IT036CAXXX | 13/08/2013 | CA | ASL 8 CAGLIARI | ovicaprini |
| 2013 | IT036CAXXX | 13/08/2013 | CA | ASL 8 CAGLIARI | ovicaprini |
| 2013 | IT036CAXXX | 16/08/2013 | CA | ASL 8 CAGLIARI | ovicaprini |
| 2013 | IT001CAXXX | 19/08/2013 | CA | ASL 8 CAGLIARI | ovicaprini |
| 2013 | IT036CAXXX | 19/08/2013 | CA | ASL 8 CAGLIARI | ovicaprini |
| 2013 | IT035CAXXX | 19/08/2013 | CA | ASL 8 CAGLIARI | ovicaprini |
| 2013 | IT086CAXXX | 19/08/2013 | CA | ASL 8 CAGLIARI | ovicaprini |
| 2013 | IT101CAXXX | 19/08/2013 | CA | ASL 8 CAGLIARI | ovicaprini |
| 2013 | IT019CAXXX | 20/08/2013 | CA | ASL 8 CAGLIARI | ovicaprini |
| 2013 | IT012ORXXX | 21/08/2013 | OR | ASL ORISTANO | ovicaprini |
| 2013 | IT096CAXXX | 23/08/2013 | CA | ASL 8 CAGLIARI | ovicaprini |
| 2013 | IT096CAXXX | 23/08/2013 | CA | ASL 8 CAGLIARI | ovicaprini |
| 2013 | IT031CAXXX | 23/08/2013 | CA | ASL 8 CAGLIARI | ovicaprini |
| 2013 | IT046CAXXX | 26/08/2013 | CA | ASL 8 CAGLIARI | ovicaprini |
| 2013 | IT046CAXXX | 26/08/2013 | CA | ASL 8 CAGLIARI | ovicaprini |
| 2013 | IT046CAXXX | 26/08/2013 | CA | ASL 8 CAGLIARI | ovicaprini |
| 2013 | IT046CAXXX | 26/08/2013 | CA | ASL 8 CAGLIARI | ovicaprini |
| 2013 | IT046CAXXX | 26/08/2013 | CA | ASL 8 CAGLIARI | ovicaprini |
| 2013 | IT053CAXXX | 26/08/2013 | CA | ASL 8 CAGLIARI | ovicaprini |
| 2013 | IT031CAXXX | 26/08/2013 | CA | ASL 8 CAGLIARI | ovicaprini |
| 2013 | IT096CAXXX | 26/08/2013 | CA | ASL 8 CAGLIARI | ovicaprini |
| 2013 | IT035CAXXX | 26/08/2013 | CA | ASL 8 CAGLIARI | ovicaprini |
| 2013 | IT055CAXXX | 26/08/2013 | CA | ASL 8 CAGLIARI | ovicaprini |
| 2013 | IT042ORXXX | 26/08/2013 | OR | ASL ORISTANO | ovicaprini |
| 2013 | IT092CAXXX | 26/08/2013 | CA | ASL 8 CAGLIARI | ovicaprini |
| 2013 | IT057CAXXX | 28/08/2013 | CA | ASL 8 CAGLIARI | ovicaprini |
| 2013 | IT086CAXXX | 30/08/2013 | CA | ASL 8 CAGLIARI | ovicaprini |
| 2013 | IT096CAXXX | 02/09/2013 | CA | ASL 8 CAGLIARI | ovicaprini |
| 2013 | IT035CAXXX | 02/09/2013 | CA | ASL 8 CAGLIARI | ovicaprini |
| 2013 | IT029ORXXX | 02/09/2013 | OR | ASL ORISTANO | ovicaprini |
| 2013 | IT087CAXXX | 05/09/2013 | CA | ASL 8 CAGLIARI | ovicaprini |
| 2013 | IT091CAXXX | 09/09/2013 | CA | ASL 8 CAGLIARI | ovicaprini |
| 2013 | IT091CAXXX | 09/09/2013 | CA | ASL 8 CAGLIARI | ovicaprini |
| 2013 | IT035CAXXX | 09/09/2013 | CA | ASL 8 CAGLIARI | ovicaprini |
| 2013 | IT010ORXXX | 09/09/2013 | OR | ASL ORISTANO | ovicaprini |
| 2013 | IT087CAXXX | 12/09/2013 | CA | ASL 8 CAGLIARI | ovicaprini |
| 2013 | IT087CAXXX | 16/09/2013 | CA | ASL 8 CAGLIARI | ovicaprini |
| 2013 | IT057CAXXX | 16/09/2013 | CA | ASL 8 CAGLIARI | ovicaprini |
| 2013 | IT092CAXXX | 12/08/2013 | CA | ASL 8 CAGLIARI | ovicaprini |
| 2013 | IT092CAXXX | 12/08/2013 | CA | ASL 8 CAGLIARI | ovicaprini |
| 2013 | IT096CAXXX | 23/08/2013 | CA | ASL 8 CAGLIARI | ovicaprini |
| 2013 | IT096CAXXX | 23/08/2013 | CA | ASL 8 CAGLIARI | ovicaprini |
| 2013 | IT031CAXXX | 23/08/2013 | CA | ASL 8 CAGLIARI | ovicaprini |
| 2013 | IT086CAXXX | 02/10/2013 | CA | ASL 8 CAGLIARI | ovicaprini |
| 2013 | IT092CAXXX | 15/11/2013 | CA | ASL 8 CAGLIARI | ovicaprini |
| 2013 | IT092CAXXX | 29/11/2013 | CA | ASL 8 CAGLIARI | ovicaprini |
| 2013 | IT072CAXXX | 17/12/2013 | CA | ASL 8 CAGLIARI | ovicaprini |
| 2013 | IT028ORXXX | 30/12/2013 | OR | ASL ORISTANO | ovicaprini |
| 2013 | IT070ORXXX | 30/12/2013 | OR | ASL ORISTANO | ovicaprini |
| 2013 | IT086CAXXX | 14/10/2013 | CA | ASL 8 CAGLIARI | ovicaprini |
| 2013 | IT045CAXXX | 04/11/2013 | CA | ASL 8 CAGLIARI | ovicaprini |
| 2013 | 033OR01XXX | 02/01/2013 | OR | ASL ORISTANO | ovicaprini |
| 2013 | 052OR08XXX | 02/01/2013 | OR | ASL ORISTANO | ovicaprini |
| 2013 | 049OR12XXX | 07/01/2013 | OR | ASL ORISTANO | ovicaprini |
| 2013 | 052OR04XXX | 07/01/2013 | OR | ASL ORISTANO | ovicaprini |
| 2013 | 044NU04XXX | 07/01/2013 | NU | ASL NUORO | ovicaprini |
| 2013 | 021OR01XXX | 10/01/2013 | OR | ASL ORISTANO | ovicaprini |
| 2013 | 045SS07XXX | 14/01/2013 | SS | ASL 1 SASSARI | ovicaprini |
| 2013 | 025OR18XXX | 14/01/2013 | OR | ASL ORISTANO | ovicaprini |
| 2013 | 083NU02XXX | 14/01/2013 | NU | ASL NUORO | ovicaprini |
| 2013 | 063OR04XXX | 14/01/2013 | OR | ASL ORISTANO | ovicaprini |
| 2013 | 056NU03XXX | 21/01/2013 | NU | ASL NUORO | ovicaprini |
| 2013 | 070NU03XXX | 21/01/2013 | NU | ASL NUORO | ovicaprini |
| 2013 | 011SS06XXX | 24/01/2013 | SS | ASL 1 SASSARI | ovicaprini |
| 2013 | 021OR02XXX | 24/01/2013 | OR | ASL ORISTANO | ovicaprini |
| 2013 | 011SS02XXX | 24/01/2013 | SS | ASL 1 SASSARI | ovicaprini |
| 2013 | 030SS04XXX | 24/01/2013 | SS | ASL 1 SASSARI | ovicaprini |
| 2013 | 050SS09XXX | 28/01/2013 | SS | ASL 1 SASSARI | ovicaprini |
| 2013 | 050SS10XXX | 28/01/2013 | SS | ASL 1 SASSARI | ovicaprini |
| 2013 | 013SS07XXX | 28/01/2013 | SS | ASL 1 SASSARI | ovicaprini |
| 2013 | 021OR02XXX | 28/01/2013 | OR | ASL ORISTANO | ovicaprini |
| 2013 | 083NU01XXX | 28/01/2013 | NU | ASL NUORO | ovicaprini |
| 2013 | 027SS02XXX | 29/01/2013 | SS | ASL 1 SASSARI | ovicaprini |
| 2013 | 049OR13XXX | 29/01/2013 | OR | ASL ORISTANO | ovicaprini |
| 2013 | 050SS04XXX | 29/01/2013 | SS | ASL 1 SASSARI | ovicaprini |
| 2013 | 094CA01XXX | 31/01/2013 | CA | ASL 8 CAGLIARI | ovicaprini |
| 2013 | 044OR09XXX | 31/01/2013 | OR | ASL ORISTANO | ovicaprini |
| 2013 | 029CA01XXX | 04/02/2013 | CA | ASL 8 CAGLIARI | ovicaprini |
| 2013 | 030SS04XXX | 04/02/2013 | SS | ASL 1 SASSARI | ovicaprini |
| 2013 | 030SS01XXX | 04/02/2013 | SS | ASL 1 SASSARI | ovicaprini |
| 2013 | 044NU04XXX | 04/02/2013 | NU | ASL NUORO | ovicaprini |
| 2013 | 064SS23XXX | 05/02/2013 | SS | ASL 1 SASSARI | ovicaprini |
| 2013 | 029CA06XXX | 05/02/2013 | CA | ASL 8 CAGLIARI | ovicaprini |
| 2013 | 029CA06XXX | 05/02/2013 | CA | ASL 8 CAGLIARI | ovicaprini |
| 2013 | 032CA10XXX | 05/02/2013 | CA | ASL 8 CAGLIARI | ovicaprini |
| 2013 | 008NU01XXX | 05/02/2013 | NU | ASL NUORO | ovicaprini |
| 2013 | 030SS05XXX | 05/02/2013 | SS | ASL 1 SASSARI | ovicaprini |
| 2013 | 008NU01XXX | 05/02/2013 | NU | ASL NUORO | ovicaprini |
| 2013 | 020OR09XXX | 07/02/2013 | OR | ASL ORISTANO | ovicaprini |
| 2013 | 050SS11XXX | 08/02/2013 | SS | ASL 1 SASSARI | ovicaprini |
| 2013 | 030SS01XXX | 08/02/2013 | SS | ASL 1 SASSARI | ovicaprini |
| 2013 | 083NU03XXX | 08/02/2013 | NU | ASL NUORO | ovicaprini |
| 2013 | 016CA01XXX | 11/02/2013 | CA | ASL 8 CAGLIARI | ovicaprini |
| 2013 | 016CA01XXX | 11/02/2013 | CA | ASL 8 CAGLIARI | ovicaprini |
| 2013 | 016CA01XXX | 11/02/2013 | CA | ASL 8 CAGLIARI | ovicaprini |
| 2013 | 057SS04XXX | 11/02/2013 | SS | ASL 1 SASSARI | ovicaprini |
| 2013 | 001OR07XXX | 12/02/2013 | OR | ASL ORISTANO | ovicaprini |
| 2013 | 049OR08XXX | 12/02/2013 | OR | ASL ORISTANO | ovicaprini |
| 2013 | 012NU14XXX | 14/02/2013 | NU | ASL NUORO | ovicaprini |
| 2013 | 010NU00XXX | 14/02/2013 | NU | ASL NUORO | ovicaprini |
| 2013 | 063OR04XXX | 15/02/2013 | OR | ASL ORISTANO | ovicaprini |
| 2013 | 033OR05XXX | 15/02/2013 | OR | ASL ORISTANO | ovicaprini |
| 2013 | 033SS14XXX | 18/02/2013 | SS | ASL 1 SASSARI | ovicaprini |
| 2013 | 033SS00XXX | 18/02/2013 | SS | ASL 1 SASSARI | ovicaprini |
| 2013 | 077NU02XXX | 18/02/2013 | NU | ASL NUORO | ovicaprini |
| 2013 | 033SS01XXX | 18/02/2013 | SS | ASL 1 SASSARI | ovicaprini |
| 2013 | 063OR04XXX | 18/02/2013 | OR | ASL ORISTANO | ovicaprini |
| 2013 | 033SS00XXX | 18/02/2013 | SS | ASL 1 SASSARI | ovicaprini |
| 2013 | 077NU02XXX | 18/02/2013 | NU | ASL NUORO | ovicaprini |
| 2013 | 033SS01XXX | 18/02/2013 | SS | ASL 1 SASSARI | ovicaprini |
| 2013 | 063OR04XXX | 18/02/2013 | OR | ASL ORISTANO | ovicaprini |
| 2013 | 012NU05XXX | 18/02/2013 | NU | ASL NUORO | ovicaprini |
| 2013 | 047OR12XXX | 18/02/2013 | OR | ASL ORISTANO | ovicaprini |
| 2013 | 044NU25XXX | 18/02/2013 | NU | ASL NUORO | ovicaprini |
| 2013 | 083NU15XXX | 18/02/2013 | NU | ASL NUORO | ovicaprini |
| 2013 | 013SS14XXX | 19/02/2013 | SS | ASL 1 SASSARI | ovicaprini |
| 2013 | 032CA10XXX | 19/02/2013 | CA | ASL 8 CAGLIARI | ovicaprini |
| 2013 | 070NU07XXX | 19/02/2013 | NU | ASL NUORO | ovicaprini |
| 2013 | 013SS12XXX | 19/02/2013 | SS | ASL 1 SASSARI | ovicaprini |
| 2013 | 033OR05XXX | 19/02/2013 | OR | ASL ORISTANO | ovicaprini |
| 2013 | 049OR13XXX | 19/02/2013 | OR | ASL ORISTANO | ovicaprini |
| 2013 | 032CA04XXX | 19/02/2013 | CA | ASL 8 CAGLIARI | ovicaprini |
| 2013 | 057SS18XXX | 19/02/2013 | SS | ASL 1 SASSARI | ovicaprini |
| 2013 | 014OR01XXX | 19/02/2013 | OR | ASL ORISTANO | ovicaprini |
| 2013 | 032CA09XXX | 19/02/2013 | CA | ASL 8 CAGLIARI | ovicaprini |
| 2013 | 049OR12XXX | 19/02/2013 | OR | ASL ORISTANO | ovicaprini |
| 2013 | 049OR12XXX | 19/02/2013 | OR | ASL ORISTANO | ovicaprini |
| 2013 | 091CA06XXX | 19/02/2013 | CA | ASL 8 CAGLIARI | ovicaprini |
| 2013 | 033OR01XXX | 19/02/2013 | OR | ASL ORISTANO | ovicaprini |
| 2013 | 024OR03XXX | 21/02/2013 | OR | ASL ORISTANO | ovicaprini |
| 2013 | 029CA01XXX | 21/02/2013 | CA | ASL 8 CAGLIARI | ovicaprini |
| 2013 | 048OR00XXX | 21/02/2013 | OR | ASL ORISTANO | ovicaprini |
| 2013 | 065CA02XXX | 21/02/2013 | CA | ASL 8 CAGLIARI | ovicaprini |
| 2013 | 056NU06XXX | 21/02/2013 | NU | ASL NUORO | ovicaprini |
| 2013 | 049OR07XXX | 22/02/2013 | OR | ASL ORISTANO | ovicaprini |
| 2013 | 013SS09XXX | 22/02/2013 | SS | ASL 1 SASSARI | ovicaprini |
| 2013 | 049OR07XXX | 22/02/2013 | OR | ASL ORISTANO | ovicaprini |
| 2013 | 083NU00XXX | 22/02/2013 | NU | ASL NUORO | ovicaprini |
| 2013 | 010NU02XXX | 25/02/2013 | NU | ASL NUORO | ovicaprini |
| 2013 | 049NU01XXX | 25/02/2013 | NU | ASL NUORO | ovicaprini |
| 2013 | 008NU00XXX | 25/02/2013 | NU | ASL NUORO | ovicaprini |
| 2013 | 038SS05XXX | 25/02/2013 | SS | ASL 1 SASSARI | ovicaprini |
| 2013 | 038SS05XXX | 25/02/2013 | SS | ASL 1 SASSARI | ovicaprini |
| 2013 | 038SS02XXX | 25/02/2013 | SS | ASL 1 SASSARI | ovicaprini |
| 2013 | 051OR17XXX | 25/02/2013 | OR | ASL ORISTANO | ovicaprini |
| 2013 | 051OR17XXX | 25/02/2013 | OR | ASL ORISTANO | ovicaprini |
| 2013 | 065CA02XXX | 26/02/2013 | CA | ASL 8 CAGLIARI | ovicaprini |
| 2013 | 021OR01XXX | 26/02/2013 | OR | ASL ORISTANO | ovicaprini |
| 2013 | 049OR07XXX | 26/02/2013 | OR | ASL ORISTANO | ovicaprini |
| 2013 | 030SS04XXX | 27/02/2013 | SS | ASL 1 SASSARI | ovicaprini |
| 2013 | 071SS07XXX | 27/02/2013 | SS | ASL 1 SASSARI | ovicaprini |
| 2013 | 008NU02XXX | 28/02/2013 | NU | ASL NUORO | ovicaprini |
| 2013 | 028NU06XXX | 28/02/2013 | NU | ASL NUORO | ovicaprini |
| 2013 | 083NU03XXX | 28/02/2013 | NU | ASL NUORO | ovicaprini |
| 2013 | 038SS07XXX | 28/02/2013 | SS | ASL 1 SASSARI | ovicaprini |
| 2013 | 079OR02XXX | 28/02/2013 | OR | ASL ORISTANO | ovicaprini |
| 2013 | 038SS08XXX | 28/02/2013 | SS | ASL 1 SASSARI | ovicaprini |
| 2013 | 078OR01XXX | 01/03/2013 | OR | ASL ORISTANO | ovicaprini |
| 2013 | 008NU01XXX | 04/03/2013 | NU | ASL NUORO | ovicaprini |
| 2013 | 091CA07XXX | 04/03/2013 | CA | ASL 8 CAGLIARI | ovicaprini |
| 2013 | 064SS70XXX | 04/03/2013 | SS | ASL 1 SASSARI | ovicaprini |
| 2013 | 025OR18XXX | 05/03/2013 | OR | ASL ORISTANO | ovicaprini |
| 2013 | 011SS06XXX | 05/03/2013 | SS | ASL 1 SASSARI | ovicaprini |
| 2013 | 049OR26XXX | 05/03/2013 | OR | ASL ORISTANO | ovicaprini |
| 2013 | 065CA02XXX | 05/03/2013 | CA | ASL 8 CAGLIARI | ovicaprini |
| 2013 | 049OR11XXX | 05/03/2013 | OR | ASL ORISTANO | ovicaprini |
| 2013 | 025OR18XXX | 05/03/2013 | OR | ASL ORISTANO | ovicaprini |
| 2013 | 055CA03XXX | 05/03/2013 | CA | ASL 8 CAGLIARI | ovicaprini |
| 2013 | 044NU04XXX | 05/03/2013 | NU | ASL NUORO | ovicaprini |
| 2013 | 027SS04XXX | 05/03/2013 | SS | ASL 1 SASSARI | ovicaprini |
| 2013 | 077NU04XXX | 07/03/2013 | NU | ASL NUORO | ovicaprini |
| 2013 | 049OR08XXX | 07/03/2013 | OR | ASL ORISTANO | ovicaprini |
| 2013 | 049OR13XXX | 07/03/2013 | OR | ASL ORISTANO | ovicaprini |
| 2013 | 012NU12XXX | 07/03/2013 | NU | ASL NUORO | ovicaprini |
| 2013 | 085CA02XXX | 08/03/2013 | CA | ASL 8 CAGLIARI | ovicaprini |
| 2013 | 059SS03XXX | 08/03/2013 | SS | ASL 1 SASSARI | ovicaprini |
| 2013 | 044NU10XXX | 08/03/2013 | NU | ASL NUORO | ovicaprini |
| 2013 | 012NU12XXX | 08/03/2013 | NU | ASL NUORO | ovicaprini |
| 2013 | 059SS08XXX | 08/03/2013 | SS | ASL 1 SASSARI | ovicaprini |
| 2013 | 057SS05XXX | 08/03/2013 | SS | ASL 1 SASSARI | ovicaprini |
| 2013 | 044NU10XXX | 08/03/2013 | NU | ASL NUORO | ovicaprini |
| 2013 | 045OR06XXX | 11/03/2013 | OR | ASL ORISTANO | ovicaprini |
| 2013 | 063OR04XXX | 11/03/2013 | OR | ASL ORISTANO | ovicaprini |
| 2013 | 057SS05XXX | 11/03/2013 | SS | ASL 1 SASSARI | ovicaprini |
| 2013 | 030SS01XXX | 11/03/2013 | SS | ASL 1 SASSARI | ovicaprini |
| 2013 | 026SS05XXX | 11/03/2013 | SS | ASL 1 SASSARI | ovicaprini |
| 2013 | 083NU00XXX | 11/03/2013 | NU | ASL NUORO | ovicaprini |
| 2013 | 083NU15XXX | 11/03/2013 | NU | ASL NUORO | ovicaprini |
| 2013 | 065CA02XXX | 12/03/2013 | CA | ASL 8 CAGLIARI | ovicaprini |
| 2013 | 056NU03XXX | 12/03/2013 | NU | ASL NUORO | ovicaprini |
| 2013 | 026SS03XXX | 12/03/2013 | SS | ASL 1 SASSARI | ovicaprini |
| 2013 | 064SS72XXX | 12/03/2013 | SS | ASL 1 SASSARI | ovicaprini |
| 2013 | 064SS24XXX | 12/03/2013 | SS | ASL 1 SASSARI | ovicaprini |
| 2013 | 064SS09XXX | 12/03/2013 | SS | ASL 1 SASSARI | ovicaprini |
| 2013 | 064SS01XXX | 12/03/2013 | SS | ASL 1 SASSARI | ovicaprini |
| 2013 | 064SS90XXX | 12/03/2013 | SS | ASL 1 SASSARI | ovicaprini |
| 2013 | 064SS31XXX | 12/03/2013 | SS | ASL 1 SASSARI | ovicaprini |
| 2013 | 064SS64XXX | 14/03/2013 | SS | ASL 1 SASSARI | ovicaprini |
| 2013 | 064SS88XXX | 14/03/2013 | SS | ASL 1 SASSARI | ovicaprini |
| 2013 | 021OR12XXX | 14/03/2013 | OR | ASL ORISTANO | ovicaprini |
| 2013 | 008NU01XXX | 18/03/2013 | NU | ASL NUORO | ovicaprini |
| 2013 | 013SS05XXX | 18/03/2013 | SS | ASL 1 SASSARI | ovicaprini |
| 2013 | 050SS04XXX | 18/03/2013 | SS | ASL 1 SASSARI | ovicaprini |
| 2013 | 052OR05XXX | 18/03/2013 | OR | ASL ORISTANO | ovicaprini |
| 2013 | 030SS07XXX | 18/03/2013 | SS | ASL 1 SASSARI | ovicaprini |
| 2013 | 013SS01XXX | 18/03/2013 | SS | ASL 1 SASSARI | ovicaprini |
| 2013 | 030SS04XXX | 18/03/2013 | SS | ASL 1 SASSARI | ovicaprini |
| 2013 | 063OR05XXX | 18/03/2013 | OR | ASL ORISTANO | ovicaprini |
| 2013 | 008NU01XXX | 18/03/2013 | NU | ASL NUORO | ovicaprini |
| 2013 | 065CA02XXX | 19/03/2013 | CA | ASL 8 CAGLIARI | ovicaprini |
| 2013 | 065CA02XXX | 19/03/2013 | CA | ASL 8 CAGLIARI | ovicaprini |
| 2013 | 013SS14XXX | 22/07/2013 | SS | ASL 1 SASSARI | ovicaprini |
| 2013 | 066SS06XXX | 22/07/2013 | SS | ASL 1 SASSARI | ovicaprini |
| 2013 | 016SS01XXX | 22/07/2013 | SS | ASL 1 SASSARI | ovicaprini |
| 2013 | 064SSc8XXX | 23/07/2013 | SS | ASL 1 SASSARI | ovicaprini |
| 2013 | 064SS61XXX | 23/07/2013 | SS | ASL 1 SASSARI | ovicaprini |
| 2013 | 064SS65XXX | 25/07/2013 | SS | ASL 1 SASSARI | ovicaprini |
| 2013 | 013SS01XXX | 25/07/2013 | SS | ASL 1 SASSARI | ovicaprini |
| 2013 | 016SS01XXX | 25/07/2013 | SS | ASL 1 SASSARI | ovicaprini |
| 2013 | 077NU10XXX | 29/07/2013 | NU | ASL NUORO | ovicaprini |
| 2013 | 029SS05XXX | 29/07/2013 | SS | ASL 1 SASSARI | ovicaprini |
| 2013 | 083NU02XXX | 30/07/2013 | NU | ASL NUORO | ovicaprini |
| 2013 | 064SS56XXX | 30/07/2013 | SS | ASL 1 SASSARI | ovicaprini |
| 2013 | 009NU04XXX | 02/09/2013 | NU | ASL NUORO | ovicaprini |
| 2013 | 009NU06XXX | 02/09/2013 | NU | ASL NUORO | ovicaprini |
| 2013 | 068NU00XXX | 02/09/2013 | NU | ASL NUORO | ovicaprini |
| 2013 | 009NU00XXX | 16/09/2013 | NU | ASL NUORO | ovicaprini |
| 2013 | 009NU05XXX | 07/10/2013 | NU | ASL NUORO | ovicaprini |
| 2013 | 009NU05XXX | 10/10/2013 | NU | ASL NUORO | ovicaprini |
| 2013 | 013SS02XXX | 04/10/2013 | SS | ASL 1 SASSARI | ovicaprini |
| 2013 | 012NU14XXX | 07/10/2013 | NU | ASL NUORO | ovicaprini |
| 2013 | 012NU14XXX | 08/10/2013 | NU | ASL NUORO | ovicaprini |
| 2013 | 012NU14XXX | 10/10/2013 | NU | ASL NUORO | ovicaprini |
| 2013 | 009NU05XXX | 14/10/2013 | NU | ASL NUORO | ovicaprini |
| 2013 | 012NU14XXX | 14/10/2013 | NU | ASL NUORO | ovicaprini |
| 2013 | 012NU14XXX | 11/10/2013 | NU | ASL NUORO | ovicaprini |
| 2013 | 012NU14XXX | 18/10/2013 | NU | ASL NUORO | ovicaprini |
| 2013 | 012NU14XXX | 21/10/2013 | NU | ASL NUORO | ovicaprini |
| 2013 | 012NU14XXX | 22/10/2013 | NU | ASL NUORO | ovicaprini |
| 2013 | 012NU14XXX | 24/10/2013 | NU | ASL NUORO | ovicaprini |
| 2013 | 012NU14XXX | 28/10/2013 | NU | ASL NUORO | ovicaprini |
| 2014 | IT070ORXXX | 02/01/2014 | OR | ASL ORISTANO | ovicaprini |
| 2014 | IT032CAXXX | 07/01/2014 | CA | ASL 8 CAGLIARI | ovicaprini |
| 2014 | IT010CAXXX | 14/01/2014 | CA | ASL 8 CAGLIARI | ovicaprini |
| 2014 | IT072CAXXX | 20/01/2014 | CA | ASL 8 CAGLIARI | ovicaprini |
| 2014 | IT057CAXXX | 20/01/2014 | CA | ASL 8 CAGLIARI | ovicaprini |
| 2014 | IT086CAXXX | 20/01/2014 | CA | ASL 8 CAGLIARI | ovicaprini |
| 2014 | IT029CAXXX | 20/01/2014 | CA | ASL 8 CAGLIARI | ovicaprini |
| 2014 | IT032CAXXX | 20/01/2014 | CA | ASL 8 CAGLIARI | ovicaprini |
| 2014 | IT069CAXXX | 20/01/2014 | CA | ASL 8 CAGLIARI | ovicaprini |
| 2014 | IT057ORXXX | 21/01/2014 | OR | ASL ORISTANO | ovicaprini |
| 2014 | IT057CAXXX | 23/01/2014 | CA | ASL 8 CAGLIARI | ovicaprini |
| 2014 | IT096CAXXX | 23/01/2014 | CA | ASL 8 CAGLIARI | ovicaprini |
| 2014 | IT101CAXXX | 27/01/2014 | CA | ASL 8 CAGLIARI | ovicaprini |
| 2014 | IT096CAXXX | 27/01/2014 | CA | ASL 8 CAGLIARI | ovicaprini |
| 2014 | IT070CAXXX | 27/01/2014 | CA | ASL 8 CAGLIARI | ovicaprini |
| 2014 | IT096CAXXX | 27/01/2014 | CA | ASL 8 CAGLIARI | ovicaprini |
| 2014 | IT092CAXXX | 03/02/2014 | CA | ASL 8 CAGLIARI | ovicaprini |
| 2014 | IT032CAXXX | 03/02/2014 | CA | ASL 8 CAGLIARI | ovicaprini |
| 2014 | IT029ORXXX | 03/02/2014 | OR | ASL ORISTANO | ovicaprini |
| 2014 | IT045CAXXX | 03/02/2014 | CA | ASL 8 CAGLIARI | ovicaprini |
| 2014 | IT045CAXXX | 03/02/2014 | CA | ASL 8 CAGLIARI | ovicaprini |
| 2014 | IT031CAXXX | 03/02/2014 | CA | ASL 8 CAGLIARI | ovicaprini |
| 2014 | IT069CAXXX | 03/02/2014 | CA | ASL 8 CAGLIARI | ovicaprini |
| 2014 | IT057CAXXX | 05/02/2014 | CA | ASL 8 CAGLIARI | ovicaprini |
| 2014 | IT094CAXXX | 06/02/2014 | CA | ASL 8 CAGLIARI | ovicaprini |
| 2014 | IT012ORXXX | 10/02/2014 | OR | ASL ORISTANO | ovicaprini |
| 2014 | IT012ORXXX | 10/02/2014 | OR | ASL ORISTANO | ovicaprini |
| 2014 | IT029CAXXX | 10/02/2014 | CA | ASL 8 CAGLIARI | ovicaprini |
| 2014 | IT031CAXXX | 10/02/2014 | CA | ASL 8 CAGLIARI | ovicaprini |
| 2014 | IT028ORXXX | 11/02/2014 | OR | ASL ORISTANO | ovicaprini |
| 2014 | IT029ORXXX | 11/02/2014 | OR | ASL ORISTANO | ovicaprini |
| 2014 | IT080NUXXX | 12/02/2014 | NU | ASL NUORO | ovicaprini |
| 2014 | IT034NUXXX | 12/02/2014 | NU | ASL NUORO | ovicaprini |
| 2014 | IT066ORXXX | 12/02/2014 | OR | ASL ORISTANO | ovicaprini |
| 2014 | IT091CAXXX | 12/02/2014 | CA | ASL 8 CAGLIARI | ovicaprini |
| 2014 | IT010CAXXX | 13/02/2014 | CA | ASL 8 CAGLIARI | ovicaprini |
| 2014 | IT091CAXXX | 17/02/2014 | CA | ASL 8 CAGLIARI | ovicaprini |
| 2014 | IT091CAXXX | 17/02/2014 | CA | ASL 8 CAGLIARI | ovicaprini |
| 2014 | IT035CAXXX | 17/02/2014 | CA | ASL 8 CAGLIARI | ovicaprini |
| 2014 | IT029ORXXX | 17/02/2014 | OR | ASL ORISTANO | ovicaprini |
| 2014 | IT092CAXXX | 17/02/2014 | CA | ASL 8 CAGLIARI | ovicaprini |
| 2014 | IT057ORXXX | 17/02/2014 | OR | ASL ORISTANO | ovicaprini |
| 2014 | IT057ORXXX | 17/02/2014 | OR | ASL ORISTANO | ovicaprini |
| 2014 | IT089CAXXX | 17/02/2014 | CA | ASL 8 CAGLIARI | ovicaprini |
| 2014 | IT072CAXXX | 21/02/2014 | CA | ASL 8 CAGLIARI | ovicaprini |
| 2014 | IT056CAXXX | 24/02/2014 | CA | ASL 8 CAGLIARI | ovicaprini |
| 2014 | IT034CAXXX | 24/02/2014 | CA | ASL 8 CAGLIARI | ovicaprini |
| 2014 | IT029ORXXX | 24/02/2014 | OR | ASL ORISTANO | ovicaprini |
| 2014 | IT060CAXXX | 24/02/2014 | CA | ASL 8 CAGLIARI | ovicaprini |
| 2014 | IT096CAXXX | 24/02/2014 | CA | ASL 8 CAGLIARI | ovicaprini |
| 2014 | IT096CAXXX | 24/02/2014 | CA | ASL 8 CAGLIARI | ovicaprini |
| 2014 | IT096CAXXX | 24/02/2014 | CA | ASL 8 CAGLIARI | ovicaprini |
| 2014 | IT047ORXXX | 26/02/2014 | OR | ASL ORISTANO | ovicaprini |
| 2014 | IT094CAXXX | 27/02/2014 | CA | ASL 8 CAGLIARI | ovicaprini |
| 2014 | IT072CAXXX | 27/02/2014 | CA | ASL 8 CAGLIARI | ovicaprini |
| 2014 | IT052CAXXX | 27/02/2014 | CA | ASL 8 CAGLIARI | ovicaprini |
| 2014 | IT049SSXXX | 27/02/2014 | SS | ASL 1 SASSARI | ovicaprini |
| 2014 | IT049SSXXX | 27/02/2014 | SS | ASL 1 SASSARI | ovicaprini |
| 2014 | IT024NUXXX | 27/02/2014 | NU | ASL NUORO | ovicaprini |
| 2014 | IT049SSXXX | 27/02/2014 | SS | ASL 1 SASSARI | ovicaprini |
| 2014 | IT024NUXXX | 27/02/2014 | NU | ASL NUORO | ovicaprini |
| 2014 | IT049SSXXX | 27/02/2014 | SS | ASL 1 SASSARI | ovicaprini |
| 2014 | IT034CAXXX | 03/03/2014 | CA | ASL 8 CAGLIARI | ovicaprini |
| 2014 | IT091CAXXX | 03/03/2014 | CA | ASL 8 CAGLIARI | ovicaprini |
| 2014 | IT049SSXXX | 03/03/2014 | SS | ASL 1 SASSARI | ovicaprini |
| 2014 | IT048CAXXX | 03/03/2014 | CA | ASL 8 CAGLIARI | ovicaprini |
| 2014 | IT086CAXXX | 03/03/2014 | CA | ASL 8 CAGLIARI | ovicaprini |
| 2014 | IT046CAXXX | 03/03/2014 | CA | ASL 8 CAGLIARI | ovicaprini |
| 2014 | IT029CAXXX | 03/03/2014 | CA | ASL 8 CAGLIARI | ovicaprini |
| 2014 | IT043CAXXX | 03/03/2014 | CA | ASL 8 CAGLIARI | ovicaprini |
| 2014 | IT032CAXXX | 03/03/2014 | CA | ASL 8 CAGLIARI | ovicaprini |
| 2014 | IT092CAXXX | 03/03/2014 | CA | ASL 8 CAGLIARI | ovicaprini |
| 2014 | IT092CAXXX | 03/03/2014 | CA | ASL 8 CAGLIARI | ovicaprini |
| 2014 | IT069CAXXX | 03/03/2014 | CA | ASL 8 CAGLIARI | ovicaprini |
| 2014 | IT040CAXXX | 04/03/2014 | CA | ASL 8 CAGLIARI | ovicaprini |
| 2014 | IT094CAXXX | 04/03/2014 | CA | ASL 8 CAGLIARI | ovicaprini |
| 2014 | IT094CAXXX | 04/03/2014 | CA | ASL 8 CAGLIARI | ovicaprini |
| 2014 | IT094CAXXX | 04/03/2014 | CA | ASL 8 CAGLIARI | ovicaprini |
| 2014 | IT034CAXXX | 05/03/2014 | CA | ASL 8 CAGLIARI | ovicaprini |
| 2014 | IT101CAXXX | 06/03/2014 | CA | ASL 8 CAGLIARI | ovicaprini |
| 2014 | IT066SSXXX | 06/03/2014 | SS | ASL 1 SASSARI | ovicaprini |
| 2014 | IT052SSXXX | 06/03/2014 | SS | ASL 1 SASSARI | ovicaprini |
| 2014 | IT033CAXXX | 06/03/2014 | CA | ASL 8 CAGLIARI | ovicaprini |
| 2014 | IT040CAXXX | 06/03/2014 | CA | ASL 8 CAGLIARI | ovicaprini |
| 2014 | IT031CAXXX | 07/03/2014 | CA | ASL 8 CAGLIARI | ovicaprini |
| 2014 | IT024CAXXX | 07/03/2014 | CA | ASL 8 CAGLIARI | ovicaprini |
| 2014 | IT078CAXXX | 10/03/2014 | CA | ASL 8 CAGLIARI | ovicaprini |
| 2014 | IT091CAXXX | 10/03/2014 | CA | ASL 8 CAGLIARI | ovicaprini |
| 2014 | IT077ORXXX | 10/03/2014 | OR | ASL ORISTANO | ovicaprini |
| 2014 | IT101CAXXX | 10/03/2014 | CA | ASL 8 CAGLIARI | ovicaprini |
| 2014 | IT028ORXXX | 10/03/2014 | OR | ASL ORISTANO | ovicaprini |
| 2014 | IT101CAXXX | 10/03/2014 | CA | ASL 8 CAGLIARI | ovicaprini |
| 2014 | IT096CAXXX | 10/03/2014 | CA | ASL 8 CAGLIARI | ovicaprini |
| 2014 | IT101CAXXX | 11/03/2014 | CA | ASL 8 CAGLIARI | ovicaprini |
| 2014 | IT101CAXXX | 11/03/2014 | CA | ASL 8 CAGLIARI | ovicaprini |
| 2014 | IT077ORXXX | 11/03/2014 | OR | ASL ORISTANO | ovicaprini |
| 2014 | IT057ORXXX | 11/03/2014 | OR | ASL ORISTANO | ovicaprini |
| 2014 | IT029CAXXX | 11/03/2014 | CA | ASL 8 CAGLIARI | ovicaprini |
| 2014 | IT034NUXXX | 12/03/2014 | NU | ASL NUORO | ovicaprini |
| 2014 | IT012CAXXX | 12/03/2014 | CA | ASL 8 CAGLIARI | ovicaprini |
| 2014 | IT025CAXXX | 13/03/2014 | CA | ASL 8 CAGLIARI | ovicaprini |
| 2014 | IT053SSXXX | 14/03/2014 | SS | ASL 1 SASSARI | ovicaprini |
| 2014 | IT035CAXXX | 17/03/2014 | CA | ASL 8 CAGLIARI | ovicaprini |
| 2014 | IT094CAXXX | 17/03/2014 | CA | ASL 8 CAGLIARI | ovicaprini |
| 2014 | IT045CAXXX | 18/03/2014 | CA | ASL 8 CAGLIARI | ovicaprini |
| 2014 | IT032CAXXX | 18/03/2014 | CA | ASL 8 CAGLIARI | ovicaprini |
| 2014 | IT045CAXXX | 18/03/2014 | CA | ASL 8 CAGLIARI | ovicaprini |
| 2014 | IT101CAXXX | 18/03/2014 | CA | ASL 8 CAGLIARI | ovicaprini |
| 2014 | IT029CAXXX | 18/03/2014 | CA | ASL 8 CAGLIARI | ovicaprini |
| 2014 | IT034NUXXX | 19/03/2014 | NU | ASL NUORO | ovicaprini |
| 2014 | IT035CAXXX | 19/03/2014 | CA | ASL 8 CAGLIARI | ovicaprini |
| 2014 | IT010CAXXX | 19/03/2014 | CA | ASL 8 CAGLIARI | ovicaprini |
| 2014 | IT101CAXXX | 21/03/2014 | CA | ASL 8 CAGLIARI | ovicaprini |
| 2014 | IT010CAXXX | 21/03/2014 | CA | ASL 8 CAGLIARI | ovicaprini |
| 2014 | IT078CAXXX | 21/03/2014 | CA | ASL 8 CAGLIARI | ovicaprini |
| 2014 | IT025CAXXX | 24/03/2014 | CA | ASL 8 CAGLIARI | ovicaprini |
| 2014 | IT035CAXXX | 24/03/2014 | CA | ASL 8 CAGLIARI | ovicaprini |
| 2014 | IT035CAXXX | 24/03/2014 | CA | ASL 8 CAGLIARI | ovicaprini |
| 2014 | IT057CAXXX | 24/03/2014 | CA | ASL 8 CAGLIARI | ovicaprini |
| 2014 | IT016CAXXX | 25/03/2014 | CA | ASL 8 CAGLIARI | ovicaprini |
| 2014 | IT029CAXXX | 25/03/2014 | CA | ASL 8 CAGLIARI | ovicaprini |
| 2014 | IT078CAXXX | 25/03/2014 | CA | ASL 8 CAGLIARI | ovicaprini |
| 2014 | IT019CAXXX | 25/03/2014 | CA | ASL 8 CAGLIARI | ovicaprini |
| 2014 | IT057ORXXX | 25/03/2014 | OR | ASL ORISTANO | ovicaprini |
| 2014 | IT034NUXXX | 26/03/2014 | NU | ASL NUORO | ovicaprini |
| 2014 | IT016CAXXX | 27/03/2014 | CA | ASL 8 CAGLIARI | ovicaprini |
| 2014 | IT096CAXXX | 28/03/2014 | CA | ASL 8 CAGLIARI | ovicaprini |
| 2014 | IT031CAXXX | 28/03/2014 | CA | ASL 8 CAGLIARI | ovicaprini |
| 2014 | IT096CAXXX | 28/03/2014 | CA | ASL 8 CAGLIARI | ovicaprini |
| 2014 | IT054ORXXX | 31/03/2014 | OR | ASL ORISTANO | ovicaprini |
| 2014 | IT032CAXXX | 31/03/2014 | CA | ASL 8 CAGLIARI | ovicaprini |
| 2014 | IT012ORXXX | 31/03/2014 | OR | ASL ORISTANO | ovicaprini |
| 2014 | IT101CAXXX | 01/04/2014 | CA | ASL 8 CAGLIARI | ovicaprini |
| 2014 | IT032CAXXX | 02/04/2014 | CA | ASL 8 CAGLIARI | ovicaprini |
| 2014 | IT033CAXXX | 03/04/2014 | CA | ASL 8 CAGLIARI | ovicaprini |
| 2014 | IT060CAXXX | 04/04/2014 | CA | ASL 8 CAGLIARI | ovicaprini |
| 2014 | IT048ORXXX | 07/04/2014 | OR | ASL ORISTANO | ovicaprini |
| 2014 | IT092CAXXX | 07/04/2014 | CA | ASL 8 CAGLIARI | ovicaprini |
| 2014 | IT101CAXXX | 07/04/2014 | CA | ASL 8 CAGLIARI | ovicaprini |
| 2014 | IT101CAXXX | 07/04/2014 | CA | ASL 8 CAGLIARI | ovicaprini |
| 2014 | IT084CAXXX | 07/04/2014 | CA | ASL 8 CAGLIARI | ovicaprini |
| 2014 | IT026ORXXX | 07/04/2014 | OR | ASL ORISTANO | ovicaprini |
| 2014 | IT092CAXXX | 07/04/2014 | CA | ASL 8 CAGLIARI | ovicaprini |
| 2014 | IT070CAXXX | 07/04/2014 | CA | ASL 8 CAGLIARI | ovicaprini |
| 2014 | IT101CAXXX | 08/04/2014 | CA | ASL 8 CAGLIARI | ovicaprini |
| 2014 | IT070ORXXX | 10/04/2014 | OR | ASL ORISTANO | ovicaprini |
| 2014 | IT096CAXXX | 10/04/2014 | CA | ASL 8 CAGLIARI | ovicaprini |
| 2014 | IT012ORXXX | 11/04/2014 | OR | ASL ORISTANO | ovicaprini |
| 2014 | IT065CAXXX | 11/04/2014 | CA | ASL 8 CAGLIARI | ovicaprini |
| 2014 | IT012ORXXX | 11/04/2014 | OR | ASL ORISTANO | ovicaprini |
| 2014 | IT042ORXXX | 11/04/2014 | OR | ASL ORISTANO | ovicaprini |
| 2014 | IT078CAXXX | 11/04/2014 | CA | ASL 8 CAGLIARI | ovicaprini |
| 2014 | IT070ORXXX | 14/04/2014 | OR | ASL ORISTANO | ovicaprini |
| 2014 | IT084CAXXX | 14/04/2014 | CA | ASL 8 CAGLIARI | ovicaprini |
| 2014 | IT029CAXXX | 14/04/2014 | CA | ASL 8 CAGLIARI | ovicaprini |
| 2014 | IT092CAXXX | 14/04/2014 | CA | ASL 8 CAGLIARI | ovicaprini |
| 2014 | IT034NUXXX | 16/04/2014 | NU | ASL NUORO | ovicaprini |
| 2014 | IT012ORXXX | 18/04/2014 | OR | ASL ORISTANO | ovicaprini |
| 2014 | IT070ORXXX | 18/04/2014 | OR | ASL ORISTANO | ovicaprini |
| 2014 | IT012ORXXX | 18/04/2014 | OR | ASL ORISTANO | ovicaprini |
| 2014 | IT029CAXXX | 22/04/2014 | CA | ASL 8 CAGLIARI | ovicaprini |
| 2014 | IT012ORXXX | 24/04/2014 | OR | ASL ORISTANO | ovicaprini |
| 2014 | IT029ORXXX | 28/04/2014 | OR | ASL ORISTANO | ovicaprini |
| 2014 | IT077CAXXX | 28/04/2014 | CA | ASL 8 CAGLIARI | ovicaprini |
| 2014 | IT057ORXXX | 28/04/2014 | OR | ASL ORISTANO | ovicaprini |
| 2014 | IT055CAXXX | 30/04/2014 | CA | ASL 8 CAGLIARI | ovicaprini |
| 2014 | IT055CAXXX | 30/04/2014 | CA | ASL 8 CAGLIARI | ovicaprini |
| 2014 | IT096CAXXX | 02/05/2014 | CA | ASL 8 CAGLIARI | ovicaprini |
| 2014 | IT031CAXXX | 02/05/2014 | CA | ASL 8 CAGLIARI | ovicaprini |
| 2014 | IT053CAXXX | 03/05/2014 | CA | ASL 8 CAGLIARI | ovicaprini |
| 2014 | IT031CAXXX | 05/05/2014 | CA | ASL 8 CAGLIARI | ovicaprini |
| 2014 | IT092CAXXX | 05/05/2014 | CA | ASL 8 CAGLIARI | ovicaprini |
| 2014 | IT036CAXXX | 05/05/2014 | CA | ASL 8 CAGLIARI | ovicaprini |
| 2014 | IT078CAXXX | 05/05/2014 | CA | ASL 8 CAGLIARI | ovicaprini |
| 2014 | IT029ORXXX | 05/05/2014 | OR | ASL ORISTANO | ovicaprini |
| 2014 | IT012ORXXX | 05/05/2014 | OR | ASL ORISTANO | ovicaprini |
| 2014 | IT029CAXXX | 05/05/2014 | CA | ASL 8 CAGLIARI | ovicaprini |
| 2014 | IT045CAXXX | 05/05/2014 | CA | ASL 8 CAGLIARI | ovicaprini |
| 2014 | IT084CAXXX | 05/05/2014 | CA | ASL 8 CAGLIARI | ovicaprini |
| 2014 | IT057CAXXX | 05/05/2014 | CA | ASL 8 CAGLIARI | ovicaprini |
| 2014 | IT092CAXXX | 05/05/2014 | CA | ASL 8 CAGLIARI | ovicaprini |
| 2014 | IT042ORXXX | 05/05/2014 | OR | ASL ORISTANO | ovicaprini |
| 2014 | IT012CAXXX | 06/05/2014 | CA | ASL 8 CAGLIARI | ovicaprini |
| 2014 | IT057CAXXX | 06/05/2014 | CA | ASL 8 CAGLIARI | ovicaprini |
| 2014 | IT057CAXXX | 07/05/2014 | CA | ASL 8 CAGLIARI | ovicaprini |
| 2014 | IT057CAXXX | 07/05/2014 | CA | ASL 8 CAGLIARI | ovicaprini |
| 2014 | IT006CAXXX | 09/05/2014 | CA | ASL 8 CAGLIARI | ovicaprini |
| 2014 | IT006CAXXX | 09/05/2014 | CA | ASL 8 CAGLIARI | ovicaprini |
| 2014 | IT078CAXXX | 09/05/2014 | CA | ASL 8 CAGLIARI | ovicaprini |
| 2014 | IT078CAXXX | 09/05/2014 | CA | ASL 8 CAGLIARI | ovicaprini |
| 2014 | IT055CAXXX | 09/05/2014 | CA | ASL 8 CAGLIARI | ovicaprini |
| 2014 | IT006CAXXX | 09/05/2014 | CA | ASL 8 CAGLIARI | ovicaprini |
| 2014 | IT069CAXXX | 09/05/2014 | CA | ASL 8 CAGLIARI | ovicaprini |
| 2014 | IT031CAXXX | 09/05/2014 | CA | ASL 8 CAGLIARI | ovicaprini |
| 2014 | IT057CAXXX | 12/05/2014 | CA | ASL 8 CAGLIARI | ovicaprini |
| 2014 | IT055CAXXX | 12/05/2014 | CA | ASL 8 CAGLIARI | ovicaprini |
| 2014 | IT045CAXXX | 12/05/2014 | CA | ASL 8 CAGLIARI | ovicaprini |
| 2014 | IT023CAXXX | 12/05/2014 | CA | ASL 8 CAGLIARI | ovicaprini |
| 2014 | IT023CAXXX | 12/05/2014 | CA | ASL 8 CAGLIARI | ovicaprini |
| 2014 | IT084CAXXX | 12/05/2014 | CA | ASL 8 CAGLIARI | ovicaprini |
| 2014 | IT029CAXXX | 12/05/2014 | CA | ASL 8 CAGLIARI | ovicaprini |
| 2014 | IT096CAXXX | 12/05/2014 | CA | ASL 8 CAGLIARI | ovicaprini |
| 2014 | IT012ORXXX | 13/05/2014 | OR | ASL ORISTANO | ovicaprini |
| 2014 | IT057CAXXX | 14/05/2014 | CA | ASL 8 CAGLIARI | ovicaprini |
| 2014 | IT029CAXXX | 14/05/2014 | CA | ASL 8 CAGLIARI | ovicaprini |
| 2014 | IT034NUXXX | 14/05/2014 | NU | ASL NUORO | ovicaprini |
| 2014 | IT032CAXXX | 14/05/2014 | CA | ASL 8 CAGLIARI | ovicaprini |
| 2014 | IT29CA0XXX | 15/05/2014 | CA | ASL 8 CAGLIARI | ovicaprini |
| 2014 | IT078CAXXX | 16/05/2014 | CA | ASL 8 CAGLIARI | ovicaprini |
| 2014 | IT032CAXXX | 16/05/2014 | CA | ASL 8 CAGLIARI | ovicaprini |
| 2014 | IT031CAXXX | 19/05/2014 | CA | ASL 8 CAGLIARI | ovicaprini |
| 2014 | IT072CAXXX | 19/05/2014 | CA | ASL 8 CAGLIARI | ovicaprini |
| 2014 | IT046CAXXX | 19/05/2014 | CA | ASL 8 CAGLIARI | ovicaprini |
| 2014 | IT046CAXXX | 19/05/2014 | CA | ASL 8 CAGLIARI | ovicaprini |
| 2014 | IT104CAXXX | 19/05/2014 | CA | ASL 8 CAGLIARI | ovicaprini |
| 2014 | IT045CAXXX | 19/05/2014 | CA | ASL 8 CAGLIARI | ovicaprini |
| 2014 | IT039ORXXX | 19/05/2014 | OR | ASL ORISTANO | ovicaprini |
| 2014 | IT029CAXXX | 19/05/2014 | CA | ASL 8 CAGLIARI | ovicaprini |
| 2014 | IT092CAXXX | 19/05/2014 | CA | ASL 8 CAGLIARI | ovicaprini |
| 2014 | IT096CAXXX | 19/05/2014 | CA | ASL 8 CAGLIARI | ovicaprini |
| 2014 | IT096CAXXX | 19/05/2014 | CA | ASL 8 CAGLIARI | ovicaprini |
| 2014 | IT034NUXXX | 21/05/2014 | NU | ASL NUORO | ovicaprini |
| 2014 | IT057CAXXX | 21/05/2014 | CA | ASL 8 CAGLIARI | ovicaprini |
| 2014 | IT029CAXXX | 22/05/2014 | CA | ASL 8 CAGLIARI | ovicaprini |
| 2014 | IT025CAXXX | 23/05/2014 | CA | ASL 8 CAGLIARI | ovicaprini |
| 2014 | IT025CAXXX | 23/05/2014 | CA | ASL 8 CAGLIARI | ovicaprini |
| 2014 | IT057CAXXX | 23/05/2014 | CA | ASL 8 CAGLIARI | ovicaprini |
| 2014 | IT031CAXXX | 26/05/2014 | CA | ASL 8 CAGLIARI | ovicaprini |
| 2014 | IT025CAXXX | 26/05/2014 | CA | ASL 8 CAGLIARI | ovicaprini |
| 2014 | IT016CAXXX | 26/05/2014 | CA | ASL 8 CAGLIARI | ovicaprini |
| 2014 | IT033CAXXX | 26/05/2014 | CA | ASL 8 CAGLIARI | ovicaprini |
| 2014 | IT086CAXXX | 26/05/2014 | CA | ASL 8 CAGLIARI | ovicaprini |
| 2014 | IT091CAXXX | 26/05/2014 | CA | ASL 8 CAGLIARI | ovicaprini |
| 2014 | IT091CAXXX | 26/05/2014 | CA | ASL 8 CAGLIARI | ovicaprini |
| 2014 | IT036NUXXX | 26/05/2014 | NU | ASL NUORO | ovicaprini |
| 2014 | IT084CAXXX | 26/05/2014 | CA | ASL 8 CAGLIARI | ovicaprini |
| 2014 | IT029ORXXX | 26/05/2014 | OR | ASL ORISTANO | ovicaprini |
| 2014 | IT045CAXXX | 26/05/2014 | CA | ASL 8 CAGLIARI | ovicaprini |
| 2014 | IT046ORXXX | 26/05/2014 | OR | ASL ORISTANO | ovicaprini |
| 2014 | IT053CAXXX | 26/05/2014 | CA | ASL 8 CAGLIARI | ovicaprini |
| 2014 | IT034NUXXX | 28/05/2014 | NU | ASL NUORO | ovicaprini |
| 2014 | IT057CAXXX | 28/05/2014 | CA | ASL 8 CAGLIARI | ovicaprini |
| 2014 | IT070ORXXX | 29/05/2014 | OR | ASL ORISTANO | ovicaprini |
| 2014 | IT032CAXXX | 29/05/2014 | CA | ASL 8 CAGLIARI | ovicaprini |
| 2014 | IT012CAXXX | 30/05/2014 | CA | ASL 8 CAGLIARI | ovicaprini |
| 2014 | IT012CAXXX | 30/05/2014 | CA | ASL 8 CAGLIARI | ovicaprini |
| 2014 | IT036ORXXX | 30/05/2014 | OR | ASL ORISTANO | ovicaprini |
| 2014 | IT086CAXXX | 30/05/2014 | CA | ASL 8 CAGLIARI | ovicaprini |
| 2014 | IT086CAXXX | 30/05/2014 | CA | ASL 8 CAGLIARI | ovicaprini |
| 2014 | IT046CAXXX | 30/05/2014 | CA | ASL 8 CAGLIARI | ovicaprini |
| 2014 | IT070ORXXX | 30/05/2014 | OR | ASL ORISTANO | ovicaprini |
| 2014 | IT078CAXXX | 30/05/2014 | CA | ASL 8 CAGLIARI | ovicaprini |
| 2014 | IT032CAXXX | 30/05/2014 | CA | ASL 8 CAGLIARI | ovicaprini |
| 2014 | IT065CAXXX | 03/06/2014 | CA | ASL 8 CAGLIARI | ovicaprini |
| 2014 | IT091CAXXX | 03/06/2014 | CA | ASL 8 CAGLIARI | ovicaprini |
| 2014 | IT091CAXXX | 03/06/2014 | CA | ASL 8 CAGLIARI | ovicaprini |
| 2014 | IT039ORXXX | 03/06/2014 | OR | ASL ORISTANO | ovicaprini |
| 2014 | IT086CAXXX | 03/06/2014 | CA | ASL 8 CAGLIARI | ovicaprini |
| 2014 | IT087CAXXX | 03/06/2014 | CA | ASL 8 CAGLIARI | ovicaprini |
| 2014 | IT029CAXXX | 03/06/2014 | CA | ASL 8 CAGLIARI | ovicaprini |
| 2014 | IT045CAXXX | 03/06/2014 | CA | ASL 8 CAGLIARI | ovicaprini |
| 2014 | IT031CAXXX | 03/06/2014 | CA | ASL 8 CAGLIARI | ovicaprini |
| 2014 | IT070CAXXX | 03/06/2014 | CA | ASL 8 CAGLIARI | ovicaprini |
| 2014 | IT034NUXXX | 04/06/2014 | NU | ASL NUORO | ovicaprini |
| 2014 | IT057CAXXX | 05/06/2014 | CA | ASL 8 CAGLIARI | ovicaprini |
| 2014 | IT092CAXXX | 06/06/2014 | CA | ASL 8 CAGLIARI | ovicaprini |
| 2014 | IT047CAXXX | 06/06/2014 | CA | ASL 8 CAGLIARI | ovicaprini |
| 2014 | IT033CAXXX | 06/06/2014 | CA | ASL 8 CAGLIARI | ovicaprini |
| 2014 | IT012CAXXX | 06/06/2014 | CA | ASL 8 CAGLIARI | ovicaprini |
| 2014 | IT047CAXXX | 06/06/2014 | CA | ASL 8 CAGLIARI | ovicaprini |
| 2014 | IT036NUXXX | 06/06/2014 | NU | ASL NUORO | ovicaprini |
| 2014 | IT033CAXXX | 06/06/2014 | CA | ASL 8 CAGLIARI | ovicaprini |
| 2014 | IT036NUXXX | 06/06/2014 | NU | ASL NUORO | ovicaprini |
| 2014 | IT047CAXXX | 06/06/2014 | CA | ASL 8 CAGLIARI | ovicaprini |
| 2014 | IT032CAXXX | 06/06/2014 | CA | ASL 8 CAGLIARI | ovicaprini |
| 2014 | IT057CAXXX | 09/06/2014 | CA | ASL 8 CAGLIARI | ovicaprini |
| 2014 | IT092CAXXX | 09/06/2014 | CA | ASL 8 CAGLIARI | ovicaprini |
| 2014 | IT095CAXXX | 09/06/2014 | CA | ASL 8 CAGLIARI | ovicaprini |
| 2014 | IT045CAXXX | 09/06/2014 | CA | ASL 8 CAGLIARI | ovicaprini |
| 2014 | IT101CAXXX | 09/06/2014 | CA | ASL 8 CAGLIARI | ovicaprini |
| 2014 | IT047CAXXX | 09/06/2014 | CA | ASL 8 CAGLIARI | ovicaprini |
| 2014 | IT091CAXXX | 09/06/2014 | CA | ASL 8 CAGLIARI | ovicaprini |
| 2014 | IT091CAXXX | 09/06/2014 | CA | ASL 8 CAGLIARI | ovicaprini |
| 2014 | IT048ORXXX | 09/06/2014 | OR | ASL ORISTANO | ovicaprini |
| 2014 | IT012ORXXX | 09/06/2014 | OR | ASL ORISTANO | ovicaprini |
| 2014 | IT070CAXXX | 09/06/2014 | CA | ASL 8 CAGLIARI | ovicaprini |
| 2014 | IT031CAXXX | 09/06/2014 | CA | ASL 8 CAGLIARI | ovicaprini |
| 2014 | IT096CAXXX | 09/06/2014 | CA | ASL 8 CAGLIARI | ovicaprini |
| 2014 | IT001CAXXX | 11/06/2014 | CA | ASL 8 CAGLIARI | ovicaprini |
| 2014 | IT028ORXXX | 11/06/2014 | OR | ASL ORISTANO | ovicaprini |
| 2014 | IT032CAXXX | 13/06/2014 | CA | ASL 8 CAGLIARI | ovicaprini |
| 2014 | IT065CAXXX | 13/06/2014 | CA | ASL 8 CAGLIARI | ovicaprini |
| 2014 | IT094CAXXX | 13/06/2014 | CA | ASL 8 CAGLIARI | ovicaprini |
| 2014 | IT094CAXXX | 13/06/2014 | CA | ASL 8 CAGLIARI | ovicaprini |
| 2014 | IT001CAXXX | 13/06/2014 | CA | ASL 8 CAGLIARI | ovicaprini |
| 2014 | IT001CAXXX | 13/06/2014 | CA | ASL 8 CAGLIARI | ovicaprini |
| 2014 | IT065CAXXX | 16/06/2014 | CA | ASL 8 CAGLIARI | ovicaprini |
| 2014 | IT092CAXXX | 16/06/2014 | CA | ASL 8 CAGLIARI | ovicaprini |
| 2014 | IT057CAXXX | 16/06/2014 | CA | ASL 8 CAGLIARI | ovicaprini |
| 2014 | IT047CAXXX | 16/06/2014 | CA | ASL 8 CAGLIARI | ovicaprini |
| 2014 | IT004ORXXX | 16/06/2014 | OR | ASL ORISTANO | ovicaprini |
| 2014 | IT086CAXXX | 16/06/2014 | CA | ASL 8 CAGLIARI | ovicaprini |
| 2014 | IT086CAXXX | 16/06/2014 | CA | ASL 8 CAGLIARI | ovicaprini |
| 2014 | IT045CAXXX | 16/06/2014 | CA | ASL 8 CAGLIARI | ovicaprini |
| 2014 | IT029CAXXX | 16/06/2014 | CA | ASL 8 CAGLIARI | ovicaprini |
| 2014 | IT032CAXXX | 16/06/2014 | CA | ASL 8 CAGLIARI | ovicaprini |
| 2014 | IT096CAXXX | 16/06/2014 | CA | ASL 8 CAGLIARI | ovicaprini |
| 2014 | IT096CAXXX | 16/06/2014 | CA | ASL 8 CAGLIARI | ovicaprini |
| 2014 | IT001CAXXX | 18/06/2014 | CA | ASL 8 CAGLIARI | ovicaprini |
| 2014 | IT029CAXXX | 20/06/2014 | CA | ASL 8 CAGLIARI | ovicaprini |
| 2014 | IT029CAXXX | 20/06/2014 | CA | ASL 8 CAGLIARI | ovicaprini |
| 2014 | IT073CAXXX | 23/06/2014 | CA | ASL 8 CAGLIARI | ovicaprini |
| 2014 | IT073CAXXX | 23/06/2014 | CA | ASL 8 CAGLIARI | ovicaprini |
| 2014 | IT073CAXXX | 23/06/2014 | CA | ASL 8 CAGLIARI | ovicaprini |
| 2014 | IT066CAXXX | 23/06/2014 | CA | ASL 8 CAGLIARI | ovicaprini |
| 2014 | IT046CAXXX | 23/06/2014 | CA | ASL 8 CAGLIARI | ovicaprini |
| 2014 | IT045CAXXX | 23/06/2014 | CA | ASL 8 CAGLIARI | ovicaprini |
| 2014 | IT091CAXXX | 23/06/2014 | CA | ASL 8 CAGLIARI | ovicaprini |
| 2014 | IT011NUXXX | 23/06/2014 | NU | ASL NUORO | ovicaprini |
| 2014 | IT069ORXXX | 23/06/2014 | OR | ASL ORISTANO | ovicaprini |
| 2014 | IT032CAXXX | 23/06/2014 | CA | ASL 8 CAGLIARI | ovicaprini |
| 2014 | IT092CAXXX | 27/06/2014 | CA | ASL 8 CAGLIARI | ovicaprini |
| 2014 | IT086CAXXX | 30/06/2014 | CA | ASL 8 CAGLIARI | ovicaprini |
| 2014 | IT029ORXXX | 30/06/2014 | OR | ASL ORISTANO | ovicaprini |
| 2014 | IT052NUXXX | 30/06/2014 | NU | ASL NUORO | ovicaprini |
| 2014 | IT091CAXXX | 30/06/2014 | CA | ASL 8 CAGLIARI | ovicaprini |
| 2014 | IT092CAXXX | 30/06/2014 | CA | ASL 8 CAGLIARI | ovicaprini |
| 2014 | IT101CAXXX | 30/06/2014 | CA | ASL 8 CAGLIARI | ovicaprini |
| 2014 | IT069ORXXX | 30/06/2014 | OR | ASL ORISTANO | ovicaprini |
| 2014 | IT045CAXXX | 30/06/2014 | CA | ASL 8 CAGLIARI | ovicaprini |
| 2014 | IT032CAXXX | 30/06/2014 | CA | ASL 8 CAGLIARI | ovicaprini |
| 2014 | IT066CAXXX | 02/07/2014 | CA | ASL 8 CAGLIARI | ovicaprini |
| 2014 | IT024CAXXX | 07/07/2014 | CA | ASL 8 CAGLIARI | ovicaprini |
| 2014 | IT073CAXXX | 07/07/2014 | CA | ASL 8 CAGLIARI | ovicaprini |
| 2014 | IT073CAXXX | 07/07/2014 | CA | ASL 8 CAGLIARI | ovicaprini |
| 2014 | IT044ORXXX | 07/07/2014 | OR | ASL ORISTANO | ovicaprini |
| 2014 | IT077CAXXX | 07/07/2014 | CA | ASL 8 CAGLIARI | ovicaprini |
| 2014 | IT091CAXXX | 07/07/2014 | CA | ASL 8 CAGLIARI | ovicaprini |
| 2014 | IT091CAXXX | 07/07/2014 | CA | ASL 8 CAGLIARI | ovicaprini |
| 2014 | IT077CAXXX | 07/07/2014 | CA | ASL 8 CAGLIARI | ovicaprini |
| 2014 | IT045CAXXX | 07/07/2014 | CA | ASL 8 CAGLIARI | ovicaprini |
| 2014 | IT050NUXXX | 07/07/2014 | NU | ASL NUORO | ovicaprini |
| 2014 | IT069ORXXX | 07/07/2014 | OR | ASL ORISTANO | ovicaprini |
| 2014 | IT001CAXXX | 09/07/2014 | CA | ASL 8 CAGLIARI | ovicaprini |
| 2014 | IT029CAXXX | 09/07/2014 | CA | ASL 8 CAGLIARI | ovicaprini |
| 2014 | IT044ORXXX | 09/07/2014 | OR | ASL ORISTANO | ovicaprini |
| 2014 | IT057CAXXX | 09/07/2014 | CA | ASL 8 CAGLIARI | ovicaprini |
| 2014 | IT036NUXXX | 11/07/2014 | NU | ASL NUORO | ovicaprini |
| 2014 | IT065CAXXX | 14/07/2014 | CA | ASL 8 CAGLIARI | ovicaprini |
| 2014 | IT024CAXXX | 14/07/2014 | CA | ASL 8 CAGLIARI | ovicaprini |
| 2014 | IT048ORXXX | 14/07/2014 | OR | ASL ORISTANO | ovicaprini |
| 2014 | IT070ORXXX | 14/07/2014 | OR | ASL ORISTANO | ovicaprini |
| 2014 | IT069ORXXX | 14/07/2014 | OR | ASL ORISTANO | ovicaprini |
| 2014 | IT086CAXXX | 14/07/2014 | CA | ASL 8 CAGLIARI | ovicaprini |
| 2014 | IT045CAXXX | 14/07/2014 | CA | ASL 8 CAGLIARI | ovicaprini |
| 2014 | IT050NUXXX | 14/07/2014 | NU | ASL NUORO | ovicaprini |
| 2014 | IT031CAXXX | 14/07/2014 | CA | ASL 8 CAGLIARI | ovicaprini |
| 2014 | IT092CAXXX | 18/07/2014 | CA | ASL 8 CAGLIARI | ovicaprini |
| 2014 | IT065CAXXX | 21/07/2014 | CA | ASL 8 CAGLIARI | ovicaprini |
| 2014 | IT024CAXXX | 21/07/2014 | CA | ASL 8 CAGLIARI | ovicaprini |
| 2014 | IT057CAXXX | 21/07/2014 | CA | ASL 8 CAGLIARI | ovicaprini |
| 2014 | IT094CAXXX | 21/07/2014 | CA | ASL 8 CAGLIARI | ovicaprini |
| 2014 | IT039ORXXX | 21/07/2014 | OR | ASL ORISTANO | ovicaprini |
| 2014 | IT086CAXXX | 21/07/2014 | CA | ASL 8 CAGLIARI | ovicaprini |
| 2014 | IT091CAXXX | 21/07/2014 | CA | ASL 8 CAGLIARI | ovicaprini |
| 2014 | IT045CAXXX | 21/07/2014 | CA | ASL 8 CAGLIARI | ovicaprini |
| 2014 | IT048ORXXX | 21/07/2014 | OR | ASL ORISTANO | ovicaprini |
| 2014 | IT092CAXXX | 21/07/2014 | CA | ASL 8 CAGLIARI | ovicaprini |
| 2014 | IT060SSXXX | 23/07/2014 | SS | ASL 1 SASSARI | ovicaprini |
| 2014 | IT029CAXXX | 23/07/2014 | CA | ASL 8 CAGLIARI | ovicaprini |
| 2014 | IT055SSXXX | 25/07/2014 | SS | ASL 1 SASSARI | ovicaprini |
| 2014 | IT065CAXXX | 28/07/2014 | CA | ASL 8 CAGLIARI | ovicaprini |
| 2014 | IT001CAXXX | 28/07/2014 | CA | ASL 8 CAGLIARI | ovicaprini |
| 2014 | IT055SSXXX | 28/07/2014 | SS | ASL 1 SASSARI | ovicaprini |
| 2014 | IT086CAXXX | 28/07/2014 | CA | ASL 8 CAGLIARI | ovicaprini |
| 2014 | IT042ORXXX | 28/07/2014 | OR | ASL ORISTANO | ovicaprini |
| 2014 | IT094CAXXX | 28/07/2014 | CA | ASL 8 CAGLIARI | ovicaprini |
| 2014 | IT069ORXXX | 28/07/2014 | OR | ASL ORISTANO | ovicaprini |
| 2014 | IT090CAXXX | 28/07/2014 | CA | ASL 8 CAGLIARI | ovicaprini |
| 2014 | IT091CAXXX | 28/07/2014 | CA | ASL 8 CAGLIARI | ovicaprini |
| 2014 | IT086CAXXX | 30/07/2014 | CA | ASL 8 CAGLIARI | ovicaprini |
| 2014 | IT065CAXXX | 04/08/2014 | CA | ASL 8 CAGLIARI | ovicaprini |
| 2014 | IT024CAXXX | 04/08/2014 | CA | ASL 8 CAGLIARI | ovicaprini |
| 2014 | IT094CAXXX | 04/08/2014 | CA | ASL 8 CAGLIARI | ovicaprini |
| 2014 | IT094CAXXX | 04/08/2014 | CA | ASL 8 CAGLIARI | ovicaprini |
| 2014 | IT057ORXXX | 04/08/2014 | OR | ASL ORISTANO | ovicaprini |
| 2014 | IT035CAXXX | 04/08/2014 | CA | ASL 8 CAGLIARI | ovicaprini |
| 2014 | IT052CAXXX | 06/08/2014 | CA | ASL 8 CAGLIARI | ovicaprini |
| 2014 | IT091CAXXX | 06/08/2014 | CA | ASL 8 CAGLIARI | ovicaprini |
| 2014 | IT091CAXXX | 08/08/2014 | CA | ASL 8 CAGLIARI | ovicaprini |
| 2014 | IT057ORXXX | 08/08/2014 | OR | ASL ORISTANO | ovicaprini |
| 2014 | IT086CAXXX | 08/08/2014 | CA | ASL 8 CAGLIARI | ovicaprini |
| 2014 | IT086CAXXX | 08/08/2014 | CA | ASL 8 CAGLIARI | ovicaprini |
| 2014 | IT057ORXXX | 11/08/2014 | OR | ASL ORISTANO | ovicaprini |
| 2014 | IT012ORXXX | 11/08/2014 | OR | ASL ORISTANO | ovicaprini |
| 2014 | IT086CAXXX | 11/08/2014 | CA | ASL 8 CAGLIARI | ovicaprini |
| 2014 | IT039ORXXX | 11/08/2014 | OR | ASL ORISTANO | ovicaprini |
| 2014 | IT077CAXXX | 11/08/2014 | CA | ASL 8 CAGLIARI | ovicaprini |
| 2014 | IT057ORXXX | 11/08/2014 | OR | ASL ORISTANO | ovicaprini |
| 2014 | IT029ORXXX | 11/08/2014 | OR | ASL ORISTANO | ovicaprini |
| 2014 | IT077CAXXX | 11/08/2014 | CA | ASL 8 CAGLIARI | ovicaprini |
| 2014 | IT092CAXXX | 11/08/2014 | CA | ASL 8 CAGLIARI | ovicaprini |
| 2014 | IT029CAXXX | 11/08/2014 | CA | ASL 8 CAGLIARI | ovicaprini |
| 2014 | IT012ORXXX | 12/08/2014 | OR | ASL ORISTANO | ovicaprini |
| 2014 | IT029CAXXX | 13/08/2014 | CA | ASL 8 CAGLIARI | ovicaprini |
| 2014 | IT001CAXXX | 13/08/2014 | CA | ASL 8 CAGLIARI | ovicaprini |
| 2014 | IT054ORXXX | 13/08/2014 | OR | ASL ORISTANO | ovicaprini |
| 2014 | IT065CAXXX | 18/08/2014 | CA | ASL 8 CAGLIARI | ovicaprini |
| 2014 | IT001CAXXX | 18/08/2014 | CA | ASL 8 CAGLIARI | ovicaprini |
| 2014 | IT016CAXXX | 18/08/2014 | CA | ASL 8 CAGLIARI | ovicaprini |
| 2014 | IT012ORXXX | 18/08/2014 | OR | ASL ORISTANO | ovicaprini |
| 2014 | IT091CAXXX | 18/08/2014 | CA | ASL 8 CAGLIARI | ovicaprini |
| 2014 | IT046CAXXX | 27/08/2014 | CA | ASL 8 CAGLIARI | ovicaprini |
| 2014 | IT046CAXXX | 27/08/2014 | CA | ASL 8 CAGLIARI | ovicaprini |
| 2014 | IT046CAXXX | 27/08/2014 | CA | ASL 8 CAGLIARI | ovicaprini |
| 2014 | IT046CAXXX | 27/08/2014 | CA | ASL 8 CAGLIARI | ovicaprini |
| 2014 | IT065CAXXX | 01/09/2014 | CA | ASL 8 CAGLIARI | ovicaprini |
| 2014 | IT094CAXXX | 01/09/2014 | CA | ASL 8 CAGLIARI | ovicaprini |
| 2014 | IT055CAXXX | 01/09/2014 | CA | ASL 8 CAGLIARI | ovicaprini |
| 2014 | IT069ORXXX | 01/09/2014 | OR | ASL ORISTANO | ovicaprini |
| 2014 | IT035CAXXX | 03/09/2014 | CA | ASL 8 CAGLIARI | ovicaprini |
| 2014 | IT065CAXXX | 08/09/2014 | CA | ASL 8 CAGLIARI | ovicaprini |
| 2014 | IT012ORXXX | 08/09/2014 | OR | ASL ORISTANO | ovicaprini |
| 2014 | IT036CAXXX | 10/09/2014 | CA | ASL 8 CAGLIARI | ovicaprini |
| 2014 | IT065CAXXX | 15/09/2014 | CA | ASL 8 CAGLIARI | ovicaprini |
| 2014 | IT096CAXXX | 15/09/2014 | CA | ASL 8 CAGLIARI | ovicaprini |
| 2014 | IT096CAXXX | 15/09/2014 | CA | ASL 8 CAGLIARI | ovicaprini |
| 2014 | IT072CAXXX | 22/09/2014 | CA | ASL 8 CAGLIARI | ovicaprini |
| 2014 | IT065CAXXX | 29/09/2014 | CA | ASL 8 CAGLIARI | ovicaprini |
| 2014 | IT072CAXXX | 29/09/2014 | CA | ASL 8 CAGLIARI | ovicaprini |
| 2014 | IT092CAXXX | 10/10/2014 | CA | ASL 8 CAGLIARI | ovicaprini |
| 2014 | IT065CAXXX | 13/10/2014 | CA | ASL 8 CAGLIARI | ovicaprini |
| 2014 | IT026ORXXX | 13/10/2014 | OR | ASL ORISTANO | ovicaprini |
| 2014 | IT023CAXXX | 13/10/2014 | CA | ASL 8 CAGLIARI | ovicaprini |
| 2014 | IT023CAXXX | 13/10/2014 | CA | ASL 8 CAGLIARI | ovicaprini |
| 2014 | IT092CAXXX | 24/10/2014 | CA | ASL 8 CAGLIARI | ovicaprini |
| 2014 | IT070ORXXX | 29/10/2014 | OR | ASL ORISTANO | ovicaprini |
| 2014 | IT092CAXXX | 31/10/2014 | CA | ASL 8 CAGLIARI | ovicaprini |
| 2014 | IT069ORXXX | 10/11/2014 | OR | ASL ORISTANO | ovicaprini |
| 2014 | IT066CAXXX | 05/12/2014 | CA | ASL 8 CAGLIARI | ovicaprini |
| 2014 | IT052CAXXX | 18/12/2014 | CA | ASL 8 CAGLIARI | ovicaprini |
| 2014 | IT012CAXXX | 23/12/2014 | CA | ASL 8 CAGLIARI | ovicaprini |
| 2015 | 033CA10XXX | 08/01/2015 | CA | ASL 8 CAGLIARI | ovicaprini |
| 2015 | 012NU04XXX | 08/01/2015 | NU | ASL NUORO | ovicaprini |
| 2015 | 049OR13XXX | 08/01/2015 | OR | ASL ORISTANO | ovicaprini |
| 2015 | 044NU04XXX | 08/01/2015 | NU | ASL NUORO | ovicaprini |
| 2015 | 013SS11XXX | 09/01/2015 | SS | ASL 1 SASSARI | ovicaprini |
| 2015 | 030SS04XXX | 09/01/2015 | SS | ASL 1 SASSARI | ovicaprini |
| 2015 | 030SS07XXX | 09/01/2015 | SS | ASL 1 SASSARI | ovicaprini |
| 2015 | 013SS07XXX | 09/01/2015 | SS | ASL 1 SASSARI | ovicaprini |
| 2015 | 044NU16XXX | 09/01/2015 | NU | ASL NUORO | ovicaprini |
| 2015 | 051OR17XXX | 12/01/2015 | OR | ASL ORISTANO | ovicaprini |
| 2015 | 012NU05XXX | 15/01/2015 | NU | ASL NUORO | ovicaprini |
| 2015 | 049OR12XXX | 15/01/2015 | OR | ASL ORISTANO | ovicaprini |
| 2015 | 049OR12XXX | 15/01/2015 | OR | ASL ORISTANO | ovicaprini |
| 2015 | 064NU03XXX | 15/01/2015 | NU | ASL NUORO | ovicaprini |
| 2015 | 044NU08XXX | 16/01/2015 | NU | ASL NUORO | ovicaprini |
| 2015 | 049OR21XXX | 19/01/2015 | OR | ASL ORISTANO | ovicaprini |
| 2015 | 049OR01XXX | 19/01/2015 | OR | ASL ORISTANO | ovicaprini |
| 2015 | 063OR04XXX | 19/01/2015 | OR | ASL ORISTANO | ovicaprini |
| 2015 | 013SS08XXX | 19/01/2015 | SS | ASL 1 SASSARI | ovicaprini |
| 2015 | 049NU01XXX | 22/01/2015 | NU | ASL NUORO | ovicaprini |
| 2015 | 059SS06XXX | 22/01/2015 | SS | ASL 1 SASSARI | ovicaprini |
| 2015 | 064SS01XXX | 22/01/2015 | SS | ASL 1 SASSARI | ovicaprini |
| 2015 | 003SS05XXX | 22/01/2015 | SS | ASL 1 SASSARI | ovicaprini |
| 2015 | 064SS01XXX | 22/01/2015 | SS | ASL 1 SASSARI | ovicaprini |
| 2015 | 065SS68XXX | 22/01/2015 | SS | ASL 1 SASSARI | ovicaprini |
| 2015 | 064SSD4XXX | 22/01/2015 | SS | ASL 1 SASSARI | ovicaprini |
| 2015 | 064SS73XXX | 22/01/2015 | SS | ASL 1 SASSARI | ovicaprini |
| 2015 | 083NU15XXX | 23/01/2015 | NU | ASL NUORO | ovicaprini |
| 2015 | 092CA07XXX | 23/01/2015 | CA | ASL 8 CAGLIARI | ovicaprini |
| 2015 | 050SS04XXX | 26/01/2015 | SS | ASL 1 SASSARI | ovicaprini |
| 2015 | 057SS05XXX | 27/01/2015 | SS | ASL 1 SASSARI | ovicaprini |
| 2015 | 049OR21XXX | 27/01/2015 | OR | ASL ORISTANO | ovicaprini |
| 2015 | 078OR00XXX | 27/01/2015 | OR | ASL ORISTANO | ovicaprini |
| 2015 | 032CA00XXX | 29/01/2015 | CA | ASL 8 CAGLIARI | ovicaprini |
| 2015 | 077NU01XXX | 27/02/2015 | NU | ASL NUORO | ovicaprini |
| 2015 | 018NU03XXX | 27/02/2015 | NU | ASL NUORO | ovicaprini |
| 2015 | 009NU02XXX | 27/04/2015 | NU | ASL NUORO | ovicaprini |
| 2015 | 071SS07XXX | 02/02/2015 | SS | ASL 1 SASSARI | ovicaprini |
| 2015 | IT063NUXXX | 02/03/2015 | NU | ASL NUORO | ovicaprini |
| 2015 | IT009NUXXX | 02/03/2014 | NU | ASL NUORO | ovicaprini |
| 2015 | 044NU04XXX | 02/02/2015 | NU | ASL NUORO | ovicaprini |
| 2015 | 064SSD4XXX | 03/02/2015 | SS | ASL 1 SASSARI | ovicaprini |
| 2015 | 064SS73XXX | 03/02/2015 | SS | ASL 1 SASSARI | ovicaprini |
| 2015 | 005CA03XXX | 03/02/2015 | CA | ASL 8 CAGLIARI | ovicaprini |
| 2015 | 066SS06XXX | 05/02/2015 | SS | ASL 1 SASSARI | ovicaprini |
| 2015 | 057SS18XXX | 05/02/2015 | SS | ASL 1 SASSARI | ovicaprini |
| 2015 | 012NU14XXX | 05/02/2015 | NU | ASL NUORO | ovicaprini |
| 2015 | 014OR01XXX | 09/02/2015 | OR | ASL ORISTANO | ovicaprini |
| 2015 | 009NU01XXX | 09/02/2015 | NU | ASL NUORO | ovicaprini |
| 2015 | 057CA06XXX | 09/02/2015 | CA | ASL 8 CAGLIARI | ovicaprini |
| 2015 | 092CA01XXX | 09/02/2015 | CA | ASL 8 CAGLIARI | ovicaprini |
| 2015 | 078CA07XXX | 09/02/2015 | CA | ASL 8 CAGLIARI | ovicaprini |
| 2015 | 009NU18XXX | 09/02/2015 | NU | ASL NUORO | ovicaprini |
| 2015 | 029CA02XXX | 09/02/2015 | CA | ASL 8 CAGLIARI | ovicaprini |
| 2015 | 049OR04XXX | 10/02/2015 | OR | ASL ORISTANO | ovicaprini |
| 2015 | 049OR12XXX | 10/02/2015 | OR | ASL ORISTANO | ovicaprini |
| 2015 | 041SS06XXX | 10/02/2015 | SS | ASL 1 SASSARI | ovicaprini |
| 2015 | 049OR17XXX | 12/02/2015 | OR | ASL ORISTANO | ovicaprini |
| 2015 | 030SS01XXX | 12/02/2015 | SS | ASL 1 SASSARI | ovicaprini |
| 2015 | 013SS01XXX | 12/02/2015 | SS | ASL 1 SASSARI | ovicaprini |
| 2015 | 013SS04XXX | 12/02/2015 | SS | ASL 1 SASSARI | ovicaprini |
| 2015 | 012NU17XXX | 12/02/2015 | NU | ASL NUORO | ovicaprini |
| 2015 | 024NU01XXX | 12/02/2015 | NU | ASL NUORO | ovicaprini |
| 2015 | 009NU07XXX | 13/02/2015 | NU | ASL NUORO | ovicaprini |
| 2015 | 041SS44XXX | 13/02/2015 | SS | ASL 1 SASSARI | ovicaprini |
| 2015 | 047SS02XXX | 13/02/2015 | SS | ASL 1 SASSARI | ovicaprini |
| 2015 | 047SS37XXX | 13/02/2015 | SS | ASL 1 SASSARI | ovicaprini |
| 2015 | 078SS15XXX | 15/02/2015 | SS | ASL 1 SASSARI | ovicaprini |
| 2015 | 087NU06XXX | 16/02/2015 | NU | ASL NUORO | ovicaprini |
| 2015 | 029CA02XXX | 17/02/2015 | CA | ASL 8 CAGLIARI | ovicaprini |
| 2015 | 078CA18XXX | 17/02/2015 | CA | ASL 8 CAGLIARI | ovicaprini |
| 2015 | 020OR09XXX | 19/02/2015 | OR | ASL ORISTANO | ovicaprini |
| 2015 | 064SS65XXX | 19/02/2015 | SS | ASL 1 SASSARI | ovicaprini |
| 2015 | 038NU01XXX | 19/02/2015 | NU | ASL NUORO | ovicaprini |
| 2015 | 041OR14XXX | 20/02/2015 | OR | ASL ORISTANO | ovicaprini |
| 2015 | 060SS00XXX | 23/02/2015 | SS | ASL 1 SASSARI | ovicaprini |
| 2015 | 028SS17XXX | 23/02/2015 | SS | ASL 1 SASSARI | ovicaprini |
| 2015 | 013SS18XXX | 23/02/2015 | SS | ASL 1 SASSARI | ovicaprini |
| 2015 | 013SS01XXX | 24/02/2015 | SS | ASL 1 SASSARI | ovicaprini |
| 2015 | 030SS06XXX | 24/02/2015 | SS | ASL 1 SASSARI | ovicaprini |
| 2015 | 012NU04XXX | 24/02/2015 | NU | ASL NUORO | ovicaprini |
| 2015 | 001OR11XXX | 26/02/2015 | OR | ASL ORISTANO | ovicaprini |
| 2015 | 049OR17XXX | 26/02/2015 | OR | ASL ORISTANO | ovicaprini |
| 2015 | 044NU04XXX | 26/02/2015 | NU | ASL NUORO | ovicaprini |
| 2015 | 083NU04XXX | 26/02/2015 | NU | ASL NUORO | ovicaprini |
| 2015 | 042CA01XXX | 26/02/2015 | CA | ASL 8 CAGLIARI | ovicaprini |
| 2015 | 032CA10XXX | 26/02/2015 | CA | ASL 8 CAGLIARI | ovicaprini |
| 2015 | 064SS70XXX | 27/02/2015 | SS | ASL 1 SASSARI | ovicaprini |
| 2015 | 071SS07XXX | 27/02/2015 | SS | ASL 1 SASSARI | ovicaprini |
| 2015 | 064SS04XXX | 27/02/2015 | SS | ASL 1 SASSARI | ovicaprini |
| 2015 | 040SS01XXX | 03/03/2015 | SS | ASL 1 SASSARI | ovicaprini |
| 2015 | 088NU01XXX | 03/03/2015 | NU | ASL NUORO | ovicaprini |
| 2015 | 064SS65XXX | 03/03/2015 | SS | ASL 1 SASSARI | ovicaprini |
| 2015 | 057NU02XXX | 05/03/2015 | NU | ASL NUORO | ovicaprini |
| 2015 | 057NU02XXX | 05/03/2015 | NU | ASL NUORO | ovicaprini |
| 2015 | 083NU21XXX | 05/03/2015 | NU | ASL NUORO | ovicaprini |
| 2015 | 049SS27XXX | 05/03/2015 | SS | ASL 1 SASSARI | ovicaprini |
| 2015 | 049SS09XXX | 05/03/2015 | SS | ASL 1 SASSARI | ovicaprini |
| 2015 | 049SS19XXX | 05/03/2015 | SS | ASL 1 SASSARI | ovicaprini |
| 2015 | 075SS07XXX | 05/03/2015 | SS | ASL 1 SASSARI | ovicaprini |
| 2015 | 0212OR1XXX | 05/03/2015 | OR | ASL ORISTANO | ovicaprini |
| 2015 | 025OR18XXX | 05/03/2015 | OR | ASL ORISTANO | ovicaprini |
| 2015 | 049SS08XXX | 06/03/2015 | SS | ASL 1 SASSARI | ovicaprini |
| 2015 | 049SS23XXX | 06/03/2015 | SS | ASL 1 SASSARI | ovicaprini |
| 2015 | 044NU04XXX | 09/03/2015 | NU | ASL NUORO | ovicaprini |
| 2015 | 008NU01XXX | 09/03/2015 | NU | ASL NUORO | ovicaprini |
| 2015 | 008NU01XXX | 09/03/2015 | NU | ASL NUORO | ovicaprini |
| 2015 | 083NU15XXX | 09/03/2015 | NU | ASL NUORO | ovicaprini |
| 2015 | 083NU15XXX | 09/03/2015 | NU | ASL NUORO | ovicaprini |
| 2015 | 012NU04XXX | 10/03/2015 | NU | ASL NUORO | ovicaprini |
| 2015 | 027SS02XXX | 10/03/2015 | SS | ASL 1 SASSARI | ovicaprini |
| 2015 | 011SS02XXX | 10/03/2015 | SS | ASL 1 SASSARI | ovicaprini |
| 2015 | 101CA02XXX | 10/03/2015 | CA | ASL 8 CAGLIARI | ovicaprini |
| 2015 | 101CA02XXX | 10/03/2015 | CA | ASL 8 CAGLIARI | ovicaprini |
| 2015 | 058SS10XXX | 12/03/2015 | SS | ASL 1 SASSARI | ovicaprini |
| 2015 | 013SS11XXX | 12/03/2015 | SS | ASL 1 SASSARI | ovicaprini |
| 2015 | 011NU00XXX | 12/03/2015 | NU | ASL NUORO | ovicaprini |
| 2015 | 030SS01XXX | 16/03/2015 | SS | ASL 1 SASSARI | ovicaprini |
| 2015 | 030SS01XXX | 16/03/2015 | SS | ASL 1 SASSARI | ovicaprini |
| 2015 | 035CA01XXX | 16/03/2015 | CA | ASL 8 CAGLIARI | ovicaprini |
| 2015 | 033OR04XXX | 16/03/2015 | OR | ASL ORISTANO | ovicaprini |
| 2015 | 049OR13XXX | 16/03/2015 | OR | ASL ORISTANO | ovicaprini |
| 2015 | 059SS06XXX | 17/03/2015 | SS | ASL 1 SASSARI | ovicaprini |
| 2015 | 058SS02XXX | 17/03/2015 | SS | ASL 1 SASSARI | ovicaprini |
| 2015 | 001OR15XXX | 17/03/2015 | OR | ASL ORISTANO | ovicaprini |
| 2015 | 049OR13XXX | 17/03/2015 | OR | ASL ORISTANO | ovicaprini |
| 2015 | 012NU06XXX | 17/03/2015 | NU | ASL NUORO | ovicaprini |
| 2015 | 029CA02XXX | 17/03/2015 | CA | ASL 8 CAGLIARI | ovicaprini |
| 2015 | 003SS05XXX | 19/03/2015 | SS | ASL 1 SASSARI | ovicaprini |
| 2015 | 064SS75XXX | 19/03/2015 | SS | ASL 1 SASSARI | ovicaprini |
| 2015 | 059SS06XXX | 19/03/2015 | SS | ASL 1 SASSARI | ovicaprini |
| 2015 | 064SS68XXX | 20/03/2015 | SS | ASL 1 SASSARI | ovicaprini |
| 2015 | 003SS05XXX | 20/03/2015 | SS | ASL 1 SASSARI | ovicaprini |
| 2015 | 049OR04XXX | 20/03/2015 | OR | ASL ORISTANO | ovicaprini |
| 2015 | 087OR01XXX | 20/03/2015 | OR | ASL ORISTANO | ovicaprini |
| 2015 | 019OR19XXX | 23/03/2015 | OR | ASL ORISTANO | ovicaprini |
| 2015 | 078CA04XXX | 23/03/2015 | CA | ASL 8 CAGLIARI | ovicaprini |
| 2015 | 012NU08XXX | 23/03/2015 | NU | ASL NUORO | ovicaprini |
| 2015 | 051OR05XXX | 23/03/2015 | OR | ASL ORISTANO | ovicaprini |
| 2015 | 087OR01XXX | 24/03/2015 | OR | ASL ORISTANO | ovicaprini |
| 2015 | 091CA33XXX | 24/03/2015 | CA | ASL 8 CAGLIARI | ovicaprini |
| 2015 | 012NU14XXX | 25/03/2015 | NU | ASL NUORO | ovicaprini |
| 2015 | 016OR01XXX | 25/03/2015 | OR | ASL ORISTANO | ovicaprini |
| 2015 | 019OR20XXX | 25/03/2015 | OR | ASL ORISTANO | ovicaprini |
| 2015 | 016CA01XXX | 25/03/2015 | CA | ASL 8 CAGLIARI | ovicaprini |
| 2015 | 049OR04XXX | 01/04/2015 | OR | ASL ORISTANO | ovicaprini |
| 2015 | 001OR15XXX | 01/04/2015 | OR | ASL ORISTANO | ovicaprini |
| 2015 | 012NU14XXX | 01/04/2015 | NU | ASL NUORO | ovicaprini |
| 2015 | 078CA05XXX | 02/04/2015 | CA | ASL 8 CAGLIARI | ovicaprini |
| 2015 | 012NU14XXX | 03/04/2015 | NU | ASL NUORO | ovicaprini |
| 2015 | 029CA02XXX | 03/04/2015 | CA | ASL 8 CAGLIARI | ovicaprini |
| 2015 | 078CA13XXX | 07/04/2015 | CA | ASL 8 CAGLIARI | ovicaprini |
| 2015 | 008NU05XXX | 07/04/2015 | NU | ASL NUORO | ovicaprini |
| 2015 | 012NU14XXX | 07/04/2015 | NU | ASL NUORO | ovicaprini |
| 2015 | 019OR13XXX | 04/05/2015 | OR | ASL ORISTANO | ovicaprini |
| 2015 | 013SS06XXX | 04/05/2015 | SS | ASL 1 SASSARI | ovicaprini |
| 2015 | 063OR05XXX | 04/05/2015 | OR | ASL ORISTANO | ovicaprini |
| 2015 | 013SS14XXX | 04/05/2015 | SS | ASL 1 SASSARI | ovicaprini |
| 2015 | 026SS00XXX | 04/05/2015 | SS | ASL 1 SASSARI | ovicaprini |
| 2015 | 013SS24XXX | 04/05/2015 | SS | ASL 1 SASSARI | ovicaprini |
| 2015 | 012NU14XXX | 04/05/2015 | NU | ASL NUORO | ovicaprini |
| 2015 | 012NU14XXX | 05/05/2015 | NU | ASL NUORO | ovicaprini |
| 2015 | 012NU14XXX | 05/05/2015 | NU | ASL NUORO | ovicaprini |
| 2015 | 001OR00XXX | 05/05/2015 | OR | ASL ORISTANO | ovicaprini |
| 2015 | 083NU12XXX | 05/05/2015 | NU | ASL NUORO | ovicaprini |
| 2015 | 033OR08XXX | 05/05/2015 | OR | ASL ORISTANO | ovicaprini |
| 2015 | 029CA06XXX | 05/05/2015 | CA | ASL 8 CAGLIARI | ovicaprini |
| 2015 | 029CA06XXX | 05/05/2015 | CA | ASL 8 CAGLIARI | ovicaprini |
| 2015 | 057NU05XXX | 07/05/2015 | NU | ASL NUORO | ovicaprini |
| 2015 | 012NU14XXX | 07/05/2015 | NU | ASL NUORO | ovicaprini |
| 2015 | 065CA02XXX | 08/05/2015 | CA | ASL 8 CAGLIARI | ovicaprini |
| 2015 | 078CA02XXX | 08/05/2015 | CA | ASL 8 CAGLIARI | ovicaprini |
| 2015 | 025OR18XXX | 11/05/2015 | OR | ASL ORISTANO | ovicaprini |
| 2015 | 009NU07XXX | 11/05/2015 | NU | ASL NUORO | ovicaprini |
| 2015 | 008NU01XXX | 11/05/2015 | NU | ASL NUORO | ovicaprini |
| 2015 | 040CA04XXX | 12/05/2015 | CA | ASL 8 CAGLIARI | ovicaprini |
| 2015 | 024NU06XXX | 12/05/2015 | NU | ASL NUORO | ovicaprini |
| 2015 | 018NU11XXX | 12/05/2015 | NU | ASL NUORO | ovicaprini |
| 2015 | 078CA02XXX | 12/05/2015 | CA | ASL 8 CAGLIARI | ovicaprini |
| 2015 | 033OR01XXX | 12/05/2015 | OR | ASL ORISTANO | ovicaprini |
| 2015 | 064CA00XXX | 12/05/2015 | CA | ASL 8 CAGLIARI | ovicaprini |
| 2015 | 0269OR0XXX | 12/05/2015 | OR | ASL ORISTANO | ovicaprini |
| 2015 | 030SS01XXX | 12/05/2015 | SS | ASL 1 SASSARI | ovicaprini |
| 2015 | 024NU03XXX | 12/05/2015 | NU | ASL NUORO | ovicaprini |
| 2015 | 019OR14XXX | 14/05/2015 | OR | ASL ORISTANO | ovicaprini |
| 2015 | 012NU14XXX | 15/05/2015 | NU | ASL NUORO | ovicaprini |
| 2015 | 064SS18XXX | 15/05/2015 | SS | ASL 1 SASSARI | ovicaprini |
| 2015 | 083NU04XXX | 18/05/2015 | NU | ASL NUORO | ovicaprini |
| 2015 | 019OR04XXX | 18/05/2015 | OR | ASL ORISTANO | ovicaprini |
| 2015 | 017OR12XXX | 18/05/2015 | OR | ASL ORISTANO | ovicaprini |
| 2015 | 013SS01XXX | 18/05/2015 | SS | ASL 1 SASSARI | ovicaprini |
| 2015 | 066SS02XXX | 18/05/2015 | SS | ASL 1 SASSARI | ovicaprini |
| 2015 | 050NU06XXX | 19/05/2015 | NU | ASL NUORO | ovicaprini |
| 2015 | 030SS01XXX | 19/05/2015 | SS | ASL 1 SASSARI | ovicaprini |
| 2015 | 012NU14XXX | 19/05/2015 | NU | ASL NUORO | ovicaprini |
| 2015 | 008NU02XXX | 19/05/2015 | NU | ASL NUORO | ovicaprini |
| 2015 | 012NU14XXX | 19/05/2015 | NU | ASL NUORO | ovicaprini |
| 2015 | 049OR21XXX | 19/05/2015 | OR | ASL ORISTANO | ovicaprini |
| 2015 | 064SS61XXX | 19/05/2015 | SS | ASL 1 SASSARI | ovicaprini |
| 2015 | 050NU06XXX | 19/05/2015 | NU | ASL NUORO | ovicaprini |
| 2015 | 064SS40XXX | 19/05/2015 | SS | ASL 1 SASSARI | ovicaprini |
| 2015 | 0656NU0XXX | 21/05/2015 | NU | ASL NUORO | ovicaprini |
| 2015 | 019OR09XXX | 21/05/2015 | OR | ASL ORISTANO | ovicaprini |
| 2015 | 023NU00XXX | 21/05/2015 | NU | ASL NUORO | ovicaprini |
| 2015 | 012NU14XXX | 21/05/2015 | NU | ASL NUORO | ovicaprini |
| 2015 | 045OR16XXX | 21/05/2015 | OR | ASL ORISTANO | ovicaprini |
| 2015 | 064SSb3XXX | 22/05/2015 | SS | ASL 1 SASSARI | ovicaprini |
| 2015 | 064SS24XXX | 22/05/2015 | SS | ASL 1 SASSARI | ovicaprini |
| 2015 | 064SS61XXX | 22/05/2015 | SS | ASL 1 SASSARI | ovicaprini |
| 2015 | 009NU18XXX | 22/05/2015 | NU | ASL NUORO | ovicaprini |
| 2015 | 012NU14XXX | 22/05/2015 | NU | ASL NUORO | ovicaprini |
| 2015 | 012NU14XXX | 25/05/2015 | NU | ASL NUORO | ovicaprini |
| 2015 | 083NU02XXX | 25/05/2015 | NU | ASL NUORO | ovicaprini |
| 2015 | 001OR05XXX | 26/05/2015 | OR | ASL ORISTANO | ovicaprini |
| 2015 | 061SS06XXX | 26/05/2015 | SS | ASL 1 SASSARI | ovicaprini |
| 2015 | 049OR07XXX | 26/05/2015 | OR | ASL ORISTANO | ovicaprini |
| 2015 | 041OR11XXX | 26/05/2015 | OR | ASL ORISTANO | ovicaprini |
| 2015 | 049OR13XXX | 26/05/2015 | OR | ASL ORISTANO | ovicaprini |
| 2015 | 049OR13XXX | 26/05/2015 | OR | ASL ORISTANO | ovicaprini |
| 2015 | 049OR21XXX | 26/05/2015 | OR | ASL ORISTANO | ovicaprini |
| 2015 | 057SS18XXX | 26/05/2015 | SS | ASL 1 SASSARI | ovicaprini |
| 2015 | 012NU14XXX | 28/05/2015 | NU | ASL NUORO | ovicaprini |
| 2015 | 102CA01XXX | 29/05/2015 | CA | ASL 8 CAGLIARI | ovicaprini |
| 2015 | 102CA01XXX | 29/05/2015 | CA | ASL 8 CAGLIARI | ovicaprini |
| 2015 | 029CA06XXX | 29/05/2015 | CA | ASL 8 CAGLIARI | ovicaprini |
| 2015 | 019OR21XXX | 29/05/2015 | OR | ASL ORISTANO | ovicaprini |
| 2015 | 019OR20XXX | 29/05/2015 | OR | ASL ORISTANO | ovicaprini |
| 2015 | 012NU14XXX | 29/05/2015 | NU | ASL NUORO | ovicaprini |
| 2015 | 015OR00XXX | 01/06/2015 | OR | ASL ORISTANO | ovicaprini |
| 2015 | 012NU14XXX | 01/06/2015 | NU | ASL NUORO | ovicaprini |
| 2015 | 011NU00XXX | 01/06/2015 | NU | ASL NUORO | ovicaprini |
| 2015 | 012NU14XXX | 03/06/2015 | NU | ASL NUORO | ovicaprini |
| 2015 | 071NU01XXX | 04/06/2015 | NU | ASL NUORO | ovicaprini |
| 2015 | 078SS17XXX | 04/06/2015 | SS | ASL 1 SASSARI | ovicaprini |
| 2015 | 010NU02XXX | 04/06/2015 | NU | ASL NUORO | ovicaprini |
| 2015 | 060SS00XXX | 04/06/2015 | SS | ASL 1 SASSARI | ovicaprini |
| 2015 | 012NU14XXX | 04/06/2015 | NU | ASL NUORO | ovicaprini |
| 2015 | 012NU14XXX | 05/06/2015 | NU | ASL NUORO | ovicaprini |
| 2015 | 009NU07XXX | 05/06/2015 | NU | ASL NUORO | ovicaprini |
| 2015 | 049OR12XXX | 05/06/2015 | OR | ASL ORISTANO | ovicaprini |
| 2015 | 012NU14XXX | 08/06/2015 | NU | ASL NUORO | ovicaprini |
| 2015 | 012NU14XXX | 09/06/2015 | NU | ASL NUORO | ovicaprini |
| 2015 | 012NU14XXX | 11/06/2015 | NU | ASL NUORO | ovicaprini |
| 2015 | 083NU02XXX | 11/06/2015 | NU | ASL NUORO | ovicaprini |
| 2015 | 041OR16XXX | 12/06/2015 | OR | ASL ORISTANO | ovicaprini |
| 2015 | 012NU14XXX | 12/06/2015 | NU | ASL NUORO | ovicaprini |
| 2015 | 024CA04XXX | 12/06/2015 | CA | ASL 8 CAGLIARI | ovicaprini |
| 2015 | 011SS02XXX | 12/06/2015 | SS | ASL 1 SASSARI | ovicaprini |
| 2015 | 065CA02XXX | 12/06/2015 | CA | ASL 8 CAGLIARI | ovicaprini |
| 2015 | 083NU04XXX | 12/06/2015 | NU | ASL NUORO | ovicaprini |
| 2015 | 051OR00XXX | 15/06/2015 | OR | ASL ORISTANO | ovicaprini |
| 2015 | 010NU08XXX | 15/06/2015 | NU | ASL NUORO | ovicaprini |
| 2015 | 012NU14XXX | 16/06/2015 | NU | ASL NUORO | ovicaprini |
| 2015 | 052OR32XXX | 16/06/2015 | OR | ASL ORISTANO | ovicaprini |
| 2015 | 042SS05XXX | 16/06/2015 | SS | ASL 1 SASSARI | ovicaprini |
| 2015 | 064NU05XXX | 19/06/2015 | NU | ASL NUORO | ovicaprini |
| 2015 | 035CA01XXX | 16/06/2015 | CA | ASL 8 CAGLIARI | ovicaprini |
| 2015 | 052OR08XXX | 18/06/2015 | OR | ASL ORISTANO | ovicaprini |
| 2015 | 012NU14XXX | 18/06/2015 | NU | ASL NUORO | ovicaprini |
| 2015 | 064SS52XXX | 22/06/2015 | SS | ASL 1 SASSARI | ovicaprini |
| 2015 | 012NU14XXX | 22/06/2015 | NU | ASL NUORO | ovicaprini |
| 2015 | 008NU02XXX | 22/06/2015 | NU | ASL NUORO | ovicaprini |
| 2015 | 050SS02XXX | 22/06/2015 | SS | ASL 1 SASSARI | ovicaprini |
| 2015 | 049OR09XXX | 23/06/2015 | OR | ASL ORISTANO | ovicaprini |
| 2015 | 092CA15XXX | 23/06/2015 | CA | ASL 8 CAGLIARI | ovicaprini |
| 2015 | 012NU14XXX | 23/06/2015 | NU | ASL NUORO | ovicaprini |
| 2015 | 012NU14XXX | 25/06/2015 | NU | ASL NUORO | ovicaprini |
| 2015 | 029SS09XXX | 25/06/2015 | SS | ASL 1 SASSARI | ovicaprini |
| 2015 | 020OR09XXX | 25/06/2015 | OR | ASL ORISTANO | ovicaprini |
| 2015 | 012NU14XXX | 26/06/2015 | NU | ASL NUORO | ovicaprini |
| 2015 | 078CA09XXX | 26/06/2015 | CA | ASL 8 CAGLIARI | ovicaprini |
| 2015 | 012NU14XXX | 29/06/2015 | NU | ASL NUORO | ovicaprini |
| 2015 | 012NU14XXX | 30/06/2015 | NU | ASL NUORO | ovicaprini |
| 2015 | 045OR07XXX | 30/06/2015 | OR | ASL ORISTANO | ovicaprini |
| 2015 | 045OR15XXX | 30/06/2015 | OR | ASL ORISTANO | ovicaprini |
| 2015 | 063OR06XXX | 30/06/2015 | OR | ASL ORISTANO | ovicaprini |
| 2015 | 064SS38XXX | 02/07/2015 | SS | ASL 1 SASSARI | ovicaprini |
| 2015 | 064SS26XXX | 02/07/2015 | SS | ASL 1 SASSARI | ovicaprini |
| 2015 | 064SS65XXX | 02/07/2015 | SS | ASL 1 SASSARI | ovicaprini |
| 2015 | 064SS08XXX | 02/07/2015 | SS | ASL 1 SASSARI | ovicaprini |
| 2015 | 064SS38XXX | 02/07/2015 | SS | ASL 1 SASSARI | ovicaprini |
| 2015 | 044NU04XXX | 02/07/2015 | NU | ASL NUORO | ovicaprini |
| 2015 | 051OR01XXX | 03/07/2015 | OR | ASL ORISTANO | ovicaprini |
| 2015 | 051OR04XXX | 03/07/2015 | OR | ASL ORISTANO | ovicaprini |
| 2015 | 012NU14XXX | 03/07/2015 | NU | ASL NUORO | ovicaprini |
| 2015 | 051OR04XXX | 03/07/2015 | OR | ASL ORISTANO | ovicaprini |
| 2015 | 064OR00XXX | 06/07/2015 | OR | ASL ORISTANO | ovicaprini |
| 2015 | 012NU14XXX | 07/07/2015 | NU | ASL NUORO | ovicaprini |
| 2015 | 019OR08XXX | 07/07/2015 | OR | ASL ORISTANO | ovicaprini |
| 2015 | 064SS01XXX | 09/07/2015 | SS | ASL 1 SASSARI | ovicaprini |
| 2015 | 010NU07XXX | 09/07/2015 | NU | ASL NUORO | ovicaprini |
| 2015 | 064SS40XXX | 09/07/2015 | SS | ASL 1 SASSARI | ovicaprini |
| 2015 | 064SS09XXX | 09/07/2015 | SS | ASL 1 SASSARI | ovicaprini |
| 2015 | 064SS68XXX | 10/07/2015 | SS | ASL 1 SASSARI | ovicaprini |
| 2015 | 064SS65XXX | 10/07/2015 | SS | ASL 1 SASSARI | ovicaprini |
| 2015 | 058SS10XXX | 10/07/2015 | SS | ASL 1 SASSARI | ovicaprini |
| 2015 | 084NU06XXX | 13/07/2015 | NU | ASL NUORO | ovicaprini |
| 2015 | 078SS04XXX | 13/07/2015 | SS | ASL 1 SASSARI | ovicaprini |
| 2015 | 059SS09XXX | 13/07/2015 | SS | ASL 1 SASSARI | ovicaprini |
| 2015 | 059SS14XXX | 13/07/2015 | SS | ASL 1 SASSARI | ovicaprini |
| 2015 | 045CA01XXX | 13/07/2015 | CA | ASL 8 CAGLIARI | ovicaprini |
| 2015 | 049OR13XXX | 14/07/2015 | OR | ASL ORISTANO | ovicaprini |
| 2015 | 049OR13XXX | 14/07/2015 | OR | ASL ORISTANO | ovicaprini |
| 2015 | 078CA02XXX | 14/07/2015 | CA | ASL 8 CAGLIARI | ovicaprini |
| 2015 | 101CA02XXX | 14/07/2015 | CA | ASL 8 CAGLIARI | ovicaprini |
| 2015 | 084NU05XXX | 16/07/2015 | NU | ASL NUORO | ovicaprini |
| 2015 | 044NU05XXX | 16/07/2015 | NU | ASL NUORO | ovicaprini |
| 2015 | 012NU14XXX | 17/07/2015 | NU | ASL NUORO | ovicaprini |
| 2015 | 071SS07XXX | 20/07/2015 | SS | ASL 1 SASSARI | ovicaprini |
| 2015 | 012NU14XXX | 20/07/2015 | NU | ASL NUORO | ovicaprini |
| 2015 | 012NU14XXX | 21/07/2015 | NU | ASL NUORO | ovicaprini |
| 2015 | 012NU14XXX | 23/07/2015 | NU | ASL NUORO | ovicaprini |
| 2015 | 062NU27XXX | 24/07/2015 | NU | ASL NUORO | ovicaprini |
| 2015 | 049OR23XXX | 24/07/2015 | OR | ASL ORISTANO | ovicaprini |
| 2015 | 011NU00XXX | 27/07/2015 | NU | ASL NUORO | ovicaprini |
| 2015 | 064SS09XXX | 27/07/2015 | SS | ASL 1 SASSARI | ovicaprini |
| 2015 | 012NU14XXX | 27/07/2015 | NU | ASL NUORO | ovicaprini |
| 2015 | 012NU14XXX | 28/07/2015 | NU | ASL NUORO | ovicaprini |
| 2015 | 012NU14XXX | 30/07/2015 | NU | ASL NUORO | ovicaprini |
| 2015 | 050SS15XXX | 31/07/2015 | SS | ASL 1 SASSARI | ovicaprini |
| 2015 | 013SS13XXX | 31/07/2015 | SS | ASL 1 SASSARI | ovicaprini |
| 2015 | 084NU04XXX | 07/08/2015 | NU | ASL NUORO | ovicaprini |
| 2015 | 027SS01XXX | 27/08/2015 | SS | ASL 1 SASSARI | ovicaprini |
| 2015 | 059SS09XXX | 10/09/2015 | SS | ASL 1 SASSARI | ovicaprini |
| 2015 | 033OR04XXX | 21/09/2015 | OR | ASL ORISTANO | ovicaprini |
| 2015 | 013SS04XXX | 27/12/2015 | SS | ASL 1 SASSARI | ovicaprini |
