# Supplementary material for: Prevalence estimation of Italian ovine cystic echinococcosis in slaughterhouses: A retrospective Bayesian data analysis, 2010–2015
Source: PLoS One. 2019 Apr 1;14(4):e0214224. doi: 10.1371/journal.pone.0214224 (PMC6443144; doi:10.1371/journal.pone.0214224)
Supplement: S3 Table — (DOCX) [file pone.0214224.s004.docx]

**S3 Table. Ad hoc database for Italian farms resulted CE positive, including year of observation, positive farm code (encripped), data of slaughter, province and Local Sanitary Agency code, species.**

| **REGION** | **ASL** | **data_slaught** | **Specie** | **positive_farm_code** |
| --- | --- | --- | --- | --- |
| EMILIA-ROMAGNA | A.USL FORLI' | 28/06/2010 | OVICAPRINI | 019FXXX |
| EMILIA-ROMAGNA | A.USL CESENA | 05/05/2010 | OVICAPRINI | 020FXXX |
| EMILIA-ROMAGNA | A.USL FORLI' | 07/05/2010 | OVICAPRINI | 036FXXX |
| EMILIA-ROMAGNA | A.USL PARMA | 26/10/2011 | OVICAPRINI | 026PXXX |
| EMILIA-ROMAGNA | A.USL PARMA | 26/10/2011 | OVICAPRINI | 022PXXX |
| EMILIA-ROMAGNA | A.USL PARMA | 22/08/2011 | OVICAPRINI | 022PXXX |
| EMILIA-ROMAGNA | A.USL PARMA | 22/08/2011 | OVICAPRINI | 022PXXX |
| EMILIA-ROMAGNA | A.USL PARMA | 03/08/2011 | OVICAPRINI | 026PXXX |
| EMILIA-ROMAGNA | A.USL PARMA | 23/05/2011 | OVICAPRINI | 031PXXX |
| EMILIA-ROMAGNA | A.USL FORLI' | 14/03/2011 | OVICAPRINI | 019FXXX |
| EMILIA-ROMAGNA | A.USL CESENA | 19/01/2011 | OVICAPRINI | 007FXXX |
| EMILIA-ROMAGNA | A.USL CESENA | 07/03/2011 | OVICAPRINI | 046FXXX |
| EMILIA-ROMAGNA | A.USL CESENA | 24/03/2011 | OVICAPRINI | 046FXXX |
| EMILIA-ROMAGNA | A.USL RIMINI | 04/04/2011 | OVICAPRINI | 042PXXX |
| EMILIA-ROMAGNA | A.USL FERRARA | 31/12/2012 | OVICAPRINI | 012FXXX |
| EMILIA-ROMAGNA | A.USL FORLI' | 17/01/2012 | OVICAPRINI | 012FXXX |
| EMILIA-ROMAGNA | A.USL CESENA | 22/05/2012 | OVICAPRINI | 050FXXX |
| EMILIA-ROMAGNA | A.USL CESENA | 22/05/2012 | OVICAPRINI | 046FXXX |
| EMILIA-ROMAGNA | A.USL CESENA | 28/02/2012 | OVICAPRINI | 001FXXX |
| EMILIA-ROMAGNA | A.USL RIMINI | 19/03/2012 | OVICAPRINI | 042PXXX |
| EMILIA-ROMAGNA | A.USL FORLI' | 25/02/2012 | OVICAPRINI | 012FXXX |
| EMILIA-ROMAGNA | A.USL REGGIO EMILIA | 30/07/2013 | OVICAPRINI | 012RXXX |
| EMILIA-ROMAGNA | A.USL FERRARA | 31/12/2013 | OVICAPRINI | 012FXXX |
| EMILIA-ROMAGNA | A.USL CESENA | 16/04/2013 | OVICAPRINI | 001FXXX |
| EMILIA-ROMAGNA | A.USL RIMINI | 13/05/2013 | OVICAPRINI | 042PXXX |
| EMILIA-ROMAGNA | A.USL FERRARA | 09/05/2014 | OVICAPRINI | 012FXXX |
| EMILIA-ROMAGNA | A.USL FORLI' | 17/04/2014 | OVICAPRINI | 043FXXX |
| EMILIA-ROMAGNA | A.USL FORLI' | 17/04/2014 | OVICAPRINI | 003FXXX |
| EMILIA-ROMAGNA | A.USL RIMINI | 26/03/2014 | OVICAPRINI | 042PXXX |
| EMILIA-ROMAGNA | A.USL CESENA | 29/04/2015 | OVICAPRINI | 007FXXX |
| CAMPANIA | CASERTA | 21/11/2014 | OVICAPRINI | 087sXXX |
| CAMPANIA | SALERNO | 28/11/2014 | OVICAPRINI | 112sXXX |
| CAMPANIA | SALERNO | 03/12/2014 | OVICAPRINI | 140sXXX |
| CAMPANIA | SALERNO | 09/12/2014 | OVICAPRINI | 140sXXX |
| CAMPANIA | SALERNO | 09/12/2014 | OVICAPRINI | 025sXXX |
| CAMPANIA | SALERNO | 18/12/2014 | OVICAPRINI | 053pXXX |
| CAMPANIA | CASERTA | 05/11/2014 | OVICAPRINI | 033cXXX |
| CAMPANIA | CASERTA | 19/11/2014 | OVICAPRINI | 022cXXX |
| CAMPANIA | CASERTA | 03/12/2014 | OVICAPRINI | 014cXXX |
| CAMPANIA | CASERTA | 13/06/2012 | OVICAPRINI | 003CXXX |
| CAMPANIA | CASERTA | 13/08/2012 | OVICAPRINI | 003CXXX |
| CAMPANIA | SALERNO | 12/01/2015 | OVICAPRINI | 155SXXX |
| CAMPANIA | SALERNO | 12/01/2015 | OVICAPRINI | 140SXXX |
| CAMPANIA | SALERNO | 15/01/2015 | OVICAPRINI | 059PXXX |
| CAMPANIA | SALERNO | 16/01/2015 | OVICAPRINI | 084PXXX |
| CAMPANIA | SALERNO | 16/01/2015 | OVICAPRINI | 112SXXX |
| CAMPANIA | CASERTA | 28/01/2015 | OVICAPRINI | 002BXXX |
| CAMPANIA | SALERNO | 02/02/2015 | OVICAPRINI | 120SXXX |
| CAMPANIA | SALERNO | 02/02/2015 | OVICAPRINI | 120SXXX |
| CAMPANIA | SALERNO | 06/02/2015 | OVICAPRINI | 140SXXX |
| CAMPANIA | SALERNO | 09/02/2015 | OVICAPRINI | 083SXXX |
| CAMPANIA | CASERTA | 11/02/2015 | OVICAPRINI | 003CXXX |
| CAMPANIA | SALERNO | 12/02/2015 | OVICAPRINI | 140SXXX |
| CAMPANIA | SALERNO | 16/02/2015 | OVICAPRINI | 120SXXX |
| CAMPANIA | SALERNO | 18/02/2015 | OVICAPRINI | 080SXXX |
| CAMPANIA | SALERNO | 19/02/2015 | OVICAPRINI | 012PXXX |
| CAMPANIA | SALERNO | 19/02/2015 | OVICAPRINI | 012PXXX |
| CAMPANIA | SALERNO | 19/02/2015 | OVICAPRINI | 059PXXX |
| CAMPANIA | SALERNO | 26/02/2015 | OVICAPRINI | 105SXXX |
| CAMPANIA | SALERNO | 26/02/2015 | OVICAPRINI | 120SXXX |
| CAMPANIA | SALERNO | 26/02/2015 | OVICAPRINI | 043SXXX |
| CAMPANIA | SALERNO | 26/02/2015 | OVICAPRINI | 053PXXX |
| CAMPANIA | SALERNO | 26/02/2015 | OVICAPRINI | 105SXXX |
| CAMPANIA | SALERNO | 02/03/2015 | OVICAPRINI | 017AXXX |
| CAMPANIA | SALERNO | 02/03/2015 | OVICAPRINI | 046SXXX |
| CAMPANIA | SALERNO | 02/03/2015 | OVICAPRINI | 059PXXX |
| CAMPANIA | SALERNO | 02/03/2015 | OVICAPRINI | 035AXXX |
| CAMPANIA | CASERTA | 02/03/2015 | OVICAPRINI | 076CXXX |
| CAMPANIA | SALERNO | 13/03/2015 | OVICAPRINI | 025SXXX |
| CAMPANIA | SALERNO | 21/03/2015 | OVICAPRINI | 008mXXX |
| CAMPANIA | SALERNO | 21/03/2015 | OVICAPRINI | 034SXXX |
| CAMPANIA | SALERNO | 23/03/2015 | OVICAPRINI | 059PXXX |
| CAMPANIA | CASERTA | 25/03/2015 | OVICAPRINI | 003CXXX |
| CAMPANIA | SALERNO | 26/03/2015 | OVICAPRINI | 089SXXX |
| CAMPANIA | SALERNO | 30/03/2015 | OVICAPRINI | 053PXXX |
| CAMPANIA | AVELLINO | 31/03/2015 | OVICAPRINI | 108AXXX |
| CAMPANIA | CASERTA | 31/03/2015 | OVICAPRINI | 059CXXX |
| CAMPANIA | SALERNO | 09/04/2015 | OVICAPRINI | 110SXXX |
| CAMPANIA | SALERNO | 10/04/2015 | OVICAPRINI | 092AXXX |
| CAMPANIA | SALERNO | 13/04/2015 | OVICAPRINI | 076PXXX |
| CAMPANIA | SALERNO | 13/04/2015 | OVICAPRINI | 076PXXX |
| CAMPANIA | SALERNO | 13/04/2015 | OVICAPRINI | 046SXXX |
| CAMPANIA | SALERNO | 15/04/2015 | OVICAPRINI | 036AXXX |
| CAMPANIA | AVELLINO | 17/04/2015 | OVICAPRINI | 015AXXX |
| CAMPANIA | SALERNO | 20/04/2015 | OVICAPRINI | 071PXXX |
| CAMPANIA | SALERNO | 22/04/2015 | OVICAPRINI | 026mXXX |
| CAMPANIA | SALERNO | 04/05/2015 | OVICAPRINI | 019tXXX |
| CAMPANIA | SALERNO | 07/05/2015 | OVICAPRINI | 033SXXX |
| CAMPANIA | SALERNO | 07/05/2015 | OVICAPRINI | 014AXXX |
| CAMPANIA | SALERNO | 07/05/2015 | OVICAPRINI | 084PXXX |
| CAMPANIA | SALERNO | 11/05/2015 | OVICAPRINI | 089PXXX |
| CAMPANIA | SALERNO | 11/05/2015 | OVICAPRINI | 019tXXX |
| CAMPANIA | SALERNO | 11/05/2015 | OVICAPRINI | 013tXXX |
| CAMPANIA | SALERNO | 11/05/2015 | OVICAPRINI | 059PXXX |
| CAMPANIA | SALERNO | 14/05/2015 | OVICAPRINI | 008PXXX |
| CAMPANIA | SALERNO | 14/05/2015 | OVICAPRINI | 017SXXX |
| CAMPANIA | SALERNO | 20/05/2015 | OVICAPRINI | 037SXXX |
| CAMPANIA | SALERNO | 21/05/2015 | OVICAPRINI | 017SXXX |
| CAMPANIA | SALERNO | 21/05/2015 | OVICAPRINI | 012SXXX |
| CAMPANIA | SALERNO | 21/05/2015 | OVICAPRINI | 053SXXX |
| CAMPANIA | SALERNO | 21/05/2015 | OVICAPRINI | 053PXXX |
| CAMPANIA | SALERNO | 25/05/2015 | OVICAPRINI | 081cXXX |
| CAMPANIA | AVELLINO | 29/05/2015 | OVICAPRINI | 017AXXX |
| CAMPANIA | SALERNO | 30/05/2015 | OVICAPRINI | 051SXXX |
| CAMPANIA | SALERNO | 01/06/2015 | OVICAPRINI | 105SXXX |
| CAMPANIA | SALERNO | 01/06/2015 | OVICAPRINI | 032PXXX |
| CAMPANIA | SALERNO | 04/06/2015 | OVICAPRINI | 095SXXX |
| CAMPANIA | SALERNO | 04/06/2015 | OVICAPRINI | 053PXXX |
| CAMPANIA | SALERNO | 10/06/2015 | OVICAPRINI | 003SXXX |
| CAMPANIA | SALERNO | 10/06/2015 | OVICAPRINI | 092AXXX |
| CAMPANIA | SALERNO | 10/06/2015 | OVICAPRINI | 040AXXX |
| CAMPANIA | SALERNO | 11/06/2015 | OVICAPRINI | 105SXXX |
| CAMPANIA | AVELLINO | 12/06/2015 | OVICAPRINI | 004AXXX |
| CAMPANIA | SALERNO | 12/06/2015 | OVICAPRINI | 037SXXX |
| CAMPANIA | SALERNO | 12/06/2015 | OVICAPRINI | 027tXXX |
| CAMPANIA | SALERNO | 19/06/2015 | OVICAPRINI | 023bXXX |
| CAMPANIA | SALERNO | 22/06/2015 | OVICAPRINI | 112SXXX |
| CAMPANIA | SALERNO | 22/06/2015 | OVICAPRINI | 095SXXX |
| CAMPANIA | SALERNO | 22/06/2015 | OVICAPRINI | 106SXXX |
| CAMPANIA | SALERNO | 22/06/2015 | OVICAPRINI | 143SXXX |
| CAMPANIA | SALERNO | 22/06/2015 | OVICAPRINI | 140SXXX |
| CAMPANIA | SALERNO | 25/06/2015 | OVICAPRINI | 053PXXX |
| CAMPANIA | SALERNO | 25/06/2015 | OVICAPRINI | 155SXXX |
| CAMPANIA | SALERNO | 25/06/2015 | OVICAPRINI | 043SXXX |
| CAMPANIA | SALERNO | 25/06/2015 | OVICAPRINI | 120SXXX |
| CAMPANIA | SALERNO | 25/06/2015 | OVICAPRINI | 043SXXX |
| CAMPANIA | SALERNO | 25/06/2015 | OVICAPRINI | 008PXXX |
| CAMPANIA | SALERNO | 29/06/2015 | OVICAPRINI | 096PXXX |
| CAMPANIA | SALERNO | 29/06/2015 | OVICAPRINI | 146SXXX |
| CAMPANIA | SALERNO | 01/07/2015 | OVICAPRINI | 075SXXX |
| CAMPANIA | SALERNO | 01/07/2015 | OVICAPRINI | 138SXXX |
| CAMPANIA | SALERNO | 01/07/2015 | OVICAPRINI | 010SXXX |
| CAMPANIA | SALERNO | 01/07/2015 | OVICAPRINI | 136SXXX |
| CAMPANIA | SALERNO | 01/07/2015 | OVICAPRINI | 075SXXX |
| CAMPANIA | SALERNO | 01/07/2015 | OVICAPRINI | 126SXXX |
| CAMPANIA | SALERNO | 01/07/2015 | OVICAPRINI | 071SXXX |
| CAMPANIA | SALERNO | 01/07/2015 | OVICAPRINI | 136SXXX |
| CAMPANIA | SALERNO | 01/07/2015 | OVICAPRINI | 022SXXX |
| CAMPANIA | CASERTA | 01/07/2015 | OVICAPRINI | 074BXXX |
| CAMPANIA | SALERNO | 09/07/2015 | OVICAPRINI | 008PXXX |
| CAMPANIA | SALERNO | 13/07/2015 | OVICAPRINI | 155SXXX |
| CAMPANIA | SALERNO | 13/07/2015 | OVICAPRINI | 064SXXX |
| CAMPANIA | SALERNO | 13/07/2015 | OVICAPRINI | 095SXXX |
| CAMPANIA | SALERNO | 13/07/2015 | OVICAPRINI | 061SXXX |
| CAMPANIA | SALERNO | 13/07/2015 | OVICAPRINI | 070SXXX |
| CAMPANIA | SALERNO | 13/07/2015 | OVICAPRINI | 048SXXX |
| CAMPANIA | AVELLINO | 15/07/2015 | OVICAPRINI | 041AXXX |
| CAMPANIA | SALERNO | 15/07/2015 | OVICAPRINI | 129SXXX |
| CAMPANIA | AVELLINO | 17/07/2015 | OVICAPRINI | 015AXXX |
| CAMPANIA | SALERNO | 17/07/2015 | OVICAPRINI | 087SXXX |
| CAMPANIA | SALERNO | 17/07/2015 | OVICAPRINI | 120SXXX |
| CAMPANIA | SALERNO | 17/07/2015 | OVICAPRINI | 105SXXX |
| CAMPANIA | SALERNO | 17/07/2015 | OVICAPRINI | 022SXXX |
| CAMPANIA | SALERNO | 17/07/2015 | OVICAPRINI | 050SXXX |
| CAMPANIA | SALERNO | 20/07/2015 | OVICAPRINI | 004PXXX |
| CAMPANIA | SALERNO | 22/07/2015 | OVICAPRINI | 048SXXX |
| CAMPANIA | SALERNO | 22/07/2015 | OVICAPRINI | 073SXXX |
| CAMPANIA | SALERNO | 22/07/2015 | OVICAPRINI | 086SXXX |
| CAMPANIA | SALERNO | 22/07/2015 | OVICAPRINI | 017SXXX |
| CAMPANIA | SALERNO | 22/07/2015 | OVICAPRINI | 120SXXX |
| CAMPANIA | SALERNO | 22/07/2015 | OVICAPRINI | 110SXXX |
| CAMPANIA | SALERNO | 29/07/2015 | OVICAPRINI | 037SXXX |
| CAMPANIA | SALERNO | 29/07/2015 | OVICAPRINI | 073SXXX |
| CAMPANIA | SALERNO | 29/07/2015 | OVICAPRINI | 072SXXX |
| CAMPANIA | SALERNO | 29/07/2015 | OVICAPRINI | 040SXXX |
| CAMPANIA | SALERNO | 29/07/2015 | OVICAPRINI | 079SXXX |
| CAMPANIA | CASERTA | 29/07/2015 | OVICAPRINI | 038BXXX |
| CAMPANIA | SALERNO | 31/07/2015 | OVICAPRINI | 012PXXX |
| CAMPANIA | SALERNO | 03/08/2015 | OVICAPRINI | 028SXXX |
| CAMPANIA | SALERNO | 03/08/2015 | OVICAPRINI | 027SXXX |
| CAMPANIA | SALERNO | 03/08/2015 | OVICAPRINI | 027SXXX |
| CAMPANIA | SALERNO | 03/08/2015 | OVICAPRINI | 040SXXX |
| CAMPANIA | CASERTA | 03/08/2015 | OVICAPRINI | 034CXXX |
| CAMPANIA | SALERNO | 05/08/2015 | OVICAPRINI | 024cXXX |
| CAMPANIA | SALERNO | 06/08/2015 | OVICAPRINI | 017SXXX |
| CAMPANIA | SALERNO | 06/08/2015 | OVICAPRINI | 059PXXX |
| CAMPANIA | AVELLINO | 10/08/2015 | OVICAPRINI | 040AXXX |
| CAMPANIA | AVELLINO | 11/08/2015 | OVICAPRINI | 029CXXX |
| CAMPANIA | SALERNO | 13/08/2015 | OVICAPRINI | 120SXXX |
| CAMPANIA | CASERTA | 20/08/2015 | OVICAPRINI | 008PXXX |
| CAMPANIA | SALERNO | 20/08/2015 | OVICAPRINI | 008PXXX |
| CAMPANIA | SALERNO | 20/08/2015 | OVICAPRINI | 008PXXX |
| CAMPANIA | CASERTA | 21/08/2015 | OVICAPRINI | 064BXXX |
| CAMPANIA | SALERNO | 21/08/2015 | OVICAPRINI | 115SXXX |
| CAMPANIA | SALERNO | 21/08/2015 | OVICAPRINI | 126SXXX |
| CAMPANIA | SALERNO | 21/08/2015 | OVICAPRINI | 058SXXX |
| CAMPANIA | SALERNO | 21/08/2015 | OVICAPRINI | 002SXXX |
| CAMPANIA | SALERNO | 24/08/2015 | OVICAPRINI | 009PXXX |
| CAMPANIA | SALERNO | 27/08/2015 | OVICAPRINI | 105SXXX |
| CAMPANIA | SALERNO | 27/08/2015 | OVICAPRINI | 096PXXX |
| CAMPANIA | SALERNO | 28/08/2015 | OVICAPRINI | 105SXXX |
| CAMPANIA | SALERNO | 02/09/2015 | OVICAPRINI | 129SXXX |
| CAMPANIA | SALERNO | 02/09/2015 | OVICAPRINI | 058SXXX |
| CAMPANIA | SALERNO | 02/09/2015 | OVICAPRINI | 024SXXX |
| CAMPANIA | CASERTA | 02/09/2015 | OVICAPRINI | 075BXXX |
| CAMPANIA | SALERNO | 04/09/2015 | OVICAPRINI | 041PXXX |
| CAMPANIA | SALERNO | 04/09/2015 | OVICAPRINI | 014PXXX |
| CAMPANIA | SALERNO | 09/09/2015 | OVICAPRINI | 019SXXX |
| CAMPANIA | SALERNO | 21/09/2015 | OVICAPRINI | 027SXXX |
| CAMPANIA | SALERNO | 21/09/2015 | OVICAPRINI | 133SXXX |
| CAMPANIA | SALERNO | 21/09/2015 | OVICAPRINI | 059PXXX |
| CAMPANIA | SALERNO | 21/09/2015 | OVICAPRINI | 060SXXX |
| CAMPANIA | SALERNO | 21/09/2015 | OVICAPRINI | 081SXXX |
| CAMPANIA | SALERNO | 25/09/2015 | OVICAPRINI | 006SXXX |
| CAMPANIA | SALERNO | 28/09/2015 | OVICAPRINI | 054SXXX |
| CAMPANIA | SALERNO | 30/09/2015 | OVICAPRINI | 019SXXX |
| CAMPANIA | SALERNO | 30/09/2015 | OVICAPRINI | 098SXXX |
| CAMPANIA | SALERNO | 02/10/2015 | OVICAPRINI | 003nXXX |
| CAMPANIA | SALERNO | 05/10/2015 | OVICAPRINI | 079AXXX |
| CAMPANIA | CASERTA | 05/10/2015 | OVICAPRINI | 063CXXX |
| CAMPANIA | SALERNO | 08/10/2015 | OVICAPRINI | 059PXXX |
| CAMPANIA | SALERNO | 08/10/2015 | OVICAPRINI | 053PXXX |
| CAMPANIA | SALERNO | 08/10/2015 | OVICAPRINI | 053PXXX |
| CAMPANIA | SALERNO | 08/10/2015 | OVICAPRINI | 059PXXX |
| CAMPANIA | SALERNO | 14/10/2015 | OVICAPRINI | 101SXXX |
| CAMPANIA | SALERNO | 14/10/2015 | OVICAPRINI | 024SXXX |
| CAMPANIA | SALERNO | 14/10/2015 | OVICAPRINI | 101SXXX |
| CAMPANIA | SALERNO | 15/10/2015 | OVICAPRINI | 012PXXX |
| CAMPANIA | SALERNO | 19/10/2015 | OVICAPRINI | 076SXXX |
| CAMPANIA | SALERNO | 19/10/2015 | OVICAPRINI | 017BXXX |
| CAMPANIA | CASERTA | 21/10/2015 | OVICAPRINI | 034CXXX |
| CAMPANIA | SALERNO | 23/10/2015 | OVICAPRINI | 113SXXX |
| CAMPANIA | SALERNO | 23/10/2015 | OVICAPRINI | 025SXXX |
| CAMPANIA | SALERNO | 23/10/2015 | OVICAPRINI | 023SXXX |
| CAMPANIA | SALERNO | 26/10/2015 | OVICAPRINI | 061SXXX |
| CAMPANIA | SALERNO | 29/10/2015 | OVICAPRINI | 059PXXX |
| CAMPANIA | SALERNO | 29/10/2015 | OVICAPRINI | 059PXXX |
| CAMPANIA | SALERNO | 29/10/2015 | OVICAPRINI | 008PXXX |
| CAMPANIA | SALERNO | 02/11/2015 | OVICAPRINI | 048cXXX |
| CAMPANIA | SALERNO | 04/11/2015 | OVICAPRINI | 031SXXX |
| CAMPANIA | SALERNO | 11/11/2015 | OVICAPRINI | 017SXXX |
| CAMPANIA | SALERNO | 13/11/2015 | OVICAPRINI | 087SXXX |
| CAMPANIA | SALERNO | 13/11/2015 | OVICAPRINI | 081SXXX |
| CAMPANIA | SALERNO | 13/11/2015 | OVICAPRINI | 003SXXX |
| CAMPANIA | SALERNO | 16/11/2015 | OVICAPRINI | 013tXXX |
| CAMPANIA | SALERNO | 14/12/2015 | OVICAPRINI | 010SXXX |
| CAMPANIA | SALERNO | 23/12/2015 | OVICAPRINI | 059pXXX |
| CAMPANIA | SALERNO | 23/12/2015 | OVICAPRINI | 035aXXX |
| PIEMONTE | ASL TO 5 | 11/01/2012 | OVICAPRINI | 078TXXX |
| PIEMONTE | ASL TO 6 | 20/02/2012 | OVICAPRINI | IT00XXX |
| PIEMONTE | ASL TO 7 | 20/02/2012 | OVICAPRINI | IT15XXX |
| PIEMONTE | ASL TO 8 | 20/02/2012 | OVICAPRINI | IT15XXX |
| PIEMONTE | ASL TO 9 | 20/02/2012 | OVICAPRINI | IT00XXX |
| PIEMONTE | ASL CN 1 | 14/03/2012 | OVICAPRINI | 157CXXX |
| PIEMONTE | ASL TO 3 | 02/04/2012 | OVICAPRINI | 070TXXX |
| PIEMONTE | ASL TO 3 | 13/04/2012 | OVICAPRINI | 099TXXX |
| PIEMONTE | ASL CN 1 | 07/05/2012 | OVICAPRINI | 203CXXX |
| PIEMONTE | ASL CN 1 | 04/06/2012 | OVICAPRINI | 187CXXX |
| PIEMONTE | ASL CN 1 | 02/07/2012 | OVICAPRINI | 090CXXX |
| PIEMONTE | ASL CN 1 | 02/07/2012 | OVICAPRINI | 090CXXX |
| PIEMONTE | ASL CN 1 | 17/09/2012 | OVICAPRINI | 090CXXX |
| PIEMONTE | ASL CN 1 | 09/01/2012 | OVICAPRINI | 090CXXX |
| PIEMONTE | ASL CN 1 | 09/01/2012 | OVICAPRINI | 090CXXX |
| PIEMONTE | ASL AT | 09/01/2012 | OVICAPRINI | 052AXXX |
| PIEMONTE | ASL CN 1 | 09/01/2012 | OVICAPRINI | 090CXXX |
| PIEMONTE | ASL CN 1 | 09/01/2012 | OVICAPRINI | 090CXXX |
| PIEMONTE | ASL CN 1 | 20/02/2012 | OVICAPRINI | 123CXXX |
| PIEMONTE | ASL CN 1 | 01/02/2012 | OVICAPRINI | 009CXXX |
| PIEMONTE | ASL TO 3 | 06/02/2012 | OVICAPRINI | 099TXXX |
| PIEMONTE | ASL TO 3 | 06/02/2012 | OVICAPRINI | 099TXXX |
| PIEMONTE | ASL TO 3 | 22/02/2012 | OVICAPRINI | 070TXXX |
| PIEMONTE | ASL TO 3 | 09/03/2012 | OVICAPRINI | 070TXXX |
| PIEMONTE | ASL TO 5 | 09/03/2012 | OVICAPRINI | 197TXXX |
| PIEMONTE | ASL TO 3 | 02/04/2012 | OVICAPRINI | 070TXXX |
| PIEMONTE | ASL CN 1 | 02/04/2012 | OVICAPRINI | 090CXXX |
| PIEMONTE | ASL CN 1 | 07/05/2012 | OVICAPRINI | 128CXXX |
| PIEMONTE | ASL TO 3 | 14/05/2012 | OVICAPRINI | 275TXXX |
| PIEMONTE | ASL CN 1 | 21/05/2012 | OVICAPRINI | 090CXXX |
| PIEMONTE | ASL CN 1 | 21/05/2012 | OVICAPRINI | 090CXXX |
| PIEMONTE | ASL CN 1 | 21/05/2012 | OVICAPRINI | 090CXXX |
| PIEMONTE | ASL CN 1 | 21/05/2012 | OVICAPRINI | 090CXXX |
| PIEMONTE | ASL TO 3 | 04/06/2012 | OVICAPRINI | 070TXXX |
| PIEMONTE | ASL CN 1 | 29/06/2012 | OVICAPRINI | 209CXXX |
| PIEMONTE | ASL TO 3 | 29/06/2012 | OVICAPRINI | 139TXXX |
| PIEMONTE | ASL CN 1 | 02/07/2012 | OVICAPRINI | 090CXXX |
| PIEMONTE | ASL CN 1 | 13/07/2012 | OVICAPRINI | 090CXXX |
| PIEMONTE | ASL CN 1 | 13/07/2012 | OVICAPRINI | 090CXXX |
| PIEMONTE | ASL CN 1 | 13/07/2012 | OVICAPRINI | 090CXXX |
| PIEMONTE | ASL CN 1 | 13/07/2012 | OVICAPRINI | 090CXXX |
| PIEMONTE | ASL CN 1 | 08/08/2012 | OVICAPRINI | 090CXXX |
| PIEMONTE | ASL CN 1 | 10/08/2012 | OVICAPRINI | 090CXXX |
| PIEMONTE | ASL CN 1 | 13/08/2012 | OVICAPRINI | 090CXXX |
| PIEMONTE | ASL CN 1 | 13/08/2012 | OVICAPRINI | 090CXXX |
| PIEMONTE | ASL TO 3 | 03/09/2012 | OVICAPRINI | 070TXXX |
| PIEMONTE | ASL TO 3 | 03/09/2012 | OVICAPRINI | 070TXXX |
| PIEMONTE | ASL CN 1 | 10/09/2012 | OVICAPRINI | 009CXXX |
| PIEMONTE | ASL TO 3 | 17/09/2012 | OVICAPRINI | 070TXXX |
| PIEMONTE | ASL CN 1 | 17/09/2012 | OVICAPRINI | 090CXXX |
| PIEMONTE | ASL CN 1 | 17/09/2012 | OVICAPRINI | 090CXXX |
| PIEMONTE | ASL CN 1 | 17/09/2012 | OVICAPRINI | 090CXXX |
| PIEMONTE | ASL CN 1 | 24/09/2012 | OVICAPRINI | 090CXXX |
| PIEMONTE | ASL CN 1 | 28/09/2012 | OVICAPRINI | 090CXXX |
| PIEMONTE | ASL CN 1 | 12/10/2012 | OVICAPRINI | 090CXXX |
| PIEMONTE | ASL CN 1 | 01/10/2012 | OVICAPRINI | 203CXXX |
| PIEMONTE | ASL CN 1 | 01/10/2012 | OVICAPRINI | 090CXXX |
| PIEMONTE | ASL CN 1 | 01/10/2012 | OVICAPRINI | 090CXXX |
| PIEMONTE | ASL CN 1 | 01/10/2012 | OVICAPRINI | 090CXXX |
| PIEMONTE | ASL CN 1 | 08/10/2012 | OVICAPRINI | 211CXXX |
| PIEMONTE | ASL TO 3 | 15/10/2012 | OVICAPRINI | 070TXXX |
| PIEMONTE | ASL TO 3 | 17/10/2012 | OVICAPRINI | 191TXXX |
| PIEMONTE | ASL CN 1 | 22/10/2012 | OVICAPRINI | 203CXXX |
| PIEMONTE | ASL TO 3 | 22/10/2012 | OVICAPRINI | 306TXXX |
| PIEMONTE | ASL CN 1 | 22/10/2012 | OVICAPRINI | 203CXXX |
| PIEMONTE | ASL AT | 29/10/2012 | OVICAPRINI | 101AXXX |
| PIEMONTE | ASL CN 1 | 29/10/2012 | OVICAPRINI | 090CXXX |
| PIEMONTE | ASL TO 3 | 31/10/2012 | OVICAPRINI | 191TXXX |
| PIEMONTE | ASL TO 3 | 31/10/2012 | OVICAPRINI | 306TXXX |
| PIEMONTE | ASL TO 3 | 05/11/2012 | OVICAPRINI | 070TXXX |
| PIEMONTE | ASL TO 3 | 07/11/2012 | OVICAPRINI | 142TXXX |
| PIEMONTE | ASL TO 3 | 14/11/2012 | OVICAPRINI | 070TXXX |
| PIEMONTE | ASL CN 1 | 19/11/2012 | OVICAPRINI | 090CXXX |
| PIEMONTE | ASL TO 3 | 23/11/2012 | OVICAPRINI | 191TXXX |
| PIEMONTE | ASL TO 3 | 28/11/2012 | OVICAPRINI | 142TXXX |
| PIEMONTE | ASL TO 3 | 28/11/2012 | OVICAPRINI | 142TXXX |
| PIEMONTE | ASL TO 3 | 10/12/2012 | OVICAPRINI | 049TXXX |
| PIEMONTE | ASL TO 3 | 10/12/2012 | OVICAPRINI | 049TXXX |
| PIEMONTE | ASL TO 3 | 09/01/2013 | OVICAPRINI | 300TXXX |
| PIEMONTE | ASL CN 1 | 09/01/2013 | OVICAPRINI | 082CXXX |
| PIEMONTE | ASL TO 3 | 04/02/2013 | OVICAPRINI | 142TXXX |
| PIEMONTE | ASL TO 3 | 04/02/2013 | OVICAPRINI | 142TXXX |
| PIEMONTE | ASL TO 3 | 25/02/2013 | OVICAPRINI | 070TXXX |
| PIEMONTE | ASL CN 1 | 25/02/2013 | OVICAPRINI | 078CXXX |
| PIEMONTE | ASL CN 1 | 13/03/2013 | OVICAPRINI | 203CXXX |
| PIEMONTE | ASL TO 3 | 28/03/2013 | OVICAPRINI | 191TXXX |
| PIEMONTE | ASL TO 3 | 03/04/2013 | OVICAPRINI | 191TXXX |
| PIEMONTE | ASL TO 3 | 12/04/2013 | OVICAPRINI | 142TXXX |
| PIEMONTE | ASL CN 1 | 19/04/2013 | OVICAPRINI | 090CXXX |
| PIEMONTE | ASL TO 3 | 03/05/2013 | OVICAPRINI | 191TXXX |
| PIEMONTE | ASL TO 3 | 03/05/2013 | OVICAPRINI | 041TXXX |
| PIEMONTE | ASL TO 3 | 10/05/2013 | OVICAPRINI | 070TXXX |
| PIEMONTE | ASL TO 3 | 13/05/2013 | OVICAPRINI | 026TXXX |
| PIEMONTE | ASL TO 3 | 13/05/2013 | OVICAPRINI | 070TXXX |
| PIEMONTE | ASL TO 5 | 20/05/2013 | OVICAPRINI | 078TXXX |
| PIEMONTE | ASL TO 3 | 22/05/2013 | OVICAPRINI | 139TXXX |
| PIEMONTE | ASL TO 3 | 03/06/2013 | OVICAPRINI | 070TXXX |
| PIEMONTE | ASL TO 3 | 05/06/2013 | OVICAPRINI | 026TXXX |
| PIEMONTE | ASL TO 3 | 10/06/2013 | OVICAPRINI | 041TXXX |
| PIEMONTE | ASL TO 3 | 17/06/2013 | OVICAPRINI | 025TXXX |
| PIEMONTE | ASL TO 3 | 12/08/2013 | OVICAPRINI | 191TXXX |
| PIEMONTE | ASL TO 3 | 12/08/2013 | OVICAPRINI | 025TXXX |
| PIEMONTE | ASL CN 1 | 08/07/2013 | OVICAPRINI | 203CXXX |
| PIEMONTE | ASL CN 1 | 23/08/2013 | OVICAPRINI | 034CXXX |
| PIEMONTE | ASL CN 1 | 23/08/2013 | OVICAPRINI | 034CXXX |
| PIEMONTE | ASL TO 3 | 09/09/2013 | OVICAPRINI | 070TXXX |
| PIEMONTE | ASL TO 3 | 09/09/2013 | OVICAPRINI | 070TXXX |
| PIEMONTE | ASL TO 3 | 11/09/2013 | OVICAPRINI | 191TXXX |
| PIEMONTE | ASL CN 1 | 16/09/2013 | OVICAPRINI | 203CXXX |
| PIEMONTE | ASL CN 1 | 16/09/2013 | OVICAPRINI | 203CXXX |
| PIEMONTE | ASL CN 1 | 25/09/2013 | OVICAPRINI | 090CXXX |
| PIEMONTE | ASL CN 1 | 30/09/2013 | OVICAPRINI | 090CXXX |
| PIEMONTE | ASL CN 1 | 16/10/2013 | OVICAPRINI | 090CXXX |
| PIEMONTE | ASL CN 1 | 21/10/2013 | OVICAPRINI | 090CXXX |
| PIEMONTE | ASL TO 3 | 23/10/2013 | OVICAPRINI | 070TXXX |
| PIEMONTE | ASL TO 3 | 28/10/2013 | OVICAPRINI | 142TXXX |
| PIEMONTE | ASL TO 3 | 06/11/2013 | OVICAPRINI | 142TXXX |
| PIEMONTE | ASL TO 3 | 13/11/2013 | OVICAPRINI | 035TXXX |
| PIEMONTE | ASL CN 1 | 18/11/2013 | OVICAPRINI | 090CXXX |
| PIEMONTE | ASL CN 1 | 18/11/2013 | OVICAPRINI | 090CXXX |
| PIEMONTE | ASL CN 1 | 18/11/2013 | OVICAPRINI | 009CXXX |
| PIEMONTE | ASL TO 3 | 29/11/2013 | OVICAPRINI | 275TXXX |
| PIEMONTE | ASL TO 3 | 09/12/2013 | OVICAPRINI | 191TXXX |
| PIEMONTE | ASL TO 3 | 03/02/2014 | OVICAPRINI | 306TXXX |
| PIEMONTE | ASL TO 3 | 03/02/2014 | OVICAPRINI | 306TXXX |
| PIEMONTE | ASL TO 3 | 17/03/2014 | OVICAPRINI | 275TXXX |
| PIEMONTE | ASL TO 3 | 28/03/2014 | OVICAPRINI | 306TXXX |
| PIEMONTE | ASL TO 3 | 02/04/2014 | OVICAPRINI | 142TXXX |
| PIEMONTE | ASL TO 3 | 02/04/2014 | OVICAPRINI | 142TXXX |
| PIEMONTE | ASL TO 3 | 22/04/2014 | OVICAPRINI | 099TXXX |
| PIEMONTE | ASL CN 1 | 30/04/2014 | OVICAPRINI | 203CXXX |
| PIEMONTE | ASL AT | 05/05/2014 | OVICAPRINI | 003AXXX |
| PIEMONTE | ASL CN 1 | 05/05/2014 | OVICAPRINI | 090CXXX |
| PIEMONTE | ASL CN 1 | 05/05/2014 | OVICAPRINI | 181CXXX |
| PIEMONTE | ASL CN 1 | 05/05/2014 | OVICAPRINI | 090CXXX |
| PIEMONTE | ASL CN 1 | 05/05/2014 | OVICAPRINI | 090CXXX |
| PIEMONTE | ASL TO 3 | 19/05/2014 | OVICAPRINI | 191TXXX |
| PIEMONTE | ASL TO 3 | 07/05/2014 | OVICAPRINI | 142TXXX |
| PIEMONTE | ASL TO 3 | 07/05/2014 | OVICAPRINI | 070TXXX |
| PIEMONTE | ASL CN 1 | 07/05/2014 | OVICAPRINI | 009CXXX |
| PIEMONTE | ASL CN 1 | 12/05/2014 | OVICAPRINI | 090CXXX |
| PIEMONTE | ASL TO 3 | 12/05/2014 | OVICAPRINI | 191TXXX |
| PIEMONTE | ASL CN 1 | 12/05/2014 | OVICAPRINI | 090CXXX |
| PIEMONTE | ASL TO 3 | 12/05/2014 | OVICAPRINI | 191TXXX |
| PIEMONTE | ASL TO 3 | 14/05/2014 | OVICAPRINI | 142TXXX |
| PIEMONTE | ASL TO 3 | 14/05/2014 | OVICAPRINI | 191TXXX |
| PIEMONTE | ASL TO 3 | 14/05/2014 | OVICAPRINI | 191TXXX |
| PIEMONTE | ASL CN 1 | 26/05/2014 | OVICAPRINI | 012CXXX |
| PIEMONTE | ASL TO 3 | 26/05/2014 | OVICAPRINI | 254TXXX |
| PIEMONTE | ASL TO 3 | 09/06/2014 | OVICAPRINI | 306TXXX |
| PIEMONTE | ASL TO 3 | 16/06/2014 | OVICAPRINI | 191TXXX |
| PIEMONTE | ASL AT | 23/06/2014 | OVICAPRINI | 003AXXX |
| PIEMONTE | ASL TO 3 | 25/06/2014 | OVICAPRINI | 191TXXX |
| PIEMONTE | ASL CN 1 | 30/06/2014 | OVICAPRINI | 203CXXX |
| PIEMONTE | ASL TO 3 | 30/06/2014 | OVICAPRINI | 306TXXX |
| PIEMONTE | ASL CN 1 | 07/07/2014 | OVICAPRINI | 203CXXX |
| PIEMONTE | ASL TO 3 | 09/07/2014 | OVICAPRINI | 306TXXX |
| PIEMONTE | ASL TO 3 | 09/07/2014 | OVICAPRINI | 142TXXX |
| PIEMONTE | ASL TO 3 | 11/07/2014 | OVICAPRINI | 011TXXX |
| PIEMONTE | ASL TO 3 | 14/07/2014 | OVICAPRINI | 142TXXX |
| PIEMONTE | ASL TO 3 | 14/07/2014 | OVICAPRINI | 011TXXX |
| PIEMONTE | ASL TO 3 | 14/07/2014 | OVICAPRINI | 306TXXX |
| PIEMONTE | ASL TO 3 | 21/07/2014 | OVICAPRINI | 011TXXX |
| PIEMONTE | ASL CN 1 | 21/07/2014 | OVICAPRINI | 090CXXX |
| PIEMONTE | ASL CN 1 | 21/07/2014 | OVICAPRINI | 090CXXX |
| PIEMONTE | ASL CN 1 | 21/07/2014 | OVICAPRINI | 090CXXX |
| PIEMONTE | ASL CN 2 | 05/08/2014 | OVICAPRINI | 090CXXX |
| PIEMONTE | ASL CN 1 | 01/08/2014 | OVICAPRINI | 090CXXX |
| PIEMONTE | ASL CN 1 | 01/08/2014 | OVICAPRINI | 090CXXX |
| PIEMONTE | ASL AT | 13/08/2014 | OVICAPRINI | 003AXXX |
| PIEMONTE | ASL AT | 13/08/2014 | OVICAPRINI | 003AXXX |
| PIEMONTE | ASL TO 5 | 05/09/2014 | OVICAPRINI | 078TXXX |
| PIEMONTE | ASL TO 3 | 24/09/2014 | OVICAPRINI | 142TXXX |
| PIEMONTE | ASL AT | 29/09/2014 | OVICAPRINI | 003AXXX |
| PIEMONTE | ASL CN 1 | 29/09/2014 | OVICAPRINI | 090CXXX |
| PIEMONTE | ASL CN 1 | 29/09/2014 | OVICAPRINI | 090CXXX |
| PIEMONTE | ASL CN 1 | 01/10/2014 | OVICAPRINI | 090CXXX |
| PIEMONTE | ASL TO 3 | 01/10/2014 | OVICAPRINI | 070TXXX |
| PIEMONTE | ASL TO 3 | 06/10/2014 | OVICAPRINI | 142TXXX |
| PIEMONTE | ASL TO 3 | 06/10/2014 | OVICAPRINI | 142TXXX |
| PIEMONTE | ASL TO 3 | 06/10/2014 | OVICAPRINI | 142TXXX |
| PIEMONTE | ASL TO 3 | 27/10/2014 | OVICAPRINI | 070TXXX |
| PIEMONTE | ASL TO 3 | 27/10/2014 | OVICAPRINI | 070TXXX |
| PIEMONTE | ASL TO 3 | 27/10/2014 | OVICAPRINI | 070TXXX |
| PIEMONTE | ASL TO 3 | 10/11/2014 | OVICAPRINI | 026TXXX |
| PIEMONTE | ASL TO 3 | 19/11/2014 | OVICAPRINI | 142TXXX |
| PIEMONTE | ASL TO 3 | 01/12/2014 | OVICAPRINI | 090TXXX |
| PIEMONTE | ASL TO 5 | 12/12/2014 | OVICAPRINI | 078TXXX |
| PIEMONTE | ASL TO 5 | 12/12/2014 | OVICAPRINI | 078TXXX |
| PIEMONTE | ASL TO 5 | 12/12/2014 | OVICAPRINI | 078TXXX |
| PIEMONTE | ASL TO 3 | 18/12/2014 | OVICAPRINI | 142TXXX |
| PIEMONTE | ASL TO 3 | 18/12/2014 | OVICAPRINI | 142TXXX |
| PIEMONTE | ASL CN 1 | 16/02/2015 | OVICAPRINI | 203CXXX |
| PIEMONTE | ASL CN 1 | 16/02/2015 | OVICAPRINI | 203CXXX |
| PIEMONTE | ASL CN 1 | 16/02/2015 | OVICAPRINI | 203CXXX |
| PIEMONTE | ASL CN 1 | 16/03/2015 | OVICAPRINI | 090CXXX |
| PIEMONTE | ASL CN 1 | 16/03/2015 | OVICAPRINI | 090CXXX |
| PIEMONTE | ASL CN 1 | 16/03/2015 | OVICAPRINI | 090CXXX |
| PIEMONTE | ASL CN 1 | 23/03/2015 | OVICAPRINI | 090CXXX |
| PIEMONTE | ASL TO 3 | 25/03/2015 | OVICAPRINI | 070TXXX |
| PIEMONTE | ASL TO 3 | 25/03/2015 | OVICAPRINI | 070TXXX |
| PIEMONTE | ASL CN 1 | 30/03/2015 | OVICAPRINI | 090CXXX |
| PIEMONTE | ASL TO 3 | 30/03/2015 | OVICAPRINI | 205TXXX |
| PIEMONTE | ASL CN 1 | 30/03/2015 | OVICAPRINI | 090CXXX |
| PIEMONTE | ASL CN 1 | 30/03/2015 | OVICAPRINI | 090CXXX |
| PIEMONTE | ASL CN 1 | 30/03/2015 | OVICAPRINI | 090CXXX |
| PIEMONTE | ASL TO 3 | 10/04/2015 | OVICAPRINI | 142TXXX |
| PIEMONTE | ASL CN 1 | 15/04/2015 | OVICAPRINI | 079CXXX |
| PIEMONTE | ASL CN 1 | 20/04/2015 | OVICAPRINI | 090CXXX |
| PIEMONTE | ASL CN 1 | 04/05/2015 | OVICAPRINI | 243CXXX |
| PIEMONTE | ASL CN 1 | 04/05/2015 | OVICAPRINI | 180CXXX |
| PIEMONTE | ASL CN 1 | 18/05/2015 | OVICAPRINI | 061CXXX |
| PIEMONTE | ASL CN 1 | 18/05/2015 | OVICAPRINI | 090CXXX |
| PIEMONTE | ASL CN 1 | 18/05/2015 | OVICAPRINI | 090CXXX |
| PIEMONTE | ASL TO 3 | 18/05/2015 | OVICAPRINI | 191TXXX |
| PIEMONTE | ASL TO 3 | 25/05/2015 | OVICAPRINI | 275TXXX |
| PIEMONTE | ASL CN 1 | 25/05/2015 | OVICAPRINI | 078CXXX |
| PIEMONTE | ASL TO 3 | 25/05/2015 | OVICAPRINI | 191TXXX |
| PIEMONTE | ASL TO 3 | 25/05/2015 | OVICAPRINI | 191TXXX |
| PIEMONTE | ASL TO 3 | 25/05/2015 | OVICAPRINI | 011TXXX |
| PIEMONTE | ASL CN 1 | 03/06/2015 | OVICAPRINI | 128CXXX |
| PIEMONTE | ASL TO 3 | 15/06/2015 | OVICAPRINI | 191TXXX |
| PIEMONTE | ASL CN 1 | 22/06/2015 | OVICAPRINI | 157CXXX |
| PIEMONTE | ASL CN 1 | 22/06/2015 | OVICAPRINI | 090CXXX |
| PIEMONTE | ASL CN 1 | 22/06/2015 | OVICAPRINI | 090CXXX |
| PIEMONTE | ASL CN 1 | 22/06/2015 | OVICAPRINI | 090CXXX |
| PIEMONTE | ASL CN 1 | 29/06/2015 | OVICAPRINI | 090CXXX |
| PIEMONTE | ASL CN 1 | 24/07/2015 | OVICAPRINI | 090CXXX |
| PIEMONTE | ASL CN 1 | 24/07/2015 | OVICAPRINI | 090CXXX |
| PIEMONTE | ASL AT | 24/07/2015 | OVICAPRINI | 003AXXX |
| PIEMONTE | ASL CN 1 | 12/08/2015 | OVICAPRINI | 090CXXX |
| PIEMONTE | ASL CN 1 | 12/08/2015 | OVICAPRINI | 090CXXX |
| PIEMONTE | ASL CN 1 | 12/08/2015 | OVICAPRINI | 090CXXX |
| PIEMONTE | ASL CN 1 | 12/08/2015 | OVICAPRINI | 090CXXX |
| PIEMONTE | ASL AT | 12/08/2015 | OVICAPRINI | 003AXXX |
| PIEMONTE | ASL CN 1 | 09/09/2015 | OVICAPRINI | 090CXXX |
| PIEMONTE | ASL TO 3 | 09/09/2015 | OVICAPRINI | 011TXXX |
| PIEMONTE | ASL TO 3 | 21/09/2015 | OVICAPRINI | 139TXXX |
| PIEMONTE | ASL CN 1 | 28/09/2015 | OVICAPRINI | 090CXXX |
| PIEMONTE | ASL CN 1 | 28/09/2015 | OVICAPRINI | 090CXXX |
| PIEMONTE | ASL CN 1 | 30/09/2015 | OVICAPRINI | 090CXXX |
| PIEMONTE | ASL CN 1 | 30/09/2015 | OVICAPRINI | 090CXXX |
| PIEMONTE | ASL CN 1 | 30/09/2015 | OVICAPRINI | 090CXXX |
| PIEMONTE | ASL TO 3 | 30/09/2015 | OVICAPRINI | 142TXXX |
| PIEMONTE | ASL CN 1 | 14/10/2015 | OVICAPRINI | 090CXXX |
| PIEMONTE | ASL CN 1 | 21/10/2015 | OVICAPRINI | 090CXXX |
| PIEMONTE | ASL CN 1 | 21/10/2015 | OVICAPRINI | 090CXXX |
| PIEMONTE | ASL CN 1 | 21/10/2015 | OVICAPRINI | 090CXXX |
| PIEMONTE | ASL CN 1 | 21/10/2015 | OVICAPRINI | 090CXXX |
| PIEMONTE | ASL CN 1 | 23/10/2015 | OVICAPRINI | 082CXXX |
| PIEMONTE | ASL TO 3 | 02/11/2015 | OVICAPRINI | 111TXXX |
| PIEMONTE | ASL CN 1 | 02/11/2015 | OVICAPRINI | 090CXXX |
| PIEMONTE | ASL CN 1 | 02/11/2015 | OVICAPRINI | 090CXXX |
| PIEMONTE | ASL TO 3 | 02/11/2015 | OVICAPRINI | 111TXXX |
| PIEMONTE | ASL CN 1 | 02/11/2015 | OVICAPRINI | 090CXXX |
| PIEMONTE | ASL CN 1 | 16/11/2015 | OVICAPRINI | 090TXXX |
| PIEMONTE | ASL CN 1 | 23/11/2015 | OVICAPRINI | 090CXXX |
| PIEMONTE | ASL CN 1 | 23/11/2015 | OVICAPRINI | 090CXXX |
| PIEMONTE | ASL TO 4 | 25/11/2015 | OVICAPRINI | 025VXXX |
| PIEMONTE | ASL CN 1 | 27/11/2015 | OVICAPRINI | 090CXXX |
| PIEMONTE | ASL CN 1 | 27/11/2015 | OVICAPRINI | 090CXXX |
| PIEMONTE | ASL CN 1 | 27/11/2015 | OVICAPRINI | 090CXXX |
| PIEMONTE | ASL CN 1 | 27/11/2015 | OVICAPRINI | 090CXXX |
| PIEMONTE | ASL AT | 27/11/2015 | OVICAPRINI | 003AXXX |
| PIEMONTE | ASL TO 3 | 11/11/2015 | OVICAPRINI | 142TXXX |
| PIEMONTE | ASL CN 1 | 11/11/2015 | OVICAPRINI | 090CXXX |
| PIEMONTE | ASL CN 1 | 11/11/2015 | OVICAPRINI | 090CXXX |
| PIEMONTE | ASL CN 1 | 11/11/2015 | OVICAPRINI | 090CXXX |
| PIEMONTE | ASL CN 1 | 11/11/2015 | OVICAPRINI | 090CXXX |
| PIEMONTE | ASL CN 1 | 11/11/2015 | OVICAPRINI | 090CXXX |
| PIEMONTE | ASL CN 1 | 11/11/2015 | OVICAPRINI | 090CXXX |
| PIEMONTE | ASL CN 1 | 11/11/2015 | OVICAPRINI | 209CXXX |
| PIEMONTE | ASL CN 1 | 11/12/2015 | OVICAPRINI | 090CXXX |
| PIEMONTE | ASL CN 1 | 11/12/2015 | OVICAPRINI | 090CXXX |
| PIEMONTE | ASL TO 3 | 14/12/2015 | OVICAPRINI | 142TXXX |
| PIEMONTE | ASL TO 3 | 17/12/2015 | OVICAPRINI | 205TXXX |
| PIEMONTE | ASL CN 1 | 28/12/2015 | OVICAPRINI | 090CXXX |
| PIEMONTE | ASL CN 1 | 30/12/2015 | OVICAPRINI | 090CXXX |
| PIEMONTE | ASL CN 1 | 04/01/2016 | OVICAPRINI | 090CXXX |
| PIEMONTE | ASL CN 1 | 04/01/2016 | OVICAPRINI | 090CXXX |
| PIEMONTE | ASL AT | 18/01/2016 | OVICAPRINI | 003AXXX |
| PIEMONTE | ASL TO 3 | 25/01/2016 | OVICAPRINI | 306TXXX |
| PIEMONTE | ASL TO 3 | 25/01/2016 | OVICAPRINI | 041TXXX |
| PIEMONTE | ASL TO 3 | 25/01/2016 | OVICAPRINI | 041TXXX |
| PIEMONTE | ASL CN 1 | 27/01/2016 | OVICAPRINI | 157CXXX |
| PIEMONTE | ASL TO 3 | 25/01/2016 | OVICAPRINI | 070TXXX |
| PIEMONTE | ASL CN 1 | 10/02/2016 | OVICAPRINI | 064CXXX |
| PIEMONTE | ASL CN 1 | 15/02/2016 | OVICAPRINI | 203CXXX |
| PIEMONTE | ASL CN 1 | 29/02/2016 | OVICAPRINI | 090CXXX |
| PIEMONTE | ASL CN 1 | 29/02/2016 | OVICAPRINI | 090CXXX |
| PIEMONTE | ASL CN 1 | 29/02/2016 | OVICAPRINI | 157CXXX |
| PIEMONTE | ASL CN 1 | 30/03/2016 | OVICAPRINI | 157CXXX |
| PIEMONTE | ASL CN 1 | 30/03/2016 | OVICAPRINI | 090CXXX |
| PIEMONTE | ASL CN 1 | 30/03/2016 | OVICAPRINI | 090CXXX |
| PIEMONTE | ASL CN 1 | 30/03/2016 | OVICAPRINI | 090CXXX |
| PIEMONTE | ASL CN 1 | 30/03/2016 | OVICAPRINI | 090CXXX |
| PIEMONTE | ASL CN 1 | 30/03/2016 | OVICAPRINI | 090CXXX |
| PIEMONTE | ASL CN 1 | 30/03/2016 | OVICAPRINI | 203CXXX |
| PIEMONTE | ASL TO 3 | 04/04/2016 | OVICAPRINI | 070TXXX |
| PIEMONTE | ASL TO 3 | 04/04/2016 | OVICAPRINI | 070TXXX |
| PIEMONTE | ASL TO 3 | 20/04/2016 | OVICAPRINI | 011TXXX |
| PIEMONTE | ASL TO 3 | 04/05/2016 | OVICAPRINI | 142TXXX |
| PIEMONTE | ASL TO 3 | 16/05/2016 | OVICAPRINI | 191TXXX |
| PIEMONTE | ASL TO 3 | 16/05/2016 | OVICAPRINI | 191TXXX |
| PIEMONTE | ASL TO 3 | 16/05/2016 | OVICAPRINI | 191TXXX |
| LOMBARDIA | BRESCIA | 15/01/2013 | OVICAPRINI | 143BXXX |
| LOMBARDIA | BRESCIA | 05/04/2012 | OVICAPRINI | 054BXXX |
| LOMBARDIA | BRESCIA | 08/11/2012 | OVICAPRINI | 143BXXX |
| LOMBARDIA | BRESCIA | 05/04/2012 | OVICAPRINI | 054BXXX |
| LOMBARDIA | BRESCIA | 02/04/2012 | OVICAPRINI | 016BXXX |
| LOMBARDIA | BRESCIA | 19/06/2012 | OVICAPRINI | 142BXXX |
| LOMBARDIA | BRESCIA | 19/06/2012 | OVICAPRINI | 142BXXX |
| LOMBARDIA | BRESCIA | 19/06/2012 | OVICAPRINI | 142BXXX |
| LOMBARDIA | BRESCIA | 16/07/2012 | OVICAPRINI | 166BXXX |
| LOMBARDIA | BRESCIA | 06/12/2012 | OVICAPRINI | 142BXXX |
| LOMBARDIA | BRESCIA | 06/12/2012 | OVICAPRINI | 142BXXX |
| LOMBARDIA | BRESCIA | 06/12/2012 | OVICAPRINI | 142BXXX |
| LOMBARDIA | BRESCIA | 03/04/2011 | OVICAPRINI | 016BXXX |
| LOMBARDIA | BRESCIA | 03/04/2011 | OVICAPRINI | 050BXXX |
| LOMBARDIA | BRESCIA | 12/04/2011 | OVICAPRINI | 063BXXX |
| LOMBARDIA | BRESCIA | 10/04/2011 | OVICAPRINI | 007BXXX |
| LOMBARDIA | BERGAMO | 08/02/2011 | OVICAPRINI | 143BXXX |
| LOMBARDIA | BERGAMO | 08/02/2011 | OVICAPRINI | 143BXXX |
| LOMBARDIA | BERGAMO | 03/04/2011 | OVICAPRINI | 016BXXX |
| LOMBARDIA | BERGAMO | 03/04/2011 | OVICAPRINI | 050BXXX |
| LOMBARDIA | BERGAMO | 10/04/2011 | OVICAPRINI | 007BXXX |
| LOMBARDIA | BERGAMO | 12/04/2011 | OVICAPRINI | 063BXXX |
| LOMBARDIA | BERGAMO | 02/04/2012 | OVICAPRINI | 016BXXX |
| LOMBARDIA | BERGAMO | 05/04/2012 | OVICAPRINI | 054BXXX |
| LOMBARDIA | BERGAMO | 05/04/2012 | OVICAPRINI | 054BXXX |
| LOMBARDIA | BERGAMO | 19/06/2012 | OVICAPRINI | 142BXXX |
| LOMBARDIA | BERGAMO | 19/06/2012 | OVICAPRINI | 142BXXX |
| LOMBARDIA | BERGAMO | 19/06/2012 | OVICAPRINI | 142BXXX |
| LOMBARDIA | BRIANZA | 16/07/2012 | OVICAPRINI | 166BXXX |
| LOMBARDIA | BRIANZA | 08/11/2012 | OVICAPRINI | 143BXXX |
| LOMBARDIA | BRIANZA | 06/12/2012 | OVICAPRINI | 142BXXX |
| LOMBARDIA | BRIANZA | 06/12/2012 | OVICAPRINI | 142BXXX |
| LOMBARDIA | BRIANZA | 06/12/2012 | OVICAPRINI | 142BXXX |
| LOMBARDIA | BRIANZA | 15/01/2013 | OVICAPRINI | 143BXXX |
| ABRUZZO | AZ-SU-AQ | 08/01/2013 | OVICAPRINI | 035PXXX |
| ABRUZZO | AZ-SU-AQ | 08/01/2013 | OVICAPRINI | 035PXXX |
| ABRUZZO | AZ-SU-AQ | 08/01/2013 | OVICAPRINI | 044RXXX |
| ABRUZZO | AZ-SU-AQ | 11/01/2013 | OVICAPRINI | 072AXXX |
| ABRUZZO | AZ-SU-AQ | 14/01/2013 | OVICAPRINI | 045RXXX |
| ABRUZZO | AZ-SU-AQ | 14/01/2013 | OVICAPRINI | 049AXXX |
| ABRUZZO | AZ-SU-AQ | 14/01/2013 | OVICAPRINI | 072AXXX |
| ABRUZZO | AZ-SU-AQ | 25/01/2013 | OVICAPRINI | 049AXXX |
| ABRUZZO | AZ-SU-AQ | 15/04/2013 | OVICAPRINI | 015RXXX |
| ABRUZZO | AZ-SU-AQ | 15/04/2013 | OVICAPRINI | 091RXXX |
| ABRUZZO | AZ-SU-AQ | 15/04/2013 | OVICAPRINI | 091RXXX |
| ABRUZZO | AZ-SU-AQ | 17/06/2013 | OVICAPRINI | 037RXXX |
| ABRUZZO | AZ-SU-AQ | 30/07/2013 | OVICAPRINI | 059VXXX |
| ABRUZZO | AZ-SU-AQ | 16/09/2013 | OVICAPRINI | 034PXXX |
| ABRUZZO | AZ-SU-AQ | 19/09/2013 | OVICAPRINI | 034PXXX |
| ABRUZZO | AZ-SU-AQ | 14/01/2014 | OVICAPRINI | 056AXXX |
| ABRUZZO | AZ-SU-AQ | 18/02/2014 | OVICAPRINI | 049AXXX |
| ABRUZZO | AZ-SU-AQ | 18/02/2014 | OVICAPRINI | 056AXXX |
| ABRUZZO | AZ-SU-AQ | 08/04/2014 | OVICAPRINI | 056AXXX |
| ABRUZZO | AZ-SU-AQ | 16/04/2014 | OVICAPRINI | 060AXXX |
| ABRUZZO | AZ-SU-AQ | 11/06/2014 | OVICAPRINI | 026AXXX |
| ABRUZZO | AZ-SU-AQ | 25/06/2014 | OVICAPRINI | 026AXXX |
| ABRUZZO | AZ-SU-AQ | 23/07/2014 | OVICAPRINI | 060AXXX |
| ABRUZZO | AZ-SU-AQ | 30/07/2014 | OVICAPRINI | 026AXXX |
| ABRUZZO | AZ-SU-AQ | 14/01/2015 | OVICAPRINI | 014AXXX |
| ABRUZZO | AZ-SU-AQ | 20/01/2015 | OVICAPRINI | 021AXXX |
| ABRUZZO | AZ-SU-AQ | 21/01/2015 | OVICAPRINI | 026AXXX |
| ABRUZZO | AZ-SU-AQ | 04/02/2015 | OVICAPRINI | 026AXXX |
| ABRUZZO | AZ-SU-AQ | 25/02/2015 | OVICAPRINI | 026AXXX |
| ABRUZZO | AZ-SU-AQ | 16/03/2015 | OVICAPRINI | 067AXXX |
| ABRUZZO | AZ-SU-AQ | 24/03/2015 | OVICAPRINI | 056AXXX |
| ABRUZZO | AZ-SU-AQ | 30/03/2015 | OVICAPRINI | 060AXXX |
| ABRUZZO | AZ-SU-AQ | 01/04/2015 | OVICAPRINI | 088AXXX |
| ABRUZZO | AZ-SU-AQ | 08/04/2015 | OVICAPRINI | 021AXXX |
| ABRUZZO | AZ-SU-AQ | 08/04/2015 | OVICAPRINI | 056AXXX |
| ABRUZZO | AZ-SU-AQ | 05/05/2015 | OVICAPRINI | 021AXXX |
| ABRUZZO | AZ-SU-AQ | 05/05/2015 | OVICAPRINI | 056AXXX |
| ABRUZZO | AZ-SU-AQ | 09/06/2015 | OVICAPRINI | 049AXXX |
| ABRUZZO | AZ-SU-AQ | 09/06/2015 | OVICAPRINI | 100AXXX |
| ABRUZZO | AZ-SU-AQ | 10/06/2015 | OVICAPRINI | 026AXXX |
| ABRUZZO | AZ-SU-AQ | 10/06/2015 | OVICAPRINI | 026AXXX |
| ABRUZZO | AZ-SU-AQ | 11/08/2015 | OVICAPRINI | 067AXXX |
| ABRUZZO | AZ-SU-AQ | 09/09/2015 | OVICAPRINI | 049AXXX |
| ABRUZZO | AZ-SU-AQ | 04/11/2015 | OVICAPRINI | 026AXXX |
| ABRUZZO | AZ-SU-AQ | 23/11/2015 | OVICAPRINI | 059VXXX |
| ABRUZZO | AZ-SU-AQ | 23/11/2015 | OVICAPRINI | 067AXXX |
| ABRUZZO | AZ-SU-AQ | 06/07/1905 | OVICAPRINI | 006AXXX |
| ABRUZZO | AZ-SU-AQ | 06/07/1905 | OVICAPRINI | 006AXXX |
| ABRUZZO | AZ-SU-AQ | 06/07/1905 | OVICAPRINI | 006AXXX |
| ABRUZZO | AZ-SU-AQ | 06/07/1905 | OVICAPRINI | 006AXXX |
| ABRUZZO | AZ-SU-AQ | 06/07/1905 | OVICAPRINI | 006AXXX |
| ABRUZZO | AZ-SU-AQ | 06/07/1905 | OVICAPRINI | 006AXXX |
| ABRUZZO | AZ-SU-AQ | 06/07/1905 | OVICAPRINI | 006AXXX |
| ABRUZZO | AZ-SU-AQ | 06/07/1905 | OVICAPRINI | 006AXXX |
| ABRUZZO | AZ-SU-AQ | 06/07/1905 | OVICAPRINI | 006AXXX |
| ABRUZZO | AZ-SU-AQ | 06/07/1905 | OVICAPRINI | 006AXXX |
| ABRUZZO | AZ-SU-AQ | 06/07/1905 | OVICAPRINI | 006AXXX |
| ABRUZZO | AZ-SU-AQ | 06/07/1905 | OVICAPRINI | 006AXXX |
| ABRUZZO | AZ-SU-AQ | 06/07/1905 | OVICAPRINI | 006AXXX |
| ABRUZZO | AZ-SU-AQ | 06/07/1905 | OVICAPRINI | 006AXXX |
| ABRUZZO | AZ-SU-AQ | 06/07/1905 | OVICAPRINI | 006AXXX |
| ABRUZZO | AZ-SU-AQ | 06/07/1905 | OVICAPRINI | 006AXXX |
| ABRUZZO | AZ-SU-AQ | 06/07/1905 | OVICAPRINI | 006AXXX |
| ABRUZZO | AZ-SU-AQ | 06/07/1905 | OVICAPRINI | 006AXXX |
| ABRUZZO | AZ-SU-AQ | 06/07/1905 | OVICAPRINI | 006AXXX |
| ABRUZZO | AZ-SU-AQ | 06/07/1905 | OVICAPRINI | 032AXXX |
| ABRUZZO | AZ-SU-AQ | 06/07/1905 | OVICAPRINI | 032AXXX |
| ABRUZZO | AZ-SU-AQ | 06/07/1905 | OVICAPRINI | 032AXXX |
| ABRUZZO | AZ-SU-AQ | 06/07/1905 | OVICAPRINI | 032AXXX |
| ABRUZZO | AZ-SU-AQ | 06/07/1905 | OVICAPRINI | 032AXXX |
| ABRUZZO | AZ-SU-AQ | 06/07/1905 | OVICAPRINI | 032AXXX |
| ABRUZZO | AZ-SU-AQ | 06/07/1905 | OVICAPRINI | 047AXXX |
| ABRUZZO | AZ-SU-AQ | 06/07/1905 | OVICAPRINI | 047AXXX |
| ABRUZZO | AZ-SU-AQ | 06/07/1905 | OVICAPRINI | 047AXXX |
| ABRUZZO | AZ-SU-AQ | 06/07/1905 | OVICAPRINI | 047AXXX |
| ABRUZZO | AZ-SU-AQ | 06/07/1905 | OVICAPRINI | 047AXXX |
| ABRUZZO | AZ-SU-AQ | 06/07/1905 | OVICAPRINI | 047AXXX |
| ABRUZZO | AZ-SU-AQ | 06/07/1905 | OVICAPRINI | 054AXXX |
| ABRUZZO | AZ-SU-AQ | 06/07/1905 | OVICAPRINI | 054AXXX |
| ABRUZZO | AZ-SU-AQ | 06/07/1905 | OVICAPRINI | 054AXXX |
| ABRUZZO | AZ-SU-AQ | 06/07/1905 | OVICAPRINI | 054AXXX |
| ABRUZZO | AZ-SU-AQ | 06/07/1905 | OVICAPRINI | 054AXXX |
| ABRUZZO | AZ-SU-AQ | 06/07/1905 | OVICAPRINI | 054AXXX |
| ABRUZZO | AZ-SU-AQ | 06/07/1905 | OVICAPRINI | 054AXXX |
| ABRUZZO | AZ-SU-AQ | 06/07/1905 | OVICAPRINI | 054AXXX |
| ABRUZZO | AZ-SU-AQ | 06/07/1905 | OVICAPRINI | 054AXXX |
| ABRUZZO | AZ-SU-AQ | 06/07/1905 | OVICAPRINI | 054AXXX |
| ABRUZZO | AZ-SU-AQ | 06/07/1905 | OVICAPRINI | 054AXXX |
| ABRUZZO | AZ-SU-AQ | 06/07/1905 | OVICAPRINI | 054AXXX |
| ABRUZZO | AZ-SU-AQ | 06/07/1905 | OVICAPRINI | 054AXXX |
| ABRUZZO | AZ-SU-AQ | 06/07/1905 | OVICAPRINI | 054AXXX |
| ABRUZZO | AZ-SU-AQ | 06/07/1905 | OVICAPRINI | 054AXXX |
| ABRUZZO | AZ-SU-AQ | 06/07/1905 | OVICAPRINI | 054AXXX |
| ABRUZZO | AZ-SU-AQ | 06/07/1905 | OVICAPRINI | 054AXXX |
| ABRUZZO | AZ-SU-AQ | 06/07/1905 | OVICAPRINI | 054AXXX |
| ABRUZZO | AZ-SU-AQ | 06/07/1905 | OVICAPRINI | 054AXXX |
| ABRUZZO | AZ-SU-AQ | 06/07/1905 | OVICAPRINI | 054AXXX |
| ABRUZZO | AZ-SU-AQ | 06/07/1905 | OVICAPRINI | 054AXXX |
| ABRUZZO | AZ-SU-AQ | 06/07/1905 | OVICAPRINI | 054AXXX |
| ABRUZZO | AZ-SU-AQ | 06/07/1905 | OVICAPRINI | 054AXXX |
| ABRUZZO | AZ-SU-AQ | 06/07/1905 | OVICAPRINI | 054AXXX |
| ABRUZZO | AZ-SU-AQ | 06/07/1905 | OVICAPRINI | 080AXXX |
| ABRUZZO | AZ-SU-AQ | 06/07/1905 | OVICAPRINI | 080AXXX |
| ABRUZZO | AZ-SU-AQ | 06/07/1905 | OVICAPRINI | 080AXXX |
| ABRUZZO | AZ-SU-AQ | 06/07/1905 | OVICAPRINI | 080AXXX |
| ABRUZZO | AZ-SU-AQ | 06/07/1905 | OVICAPRINI | 085AXXX |
| ABRUZZO | AZ-SU-AQ | 06/07/1905 | OVICAPRINI | 085AXXX |
| ABRUZZO | AZ-SU-AQ | 06/07/1905 | OVICAPRINI | 085AXXX |
| ABRUZZO | AZ-SU-AQ | 06/07/1905 | OVICAPRINI | 085AXXX |
| ABRUZZO | AZ-SU-AQ | 06/07/1905 | OVICAPRINI | 085AXXX |
| ABRUZZO | AZ-SU-AQ | 06/07/1905 | OVICAPRINI | 085AXXX |
| ABRUZZO | AZ-SU-AQ | 06/07/1905 | OVICAPRINI | 085AXXX |
| ABRUZZO | AZ-SU-AQ | 06/07/1905 | OVICAPRINI | 085AXXX |
| ABRUZZO | AZ-SU-AQ | 06/07/1905 | OVICAPRINI | 085AXXX |
| ABRUZZO | AZ-SU-AQ | 06/07/1905 | OVICAPRINI | 085AXXX |
| ABRUZZO | AZ-SU-AQ | 06/07/1905 | OVICAPRINI | 085AXXX |
| ABRUZZO | AZ-SU-AQ | 06/07/1905 | OVICAPRINI | 085AXXX |
| ABRUZZO | AZ-SU-AQ | 06/07/1905 | OVICAPRINI | 085AXXX |
| ABRUZZO | AZ-SU-AQ | 06/07/1905 | OVICAPRINI | 085AXXX |
| ABRUZZO | AZ-SU-AQ | 06/07/1905 | OVICAPRINI | 096AXXX |
| ABRUZZO | AZ-SU-AQ | 06/07/1905 | OVICAPRINI | 096AXXX |
| ABRUZZO | AZ-SU-AQ | 06/07/1905 | OVICAPRINI | 096AXXX |
| ABRUZZO | AZ-SU-AQ | 06/07/1905 | OVICAPRINI | 096AXXX |
| ABRUZZO | AZ-SU-AQ | 07/07/1905 | OVICAPRINI | 006AXXX |
| ABRUZZO | AZ-SU-AQ | 07/07/1905 | OVICAPRINI | 006AXXX |
| ABRUZZO | AZ-SU-AQ | 07/07/1905 | OVICAPRINI | 006AXXX |
| ABRUZZO | AZ-SU-AQ | 07/07/1905 | OVICAPRINI | 006AXXX |
| ABRUZZO | AZ-SU-AQ | 07/07/1905 | OVICAPRINI | 006AXXX |
| ABRUZZO | AZ-SU-AQ | 07/07/1905 | OVICAPRINI | 006AXXX |
| ABRUZZO | AZ-SU-AQ | 07/07/1905 | OVICAPRINI | 018AXXX |
| ABRUZZO | AZ-SU-AQ | 07/07/1905 | OVICAPRINI | 032AXXX |
| ABRUZZO | AZ-SU-AQ | 07/07/1905 | OVICAPRINI | 051AXXX |
| ABRUZZO | AZ-SU-AQ | 07/07/1905 | OVICAPRINI | 054AXXX |
| ABRUZZO | AZ-SU-AQ | 07/07/1905 | OVICAPRINI | 054AXXX |
| ABRUZZO | AZ-SU-AQ | 07/07/1905 | OVICAPRINI | 054AXXX |
| ABRUZZO | AZ-SU-AQ | 07/07/1905 | OVICAPRINI | 054AXXX |
| ABRUZZO | AZ-SU-AQ | 07/07/1905 | OVICAPRINI | 054AXXX |
| ABRUZZO | AZ-SU-AQ | 07/07/1905 | OVICAPRINI | 058AXXX |
| ABRUZZO | AZ-SU-AQ | 07/07/1905 | OVICAPRINI | 058AXXX |
| ABRUZZO | AZ-SU-AQ | 07/07/1905 | OVICAPRINI | 058AXXX |
| ABRUZZO | AZ-SU-AQ | 07/07/1905 | OVICAPRINI | 058AXXX |
| ABRUZZO | AZ-SU-AQ | 07/07/1905 | OVICAPRINI | 058AXXX |
| ABRUZZO | AZ-SU-AQ | 07/07/1905 | OVICAPRINI | 058AXXX |
| ABRUZZO | AZ-SU-AQ | 07/07/1905 | OVICAPRINI | 058AXXX |
| ABRUZZO | AZ-SU-AQ | 07/07/1905 | OVICAPRINI | 058AXXX |
| ABRUZZO | AZ-SU-AQ | 07/07/1905 | OVICAPRINI | 058AXXX |
| ABRUZZO | AZ-SU-AQ | 07/07/1905 | OVICAPRINI | 058AXXX |
| ABRUZZO | AZ-SU-AQ | 07/07/1905 | OVICAPRINI | 069AXXX |
| ABRUZZO | AZ-SU-AQ | 07/07/1905 | OVICAPRINI | 080AXXX |
| ABRUZZO | AZ-SU-AQ | 07/07/1905 | OVICAPRINI | 085AXXX |
| ABRUZZO | AZ-SU-AQ | 07/07/1905 | OVICAPRINI | 085AXXX |
| ABRUZZO | AZ-SU-AQ | 07/07/1905 | OVICAPRINI | 085AXXX |
| ABRUZZO | AZ-SU-AQ | 07/07/1905 | OVICAPRINI | 093AXXX |
| ABRUZZO | AZ-SU-AQ | 07/07/1905 | OVICAPRINI | 096AXXX |
| LAZIO | AP | 07/07/1905 | OVICAPRINI | 021AXXX |
| LAZIO | AP | 07/07/1905 | OVICAPRINI | 032AXXX |
| LAZIO | AP | 07/07/1905 | OVICAPRINI | 036AXXX |
| LAZIO | PG | 07/07/1905 | OVICAPRINI | 043PXXX |
| LAZIO | RI | 07/07/1905 | OVICAPRINI | 051RXXX |
| LAZIO | RI | 07/07/1905 | OVICAPRINI | 051RXXX |
| LAZIO | AQ | 07/07/1905 | OVICAPRINI | 056AXXX |
| LAZIO | AQ | 07/07/1905 | OVICAPRINI | 072AXXX |
| LAZIO | PG | 07/07/1905 | OVICAPRINI | 031PXXX |
| LAZIO | RI | 07/07/1905 | OVICAPRINI | 033RXXX |
| LAZIO | RI | 07/07/1905 | OVICAPRINI | 033RXXX |
| LAZIO | AQ | 07/07/1905 | OVICAPRINI | 009AXXX |
| LAZIO | AQ | 07/07/1905 | OVICAPRINI | 049AXXX |
| LAZIO | AQ | 07/07/1905 | OVICAPRINI | 082AXXX |
| LAZIO | RI | 07/07/1905 | OVICAPRINI | 033RXXX |
| LAZIO | RI | 07/07/1905 | OVICAPRINI | 035RXXX |
| LAZIO | RI | 07/07/1905 | OVICAPRINI | 035RXXX |
| LAZIO | RM | 07/07/1905 | OVICAPRINI | 015RXXX |
| LAZIO | RM | 07/07/1905 | OVICAPRINI | 018RXXX |
| LAZIO | RM | 07/07/1905 | OVICAPRINI | 036RXXX |
| LAZIO | RM | 07/07/1905 | OVICAPRINI | 080RXXX |
| LAZIO | RM | 07/07/1905 | OVICAPRINI | 091RXXX |
| LAZIO | RM | 07/07/1905 | OVICAPRINI | 099RXXX |
| LAZIO | RM | 07/07/1905 | OVICAPRINI | 110RXXX |
| LAZIO | AQ | 07/07/1905 | OVICAPRINI | 013AXXX |
| LAZIO | RM | 07/07/1905 | OVICAPRINI | 047RXXX |
| LAZIO | VT | 07/07/1905 | OVICAPRINI | 021VXXX |
| UMBRIA | USL Umbria 2 | 17/09/2014 | OVICAPRINI | 005PXXX |
| UMBRIA | USL Umbria 2 | 17/09/2014 | OVICAPRINI | 005PXXX |
| UMBRIA | USL Umbria 2 | 17/09/2014 | OVICAPRINI | 005PXXX |
| UMBRIA | USL Umbria 2 | 17/09/2014 | OVICAPRINI | 005PXXX |
| UMBRIA | USL Umbria 2 | 17/09/2014 | OVICAPRINI | 005PXXX |
| UMBRIA | USL Umbria 2 | 17/09/2014 | OVICAPRINI | 005PXXX |
| UMBRIA | USL Umbria 2 | 17/09/2014 | OVICAPRINI | 005PXXX |
| UMBRIA | USL Umbria 2 | 17/09/2014 | OVICAPRINI | 005PXXX |
| UMBRIA | USL Umbria 2 | 17/09/2014 | OVICAPRINI | 005PXXX |
| UMBRIA | USL Umbria 2 | 17/09/2014 | OVICAPRINI | 005PXXX |
| UMBRIA | USL Umbria 2 | 17/09/2014 | OVICAPRINI | 005PXXX |
| UMBRIA | USL Umbria 2 | 17/09/2014 | OVICAPRINI | 005PXXX |
| UMBRIA | USL Umbria 2 | 17/09/2014 | OVICAPRINI | 005PXXX |
| UMBRIA | USL Umbria 2 | 17/09/2014 | OVICAPRINI | 005PXXX |
| UMBRIA | USL Umbria 2 | 17/09/2014 | OVICAPRINI | 005PXXX |
| UMBRIA | USL Umbria 2 | 17/09/2014 | OVICAPRINI | 005PXXX |
| UMBRIA | USL Umbria 2 | 17/09/2014 | OVICAPRINI | 005PXXX |
| UMBRIA | USL Umbria 2 | 17/09/2014 | OVICAPRINI | 005PXXX |
| UMBRIA | USL Umbria 2 | 17/09/2014 | OVICAPRINI | 005PXXX |
| UMBRIA | USL Umbria 2 | 17/09/2014 | OVICAPRINI | 005PXXX |
| UMBRIA | USL Umbria 2 | 17/09/2014 | OVICAPRINI | 005PXXX |
| UMBRIA | USL Umbria 2 | 03/12/2012 | OVICAPRINI | 005PXXX |
| UMBRIA | USL Umbria 2 | 03/12/2012 | OVICAPRINI | 005PXXX |
| UMBRIA | USL Umbria 2 | 03/12/2012 | OVICAPRINI | 005PXXX |
| UMBRIA | USL Umbria 2 | 03/12/2012 | OVICAPRINI | 005PXXX |
| UMBRIA | USL Umbria 2 | 07/11/2012 | OVICAPRINI | 005PXXX |
| UMBRIA | USL Umbria 2 | 07/11/2012 | OVICAPRINI | 005PXXX |
| UMBRIA | USL Umbria 2 |  | OVICAPRINI | 051PXXX |
| UMBRIA | USL Umbria 2 |  | OVICAPRINI | 051PXXX |
| UMBRIA | USL Umbria 2 |  | OVICAPRINI | 051PXXX |
| UMBRIA | USL Umbria 2 |  | OVICAPRINI | 051PXXX |
| UMBRIA | USL Umbria 2 |  | OVICAPRINI | 035PXXX |
| UMBRIA | USL Umbria 2 |  | OVICAPRINI | 035PXXX |
| VENETO | F103 | 05/07/1905 | OVICAPRINI | 039VXXX |
| VENETO | F103 | 05/07/1905 | OVICAPRINI | 054VXXX |
| VENETO | F103 | 05/07/1905 | OVICAPRINI | 087VXXX |
| VENETO | F103 | 05/07/1905 | OVICAPRINI | 087VXXX |
| VENETO | F104 | 05/07/1905 | OVICAPRINI | 024VXXX |
| VENETO | F104 | 05/07/1905 | OVICAPRINI | 055VXXX |
| VENETO | F104 | 05/07/1905 | OVICAPRINI | 055VXXX |
| VENETO | F104 | 05/07/1905 | OVICAPRINI | 063VXXX |
| VENETO | F104 | 05/07/1905 | OVICAPRINI | 096VXXX |
| VENETO | F104 | 05/07/1905 | OVICAPRINI | 107VXXX |
| VENETO | F104 | 05/07/1905 | OVICAPRINI | 118VXXX |
| VENETO | F105 | 05/07/1905 | OVICAPRINI | 061VXXX |
| VENETO | F105 | 05/07/1905 | OVICAPRINI | 084VXXX |
| VENETO | F106 | 05/07/1905 | OVICAPRINI | 018VXXX |
| VENETO | F115 | 05/07/1905 | OVICAPRINI | 077PXXX |
| VENETO | F120 | 05/07/1905 | OVICAPRINI | 002VXXX |
| VENETO | F120 | 05/07/1905 | OVICAPRINI | 067VXXX |
| VENETO | F120 | 05/07/1905 | OVICAPRINI | 080VXXX |
| VENETO | F122 | 05/07/1905 | OVICAPRINI | 058VXXX |
| VENETO | F122 | 05/07/1905 | OVICAPRINI | 060VXXX |
| VENETO | F122 | 05/07/1905 | OVICAPRINI | 079VXXX |
| VENETO | F122 | 05/07/1905 | OVICAPRINI | 079VXXX |
| VENETO | F122 | 05/07/1905 | OVICAPRINI | 089VXXX |
| VENETO | F122 | 05/07/1905 | OVICAPRINI | 089VXXX |
| VENETO | F122 | 05/07/1905 | OVICAPRINI | 096VXXX |
| VENETO | F105 | 06/07/1905 | OVICAPRINI | 110VXXX |
| VENETO | F106 | 06/07/1905 | OVICAPRINI | 002VXXX |
| VENETO | F106 | 06/07/1905 | OVICAPRINI | 035VXXX |
| VENETO | F106 | 06/07/1905 | OVICAPRINI | 048VXXX |
| VENETO | F106 | 06/07/1905 | OVICAPRINI | 048VXXX |
| VENETO | F108 | 06/07/1905 | OVICAPRINI | 004TXXX |
| VENETO | F108 | 06/07/1905 | OVICAPRINI | 024TXXX |
| VENETO | F108 | 06/07/1905 | OVICAPRINI | 056TXXX |
| VENETO | F108 | 06/07/1905 | OVICAPRINI | 077TXXX |
| VENETO | F108 | 06/07/1905 | OVICAPRINI | 077TXXX |
| VENETO | F120 | 06/07/1905 | OVICAPRINI | 038VXXX |
| VENETO | F120 | 06/07/1905 | OVICAPRINI | 080VXXX |
| VENETO | F120 | 06/07/1905 | OVICAPRINI | 091VXXX |
| VENETO | F122 | 06/07/1905 | OVICAPRINI | 057VXXX |
| VENETO | F122 | 06/07/1905 | OVICAPRINI | 060VXXX |
| VENETO | F122 | 06/07/1905 | OVICAPRINI | 060VXXX |
| VENETO | F122 | 06/07/1905 | OVICAPRINI | 078VXXX |
| VENETO | F122 | 06/07/1905 | OVICAPRINI | 089VXXX |
| VENETO | F122 | 06/07/1905 | OVICAPRINI | 094VXXX |
| VENETO | F106 | 07/07/1905 | OVICAPRINI | 055VXXX |
| VENETO | F106 | 07/07/1905 | OVICAPRINI | 009VXXX |
| VENETO | F106 | 07/07/1905 | OVICAPRINI | 009VXXX |
| VENETO | F106 | 07/07/1905 | OVICAPRINI | 009VXXX |
| VENETO | F106 | 07/07/1905 | OVICAPRINI | 033VXXX |
| VENETO | F106 | 07/07/1905 | OVICAPRINI | 033VXXX |
| VENETO | F106 | 07/07/1905 | OVICAPRINI | 041VXXX |
| VENETO | F106 | 07/07/1905 | OVICAPRINI | 042VXXX |
| VENETO | F106 | 07/07/1905 | OVICAPRINI | 054VXXX |
| VENETO | F106 | 07/07/1905 | OVICAPRINI | 058VXXX |
| VENETO | F106 | 07/07/1905 | OVICAPRINI | 087VXXX |
| VENETO | F106 | 07/07/1905 | OVICAPRINI | 055VXXX |
| VENETO | F106 | 07/07/1905 | OVICAPRINI | 096VXXX |
| VENETO | F106 | 07/07/1905 | OVICAPRINI | 118VXXX |
| VENETO | F106 | 07/07/1905 | OVICAPRINI | 119VXXX |
| VENETO | F106 | 07/07/1905 | OVICAPRINI | 084VXXX |
| VENETO | F106 | 07/07/1905 | OVICAPRINI | 001VXXX |
| VENETO | F106 | 07/07/1905 | OVICAPRINI | 013VXXX |
| VENETO | F106 | 07/07/1905 | OVICAPRINI | 018VXXX |
| VENETO | F106 | 07/07/1905 | OVICAPRINI | 067VXXX |
| VENETO | F106 | 07/07/1905 | OVICAPRINI | 004TXXX |
| VENETO | F106 | 07/07/1905 | OVICAPRINI | 004TXXX |
| VENETO | F106 | 07/07/1905 | OVICAPRINI | 024TXXX |
| VENETO | F106 | 07/07/1905 | OVICAPRINI | 029TXXX |
| VENETO | F106 | 07/07/1905 | OVICAPRINI | 085TXXX |
| VENETO | F106 | 07/07/1905 | OVICAPRINI | 023PXXX |
| VENETO | F106 | 07/07/1905 | OVICAPRINI | 078PXXX |
| VENETO | F106 | 07/07/1905 | OVICAPRINI | 078PXXX |
| VENETO | F106 | 07/07/1905 | OVICAPRINI | 039VXXX |
| VENETO | F106 | 07/07/1905 | OVICAPRINI | 067VXXX |
| VENETO | F106 | 07/07/1905 | OVICAPRINI | 067VXXX |
| VENETO | F106 | 07/07/1905 | OVICAPRINI | 067VXXX |
| VENETO | F106 | 07/07/1905 | OVICAPRINI | 070VXXX |
| VENETO | F106 | 07/07/1905 | OVICAPRINI | 018VXXX |
| VENETO | F106 | 07/07/1905 | OVICAPRINI | 035VXXX |
| VENETO | F106 | 07/07/1905 | OVICAPRINI | 078VXXX |
| VENETO | F106 | 07/07/1905 | OVICAPRINI | 079VXXX |
| VENETO | F106 | 07/07/1905 | OVICAPRINI | 089VXXX |
