# Supplementary material for: Prevalence estimation of Italian ovine cystic echinococcosis in slaughterhouses: A retrospective Bayesian data analysis, 2010–2015
Source: PLoS One. 2019 Apr 1;14(4):e0214224. doi: 10.1371/journal.pone.0214224 (PMC6443144; doi:10.1371/journal.pone.0214224)
Supplement: S4 Table — Data regarding the number of farms that move animals for slaughter were available only for the Sardinian region. All data are stratified by study year. (DOCX) [file pone.0214224.s005.docx]

**S4 Table.** **Contribution of each Regional Veterinary Epidemiology Observatory (OEVR) in data collection, as number of the total farms by the National Database and the number of cystic echinococcosis (CE) positive sheep and goats found in slaughterhouses. Data regarding the number of farms that move animals for slaughter were available only for the Sardinian region. All data are stratified by study year.**

| **OEVRs** | **Region** | **Year** | **Total farms** | **Farm to slaughterhouse** | **CE-positive** |
| --- | --- | --- | --- | --- | --- |
| OEVR – SARDEGNA | Sardinia | 2010 | 16.832 | 3.560 | 962 |
|  |  | 2011 | 16.650 | 3.956 | 811 |
|  |  | 2012 | 16.282 | 3.786 | 1.081 |
|  |  | 2013 | 16.340 | 3.585 | 706 |
|  |  | 2014 | 16.247 | 3.390 | 441 |
|  |  | 2015 | 15.968 | 3.352 | 326 |
| OEVR – LAZIO | Lazio | 2010 | 7.953 | NOT AVAILABLE DATA  NOT AVAILABLE DATA | - |
|  |  | 2011 | 7.948 |  | 148 |
|  |  | 2012 | 7.934 |  | 30 |
|  |  | 2013 | 7.543 |  | 91 |
|  |  | 2014 | 7.436 |  | 0 |
|  |  | 2015 | 7.531 |  | 17 |
| OEVR - MEZZOGIORNO | Puglia | 2010 | 3.135 |  | - |
|  |  | 2011 | 3.062 |  | - |
|  |  | 2012 | 3.516 |  | - |
|  |  | 2013 | 3.312 |  | - |
|  |  | 2014 | 3.130 |  | - |
|  |  | 2015 | 3.163 |  | 6 |
|  | Basilicata | 2010 | 5.956 |  |  |
|  |  | 2011 | 6.017 |  | - |
|  |  | 2012 | 5.830 |  | - |
|  |  | 2013 | 5.795 |  | - |
|  |  | 2014 | 5.638 |  | 1 |
|  |  | 2015 | 5.586 |  | 45 |
|  | Calabria | 2010 | 7.347 |  | - |
|  |  | 2011 | 7.271 |  | - |
|  |  | 2012 | 7.160 |  | - |
|  |  | 2013 | 6.539 |  | - |
|  |  | 2014 | 6.435 |  | - |
|  |  | 2015 | 6.518 |  | - |
|  | Campania | 2010 | 3.992 |  | - |
|  |  | 2011 | 4.166 |  | - |
|  |  | 2012 | 5.917 |  | 2 |
|  |  | 2013 | 5.778 |  | - |
|  |  | 2014 | 5.869 |  | 8 |
|  |  | 2015 | 5.822 |  | 143 |
| OEVR – EMILIA ROMAGNA | Emilia Romagna | 2010 | 2.428 |  | 1 |
|  |  | 2011 | 2.402 |  | 10 |
|  |  | 2012 | 2.376 |  | 6 |
|  |  | 2013 | 2.372 |  | 3 |
|  |  | 2014 | 2.328 |  | 3 |
|  |  | 2015 | 2.295 |  | 1 |
| OEVR – PIEMONTE | Piemonte | 2010 | 4.426 |  | 5 |
|  |  | 2011 | 4.593 |  | 0 |
|  |  | 2012 | 4.171 |  | 76 |
|  |  | 2013 | 4.323 |  | 45 |
|  |  | 2014 | 4.501 |  | 68 |
|  |  | 2015 | 4.616 |  | 113 |
| OEVR – UMBRIA E MARCHE | Marche | 2010 | 2.893 |  | - |
|  |  | 2011 | 2.972 |  | 1 |
|  |  | 2012 | 3.521 |  | 1 |
|  |  | 2013 | 3.312 |  | 1 |
|  |  | 2014 | 3.180 |  | 1 |
|  |  | 2015 | 3.110 |  | 6 |
|  | Umbria | 2010 | 2.986 |  | 3 |
|  |  | 2011 | 3.175 |  | 1 |
|  |  | 2012 | 4.837 |  | 7 |
|  |  | 2013 | 5.158 |  | 6 |
|  |  | 2014 | 5.258 |  | 22 |
|  |  | 2015 | 5.332 |  | 3 |
| OEVR – LOMBARDIA | Lombardia | 2010 | 4.096 |  | - |
|  |  | 2011 | 4.526 |  | 10 |
|  |  | 2012 | 5.219 |  | 22 |
|  |  | 2013 | 4.998 |  | 2 |
|  |  | 2014 | 4.970 |  | - |
|  |  | 2015 | 5.021 |  | - |
| OEVR - VENETO | Veneto | 2010 | 1.843 |  | - |
|  |  | 2011 | 1.860 |  | - |
|  |  | 2012 | 1.989 |  | - |
|  |  | 2013 | 2.029 |  | 25 |
|  |  | 2014 | 2.110 |  | 19 |
|  |  | 2015 | 2.209 |  | 38 |
| OEVR - ABRUZZO | Abruzzo | 2010 | 6.165 |  | - |
|  |  | 2011 | 6.234 |  | - |
|  |  | 2012 | 6.008 |  | - |
|  |  | 2013 | 5.861 |  | 4 |
|  |  | 2014 | 5.397 |  | 86 |
|  |  | 2015 | 5.228 |  | 58 |
|  | Molise | 2010 | 3.214 |  | - |
|  |  | 2011 | 3.059 |  | - |
|  |  | 2012 | 3.510 |  | - |
|  |  | 2013 | 3.411 |  | - |
|  |  | 2014 | 3.176 |  | - |
|  |  | 2015 | 2.861 |  | 10 |
