# Supplementary material for: Prevalence estimation of Italian ovine cystic echinococcosis in slaughterhouses: A retrospective Bayesian data analysis, 2010–2015
Source: PLoS One. 2019 Apr 1;14(4):e0214224. doi: 10.1371/journal.pone.0214224 (PMC6443144; doi:10.1371/journal.pone.0214224)
Supplement: S5 Table — (DOCX) [file pone.0214224.s006.docx]

**S5 Table.** **Posterior inclusion probabilities for the variables assessed in the Bayesian variable selection procedure**

| **VARIABLE** | **INCLUSION PROBABILITY** | **HPM** | **MPM** | **FINAL DECISION: Inclusion/Exclusion** |
| --- | --- | --- | --- | --- |
| N. farms | 0.6577 | * | * | Yes |
| N. animals | 0.7962 | * | * | Yes |
| Human cases | 0.5434 | * | * | Yes |
| Age of the farmer | 0.8783 | * | * | Yes |
| % female farm owner | 0.8456 | * | * | Yes |
| Burnt forests (Ind_255) | 0.4965 | * |  | Not |
| Flood risk population (Ind_278) | 0.5312 | * | * | Yes |
| Cultural demand (Ind_018) | 0.7252 | * | * | Yes |
| Weight of cooperative society (Ind_120) | 0.4579 | * |  | Not |
| Air quality monitoring (Ind_265) | 0.3941 |  |  | Not |
| Enrollment rate in the business register (Ind_242) | 0.452 | * |  | Not |
| Ability to export in sectors with dynamic global demand (Ind_168) | 0.7659 | * | * | Yes |
| Unemployment rate (Ind_012) | 0.5685 | * | * | Yes |
| Employment rate (Ind_013) | 0.4633 | * |  | Not |
| Difference between male and female employment rate (Ind_057) | 0.4861 | * |  | Not |
| Participation of the population in the labor market (Ind_108) | 0.5705 | * | * | Yes |
| Homicide rate (Ind_281) | 0.6546 | * | * | Yes |
| Micro criminality index (Ind_134) | 0.6943 | * | * | Yes |
| Funding risk (Ind_162) | 0.2523 |  |  | Not |
| Municipal waste (Ind_083) | 0.8187 | * | * | Yes |
| Childhood services (Ind_142) | 0.481 | * |  | Not |
| Taking charge of all users of childcare services (Ind_414) | 0.492 | * |  | Not |
| Hospital emigration (Ind_141) | 0.5217 | * | * | Yes |
| Tourism rate (Ind_105) | 0.5137 | * | * | Yes |
